# Epidemiologic and survival analysis of malignant peripheral nerve sheath tumors: a retrospective cohort study

**DOI:** 10.1097/JS9.0000000000001756

**Published:** 2024-06-27

**Authors:** Jingxuan Huang, Haoting Shi, Lingling Ge, Beiyao Zhu, Jun Liu, Xue Wang, Zheshen Han, Qingfeng Li, Zhichao Wang

**Affiliations:** a Department of Plastic and Reconstructive Surgery, Shanghai Ninth People’s Hospital, Shanghai Jiao Tong University School of Medicine; b Neurofibromatosis Type 1 Center and Laboratory for Neurofibromatosis Type 1 Research, Shanghai Ninth People’s Hospital, Shanghai Jiao Tong University School of Medicine; c Ruijin Hospital, Shanghai Jiao Tong University School of Medicine, Shanghai; d School of Public Health, The University of Hong Kong, Hong Kong, People’s Republic of China

Highlights

- The incidence remains constant among non-White individuals and those at younger ages.
- Surgical intervention was identified as the most efficacious for malignant peripheral nerve sheath tumor (MPNST).
- Survivors are confronted with elevated risk of subsequent cancer and co-morbidities.
- Findings may inform tailored strategies and long-term surveillance for MPNST.

Malignant peripheral nerve sheath tumor (MPNST) is an aggressive soft-tissue sarcoma that originates from Schwann cells or pluripotent cells of the neural crest origin^1,2^. Here, leveraging the population-based incidence data in the US, we sought to characterize the updated epidemiological and survival profile of MPNST and describe the risk pattern of developing subsequent primary cancers (SPCs) and cause of death.

In this population-based, retrospective cohort study, we sourced national data from the Surveillance, Epidemiology, and End Result (SEER) database. Patients with MPNST during 1975–2019 were included in this study (Supplementary Figure 1, Supplemental Digital Content 1, http://links.lww.com/JS9/C899). This study adhered to the principles of the Declaration of Helsinki and the Strengthening the Reporting of cohort, cross-sectional, and case–control studies in Surgery (STROCSS, Supplemental Digital Content 2, http://links.lww.com/JS9/C900)^3^. More details regarding methodology were shown in Supplementary Methods (Supplemental Digital Content 1, http://links.lww.com/JS9/C899) and Supplementary Table 1 (Supplemental Digital Content 1, http://links.lww.com/JS9/C899).

Using SEER 17, we analyzed the incidence distribution of MPNST among different subgroups (Supplementary Figure 2, Supplemental Digital Content 1, http://links.lww.com/JS9/C899 and Table 2–6, Supplemental Digital Content 1, http://links.lww.com/JS9/C899). MPNST incidences by sex rose with age (*P*<.05). There was not significant difference in incidence between male and female under the age of 55, except for the 20–29 age group. Compared to non-Hispanic White (NHW), MPNST was more common in non-Hispanic Black (NHB), but rare in non-Hispanic Asian/Pacific Islander (NHAPI) and Hispanic.

For the incidence trend, the age-standardized incidence of MPNST significantly decreased during 2000–2019 (APC, −2.2%) (Fig. 1 and Supplementary Table 7, Supplemental Digital Content 1, http://links.lww.com/JS9/C899). The incidence declined substantially among those aged ≥40 years, but levelled off among those aged <20 years and aged 20–39 years. The incidence slightly shifted among NHB, NHAPI and Hispanic, but declined significantly among NHW (APC, −2.7%). There was an unequal decline in the incidence of high-grade MPNST, but the incidence remained stable for the low-grade subgroup. In addition, there was a nonsignificant rise for distant MPNST (APC, 0.3%), whereas a flattening trend for localized MPNST and a dip for regional MPNST (APC, −3.5%), which was mainly driven by those <40 years (Supplementary Table 8–9, Supplemental Digital Content 1, http://links.lww.com/JS9/C899).

**Figure 1:**
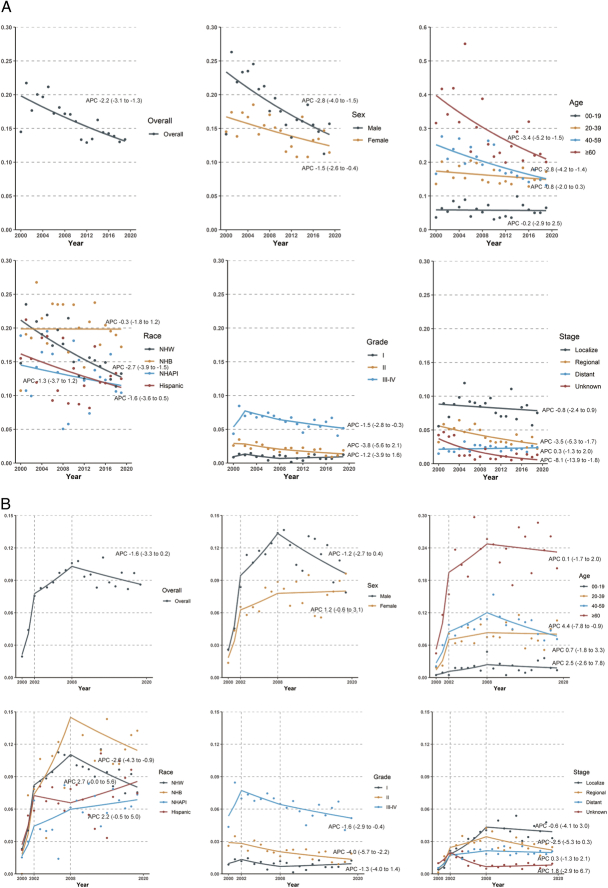
Trends of incidence and mortality rates for malignant peripheral nerve sheath tumor. The incidence (A) and mortality (B) trend in the 17 SEER registries was fit by joinpoint regression, and the APC was calculated. Filled circles represent observed age-adjusted rates; dotted lines represent the modeled age-adjusted rate. APC, annual percentage change; NHW, non-Hispanic White; NHB, non-Hispanic Black; NHAPI, non-Hispanic Asian and Pacific Islander. Notes: The mortality rate was age-adjusted and adjusted for reporting delay (race and cancer type), where the 95% CI was calculated using Tiwari’s method. The analyses of race limited in non-Hispanic White, non-Hispanic Black, non-Hispanic Asian, and Pacific Islander and Hispanic. Participants with non-Hispanic American Indian/Alaska Native and non-Hispanic unknown were excluded.

The age-standardized mortality rate for MPNST climbed from 0.02 per 100 000 persons in 2000 to 0.09 per 100 000 persons in 2002 (APC, 90.8%), fluctuated marginally through 2008 (APC, 4.9%), and then stabilized to 2019. The disparities by age, race/ethnicity, grade, and stage were also depicted in Figure 1 and Supplementary Table 10–11 (Supplemental Digital Content 1, http://links.lww.com/JS9/C899). Meanwhile, the 20-year limited-duration prevalence (Supplementary Table 12, Supplemental Digital Content 1, http://links.lww.com/JS9/C899) rose from 0.00129% in 2000 to 0.00159% in 2008 but then declined to 0.00131% in 2019 (APC, −1.6%).

For survival, the median OS for patients with MPNST was 44 months. The estimated survival rate was demonstrated in Supplementary Table 13 (Supplemental Digital Content 1, http://links.lww.com/JS9/C899). Then, we delved into the efficacy of various treatment approaches on the survival outcomes of patients with MPNST (Fig. 2). Predominantly, surgical intervention emerged as the most effective treatment strategy for MPNST (HR, 0.59). In contrast, monotherapies involving either chemotherapy or radiotherapy did not exhibit substantial benefits (HRs, 1.68 and 3.06, respectively). Regarding combined therapies, after adjustment with inverse probability of treatment weighting and propensity score matching, no survival advantage in patients at stage I–III when surgery was coupled with radiotherapy (HRs, 1.09 and 1.04, respectively). An amalgamation of surgery and chemotherapy demonstrated a deleterious impact. In the context of advanced MPNST, radiotherapy was associated with improved survival, whereas chemotherapy did not yield a significant survival benefit. Moreover, subgroup analysis was performed to further assess the effect of different therapeutic modalities on survival among different subgroups (Supplementary Figure 3, Supplemental Digital Content 1, http://links.lww.com/JS9/C899 and Supplementary Table 14–33, Supplemental Digital Content 1, http://links.lww.com/JS9/C899).

**Figure 2:**
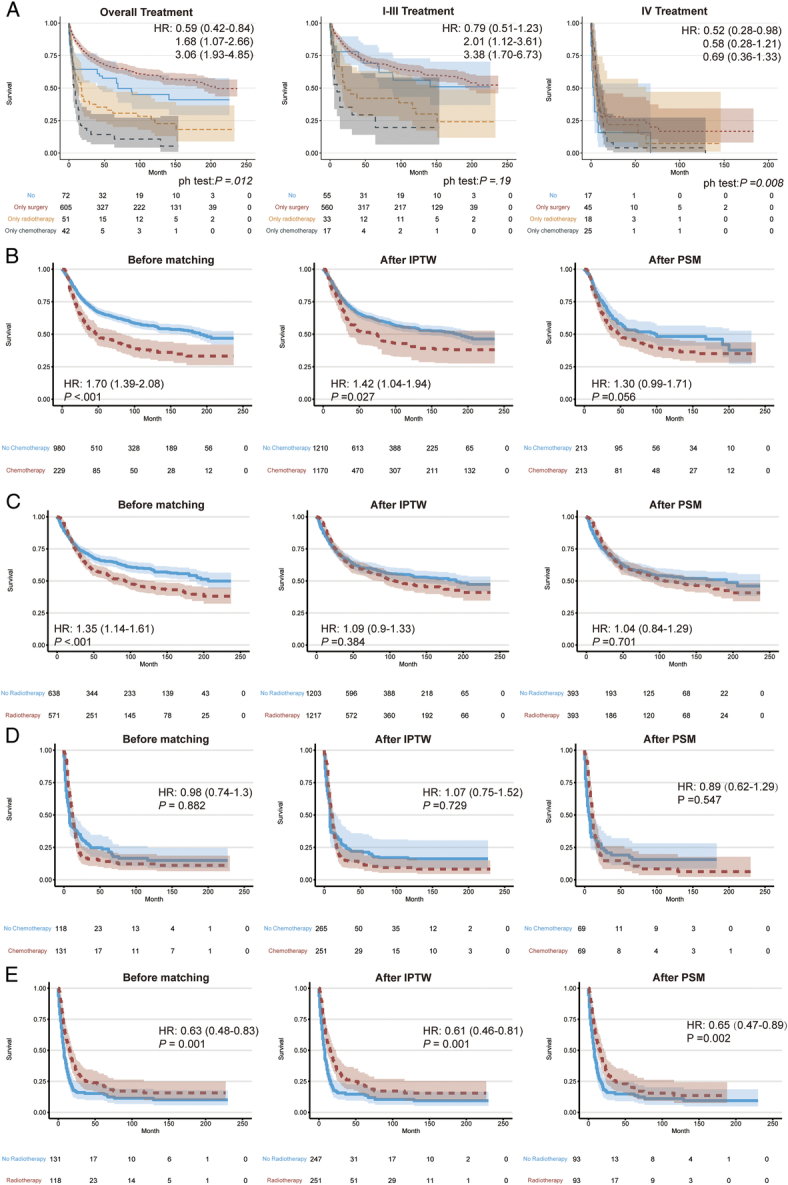
Survival curve for patients diagnosed with malignant peripheral nerve sheath tumor among different subgroups. (A) Treatment for overall patients at Stages I–III and IV. (B) Chemotherapy for patients at Stages I–III. (C) Radiotherapy for patients at Stages I–III. (D) Chemotherapy for patients at Stage IV. (E) Radiotherapy for patients at Stage IV. HR, hazard ratio.

For overall survival, compared with primary tumor, the mortality risk of secondary MPNST (Supplementary Figure 4, Supplemental Digital Content 1, http://links.lww.com/JS9/C899) was greatly increased, particularly for radiation-associated MPNST. Additionally, age, tumor size, pathological type tumor stage, and grade were also found to be significantly associated with survival (*P*<.05, Supplementary Table 34–35, Supplemental Digital Content 1, http://links.lww.com/JS9/C899), and a predictive nomogram was established (Supplementary Figure 5–6, Supplemental Digital Content 1, http://links.lww.com/JS9/C899).

Notably, 193 SPCs were observed among 1622 (Supplementary Table 36–37, Supplemental Digital Content 1, http://links.lww.com/JS9/C899), with a 1.6-fold higher risk (SIR, 2.58) compared to the general population (Supplementary Table 38–48, Supplemental Digital Content 3, http://links.lww.com/JS9/C901, Supplemental Digital Content 1, http://links.lww.com/JS9/C899 and Figure 7, Supplemental Digital Content 1, http://links.lww.com/JS9/C899). The SIRs were significantly higher for cancers of soft tissue, small intestine, brain, and other nervous system, melanoma and thyroid (SIRs, 26.53, 13.21, 9.17, 5.38, and 5.12, respectively). Younger survivors exhibited a higher SIR (SIRs,17.39, 8.72, 2.11, and 2.02 for those aged 0–19, 20–39, 40–59, ≥60 years, respectively). Additionally, higher SIRs were observed among those who received chemotherapy, especially cancers of the small intestine, colon and rectum, and brain (SIRs, 65.82, 3.77, and 46.48, respectively).

Among 1063 deaths of these patients, the leading causes of death were malignant cancers (74.32%), cardiovascular disease (6.59%), benign neoplasm (5.64%), and congenital anomalies (4.14%). Except for soft tissue cancer, the risk of 13 out of 37 types was higher than expected (Supplementary Table 49–56, Supplemental Digital Content 1, http://links.lww.com/JS9/C899, Supplemental Digital Content 3, http://links.lww.com/JS9/C901). Besides, there was an increased risk for CVDs (SMR, 16.1), especially cerebrovascular disease (SMR, 7.89).

This study characterized the evolving epidemiological and survival pattern of MPNST between 2000 and 2019. Despite the dramatic reductions in overall incidence, the incidence remained constant among those vulnerable groups who may be associated with NF1 mutation. Factors including age, tumor size, histological grade, clinical stage, and therapeutic interventions were found to exert a significant influence on overall survival. Among treatment modalities, surgical intervention was identified as the most efficacious. A nomogram was developed to facilitate the estimation of mortality risk and clinical decision-making. In addition to a poor prognosis, subsequent tumor susceptibility and elevated risk of co-morbidities further heightened the complexity of the management for MPSNT. Finally, a Chinese cohort was included to furnish a more expansive view on the epidemiologic and clinical features of MPNST across diverse demographic settings (Supplementary Table 57, Supplemental Digital Content 1, http://links.lww.com/JS9/C899).

Findings underscore the condition’s management complexity, advocating for a comprehensive strategy encompassing healthcare policy, clinical practice, patient education, and research. The research called for increased research and healthcare resources, improved screening, enhanced treatment protocols, and holistic care for MPNST survivors. The necessity for aggressive treatment in high-risk groups and the importance of regular monitoring for early SPC detection in survivors are emphasized. These insights serve as valuable educational resources for patients, stressing the importance of regular follow-ups and lifestyle adjustments.

## Ethical approval

Because the data from the SEER database were de-identified and publicly available, informed consent was not required and ethical review was exempted. Ethical approval for the Chinese cohort was obtained from the Ethics Committee of the center, and informed consent was acquired from the patients or their legal guardians.

## Consent

Because the data from the SEER database were de-identified and publicly available, informed consent was not required and ethical review was exempted. Ethical approval for the Chinese cohort was obtained from the Ethics Committee of the center, and informed consent was acquired from the patients or their legal guardians.

## Source of funding

This work was supported by grants from National Natural Science Foundation of China (82202470; 82102344; 82172228); Shanghai Rising Star Program supported by Science and Technology Commission of Shanghai Municipality (20QA1405600); Natural Science Foundation of Shanghai (22ZR1422300); Innovative research team of high-level local universities in Shanghai (SHSMU-ZDCX20210400); Clinical Research Plan of SHDC (SHDC2020CR1019B); Shanghai Clinical Research Center of Plastic and Reconstructive Surgery supported by Science and Technology Commission of Shanghai Municipality (Grant No. 22MC1940300).

## Author contribution

Z.C.W. and Q.F.L.: conception, design, and financial support; J.J.R.: conception and design; B.Y.Z. and J.L.: collection and/or assembly of data; L.L.G.: data analysis and interpretation; H.T.S. and J.X.H.: data analysis, interpretation, and manuscript writing.

## Conflicts of interest disclosure

The authors declare that the research was conducted in the absence of any commercial or financial relationships that could be construed as a potential conflict of interest.

## Research registration unique identifying number (UIN)

1. Name of the registry: Clinical trials registry.
2. Unique identifying number or registration ID: NCT02875327.
3. Hyperlink to your specific registration (must be publicly accessible and will be checked): https://classic.clinicaltrials.gov/ct2/show/NCT02875327?cond=SEER&draw=3&rank=11

## Guarantor

Wang, Li, and Han had full access to all of the data in the study and take responsibility for the integrity of the data and the accuracy of the data analysis.

## Data availability statement

All data from the SEER database used in this work were publicly available, shown at: https://seer.cancer.gov/. The data regarding the Chinese cohort are available from the corresponding author, upon reasonable request.

## Provenance and peer review

Not commissioned, externally peer-reviewed.

## Supplementary Material

SUPPLEMENTARY MATERIAL
