# Supplementary material for: Epidemiologic and survival analysis of malignant peripheral nerve sheath tumors: a retrospective cohort study
Source: Int J Surg. 2024 Jun 27;110(12):8210–4. doi: 10.1097/JS9.0000000000001756 (PMC11634120; doi:10.1097/JS9.0000000000001756)
Supplement: SUPPLEMENTARY MATERIAL [file js9-110-8210-s001.docx]

**Supplementary Materials**

**Epidemiologic and Survival Analysis of Malignant Peripheral Nerve Sheath Tumors: A Retrospective Cohort Study**

**Content**

**eMethodss**

1. Overview of SEER database

2. Study design

2.1 Objective

2.2 Study population

2.3 Outcomes

2.4 Subgroups

3. Statistical analysis

3.1 Incidence rate and the corresponding trends

3.2 Survival analysis

3.3 Risk of developing subsequent primary cancer and dying from any cause

3.4 Statistical test

3.5 Sensitivity analysis

4. Supplementary references

**Supplementary Tables**

**Supplementary table 1.** Definition and grouping strategy of subsequent primary cancer

**Supplementary table 2.** Characteristics of patients diagnosed with malignant peripheral nerve sheath tumor in the SEER database.

**Supplementary table 3.** Age-specific incidence rate and incidence rate ratio in malignant peripheral nerve sheath tumor during 2000-2019 in 17 and 22 SEER registries.

**Supplementary table 4.** Age-specific incidence rate and incidence rate ratio in malignant peripheral nerve sheath tumor by sex during 2000-2019 in 17 SEER registries.

**Supplementary table 5.** Age-standardized incidence and rate ratio in malignant peripheral nerve sheath tumor by demographic during 2000-2019 in 17 SEER registries.

**Supplementary table 6.** Age-standardized incidence and rate ratio in malignant peripheral nerve sheath tumor by registry during 2000-2019 in 22 SEER registries.

**Supplementary table 7.** Trends in incidence rate of malignant peripheral nerve sheath tumor among different subgroups in 17 SEER registries during 2000-2019.

**Supplementary table 8.** Trends in incidence rate of malignant peripheral nerve sheath tumor among different subgroups in 22 SEER registries during 2000-2019.

**Supplementary table 9.** Trends in incidence rate of malignant peripheral nerve sheath tumor among different subgroups in 8 SEER registries during 1975-2019.

**Supplementary table 10.** Trends in incidence-based mortality rate of malignant peripheral nerve sheath tumor among different subgroups in 17 SEER registries during 2009-2019.

**Supplementary table 11.** Trends in incidence-based mortality rate of malignant peripheral nerve sheath tumor among different subgroups in 12 SEER registries during 2009-2019.

**Supplementary table 12.** Age-standardized limited duration prevalence of malignant peripheral nerve sheath tumor during 2000-2019.

**Supplementary table 13**. Survival rate of primary malignant peripheral nerve sheath tumor among different subgroups during 2000-2019 in 17 SEER registries.

**Supplementary table 14**. Baseline of patients at stage I-III stratified by radiotherapy before and after matching.

**Supplementary table 15**. Analysis for radiotherapy utilization among patients at stage I-III.

**Supplementary table 16**. Cox regression of overall survival for patients at stage I-III stratified by radiotherapy.

**Supplementary table 17** Restricted mean survival time for patients at stage I-III stratified by radiotherapy.

**Supplementary table 18**. Subgroup analysis for patients at stage I-III with radiotherapy.

**Supplementary table 19**. Baseline of patients at stage I-III stratified by chemotherapy before and after matching.

**Supplementary table 20**. Analysis for chemotherapy utilization among patients at stage I-III.

**Supplementary table 21**. Cox regression of overall survival for patients at stage I-III stratified by chemotherapy.

**Supplementary table 22**. Restricted mean survival time for patients at stage I-III stratified by chemotherapy.

**Supplementary table 23**. Subgroup analysis for patients at stage I-III with chemotherapy.

**Supplementary table 24**. Baseline of patients at stage IV stratified by radiotherapy before and after matching.

**Supplementary table 25**. Analysis for radiotherapy utilization among patients at stage IV.

**Supplementary table 26**. Cox regression of overall survival for patients at stage I-III stratified by radiotherapy.

**Supplementary table 27**. Restricted mean survival time for patients at stage IV stratified by radiotherapy.

**Supplementary table 28**. Subgroup analysis for patients at stage IV with radiotherapy.

**Supplementary table 29**. Baseline of patients at stage IV stratified by chemotherapy before and after matching.

**Supplementary table 30**. Analysis for chemotherapy utilization among patients at stage IV.

**Supplementary table 31**. Cox regression of overall survival for patients at stage I-III stratified by chemotherapy.

**Supplementary table 32**. Restricted mean survival time for patients at stage IV stratified by chemotherapy.

**Supplementary table 33**. Subgroup analysis for patients at stage IV with chemotherapy.

**Supplementary table 34.** Baseline of training and validation cohorts in the nomogram for survival of primary malignant peripheral nerve sheath tumor.

**Supplementary table 35.** Univariate and multivariate analysis for malignant peripheral nerve sheath tumor.

**Supplementary table 36.** Characteristics of survivors diagnosed with primary malignant peripheral nerve sheath tumor in the SEER 17 registries.

**Supplementary table 37.** The risk of developing SPC among survivors diagnosed with primary malignant peripheral nerve sheath tumor during 2000-2019 in 17 SEER registers.

**Supplementary table 38.** The risk of developing SPC among survivors diagnosed with primary malignant peripheral nerve sheath tumor by sex during 2000-2019 in 17 SEER registers. (See in excel)

**Supplementary table 39.** The risk of developing SPC among survivors diagnosed with primary malignant peripheral nerve sheath tumor by age during 2000-2019 in 17 SEER registers.

**Supplementary table 40.** The risk of developing SPC among survivors diagnosed with primary malignant peripheral nerve sheath tumor by latency during 2000-2019 in 17 SEER registers.

**Supplementary table 41.** The risk of developing SPC among survivors diagnosed with primary malignant peripheral nerve sheath tumor by race during 2000-2019 in 17 SEER registers.

**Supplementary table 42.** The risk of developing SPC among survivors diagnosed with primary malignant peripheral nerve sheath tumor by radiotherapy during 2000-2019 in 17 SEER registers.

**Supplementary table 43.** The risk of developing SPC among survivors diagnosed with primary malignant peripheral nerve sheath tumor by chemotherapy during 2000-2019 in 17 SEER registers.

**Supplementary table 44.** The risk of developing SPC among survivors diagnosed with primary malignant peripheral nerve sheath tumor with single outcome during 2000-2019 in 17 SEER registers.

**Supplementary table 45.** The risk of developing SPC among 5-year survivors diagnosed with primary malignant peripheral nerve sheath tumor during 2000-2019 in 17 SEER registers.

**Supplementary table 46.** The risk of developing SPC among survivors diagnosed with primary malignant peripheral nerve sheath tumor (including distant stage at diagnosis) during 2000-2019 in 17 SEER registers.

**Supplementary table 47.** The risk of developing SPC among survivors diagnosed with primary malignant peripheral nerve sheath tumor (aged less than 89 years) during 2000-2019 in 17 SEER registers.

**Supplementary table 48.** The risk of developing SPC among survivors diagnosed with primary malignant peripheral nerve sheath tumor during 1975-2019 in 8 SEER registers.

**Supplementary table 49.** Causes of death among survivors diagnosed with primary malignant peripheral nerve sheath tumor during 2000-2019 in 17 SEER registers.

**Supplementary table 50.** Causes of death among survivors diagnosed with primary malignant peripheral nerve sheath tumor by sex during 2000-2019 in 17 SEER registers.

**Supplementary table 51.** Causes of death among survivors diagnosed with primary malignant peripheral nerve sheath tumor by age during 2000-2019 in 17 SEER registers.

**Supplementary table 52.** Causes of death among survivors diagnosed with primary malignant peripheral nerve sheath tumor by latency during 2000-2019 in 17 SEER registers.

**Supplementary table 53.** Causes of death among survivors diagnosed with primary malignant peripheral nerve sheath tumor by race during 2000-2019 in 17 SEER registers.

**Supplementary table 54.** Causes of death among survivors diagnosed with primary malignant peripheral nerve sheath tumor by radiotherapy during 2000-2019 in 17 SEER registers.

**Supplementary table 55.** Causes of death among survivors diagnosed with primary malignant peripheral nerve sheath tumor by chemotherapy during 2000-2019 in 17 SEER registers.

**Supplementary table 56.** Causes of death among survivors diagnosed with primary malignant peripheral nerve sheath tumor during 1975-2019 in 8 SEER registers.

**Supplementary table 57.** Comparison of patients with malignant peripheral nerve sheath tumor from the Chinese multicenter and the SEER database.

**Supplementary Figures**

**Supplementary figure 1.** Flowchart of study.

**Supplementary figure 2.** Incidence and prevalence of malignant peripheral nerve sheath tumor.

**Supplementary figure 3**. Forest plot for patients diagnosed with malignant peripheral nerve sheath tumor among different subgroups.

**Supplementary figure 4.** Survival curve for patients diagnosed with malignant peripheral nerve sheath tumor among different subgroups.

**Supplementary figure 5.** Nomogram to predict the survival probabilities of patients with malignant peripheral nerve sheath tumor and the ROC curve of the nomogram using the training and validation sets.

**Supplementary figure 6.** Calibration plot and decision curve analysis for the nomogram.

**Supplementary figure 7.** Risk of developing subsequent primary cancers and cause of death among patients with malignant peripheral nerve sheath tumor.

**eMethodss**

**1. Overview of the SEER database**

In this retrospective, population-based study, the Surveillance, Epidemiology, and End Result (SEER) database was used. (See <https://seer.cancer.gov/>) The SEER program is an authoritative source collecting data on cancer epidemiology (incidence and prevalence) and clinical characteristics (tumor site, stage at diagnosis, and follow-up for vital status) in the United States.

Here are the population and registries included in the SEER database:

SEER 8 included San Francisco-Oakland SMSA, Connecticut, Hawaii, Iowa, New Mexico, Seattle [Puget Sound], Utah, Atlanta [Metropolitan], covering a small US population (approximately 8.3%) but with a long follow-up from 1975 through 2019.

SEER 12 included San Francisco-Oakland SMSA, Connecticut, Hawaii, Iowa, New Mexico, Seattle [Puget Sound], Utah, Atlanta [Metropolitan], San Jose-Monterey, Los Angeles, Alaska Natives and Rural Georgia, covering approximately 12.2% of US population from 1992 through 2019.

SEER 17 included San Francisco-Oakland SMSA, Connecticut, Hawaii, Iowa, New Mexico, Seattle (Puget Sound), Utah, Atlanta [Metropolitan], San Jose-Monterey, Los Angeles, Rural Georgia, California excluding SF/SKM/LA, Kentucky, Louisiana, New Jersey, and Greater Georgia, covering approximately 26.5% of US population from 2000 through 2019.

SEER 22 included San Francisco-Oakland SMSA, Connecticut, Hawaii, Iowa, New Mexico, Seattle (Puget Sound), Utah, Atlanta [Metropolitan], San Jose-Monterey, Los Angeles, Rural Georgia, California excluding SF/SKM/LA, Kentucky, Louisiana, New Jersey, Greater Georgia, Idaho, Illinois, Massachusetts, New York and Texas, covering approximately 47.9% of US population from 2000 through 2019.

The following SEER databases with varying registry/year grouping were chosen in this study.

**Analyses for incidence and incidence-based mortality**

To estimate the incidence and mortality rate of malignant peripheral nerve sheath tumor (MPNST) and determine the temporal trend, *Incidence - SEER Research Limited-Field Data with Delay-Adjustment, 17 Registries, Malignant Only, Nov 2021 Sub (2000-2019)* database and *Incidence-Based Mortality - SEER Research Limited-Field Data with Delay-Adjustment, 17 Registries, Malignant Only, Nov 2021 Sub (2000-2019)* database in the “Rate Sessions” were used, respectively.

In addition, for incidence, *22 SEER registries (2000-2019)* and *8 registries (1975-2019)* database were used as sensitivity analysis whereas *12 SEER registries (2000-2019)* for incidence-based mortality.

**Analyses for limited duration prevalence**

To estimate the limited 20-year duration prevalence of MPNST, *Incidence - SEER Research Data, 8 Registries, Nov 2021 Sub (1975-2019)* database in the “Limited-Duration Prevalence Session” was used. Besides, *Incidence - SEER Research Data, 17 Registries, Nov 2021 Sub (2000-2019)* database was used to calculate the limited 10-year duration prevalence during 2010-2019.

**Analyses for the risk of subsequent primary cancer and cause of death**

To estimate the risk of subsequent primary cancers (SPCs) and cause of death (COD) among MPNST survivors, *Incidence - SEER Research Data, 17 Registries, Nov 2021 Sub (2000-2019)* database and *Incidence - SEER Research Data, 17 Registries, Nov 2021 Sub (2000-2019) for SMRs* database in the “MP-SIR/SMR” were used, respectively. Sensitivity analyses were performed using *8 SEER registries (1975-2019)*.

**2. Study design**

**2.1 Objective**

The aim of this study is to estimate and describe:

1) The incidence, mortality and prevalence of MPNST and current trend.

2) The survival outcome and the cause of death for MPNST survivors.

3) The risk of developing SPC among MPNST survivors.

**2.2 Study population**

Malignant peripheral nerve sheath tumor is defined as a malignant with evidence of Schwann cell or perineurial cell differentiation commonly arising in a peripheral nerve or in extraneural soft tissue according to the WHO classification. Given there are multiple confusing terminologies, including malignant schwannoma and malignant neurofibroma, here, we identified MPNST using the histologic codes from the *International Classification of Diseases for Oncology, 3^rd^ Edition,* (9540/3, 9542/3, 9550/3, 9560/3, 9561/3 and 9571/3). Meanwhile, we used 9540/3 alone as sensitivity analysis. Cases diagnosed based only on autopsy reports or death certificated were excluded. The corresponding histologic type to the ICD-O-3 code were shown as follow:

9540/3 - Malignant peripheral nerve sheath tumor

9542/3 - Epithelioid MPNST

9550/3 - Plexiform neurofibrosarcoma, malignant

9560/3 - Neurilemona, malignant

9561/3 - MPNST with rhabdomyoblastic differentiation

9571/3 – Perineurioma, malignant

**2.3 Outcomes**

In this study, there were several outcomes:

1) The incidence and mortality rate per 100,000 persons of MPNST and the annual percentage change (APC) or average APC (AAPC) to describe the corresponding trends.

2) The limited 20-year duration prevalence of MPNST.

3) The prognostic factors for MPNST and the survival rate among different subgroups.

4) The effects of varying treatments on the prognosis at different stages of TNM classification

5) A nomogram and the assessment of predictive performance

6) The risk of developing SPCs and death by cause among MPSNT survivors.

**2.4 Subgroups**

In this study, the following sociodemographic and clinical characteristics were included as covariates:

1) Age at diagnosis: Grouped into 00-19 years, 20-39 years, 40-59 years and ≥60 years. (Here, we adopted age of 40 years as the cut-off for the exploration of neurofibromatosis type 1, and assume those age <40 years as patients with NF1.

2) Sex: Male and female.

3) Race/ethnicity: Non-Hispanic White (NHW), non-Hispanic Black (NHB), non-Hispanic Asian/Pacific Islander (NHAPI) and Hispanic.

4) SEER registry.

5) Geographic location: Urban and rural.

6) Income: <$50000, $50000-$74999 and >$75000.

7) Tumor site: Head and neck, and others.

The identification of tumor sited in head and neck was according to the ICD-O-3 code of C00.0-C14.8, C30.0-C32.9, C41.0-C41.1, C44.0-C44.4, C47.0 and C49.0

8) Tumor size: 00-49 mm and ≥50 mm.

9) Tumor grade:

I – Well differentiated;

II- Moderately differentiated;

III and IV – Poorly differentiated, undifferentiated and anaplastic.

10) Tumor stage at diagnosis: Localized, regional and distant.

11) Histologic type: Triton tumor and others.

12) Treatment:

Receipt of chemotherapy (yes or no).

Receipt of radiotherapy (yes or no).

13) Latency: 2-11, 12-59, 60-119 and ≥120 months

Specifically, for survival analysis among different TNM stage, we included the following sociodemographic and clinical characteristics as covariates:

1. TNM stage: I, II, III and IV (I-III vs IV).
2. Diagnosis year: 2000-2004, 2005-2009, 2010-2014 and 2015-2019.
3. Age: 00-19 years, 20-64 years and ≥ 65 years
4. Sex: Male and Female.
5. Race: NHW, NHB, NHAPI, Hispanic and others.
6. Married: Yes and No.
7. Income: ≤ $49999, $50000-74999 and ≥ 75000.
8. Location: Urban and rural.
9. Site: Head and neck, intracranial, limb and others.
10. Grade: G0/GX, G1 and G2.
11. Size: 0-50mm, 51-100mm, 101-150mm and > 150mm.
12. Surgery: No and yes; local excision and radical excision.
13. Radiotherapy: No and yes.

**2.5 Follow-up, ascertainment of SPC and cause of death**

**Follow-up**

To estimate the risk of death by cause, patients with MPNST were followed after diagnosis, until death or loss to follow-up, or Dec.31, 2019 (study date–of–end), while survivors were followed from 2 months after primary MPNST diagnosed for the risk of SPC.

**Ascertainment of Subsequent primary cancer**

SPCs were ascertained as all new primary cancers, except non–melanoma skin cancer because most such tumors (i.e., basal cell and squamous cell skin cancers) are not reportable to the SEER Program. In addition, the soft tissue tumor excluded MPNST was also performed as sensitivity analysis to minimize bias cause by misclassification of recurrence. SPC was also identified per *International Classification of Diseases for Oncology (third edition)* and SEER–multiple primary/histology rules ^1^. Multiple events in one survivor were allowed (i.e., survivors remained in study after prior SPCs onset before censoring) because some SPC occurs a long time after primary cancer diagnosed ^2^. The definition of SPCs and further grouping strategy (according to SEER site groups) were shown in **Supplementary table1**. Chronic lymphocytic leukemia was classified into non–Hodgkin lymphoma per 2001 WHO classification of lymphoid neoplasms ^3^.

**Ascertainment of Cause of death**

Cause of death was determined by *International Classification of Diseases*–10. Death due to non–melanoma skin cancers were excluded in the analysis.

**Ascertainment of Radiation-related MPNST**

There is no record of radiation-related MPNST at the SEER database. Here, we determined radiation-related MPNST by previous history of initial radiotherapy.

**3. Statistical analysis**

**3.1 Incidence and mortality rate and the corresponding trends**

The age-adjusted (standardized to 2000 US population) incidence and mortality of MPNST per 100,000 persons of was estimated and adjusted for reporting delay. The annual incidence and mortality rate was obtained for overall population, and stratified by age, sex, race/ethnicity, registry, location, income, tumor site, tumor size, tumor grade and stage. The 95% confidence interval for incidence was determined using Tiwari et al., 2006 modification method ^4^.

The incidence rate ratios (IRRs), defined as the ratio of two incidence rates were estimated to quantify the risk of sociodemographic characteristics utilizing all cases diagnosed during 2000-2019. To calculate the IRR, the incidence rate among the exposed portion of the population, divided by the incidence rate in the unexposed portion of the population, gives a relative measure (IRR) of the effect of a given exposure. We also examined the heterogeneity of association magnitude between oldest and youngest age groups (≥ 40 and < 40 years, defined as sporadic and NF1-associated MPNST, respectively) by random-effects meta-regression.

The corresponding trends in incidence were quantified as annual percentage change (APC) estimated by fitting joinpoint regression models via the National Cancer Institute’s (NCI) Joinpoint software. The best-fitting log-linear regression model was adopted to identify calendar years where APCs changed significantly. The average APC (AAPC), computed as a weighted average of the APCs, was used for further comparison to determine the subgroups which demonstrated more pronounced increase. Here, we estimated the AAPC for the most recent interval based on the observed joinpoint of the overall trend (i.e., during 2000-2019 for incidence and during 2008-2019 for the mortality) . Further stratified analyses for trends were performed by sociodemographic and clinical factors except for SEER registry and treatment due to the limited sample size. The statistical significance of APC/AAPC was examined by t-test.

In addition, 20-year limited-duration prevalence, defined as the proportion of people alive on a certain day who had a diagnosis of MPNST within the past 20 years was calculated in the SEER 8 registries during 2000 to 2019. The corresponding APC was also estimated to examine the current trend. Sensitivity analyses was performed using the limited 10-year prevalence was also estimated in the SEER 17 registries during 2010 to 2019. Besides, association between the sociodemographic characteristics and the prevalence was determined by the Chi-square test.

**3.2 Survival analysis**

1-year, 3-year and 5-year survival rates were calculated by Kaplan-Meier method. Also, relative survival rates were also estimated to further quantify the excess mortality by MPNST, which is defined as the ratio of the observed survival (all cause survival) of a cohort of cancer patients to the expected survival of a comparable set of cancer free individuals.

Univariate Cox proportional hazards regression was carried out to determine the association of sociodemographic and clinical variables with OS by estimating hazard ratios (HRs) and 95%CI whereas multivariate Cox regression was used to further evaluate significant factors in univariate regression with adjustment.

A nomogram based on multivariate Cox regression with sex adjusted was constructed for clinicians. Patient was divided into training and validation cohort by 3:1. In both cohorts, the consistency index (C index) and the area under receiver operating characteristic curve (AUC) were determined for the discriminative capability of the nomogram while the calibration curve and decision curve analysis (DCA) were conducted.

We also included more comprehensive analysis of the survival status and treatment options for MPNST patients across different TNM stages. Propensity Score Matching (PSM) and Inverse Probability of Treatment Weighting (IPTW) are methods used to balance these baseline characteristics and reduce bias. Cox regression and PH test were performed to the association of clinical variables with OS. Besides, restricted mean survival time (RMST) was also estimated to represent the expected survival time within a specified time window, providing a direct summary measure of the survival curve.

For all analyses, time of follow-up was from the date of diagnosis until death, date of last contact, or end of study period.

**3.3 Risk of developing** **subsequent primary cancer and dying from any cause**

To estimate the risk of developing SPCs among MPNST survivors, standardized incidence ratio (SIR) was obtained. The corresponding absolute excess incidence (AEI) per 10,000 person–year and incidence per 100,000 person-year were calculated. Estimates were limited to observed events with five or more due to confidentiality consideration. SIR and AEI had different perspectives. SIR indicated the strength of association between MPNST and SPCs, while AEI was more widely used to measure the impact of SPC burden in a certain population, to provide information for screening or prevention programs ^5^.

$\text{SIR=}\text{O}/\text{E}$,

$\text{AEI}\text{ per 100,000 person-year=}{\text{(}\text{O}\text{-}\text{E}\text{)}}/{\text{PY}\text{×100,000}}$,

$\text{Incidence per 100,000 person-year=}\text{O}/\text{PY}\text{×100,000}$,

where *O* was the observed number of SPCs,

*E* was the expected number of the SPCs,

*PY* was the person-year at risk since 5-year after primary BC diagnosed.

To quantify the risk of dying from any cause among MPNST survivors, standardized mortality ratio (SMR), absolute excess mortality (AEM) per 10,000 person-year and mortality per 10,000 person-year were calculated similarly.

Relative risks (RRs) for SPCs stratified by sociodemographic and clinical factors were also estimated using multivariate Poisson regression or negative binary regression (when dispersion was detected). Test for heterogeneity were conducted using likelihood ratio tests ^6,7^. The regression model was adjusted for the following variables:

Age (00-19, 20-39, 40-59 and ≥60 years),

Sex (Male and female),

Latency: event time since MPNST diagnosis (2-11, 12-59, 60-119, ≥ 120 months),

Log (Expected number): the log of the expected number of cases/death as an offset (as an indirectly adjustment for attained age and calendar year).

Two Poisson regression models (negative binary regression was applied when dispersion detected) were established with or without the variable of interest. Likelihood ratio test was applied to determine the statistical significance in difference of two regression models.

Notably, we performed the analysis of the risk of SPC only among those with known stage and without distant considering their poor survival. A sensitivity analysis among all survivors was also conducted.

**3.4 Statistical test**

All analyses were performed using SEER*Stat Version 8.4.0 (National Cancer Institute), Joinpoint Regression Program (National Cancer Institute) version 4.9.1.0 and **R** statistics software. All P-values were 2-sided, and statistical significance was claimed when less than 0.05. Adjustment for multiple comparison was not applied because all analyses are exploratory.

**3.5 Sensitivity analysis**

Serval sensitivity analyses were performed to examine the robustness of our results.

**Incidence and mortality**

1) The incidence and corresponding trends of MPNST during 1975-2019, using 8 SEER registries.

2) The incidence and corresponding trends of MPNST during 2000-2019, using 22 SEER registries.

**Mortality**

The mortality and corresponding trends of MPNST during 2000-2019, using 12 SEER registries.

**Risk of subsequent primary cancer**

1) The risk of developing SPCs among MPNST survivors with single outcome diagnosed during 2000-2019, using SEER 17 database.

2) The risk of developing SPCs among MPNST survivors less than 89 years diagnosed during 2000-2019, using SEER 17 database.

3) The risk of developing SPCs among MPNST survivors by starting follow-up since 5 years after diagnosis during 2000-2019, using SEER 17 database.

4) The risk of developing SPCs among MPNST survivors diagnosed during 1975-2019, using SEER 8 database.

**Cause of death**

Causes of death among MPNST survivors diagnosed during 1975-2019, using SEER 8 database.

**Supplementary references**

1. Institute NC. 2007 Multiple Primary and Histology Coding Rules. <https://seer.cancer.gov/tools/mphrules/> (accessed 1 April 2023).

2. Institute NC. SEER Cancer Statistics Review (CSR) 1975-2017. 2020. <https://seer.cancer.gov/archive/csr/1975_2017/> (accessed 1 April 2023)

3. Turner JJ, Morton LM, Linet MS, et al. InterLymph hierarchical classification of lymphoid neoplasms for epidemiologic research based on the WHO classification (2008): update and future directions. *Blood* 2010; **116**(20): e90-8.

4. Tiwari RC, Clegg LX, Zou Z. Efficient interval estimation for age-adjusted cancer rates. *Stat Methods Med Res* 2006; **15**(6): 547-69.

5. Rochelle E. Curtis DMF, Elaine Ron, et al. New Malignancies Among Cancer Survivors: SEER Cancer Registries, 1973-2000. <https://seer.cancer.gov/archive/publications/mpmono/MPMonograph_complete.pdf>

6. Yasui Y, Liu Y, Neglia JP, et al. A methodological issue in the analysis of second-primary cancer incidence in long-term survivors of childhood cancers. *Am J Epidemiol* 2003; **158**(11): 1108-13.

7. Rostgaard K. Methods for stratification of person-time and events - a prerequisite for Poisson regression and SIR estimation. *Epidemiol Perspect Innov* 2008; **5**: 7.

**Supplementary table1. Definition and grouping strategy of subsequent primary cancer**

|  | ICD-O-3 site | ICD-O-3 histology |
| --- | --- | --- |
| Head and Neck |  | Any histologies excluding 9050- 9055, 9140, 9590-9992 |
| Oral Cavity and Pharynx | C000-C009, C019-C029, C079-C089, C040-C049, C030-C039, C050-C059, C060-C069, C110-C119, C090-C099, C100-C109, C129, C130-C139, C140, C142, C148 |  |
| Nose, Nasal Cavity and Middle Ear | C300-C301, C310-C319 |  |
| Larynx | C320-C329 |  |
| Esophagus | C150-C159 |  |
| Stomach | C160-C169 |  |
| Small Intestine | C170-C179 |  |
| Colon and Rectum | C180, C181, C182, C183, C184, C185, C186, C187, C188-C189, C260, C199, C209 |  |
| Anus, Anal Canal and Anorectum | C210-C212, C218 |  |
| Liver and intrahepatic bile duct | C220, C221, C239 |  |
| Gallbladder and other biliary | C239, C240-C249 |  |
| Pancreas | C250-C259 |  |
| Retroperitoneum, peritoneum and other digestive system | C480, C481-C482, C268-269, C488 |  |
| Lung and Bronchus | C340-C349 |  |
| Pleura, trachea and other respiratory | C384, C339, C381-C383, C388, C390, C398, C399 |  |
| Bones and Joints | C400-C419 |  |
| Soft Tissue including Heart | C380, C470-C479, C490-C499 |  |
| Melanoma of the Skin | C440-C449 | 8720-8790 |
| Breast | C500-c509 | Any histologies excluding 9050- 9055, 9140, 9590-9992 |
| Cervix Uteri | C530-C539 |  |
| Corpus and Uterus, NOS | C540-C549, C559 |  |
| Ovary | C569 |  |
| Vagina, vulva and other female genital organs | C529, C510-C519, C570-C579, C589 |  |
| Prostate | C619 |  |
| Testis | C620-C629 |  |
| Penis and other male genital organs | C600-C609, C630-C639 |  |
| Urinary Bladder | C670-C679 |  |
| Kidney and Renal Pelvis | C649, C659 |  |
| Ureter and other urinary organs | C669, C680-C689 |  |
| Eye and Orbit | C690-C699 |  |
| Brain and Other Nervous System | C710-C719, C700-C709, C720-C729 |  |
| Thyroid | C739 |  |
| Other endocrine system | C379, C740-C749, C750-C759 |  |
| Hodgkin Lymphoma |  |  |
| Nodal | C024, C098-C099, C111, C142, C379, C422, C770- C779 | 9650-9667 |
| Extranodal | Any sites |  |
| Non-Hodgkin Lymphoma |  |  |
| Nodal | C024, C098, C099, C111, C142, C379, C422, C770- C779 | 9590-9597, 9670-9671, 9673, 9675, 9678-9680, 9684, 9687- 9691, 9695, 9698-9702, 9705, 9708-9709, 9712, 9714-9719, 9724-9729, 9735, 9737-9738, 9811-9818, 9823, 9827, 9837 |
| Extranodal | All sites except C024, C098-C099, C111, C142, C379, C422, C770-C779 | 9590-9597, 9670671, 9673, 9675, 9678-9680, 9684, 9687, 9688, 9689-9691, 9695, 9698- 9702, 9705, 9708-9709, 9712, 9714-9719, 9724-9729, 9735, 9737, 9738 |
|  | All sites except C024, C098-C099, C111, C142, C379, C420-C422, C424, C770-C779 | 9811-9818, 9823, 9827, 9837 |
| Chronic Lymphocytic Leukemia | C420, C421, C424 | 9823 |
| Myeloma | Any sites | 9731-9732, 9734 |
| Acute Lymphocytic Leukemia | Any sites | 9826, 9835-9836 |
|  | C420, C421, C424 | 9811-9818, 9837 |
| Acute Non-lymphocytic Leukemia |  |  |
| Acute Myeloid Leukemia | Any sites | 9840, 9861, 9865-9867, 9869, 9871-9874, 9895-9897, 9898, 9910-9911, 9920 |
| Acute Monocytic Leukemia | Any sites | 9891 |
| Other Acute Leukemia | Any sites | 9801, 9805-9809, 9931 |
| Chronic Myeloid Leukemia | Any sites | 9863, 9875-9876, 9945-9946 |
| Others^a^ |  |  |
| Mesothelioma |  | 9050-9055 |
| Kaposi Sarcoma |  | 9140 |
| Miscellaneous |  | 9740-9741, 9750-9769, 9950, 9960-9962, 9965-9967, 9970- 9971, 9975, 9980, 9982-9987, 9989, 9991-9992 |
|  | C760-C768, C809, C420-C424, C770-C779 | Any histologies excluding 9050- 9055, 9140, 9590-9992 |
| Other respiratory organs | C384 (Pleura), C339, C381-C383, C388, C390, C398, C399 (Trachea, Mediastinum and Other Respiratory Organs) | Any histologies excluding 9050- 9055, 9140, 9590-9992 |
| Peritoneum and other digestive organs | C480, C481-C482, C268-C269, C488 |  |
| Thymus, Adrenal Gland and Other Endocrine | C379, C740-C749, C750-C759 |  |
| Ureter and other unrainy organs | C669, C680-C689 |  |
| Other leukemia |  |  |
| Other Lymphocytic Leukemia | Any sites | 9820, 9832-9834, 9940 |
| Other Myeloid/Monocytic Leukemia | Any sites | 9860, 9930 |
| Aleukemic, Subleukemic and NOS | Any sites | 9733, 9742, 9800, 9831, 9870, 9948, 9963-9964 |
|  | C420, C421, C424 | 9827 |

Abbreviations: ICD-O-3, International Classification of Diseases for Oncology (third edition); NOS not otherwise specified.

**Supplementary table2. Characteristics of patients diagnosed with malignant peripheral nerve sheath tumor in the SEER database.**

| Characteristics | SEER 8 | SEER 12 | SEER 17 | SEER 22 | Total |
| --- | --- | --- | --- | --- | --- |
| Duration | 1975-2019 | 1992-2019 | 2000-2019 | 2000-2019 | 1975-2019 |
| No. | 1,689 | 1,584 | 2,645 | 4,711 | 5,694 |
| Sex, n (%) |  |  |  |  |  |
| Male | 897 (53.1) | 857 (54.1) | 1430 (54.1) | 2548 (54.1) | 3.064 (53.8) |
| Female | 792 (46.9) | 727(45.9) | 1215 (45.9) | 2163 (45.9) | 2,630 (46.2) |
| Age, n (%) |  |  |  |  |  |
| 00-19 | 169 (10) | 172 (10.9) | 259 (9.8) | 486 (10.3) | 580 (10.2) |
| 20-39 | 482 (28.5) | 441 (27.8) | 732 (27.7) | 1332 (28.3) | 1,617 (28.4) |
| 40-59 | 499 (29.5) | 494 (31.2) | 865 (32.7) | 1503 (31.9) | 1,768 (31.1) |
| ≥ 60 | 549 (32.5) | 487 (30.7) | 798 (30.2) | 1409 (29.9) | 1,748 (30.7) |
| Race, n (%) ^a^ |  |  |  |  |  |
| NHW | 1367 (80.9) | 942 (59.5) | 1622 (61.3) | 2913 (61.8) | 3670 (64.4) |
| NHB | 143 (8.5) | 163 (10.3) | 348 (13.2) | 676 (14.3) | 756 (13.3) |
| NHAPI | .. | 118 (7.4) | 212 (8) | 296 (6.3) | 336 (5.9) |
| Hispanic | .. | 271 (17.1) | 424 (16) | 766 (16.3) | 821 (14.4) |
| Income, n (%) |  |  |  |  |  |
| <50000 | 68 (4) | 64 (4) | 313 (11.8) | 615 (13.1) | .. |
| 50000-74999 | 617 (36.5) | 985 (6.2) | 1509 (57.1) | 2766 (58.7) | .. |
| >75000 | 487 (28.8) | 536 (33.8) | 827 (31.3) | 1339 (28.4) | .. |
| Location, n (%) |  |  |  |  |  |
| Urban | 971 (57.5) | 1,400 (88.4) | 2352 (88.9) | 4194 (89) | .. |
| Rural | 179 (10.6) | 165 (10.4) | 292 (11) | 516 (11) | .. |
| Size, n (%) ^b^ |  |  |  |  |  |
| 0-49 mm | .. | .. | 371 (14) | .. | .. |
| ≥50 mm | .. | .. | 2284 (86.4) | .. | .. |
| Site, n (%) |  |  |  |  |  |
| Head and neck | 211 (12.5) | 166 (10.5) | 566 (21.4) | 617 (13.1) | 682 (12.0) |
| Others | 1485 (87.9) | 1,418 (89.5) | 1117 (42.2) | 4114 (87.3) | 5012 (88.0) |
| Grade, n (%) |  |  |  |  |  |
| I | 80 (4.7) | 76 (4.8) | 147 (5.6) | 312 (6.6) | 355 (6.2) |
| II | 193 (11.4) | 211 (13.3) | 334 (12.6) | 532 (11.3) | 641 (11.3) |
| III-IV | 488 (28.9) | 604 (38.1) | 1010 (38.2) | 1643 (34.9) | 1808 (31.8) |
| Stage, n (%) ^c^ |  |  |  |  |  |
| Localize | 834 (49.4) | 670 (42.3) | 1361 (51.5) | 1997 (42.4) | 2,458 (43.2) |
| Regional | 397 (23.5) | 335 (21.1) | 656 (24.8) | 835 (17.7) | 1,039 (18.2) |
| Distant | 236 (14) | 201 (12.7) | 363 (13.7) | 534 (11.3) | 652 (11.5) |

Abbreviations: NHW, non-Hispanic White; NHB, non-Hispanic Black; NHAPI, non-Hispanic Asian and Pacific Islander.

Notes:

a. Analyses were limited in whites and blacks in SEER 8 due to the availability of only whites and blacks in race/ethnicity

b. The analysis of tumor size was limited in SEER 17 from 2004 to 2019 due to the unavailability in SEER 22 and the repeatability in SEER 8 and SEER 12.

c. The analysis of tumor stage in SEER 22 was limited form 2004 to 2019 due to the unavailability in SEER 22.

**Supplementary table3. Age-specific incidence rate and incidence rate ratio in malignant peripheral nerve sheath tumor during 2000-2019 in 17 and 22 SEER registries.**

| **Age** | **SEER 17** | | | **SEER 22** | | |
| --- | --- | --- | --- | --- | --- | --- |
|  | **Count**  **(No.)** | **Incidence rate**  **2000-2019**  **(95%CI)** | **Incidence rate ratio**  **(95%CI)** | **Count**  **(No.)** | **Incidence rate**  **2000-2019**  **(95%CI)** | **Incidence rate ratio**  **(95% CI)** |
| **00 years** | 3 | 0.01 (0.00 to 0.04) | 1 [Reference] | 8 | 0.02 (0.01 to 0.04) | 1 [Reference] |
| **01-04 years** | 20 | 0.02 (0.01 to 0.04) | 1.67 (0.50 to 8.79) | 35 | 0.02 (0.02 to 0.03) | 1.10 (0.50 to 2.74) |
| **05-09 years** | 35 | 0.03 (0.02 to 0.04) | 2.32 (0.73 to 11.78) | 68 | 0.03 (0.03 to 0.04) | 1.69 (0.81 to 4.08) |
| **10-14 years** | 74 | 0.07 (0.05 to 0.08) | 4.75* (1.56 to 23.58) | 134 | 0.07 (0.06 to 0.08) | 3.24 (1.60 to 7.66) |
| **15-19 years** | 127 | 0.11 (0.09 to 0.13) | 8.12* (2.72 to 39.88) | 241 | 0.12 (0.10 to 0.13) | 5.76* (2.88 to 13.50) |
| **20-24 years** | 166 | 0.14 (0.12 to 0.17) | 10.45* (3.51 to 51.17) | 306 | 0.15 (0.13 to 0.16) | 7.24* (3.63 to 16.92) |
| **25-29 years** | 168 | 0.15 (0.12 to 0.17) | 10.60* (3.57 to 51.91) | 316 | 0.15 (0.14 to 0.17) | 7.46* (3.74 to 17.44) |
| **30-34 years** | 180 | 0.16 (0.14 to 0.18) | 11.52* (3.88 to 56.39) | 329 | 0.16 (0.14 to 0.18) | 7.90* (3.96 to 18.45) |
| **35-39 years** | 215 | 0.19 (0.16 to 0.22) | 13.72* (4.63 to 67.07) | 371 | 0.18 (0.16 to 0.20) | 8.93* (4.48 to 20.83) |
| **40-44 years** | 212 | 0.18 (0.16 to 0.21) | 13.43* (4.53 to 65.62) | 359 | 0.17 (0.16 to 0.19) | 8.57* (4.30 to 20.00) |
| **45-49 years** | 218 | 0.19 (0.17 to 0.22) | 13.88* (4.69 to 67.83) | 389 | 0.19 (0.17 to 0.21) | 9.33* (4.69 to 21.76) |
| **50-54 years** | 207 | 0.19 (0.16 to 0.22) | 13.81* (4.66 to 67.53) | 373 | 0.19 (0.17 to 0.21) | 9.37* (4.71 to 21.86) |
| **55-59 years** | 224 | 0.23 (0.20 to 0.26) | 16.73* (5.65 to 81.72) | 375 | 0.21 (0.19 to 0.24) | 10.53* (5.29 to 24.58) |
| **60-64 years** | 182 | 0.23 (0.20 to 0.26) | 16.52* (5.56 to 80.83) | 326 | 0.23 (0.20 to 0.25) | 11.12* (5.58 to 25.98) |
| **65-69 years** | 162 | 0.26 (0.22 to 0.30) | 18.65* (6.27 to 91.36) | 299 | 0.26 (0.23 to 0.29) | 12.94* (6.48 to 30.24) |
| **70-74 years** | 146 | 0.30 (0.25 to 0.35) | 21.77* (7.30 to 106.8) | 252 | 0.29 (0.25 to 0.32) | 14.07* (7.04 to 32.96) |
| **75-79 years** | 141 | 0.37 (0.32 to 0.44) | 27.28* (9.15 to 133.85) | 241 | 0.35 (0.31 to 0.40) | 17.41* (8.69 to 40.78) |
| **80-84 years** | 82 | 0.30 (0.24 to 0.37) | 21.64* (7.15 to 107.09) | 155 | 0.31 (0.26 to 0.36) | 15.27* (7.56 to 35.99) |
| **85+ years** | 83 | 0.31 (0.25 to 0.39) | 22.66* (7.49 to 112.1) | 134 | 0.28 (0.23 to 0.33) | 13.58* (6.70 to 32.11) |
| ***P* for trend ^a^** | - | - | < .001 | - | - | < .001 |

Notes:

Test for linear trend with 19 age groups included as a continuous variable with poisson regression.

“*” indicates the rate ratio was significantly different from 1.

**Supplementary table4. Age-specific incidence rate and incidence rate ratio in malignant peripheral nerve sheath tumor by sex during 2000-2019 in 17 SEER registries.**

| **Age** | **Male** | | | **Female** | | |
| --- | --- | --- | --- | --- | --- | --- |
|  | **Count**  **(No.)** | **Incidence rate**  **2000-2019**  **(95%CI)** | **Incidence rate ratio**  **(95%CI)** | **Count**  **(No.)** | **Incidence rate**  **2000-2019**  **(95%CI)** | **Incidence rate ratio**  **(95% CI)** |
| **00 years** | 2 | 0.02 (0 to0.06) | 1 [Reference] | 1 | 0.01 (0 to0.05) | 0.52 (0.01 to10.05) |
| **01-04 years** | 10 | 0.02 (0.01 to0.04) | 1 [Reference] | 10 | 0.02 (0.01 to0.04) | 1.05 (0.39 to2.81) |
| **05-09 years** | 22 | 0.04 (0.02 to0.06) | 1 [Reference] | 13 | 0.02 (0.01 to0.04) | 0.62 (0.29 to1.28) |
| **10-14 years** | 36 | 0.06 (0.04 to0.09) | 1 [Reference] | 38 | 0.07 (0.05 to0.09) | 1.11 (0.68 to1.8) |
| **15-19 years** | 63 | 0.11 (0.08 to0.14) | 1 [Reference] | 64 | 0.12 (0.09 to0.15) | 1.07 (0.75 to1.55) |
| **20-24 years** | 101 | 0.17 (0.14 to0.21) | 1 [Reference] | 65 | 0.12 (0.09 to0.15) | 0.69 (0.49 to0.95) * |
| **25-29 years** | 100 | 0.17 (0.14 to0.21) | 1 [Reference] | 68 | 0.12 (0.09 to0.15) | 0.71 (0.51 to0.97) * |
| **30-34 years** | 102 | 0.18 (0.14 to0.22) | 1 [Reference] | 78 | 0.14 (0.11 to0.17) | 0.78 (0.58 to1.06) |
| **35-39 years** | 110 | 0.19 (0.16 to0.23) | 1 [Reference] | 105 | 0.18 (0.15 to0.22) | 0.96 (0.73 to1.26) |
| **40-44 years** | 115 | 0.2 (0.17 to0.24) | 1 [Reference] | 97 | 0.17 (0.14 to0.21) | 0.84 (0.63 to1.11) |
| **45-49 years** | 110 | 0.19 (0.16 to0.23) | 1 [Reference] | 108 | 0.19 (0.15 to0.23) | 0.96 (0.73 to1.27) |
| **50-54 years** | 95 | 0.18 (0.14 to0.22) | 1 [Reference] | 112 | 0.2 (0.17 to0.24) | 1.14 (0.86 to1.51) |
| **55-59 years** | 128 | 0.27 (0.23 to0.32) | 1 [Reference] | 96 | 0.19 (0.16 to0.23) | 0.71 (0.54 to0.93) * |
| **60-64 years** | 103 | 0.27 (0.22 to0.33) | 1 [Reference] | 79 | 0.19 (0.15 to0.24) | 0.71 (0.52 to0.96) * |
| **65-69 years** | 86 | 0.29 (0.23 to0.36) | 1 [Reference] | 76 | 0.23 (0.18 to0.28) | 0.78 (0.56 to1.07) |
| **70-74 years** | 81 | 0.37 (0.29 to0.45) | 1 [Reference] | 65 | 0.24 (0.19 to0.31) | 0.67 (0.47 to0.94) * |
| **75-79 years** | 79 | 0.49 (0.39 to0.61) | 1 [Reference] | 62 | 0.29 (0.22 to0.37) | 0.59 (0.42 to0.84) * |
| **80-84 years** | 45 | 0.41 (0.3 to0.55) | 1 [Reference] | 37 | 0.22 (0.16 to0.31) | 0.55 (0.34 to0.87) * |
| **85+ years** | 42 | 0.47 (0.34 to0.64) | 1 [Reference] | 41 | 0.23 (0.17 to0.31) | 0.49 (0.31 to0.77) * |

“*” indicates the rate ratio was significantly different from 1.

**Supplementary table5. Age-standardized incidence and rate ratio in malignant peripheral nerve sheath tumor by demographic during 2000-2019 in 17 SEER registries.**

| Characteristics | **Overall** | | | **0-39 years** | | **≥ 40 years** | | **P**  **for**  **heterogeneity** |
| --- | --- | --- | --- | --- | --- | --- | --- | --- |
|  | **No.** | **Incidence rate**  **2000-2019**  **(95%CI)** | **Incidence rate ratio**  **(95%CI)** | **Incidence rate**  **2000-2019**  **(95%CI)** | **Incidence rate ratio**  **(95%CI)** | **Incidence rate**  **2000-2019**  **(95%CI)** | **Incidence rate ratio**  **(95%CI)** |  |
| Overall | 2,645 | 0.16 (0.16 to 0.17) | .. |  | .. |  | .. |  |
| Age |  |  |  |  |  |  |  |  |
| 0-19 | 259 | 0.06 (0.05 to 0.07) | Reference | .. | .. | .. | .. |  |
| 20-39 | 729 | 0.16 (0.15 to 0.17) | 2.78 (2.41 to 3.21) * | .. | .. | .. | .. |  |
| 40-59 | 861 | 0.20 (0.18 to 0.21) | 3.39 (2.94 to 3.91) * | .. | .. | .. | .. |  |
| ≥ 60 | 796 | 0.29 (0.27 to 0.31) | 4.96 (4.30 to 5.73) * | .. | .. | .. | .. |  |
| Sex |  |  |  |  |  |  |  |  |
| Male | 1,430 | 0.18 (0.17 to 0.19) | Reference | 0.12 (0.11 to 0.13) | Reference | 0.27 (0.25 to 0.29) | Reference | .. |
| Female | 1,215 | 0.14 (0.14 to 0.15) | 0.80 (0.74, 0.86) | 0.10 (0.09 to 0.11) | 0.85 (0.75 to 0.96) * | 0.20 (0.19 to 0.22) | 0.76 (0.69 to 0.84) * | 0.165 |
| Race |  |  |  |  |  |  |  |  |
| NHW | 1,622 | 0.17 (0.16 to 0.17) | Reference | 0.11 (0.10 to 0.12) | Reference | 0.24 (0.23 to 0.26) | Reference | .. |
| NHB | 348 | 0.20 0(0.18 to 0.22) | 1.21 (1.07 to 1.36)) * | 0.14 (0.12 to 0.16) | 1.31 (1.08 to 1.57) * | 0.28 (0.24 to 0.33) | 1.16 (0.99 to 1.36) | 0.331 |
| NHAPI | 212 | 0.13 (0.11 to 0.15) | 0.77 (0.67 to 0.90) * | 0.10 (0.08 to 0.12) | 0.91 (0.72 to 1.14) | 0.17 (0.14 to 0.20) | 0.69 (0.57 to 0.84) * | 0.071 |
| Hispanic | 424 | 0.13 (0.12 to 0.15) | 0.80 (0.71 to 0.90) * | 0.10 (0.09 to 0.11) | 0.92 (0.78 to 1.07) | 0.18 (0.15 to 0.21) | 0.73 (0.62 to 0.86) * | 0.046 |
| Income |  |  |  |  |  |  |  |  |
| <50000 | 310 | 0.16 (0.14 to 0.18) | Reference | 0.12 (0.10 to 0.14) | Reference | 0.22 (0.19 to 0.25) | Reference | .. |
| 50000-74999 | 1,509 | 0.16 (0.16 to 0.17) | 1.04 (0.92 to 1.18) | 0.11 (0.10 to 0.12) | 0.97 (0.79 to 1.19) | 0.23 (0.22 to 0.25) | 1.09 (0.93 to 1.28) | 0.379 |
| >75000 | 826 | 0.16 (0.15 to 0.17) | 1.00 (0.88 to 1.15) | 0.10 (0.09 to 0.11) | 0.88 (0.71 to 1.10) | 0.23 (0.21 to 0.25) | 1.09 (0.92 to 1.29) | 0.129 |
| Location |  |  |  |  |  |  |  |  |
| Urban | 2,352 | 0.16 (0.15 to 0.17) | Reference | 0.11 (0.10 to 0.12) | Reference | 0.23 (0.22 to 0.24) | Reference | .. |
| Rural | 292 | 0.17 (0.15 to 0.19) | 1.04 (0.92 to 1.18) | 0.12 (0.09 to 0.14) | 1.07 (0.86 to 1.32) | 0.24 (0.21 to 0.28) | 1.03 (0.88 to 1.20) | 0.778 |

Abbreviations: NHW, non-Hispanic White; NHB, non-Hispanic Black; NHAPI, non-Hispanic Asian and Pacific Islander.

“*” indicates the rate ratio was significantly different from 1.

**Supplementary table6. Age-standardized incidence and rate ratio in malignant peripheral nerve sheath tumor by registry during 2000-2019 in 22 SEER registries**

| **Registriy** | **Count**  **(No.)** | **Incidence rate**  **2000-2019 (95%CI)** | **Incidence rate ratio**  **(95% CI)** | ***P* value** |
| --- | --- | --- | --- | --- |
| **Texas ^a^** | 682 | 0.14 (0.13 to 0.15) | 1 [Reference] | .. |
| **New York** | 717 | 0.18 (0.17 to 0.19) | 1.28 (1.15 to 1.42) * | <.001 |
| **San Francisco-oakland SMSA** | 109 | 0.12 (0.10 to 0.14) | 0.85 (0.69 to 1.04) | 0.126 |
| **Connecticut** | 128 | 0.17 (0.14 to 0.20) | 1.21 (0.99 to 1.47) | 0.058 |
| **Hawaii** | 52 | 0.19 (0.14 to 0.25) | 1.34 (0.99 to 1.79) | 0.060 |
| **Iowa** | 142 | 0.22 (0.19 to 0.26) | 1.6 (1.32 to 1.93) * | <.001 |
| **New Mexico** | 47 | 0.12 (0.09 to 0.16) | 0.85 (0.61 to 1.14) | 0.306 |
| **Seattle (Puget Sound)** | 157 | 0.16 (0.14 to 0.19) | 1.18 (0.98 to 1.4) | 0.081 |
| **Utah** | 100 | 0.2 (0.16 to 0.24) | 1.41 (1.12 to 1.74) * | 0.003 |
| **Atlanta (Metropolitan)** | 98 | 0.15 (0.12 to 0.18) | 1.07 (0.85 to 1.33) | 0.581 |
| **Alaska Natives** | 1 | 0.05 (0.00 to 0.29) | 0.33 (0.01 to 2.11) | 0.477 |
| **San Jose-Monterey** | 78 | 0.15 (0.12 to 0.19) | 1.08 (0.84 to 1.36) | 0.577 |
| **Los Angeles** | 309 | 0.16 (0.14 to 0.18) | 1.14 (0.99 to 1.3) | 0.068 |
| **Rural Georgia** | 5 | 0.21 (0.07 to 0.50) | 1.53 (0.47 to 3.61) | 0.487 |
| **California excluding SF/SJM/LA** | 622 | 0.16 (0.14 to 0.17) | 1.12 (1 to 1.25) * | 0.046 |
| **Kentucky** | 125 | 0.14 (0.12 to 0.17) | 1 (0.82 to 1.22) | >.99 |
| **Louisiana** | 169 | 0.19 (0.16 to 0.22) | 1.33 (1.11 to 1.58) * | 0.002 |
| **New Jersey** | 334 | 0.18 (0.16 to 0.20) | 1.31 (1.15 to 1.5) * | < .001 |
| **Greater Georgia** | 169 | 0.14 (0.12 to 0.16) | 0.99 (0.83 to 1.18) | 0.977 |
| **Idaho** | 53 | 0.17 (0.13 to 0.22) | 1.2 (0.89 to 1.6) | 0.232 |
| **Massachusetts** | 241 | 0.18 (0.15 to 0.20) | 1.26 (1.08 to 1.46) * | 0.004 |
| **Illinois** | 373 | 0.14 (0.13 to 0.16) | 1.03 (0.9 to 1.17) | 0.666 |

**Notes:**

Texas was used as reference due to the largest population.

**Supplementary table7. Trends in incidence rate of malignant peripheral nerve sheath tumor among different subgroups in 17 SEER registries during 2000-2019.**

| **Characteristics** | **Cases**  **(No.)** | **Incidence rate (95% CI) ^c^** | | **AAPC ^d^**  **(95% CI)^c^** | ***P* value ^e^** |
| --- | --- | --- | --- | --- | --- |
|  |  | **2000** | **2019** |  |  |
| **Overall** | 2,645 | 0.14 (0.12 to 0.18) | 0.13 (0.11 to 0.16) | -2.2* (-3.1 to -1.3) | .. |
| **Sex** |  |  |  |  |  |
| Male | 1,430 | 0.14 (0.10 to 0.19) | 0.16 (0.12 to 0.20) | -2.8* (-4.0 to -1.5) | Reference |
| Female | 1,215 | 0.15 (0.11 to 0.19) | 0.11(0.08 to 0.15) | -1.5* (-2.6 to -0.4) | .126 |
| **Age** |  |  |  |  |  |
| 00-19 | 259 | 0.03 (0.02 to 0.07) | 0.06 (0.04 to 0.11) | -0.2 (-2.9 to 2.5) | .. |
| 20-39 | 732 | 0.13 (0.09 to 0.19) | 0.17 (0.12 to 0.23) | -0.8 (-2.0 to 0.3) | .. |
| 40-59 | 865 | 0.17 (0.12 to 0.23) | 0.13 (0.09 to 0.19) | -2.8* (-4.2 to -1.4) | Reference |
| ≥60 | 798 | 0.31 (0.22 to 0.44) | 0.20 (0.14 to 0.28) | -3.4* (-5.2 to -1.5) | .612 |
| **Race ^a^** |  |  |  |  |  |
| NHW | 1,622 | 0.15 (0.12 to 0.19) | 0.13 (0.10 to 0.17) | -2.7* (-3.9 to -1.5) | .. |
| NHB | 348 | 0.11 (0.04 to 0.22) | 0.17 (0.10 to 0.28) | -0.3 (-1.8 to 1.2) | .. |
| NHAPI | 212 | 0.19 (0.08 to 0.36) | 0.10 (0.05 to 0.19) | -1.3 (-3.7 to 1.2) | .. |
| Hispanic | 424 | 0.16 (0.08 to 0.27) | 0.12 (0.08 to 0.19) | -1.6 (-3.6 to 0.5) | .. |
| **Income** |  |  |  |  |  |
| < 50000 | 313 | 0.15 (0.08 to 0.27) | 0.12 (0.06 to 0.21) | -2.1* (-3.8 to -0.3) | Reference |
| 50000-74999 | 1,509 | 0.12 (0.09 to 0.16) | 0.12 (0.09 to 0.16) | -2.3* (-3.8 to -0.8) | .865 |
| ≥75000 | 827 | 0.18 (0.13 to 0.24) | 0.15 (0.11 to 0.20) | -1.0 (-4.8 to 3.0) | .. |
| **Location** |  |  |  |  |  |
| Urban | 2,352 | 0.15 (0.12 to 0.18) | 0.14 (0.11 to 0.17) | -2.2* (-3.1 to -1.3) | Reference |
| Rural | 292 | 0.12 (0.06 to 0.22) | 0.10 (0.05 to 0.20) | -2.7* (-5.0 to -0.4) | .692 |
| **ICD code** |  |  |  |  |  |
| 9540/3 | 2189 | 0.08 (0.06 to 0.10) | 0.11 (0.09 to 0.14) | -1.3* (-2.5 to -0.0) | Reference |
| Others | 462 | 0.07 (0.05 to 0.09) | 0.02 (0.01 to 0.03) | -5.9* (-9.6 to -2.0) | .024 |
| **Site ^b^** |  |  |  |  |  |
| Head and neck | 371 | 0.02 (0.01 to 0.04) | 0.02 (0.01 to 0.03) | -3.8* (-5.6 to -1.7) | Reference |
| Others | 2,284 | 0.12 (0.10 to 0.15) | 0.12 (0.10 to 0.14) | -2.0* (-2.9 to -1.0) | .104 |
| **Size** |  |  |  |  |  |
| 0-49 mm | 566 | 0.05 (0.04 to 0.07) | 0.02 (0.01 to 0.04) | -5.4 (-12.0 to 1.8) | .. |
| ≥50 mm | 1117 | 0.09 (0.07 to 0.11) | 0.09 (0.07 to 0.11) | -0.9 (-2.1 to 0.3) | .. |
| **Grade** |  |  |  |  |  |
| I | 147 | 0.01 (0.00 to 0.02) | 0.01 (0.00 to 0.01) | -1.2 (-3.9 to 1.6) | .. |
| II | 334 | 0.03 (0.02 to 0.04) | 0.02 (0.01 to 0.03) | -3.8* (-5.6 to -2.1) | Reference |
| III-IV | 1,010 | 0.04 (0.03 to 0.06) | 0.05 (0.04 to 0.07) | -1.5* (-2.8 to -0.3) | .036 |
| **Stage** |  |  |  |  |  |
| Localize | 1,361 | 0.06 (0.04 to 0.08) | 0.08 (0.06 to 0.10) | -0.8 (-2.4 to 0.9) | .. |
| Regional | 656 | 0.03 (0.02 to 0.05) | 0.02 (0.01 to 0.04) | -3.5* (-5.3 to -1.7) | Reference |
| Distant | 363 | 0.02 (0.01 to 0.03) | 0.02 (0.01 to 0.04) | 0.3 (-1.3 to 2.0) | .. |
| Unknown | 208 | 0.04 (0.03 to 0.06) | 0.01 (0.00 to 0.01) | -8.1* (-13.9 to -1.8) | .153 |
| Abbreviations: AAPC, average annual percentage change; NHW, non-Hispanic White; NHB, non-Hispanic Black; NHAPI, non-Hispanic Asian and Pacific Islander.  Notes:  The analyses of race limited in non-Hispanic White, non-Hispanic Black, non-Hispanic Asian and Pacific Islander and Hispanic. Participants with non-Hispanic American Indian/Alaska Native and non-Hispanic unknown were excluded.  The analyses of tumor size limited from 2004 to 2019 due to the unavailability before 2004.  The incidence rate was age-adjusted and adjusted for reporting delay (race and cancer type), where the 95% CI was calculated using Tiwari’s method.  The AAPC was generated as a weighted average of APCs during the study period.  Difference in AAPC was examined using Student’s *t* test among subpopulations with significant AAPCs.  “*” indicates the AAPC was significantly different from zero. | | | | | |

**Supplementary table8. Trends in incidence rate of malignant peripheral nerve sheath tumor among different subgroups in 22 SEER registries during 2000-2019.**

| **Characteristics** | **Cancer cases**  **(No.)** | **Incidence rate (95% CI) ^b^** | | **AAPC ^c^**  **(95% CI)^c^** | ***P* value ^d^** |
| --- | --- | --- | --- | --- | --- |
|  |  | **2000** | **2019** |  |  |
| **Overall** | | | | | |
| **Overall** | 4,711 | 0.13 (0.11 to 0.16) | 0.14 (0.13 to 0.16) | -1.9* (-2.8 to -1.0) | .. |
| **Sex** |  |  |  |  |  |
| Male | 2,548 | 0.13 (0.11 to 0.17) | 0.17 (0.14 to 0.20) | -2.1* (-3.2 to -1.0) | Reference |
| Female | 2,163 | 0.14 (0.11 to 0.17) | 0.12 (0.10 to 0.15) | -1.7* (-2.7 to -0.6) | .606 |
| **Age** |  |  |  |  |  |
| 00-19 | 486 | 0.04 (0.02 to 0.06) | 0.06 (0.04 to 0.09) | -1.3 (-3.7 to 1.1) | .. |
| 20-39 | 1,332 | 0.14 (0.10 to 0.18) | 0.18 (0.14 to 0.22) | -0.5 (-1.4 to 0.4) | .. |
| 40-59 | 1,503 | 0.13 (0.10 to 0.18) | 0.15 (0.12 to 0.20) | -2.4* (-3.8 to -1.0) | Reference |
| ≥60 | 1,409 | 0.30 (0.23 to 0.39) | 0.23 (0.18 to 0.30) | -2.7* (-4.0 to -1.3) | .762 |
| **Race ^a^** |  |  |  |  |  |
| NHW | 2,913 | 0.13 (0.11 to 0.16) | 0.14 (0.11 to 0.16) | -2.4* (-3.5 to -1.2) | Reference |
| NHB | 676 | 0.15 (0.09 to 0.23) | 0.21 (0.14 to 0.28) | -0.0 (-1.4 to 1.3) | .. |
| NHAPI | 296 | 0.16 (0.07 to 0.30) | 0.10 (0.05 to 0.16) | -1.2 (-2.9 to 0.7) | .. |
| Hispanic | 766 | 0.13 (0.08 to 0.20) | 0.14 (0.10 to 0.18) | -1.6* (-3.1 to -0.1) | .407 |
| **Income** |  |  |  |  |  |
| < 50000 | 615 | 0.13 (0.08 to 0.20) | 0.16 (0.10 to 0.23) | -1.5* (-3.0 to -0.0) | Reference |
| 50000-74999 | 2,766 | 0.11 (0.10 to 0.14) | 0.14 (0.12 to 0.17) | -1.8* (-3.0 to -0.6) | .760 |
| ≥75000 | 1,339 | 0.17 (0.13 to 0.22) | 0.15 (0.12 to 0.19) | -2.2* (-3.2 to -1.2) | .447 |
| **Location** |  |  |  |  |  |
| Urban | 4,194 | 0.14 (0.12 to 0.16) | 0.14 (0.13 to 0.17) | -1.9* (-2.8 to -1.0) | Reference |
| Rural | 516 | 0.11 (0.07 to 0.18) | 0.13 (0.08 to 0.20) | -2.0* (-3.8 to -0.1) | .924 |
| **Site** |  |  |  |  |  |
| Head and neck | 617 | 0.02 (0.01 to 0.03) | 0.02 (0.01 to 0.03) | -2.4* (-3.8 to -0.8) | Reference |
| Others | 4,114 | 0.11 (0.10 to 0.13) | 0.12 (0.10 to 0.14) | -1.8* (-2.8 to -0.8) | .514 |
| **Grade** |  |  |  |  |  |
| I | 312 | 0.01 (0.00 to 0.02) | 0.01 (0.01 to 0.02) | -2.4 (-4.9 to 0.2) | .. |
| II | 532 | 0.02 (0.01 to 0.03) | 0.02 (0.01 to 0.03) | -2.4* (-4.1 to -0.6) | .. |
| III-IV | 1,643 | 0.04 (0.03 to 0.05) | 0.05 (0.04 to 0.06) | -1.4 (-2.7 to -0.2) | .. |
| **Stage ^e^** |  |  |  |  |  |
| Localize | 1,997 | 0.09 (0.07 to 0.10) | 0.08 (0.06 to 0.09) | -1.8* (-2.9 to -0.6) | Reference |
| Regional | 835 | 0.05 (0.04 to 0.09) | 0.03 (0.01 to 0.03) | -4.6* (-6.8 to -2.5) | .024 |
| Distant | 534 | 0.02 (0.01 to 0.03) | 0.03 (0.02 to 0.04) | 1.4 (-4.7 to 7.9) | .. |
| Unknown | 1,356 | 0.02 (0.01 to 0.03) | 0.01 (0.01 to 0.02) | -11.6 (-24.1 to 2.9) | .. |
| **00-39 years** | | | | | |
| **Overall** | 988 | 0.09 (0.07 to 0.11) | 0.12 (0.09 to 0.14) | -0.8 (-1.8 to 0.2) | .. |
| **Sex** |  |  |  |  |  |
| Male | 546 | 0.09 (0.06 to 0.12) | 0.14 (0.11 to 0.18) | -0.9 (-2.4 to 0.6) | .. |
| Female | 442 | 0.09 (0.06 to 0.12) | 0.09 (0.06 to 0.13) | -0.7 (-2.1 to 0.8) | .. |
| **Race ^a^** |  |  |  |  |  |
| NHW | 477 | 0.08 (0.06 to 0.11) | 0.10 (0.07 to 0.13) | -1.6 (-3.0 to -0.1) * | .. |
| NHB | 153 | 0.14 (0.08 to 0.24) | 0.19 (0.12 to 0.30) | 0.6 (-1.0 to 2.3) | .. |
| NHAPI | 94 | 0.07 (0.02 to 0.19) | 0.07 (0.03 to 0.15) | 1.0 (-2.1 to 4.1) | .. |
| Hispanic | 245 | 0.08 (0.04 to 0.13) | 0.13 (0.09 to 0.18) | -0.7 (-2.5 to 1.1) | .. |
| **Stage ^e^** |  |  |  |  |  |
| Localize | 795 | 0.06 (0.04 to 0.08) | 0.06 (0.04 to 0.08) | 0.1 (-1.8 to 2.0) | .. |
| Regional | 336 | 0.04 (0.03 to 0.05) | 0.02 (0.01 to 0.04) | -3.7 (-6.3 to -1.0) * | .. |
| Distant | 200 | 0.02 (0.01 to 0.03) | 0.03 (0.01 to 0.04) | 1.6 (-4.1, 7.8) | .. |
| **≥ 40 years** | | | | | |
| **Overall** | 1661 | 0.20 (0.16 to 0.24) | 0.18 (0.15 to 0.22) | -2.6 (-3.6 to -1.6) * | .. |
| **Sex** |  |  |  |  |  |
| Male | 887 | 0.19 (0.14 to 0.26) | 0.22 (0.17 to 0.27) | -2.9 (-4.1 to -1.6) * | Reference |
| Female | 774 | 0.20 (0.15 to 0.26) | 0.16 (0.12 to 0.21) | -2.3 (-3.6 to -1.0) * |  |
| **Race ^a^** |  |  |  |  |  |
| NHW | 1146 | 0.20 (0.16 to 0.25) | 0.19 (0.15 to 0.24) | -2.9 (-4.0 to -1.7) * | .. |
| NHB | 196 | 0.17 (0.07 to 0.33) | 0.22 (0.13 to 0.36) | -0.1 (-2.8 to 1.6) | .. |
| NHAPI | 118 | 0.28 (0.10 to 0.60) | 0.13 (0.05 to 0.25) | -2.2 (-4.5 to 0.1) | .. |
| Hispanic | 181 | 0.20 (0.09 to 0.37) | 0.15 (0.09 to 0.24) | -2.1 (-4.3 to 0.2) | .. |
| **Stage ^e^** |  |  |  |  |  |
| Localize | 1204 | 0.12 (0.09 to 0.15) | 0.09 (0.07 to 0.12) | -2.8 (-4.7 to -1.0) * | Reference |
| Regional | 500 | 0.07 (0.05 to 0.10) | 0.03 (0.01 to 0.02) | -5.5 (-7.3 to -3.6) * |  |
| Distant | 334 | 0.03 (0.02 to 0.04) | 0.04 (0.02 to 0.06) | -0.2 (-2.9 to 2.6) | .. |
| Abbreviations: AAPC, average annual percentage change; NHW, non-Hispanic White; NHB, non-Hispanic Black; NHAPI, non-Hispanic Asian and Pacific Islander.  Notes:  The analyses of race limited in non-Hispanic White, non-Hispanic Black, non-Hispanic Asian and Pacific Islander and Hispanic. Participants with non-Hispanic American Indian/Alaska Native and non-Hispanic unknown were excluded.  The incidence rate was age-adjusted and adjusted for reporting delay (race and cancer type), where the 95% CI was calculated using Tiwari’s method.  The AAPC was generated as a weighted average of APCs during the study period.  Difference in AAPC was examined using Student’s *t* test among subpopulations with significant AAPCs.  The analysis of tumor stage in SEER 22 was limited form 2004 to 2019 due to the unavailability in SEER 22.  “*” indicates the AAPC was significantly different from zero. | | | | | |

**Supplementary table9. Trends in incidence rate of malignant peripheral nerve sheath tumor among different subgroups in 8 SEER registries during 1975-2019.**

| **Characteristics** | **Cases, (No.)** | **Annual percentage changes** | | | | | | | | |
| --- | --- | --- | --- | --- | --- | --- | --- | --- | --- | --- |
|  |  | **Join points** | | | **APC1**  **(95%CI)** | **APC2**  **(95%CI)** | **APC3**  **(95%CI)** | **APC4**  **(95%CI)** | **AAPC 1975-2019 ^d^**  **(95%CI)** | **AAPC 2001-2019 ^d^**  **(95% CI)** |
| **Overall** | 1,689 | 1990 | 1998 | 2001 | 1.4 (-1.1 to 3.9) | -8.6* (-15.5 to -1.1) | 24.0 (-33.1 to 129.9) | -3.2* (-4.9 to -1.6) | -1.0 (-5.3 to 3.4) | -3.2* (-4.9 to -1.6) |
| **Sex** |  |  |  |  |  |  |  |  |  |  |
| Male | 897 | - | - | - | -1.0* (-1.6 to -0.3) | - | - | - | -1.0* (-1.6 to -0.3) | -1.0* (-1.6 to -0.3) |
| Female | 792 | - | - | - | -1.2* (-1.9 to -0.5) | - | - | - | -1.2* (-1.9 to -0.5) | -1.2* (-1.9 to -0.5) |
| **Age** |  |  |  |  |  |  |  |  |  |  |
| 0-19 | 169 | .. | .. | .. | .. | .. | .. | .. | .. | .. |
| 20-39- | 482 | - | - | - | -0.2 (-0.9 to 0.4) | - | - | - | -0.2 (-0.9 to 0.4) | -0.2 (-0.9 to 0.4) |
| 40-59 | 499 | 1999 | 2006 | - | -2.4* (-4.6 to -0.2) | 7.7 (-6.2 to 23.7) | -7.6* (-11.7 to -3.3) | - | -2.5 (-5.1 to 0.3) | -3.6 (-8.2 to 1.2) |
| ≥ 60 | 549 | - | - | - | -2.0*(-2.8 to -1.1) | - | - | - | -2.0*(-2.8 to -1.1) | -2.0*(-2.8 to -1.1) |
| **Race/ethnicity ^a^** |  |  |  |  |  |  |  |  |  |  |
| White | 1,367 | 1990 | 1998 | 2001 | 1.3 (-1.3 to 3.9) | -9.2* (-16.7 to -1.1) | 29.3 (-32.9 to 149.2) | -3.8* (-5.7 to -2.0) | -1.2 (-5.7 to 3.6) | -3.8* (-5.7 to -2.0) |
| Black | 143 | .. | .. | .. | .. | .. | .. | .. | .. | .. |
| **Income ^b^** |  |  |  |  |  |  |  |  |  |  |
| < 50000 | 68 | .. | .. | .. | .. | .. | .. | .. | .. | .. |
| 50000-74999 | 617 | - | - | - | -2.0* (-3.4 to -0.5) | - | - | - | -2.0* (-3.4 to -0.5) | -2.0* (-3.4 to -0.5) |
| ≥75000 | 487 | - | - | - | -0.6 (-1.9 to 0.7) | - | - | - | -0.6 (-1.9 to 0.7) | -0.6 (-1.9 to 0.7) |
| **Location ^b^** |  |  |  |  |  |  |  |  |  |  |
| Urban | 971 | 1998 | 2001 | - | -10.0* (-15.8 to -3.7) | 26.5(-34.8 to 145.4) | -3.3* (-5.0 to -1.5) | - | -2.5 (-8.9 to 4.4) | -3.3* (-5.0 to -1.5) |
| Rural | 179 | - | - | - | -1.1 (-3.0 to 0.8) | - | - | - | -1.1 (-3.0 to 0.8) | -1.1 (-3.0 to 0.8) |
| **Site** |  |  |  |  |  |  |  |  |  |  |
| Head and neck | 211 | 2007 | - | - | -0.3 (-2.0 to 1.5) | -8.3 (-16.1 to 0.3) |  |  | -2.5 (-5.1 to 0.1) | -5.7* (-11.0 to -2.0) |
| Others | 1,485 | - | - | - | -1.0* (-1.5 to -0.4) | - | - |  | -1.0* (-1.5 to -0.4) | -1.0* (-1.5 to -0.4) |
| **Stage** |  |  |  |  |  |  |  |  |  |  |
| Localize | 834 | 1993 | 1997 | 2004 | 0.8 (-1.4 to 3.1) | -16.0 (-40.2 to 17.9) | 8.3* (1.1 to 16.1) | -4.2* (-7.1 to -1.1) | -0.9 (-4.3 to 2.7) | -0.8 (-3.6 to 2.0) |
| Regional | 397 | - | - | - | 0.1 (-1.1 to 1.3) | - | - | - | 0.1 (-1.1 to 1.3) | 0.1 (-1.1 to 1.3) |
| Distant | 236 | .. | .. | .. | .. | .. | .. | .. | .. | .. |
| Unknown | 241 | - | - | - | .. | .. | .. | .. | .. | .. |

The analyses of race limited in White and Black due to the availability of only whites and blacks in race/ethnicity in SEER 8.

Analyses of income and geographic location limited from 1990 to 2019 due to the unavailability before 1990.

a. The incidence rate was age-adjusted and adjusted for reporting delay (race and cancer type), where the 95% CI was calculated using Tiwari’s method.

b. The AAPC was generated as a weighted average of APCs during the study period.

c. Due to the joinpoint of 2001 in overall trend, the stratified analyses were limited from 2001 to 2019.

“*” indicates the AAPC was significantly different from zero.

**Supplementary table10. Trends in incidence-based mortality rate of malignant peripheral nerve sheath tumor among different subgroups in 17 SEER registries during 2000-2019.**

|  | **Cases**  **No.** | **Incidence** | | **Annual percentage changes (95% CI)** | | | | | | |
| --- | --- | --- | --- | --- | --- | --- | --- | --- | --- | --- |
|  |  | **2000** | **2019** | **Join point** | | **APC 1** | **APC 2** | **APC3** | **AAPC 2000-2019** | **AAPC 2008-2019** |
| **Overall** | 1,442 | 0.02 (0.01 to 0.03) | 0.09 (0.07 to 0.11) | 2002 | 2008 | 90.8* (11.5 to 226.8) | 4.9 (-1.7 to 11.8) | -1.6 (-3.3 to 0.2) | 7.7* (1.9 to 13.7) | -1.6 (-3.3 to 0.2) |
| **Sex** |  |  |  |  |  |  |  |  |  |  |
| Male | 818 | 0.03 (0.01to 0.05) | 0.08 (0.05 to 0.11) | 2003 | - | 65.3* (18.0 to 131.4) | -1.2 (-2.7 to 0.4) | - | 7.2* (1.9 to 12.7) | -1.2 (-2.7 to 0.4) |
| Female | 624 | 0.01 (0.00 to 0.03) | 0.10 (0.07to 0.13) | 2002 | - | 91.1 (-34.6 to 458.5) | 1.2 (-0.6 to 3.1) | - | 8.2 (-2.5 to 20.2) | 1.2 (-0.6 to 3.1) |
| **Age** |  |  |  |  |  |  |  |  |  |  |
| 0-19 | 81 | 0.00 (0.00 to 0.03) | 0.01 (0.00 to 0.04) | - | - | 2.5 (-2.6 to 7.8) | - | - | 2.5 (-2.6 to 7.8) | 2.5 (-2.6 to 7.8) |
| 20-39- | 342 | 0.02 (0.01 to 0.05) | 0.11 (0.07 to 0.16) | 2002 | - | 130.4 (-46.0 to 883.5) | 0.7 (-1.8 to 3.3) | - | 9.9 (-4.6 to 26.7) | 0.7 (-1.8 to 3.3) |
| 40-59 | 410 | 0.02 (0.01 to 0.05) | 0.07 (0.04 to 0.12) | 2009 | - | 8.8* (2.6 to 15.3) | -5.6* (-9.6 to -1.5) | - | 0.9 (-2.3 to 4.3) | -4.4* (-7.8 to -0.9) |
| ≥ 60 | 609 | 0.04 (0.01 to 0.10) | 0.20 (0.14 to 0.28) | 2003 | - | 56.1 (-1.5 to 147.3) | 0.1 (-1.7 to 2.0) | - | 7.4* (0.3 to 15.0) | 0.1 (-1.7 to 2.0) |
| **Race/ethnicity** |  |  |  |  |  |  |  |  |  |  |
| NHW | 924 | 0.02 (0.01 to 0.04) | 0.08 (0.05 to 0.10) | 2002 | 2007 | 89.4* (10.1 to 225.8) | 6.9 (-2.7 to 17.5) | -2.6* (-4.3 to -0.9) | 7.1* (1.2 to 13.3) | -2.6* (-4.3 to -0.9) |
| NHB | 193 | 0.00 (0.00 to 0.06) | 0.13 (0.07 to 0.23) | .. | .. | .. | .. | .. | .. | .. |
| NHAPI | 96 | 0.02 (0.00 to 0.09) | 0.07 (0.03 to 0.15) | - | - | 2.7 (-0.0 to 5.6) | - | - | 2.7 (-0.0 to 5.6) | 2.7 (-0.0 to 5.6) |
| Hispanic | 215 | 0.02 (0.00 to 0.09) | 0.07 (0.04 to 0.12) | - | - | 2.2 (-0.5 to 5.0) | - | - | 2.2 (-0.5 to 5.0) | 2.2 (-0.5 to 5.0) |
| **Income *** |  |  |  |  |  |  |  |  |  |  |
| < 50000 | 193 | 0.03 (0.00 to 0.1) | 0.08 (0.04 to 0.16) | - | - | 1.3 (-1.9 to 4.5) | - | - | 1.3 (-1.9 to 4.5) | 1.3 (-1.9 to 4.5) |
| 50000-74999 | 820 | 0.02 (0.01 to 0.03) | 0.09 (0.07 to 0.12) | 2002 | - | 134.7 (-4.2 to 475.4) | -0.1 (-1.5 to 1.4) | - | 9.3 (0.1 to 19.3) | -0.1 (-1.5 to 1.4) |
| ≥75000 | 430 | 0.02 (0.01 to 0.05) | 0.08 (0.05 to 0.12) | 2005 | - | 19.2 (-3.0 to 46.4) | -0.7 (-3.5 to 2.2) | - | 4.2 (-1.3 to 9.9) | -0.7 (-3.5 to 2.2) |
| **Location** |  |  |  |  |  |  |  |  |  |  |
| Urban | 1,271 | 0.02 (0.01 to 0.03) | 0.09 (0.07 to 0.11) | 2002 | 2008 | 94.8* (8.4 to 250.5) | 5.1 (-1.6 to 12.2) | -1.7 (-3.4 to 0.1) | 7.9* (1.7 to 14.5) | -1.7 (-3.4 to 0.1) |
| Rural | 171 | 0.05 (0.01 to 0.12) | 0.09 (0.04 to 0.18) | - | - | 1.2 (-1.4 to 3.8) | - | - | 1.2 (-1.4 to 3.8) | 1.2 (-1.4 to 3.8) |
| **Site** |  |  |  |  |  |  |  |  |  |  |
| Head and neck | 206 | 0.00 (0.00 to 0.01) | 0.01 (0.00 to 0.02) | 2003 | - | 129.0 (-16.7 to 529.9) | -1.3 (-4.6 to 2.1) | - |  | -1.3 (-4.6 to 2.1) |
| Others | 1,243 | 0.02 (0.01 to 0.03) | 0.08 (0.06 to 0.10) | 2002 | - | 96.3* (3.0 to 273.9) | 0.3 (-1.0 to 1.5) | - |  | 0.3 (-1.0 to 1.5) |
| **Size*** |  |  |  |  |  |  |  |  |  |  |
| 0-49 mm | 176 | 0.01 (0.00 to 0.02) | 0.00 (0.00 to 0.01) | .. | .. | .. | .. | .. | .. | .. |
| ≥50 mm | 544 | 0.01 (0.01 to 0.02) | 0.02 (0.01 to 0.03) | 2008 | 2015 | 29.1 (-0.5 to 67.5) | -0.9 (-9.2 to 8.2) | -29.1*(-44.0 to -10.1) | -2.7 (-10.8 to 6.1) | -12.2* (-19.5 to -4.2) |
| **Grade** |  |  |  |  |  |  |  |  |  |  |
| I | 147 | 0.01 (0.00 to 0.02) | 0.01 (0.01 to 0.02) | - | - | -1.3 (-4.0 to 1.4) | - | - | -1.3 (-4.0 to 1.4) | -1.3 (-4.0 to 1.4) |
| II | 334 | 0.03 (0.02 to 0.04) | 0.02 (0.01 to 0.03) | - | - | -4.0* (-5.7 to -2.2) | - | - | -4.0* (-5.7 to -2.2) | -4.0* (-5.7 to -2.2) |
| III-IV | 1,010 | 0.04 (0.03 to 0.06) | 0.05 (0.04 to 0.07) | - | - | -1.6* (-2.9 to -0.4) | - | - | -1.6* (-2.9 to -0.4) | -1.6* (-2.9 to -0.4) |
| **Stage** |  |  |  |  |  |  |  |  |  |  |
| Localize | 563 | 0.00 (0.00 to 0.01) | 0.03 (0.02to 0.04) | 2007 | - | 23.7* (6.7 to 43.4) | -0.6 (-4.1 to 3.0) | - | 7.7* (2.1 to 13.7) | -0.6 (-4.1 to 3.0) |
| Regional | 431 | 0.00 (0.00 to 0.01) | 0.01 (0.01to 0.02) | 2004 | - | 39.1 (-5.8 to 105.4) | -2.5 (-5.3 to 0.3) | - | 5.0 (-2.9 to 13.6) | -2.5 (-5.3 to 0.3) |
| Distant | 316 | 0.01 (0.00 to 0.01) | 0.00 (0.00 to 0.01) | 2002 | - | 95.4 (-17.1 to 360.5) | 0.3 (-1.3 to 2.1) |  | 7.6 (-1.0 to 17.1) | 0.3 (-1.3 to 2.1) |
| Unknown | 155 | 0.00 (0.00 to 0.01) | 0.00 (0.00 to 0.01) | 2004 | 2007 | 14.0 (-7.7 to 40.7) | -29.7 (-66.4 to 47.3) | 1.8 (-2.9 to 6.7) | -1.7 (-12.4 to 10.4) | 1.8 (-2.9 to 6.7) |

Abbreviations: AAPC, average annual percentage change; NHW, non-Hispanic White; NHB, non-Hispanic Black; NHAPI, non-Hispanic Asian and Pacific Islander.

a. The analyses of race limited in non-Hispanic White, non-Hispanic Black, non-Hispanic Asian and Pacific Islander and Hispanic. Participants with non-Hispanic American Indian/Alaska Native and non-Hispanic unknown were excluded.

b. Analyses of tumor size limited from 2004 to 2019 due to the unavailability before 2004.

c. The incidence-based mortality rate was age-adjusted and adjusted for reporting delay (race and cancer type), where the 95% CI was calculated using Tiwari’s method.

d. The AAPC was generated as a weighted average of APCs during the study period. The stratified AAPC from 2008 to 2019 was calculated due to the joinpoint of 2008 in overall trend.

e. Due to the joinpoint of 2001 in overall trend, the stratified analyses were limited from 2001 to 2019.

“*” indicates the AAPC was significantly different from zero.

**Supplementary table11. Trends in incidence-based mortality rate of malignant peripheral nerve sheath tumor among different subgroups in 12 SEER registries during 2000-2019.**

| **Characteristics** | **Cases**  **(No.)** | **Incidence rate (95% CI)^b^** | | **AAPC 2000-2019**  **(95% CI)^c^** | **AAPC 2010-2019**  **(95% CI)** |
| --- | --- | --- | --- | --- | --- |
|  |  | **2000** | **2019** |  |  |
| **Overall** | 782 | 0.08 (0.05 to 0.12) | 0.10 (0.07 to 0.14) | 0.0 (-1.5 to 1.5) | 0.0 (-1.5 to 1.5) |
| **Sex** |  |  |  |  |  |
| Male | 445 | 0.10 (0.06 to 0.16) | 0.11 (0.07 to 0.16) | -0.4 (-2.1 to 1.4) | -0.4 (-2.1 to 1.4) |
| Female | 337 | 0.06 (0.03 to 0.11) | 0.10 (0.06 to 0.15) | 0.5 (-1.9 to 3.0) | 0.5 (-1.9 to 3.0) |
| **Age** |  |  |  |  |  |
| 00-19 | 40 | 0.02 (0.00 to 0.07) | 0.02 (0.00 to 0.07) | .. | .. |
| 20-39 | 185 | 0.1 (0.05 to 0.18) | 0.14 (0.08 to 0.22) | 0.5 (-2.0 to 3.1) | 0.5 (-2.0 to 3.1) |
| 40-59 | 201 | 0.06 (0.02 to 0.13) | 0.06 (0.02 to 0.13) | -1.1 (-4.2 to 2.1) | -1.1 (-4.2 to 2.1) |
| ≥60 | 356 | 0.18 (0.08 to 0.35) | 0.25 (0.15 to 0.38) | 0.1 (-1.9 to 2.1) | 0.1 (-1.9 to 2.1) |
| **Race** |  |  |  |  |  |
| NHW | 495 | 0.09 (0.05 to 0.14) | 0.08 (0.05 to 0.13) | -0.5 (-2.5 to 1.5) | -0.5 (-2.5 to 1.5) |
| NHB | 86 | 0.17 (0.04 to 0.45) | 0.23 (0.10 to 0.45) | .. | .. |
| NHAPI | 77 | 0.00 (0.00 to 0.10) | 0.10 (0.04 to 0.21) | .. | .. |
| Hispanic | 114 | 0.09 (0.03 to 0.23) | 0.08 (0.03 to 0.17) | 0.2 (-3.1 to 3.8) | 0.2 (-3.1 to 3.8) |
| **Income** |  |  |  |  |  |
| < 50000 | 38 | 0.09 (0.00 to 0.52) | 0.00 (0.00 to 0.27) | .. | .. |
| 50000-74999 | 477 | 0.21 (0.04 to 0.64) | 0.10 (0.07 to 0.15) | -0.4 (-2.3 to 1.5) | -0.4 (-2.3 to 1.5) |
| ≥75000 | 267 | 0.09 (0.05 to 0.16) | 0.09 (0.05 to 0.15) | 0.3 (-2.2 to 2.9) | 0.3 (-2.2 to 2.9) |
| **Location** |  |  |  |  |  |
| Urban | 693 | 0.08 (0.05 to 0.12) | 0.10 (0.07 to 0.14) | -0.2 (-2.0 to 1.6) | -0.2 (-2.0 to 1.6) |
| Rural | 89 | 0.14 (0.04 to 0.34) | 0.09 (0.02 to 0.27) | 0.6 (-3.3 to 4.8) | 0.6 (-3.3 to 4.8) |
| **Site** |  |  |  |  |  |
| Head and neck | 104 | 0.01 (0.00 to 0.03) | 0.02 (0.01 to 0.04) | 0.7 (-3.3 to 4.9) | 0.7 (-3.3 to 4.9) |
| Others | 682 | 0.07 (0.05 to 0.11) | 0.08 (0.06 to 0.12) | -0.2 (-1.6 to 1.3) | -0.2 (-1.6 to 1.3) |
| **Stage** |  |  |  |  |  |
| Localize | 268 | 0.01 (0.00 to 0.02) | 0.04 (0.02 to 0.06) | 3.0* (0.1 to 6.0) | 3.0* (0.1 to 6.0) |
| Regional | 219 | 0.02 (0.01 to 0.04) | 0.04 (0.02 to 0.06) | 0.3 (-2.4 to 3.1) | 0.3 (-2.4 to 3.1) |
| Distant | 173 | 0.01 (0.00 to 0.03) | 0.01 (0.00 to 0.02) | 0.2 (-2.7 to 3.1) | 0.2 (-2.7 to 3.1) |
| Unknown | 138 | 0.05 (0.03 to 0.08) | 0.01 (0.00 to 0.03) | -6.3* (-11.9 to -0.3) | 1.9 (-4.3 to 8.5) |
| Abbreviations: AAPC, average annual percentage change; NHW, non-Hispanic White; NHB, non-Hispanic Black; NHAPI, non-Hispanic Asian and Pacific Islander.  Notes:  a. The analyses of race limited in non-Hispanic White, non-Hispanic Black, non-Hispanic Asian and Pacific Islander and Hispanic. Participants with non-Hispanic American Indian/Alaska Native and non-Hispanic unknown were excluded.  b. The incidence rate was age-adjusted and adjusted for reporting delay (race and cancer type), where the 95% CI was calculated using Tiwari’s method.  c. The AAPC was generated as a weighted average of APCs during the study period. The stratified AAPC from 2008 to 2019 was calculated due to the joinpoint of 2008 in overall trend.  d. Difference in AAPC was examined using Student’s *t* test among subpopulations with significant AAPCs.  “*” indicates the AAPC was significantly different from zero. | | | | | |

**Supplementary table12.** **Age-standardized limited duration prevalence of malignant peripheral nerve sheath tumor during 2000-2019.**

| Year | Limited 20-year Prevalence ^a^  (%, 95%CI) | Estimated Count | Limited 10-year Prevalence ^a^  (%, 95%CI) | Estimated Count |
| --- | --- | --- | --- | --- |
| 2000 | 0.00129 (0.00115 to 0.00145) | 292.5 (259.9 to 328.0) |  |  |
| 2001 | 0.00129 (0.00115 to 0.00145) | 295.2 (262.5 to 330.9) |  |  |
| 2002 | 0.00138 (0.00124 to 0.00154) | 319.0 (284.9 to 356.0) |  |  |
| 2003 | 0.00138 (0.00123 to 0.00154) | 319.8 (285.7 to 356.9) |  |  |
| 2004 | 0.00134 (0.00120 to 0.00150) | 313.6 (279.8 to 350.3) |  |  |
| 2005 | 0.00135 (0.00120 to 0.00150) | 317.2 (283.2 to 354.1) |  |  |
| 2006 | 0.00140 (0.00126 to 0.00156) | 334.1 (229.2 to 371.9) |  |  |
| 2007 | 0.00149 (0.00134 to 0.00166) | 360.8 (324.5 to 400.0) |  |  |
| 2008 | 0.00159 (0.00143 to 0.00175) | 387.5 (349.9 to 428.1) |  |  |
| 2009 | 0.00152 (0.00137 to 0.00168) | 376.0 (339.0 to 416.0) |  |  |
| 2010 | 0.00151 (0.00137 to 0.00168) | 379.1 (341.9 to 419.3) | 0.00097 (0.00090 to 0.00104) | 793.2 (739.0 to 850.4) |
| 2011 | 0.00151 (0.00136 to 0.00167) | 381.2 (343.9 to 421.5) | 0.00097 (0.00091 to 0.00104) | 805.0 (750.3 to 862.6) |
| 2012 | 0.00143 (0.00129 to 0.00159) | 365.7 (329.2 to 405.2) | 0.00093 (0.00087 to 0.00100) | 775.2 (721.6 to 831.8) |
| 2013 | 0.00142 (0.00128 to 0.00158) | 367.4 (330.8 to 407.0) | 0.00091 (0.00085 to 0.00098) | 766.1 (712.8 to 822.3) |
| 2014 | 0.00137 (0.00123 to 0.00152) | 357.3 (321.2 to 396.4) | 0.00087 (0.00081 to 0.00094) | 735.3 (683.3 to 790.4) |
| 2015 | 0.00132 (0.00118 to 0.00147) | 347.1 (311.5 to 385.6) | 0.00085 (0.00079 to 0.00092) | 726.8 (674.9 to 781.6) |
| 2016 | 0.00136 (0.00123 to 0.00151) | 362.0 (325.7 to 401.3) | 0.00082 (0.00076 to 0.00088) | 699.2 (648.3 to 753.0) |
| 2017 | 0.00136 (0.00123 to 0.00151) | 365.9 (329.4 to 405.4) | 0.00079 (0.00074 to 0.00086) | 684.4 (634.1 to 737.7) |
| 2018 | 0.00134 (0.00121 to 0.00149) | 363.0 (326.6 to 402.3) | 0.00078 (0.00073 to 0.00085) | 679.2 (629.1 to 732.3) |
| 2019 | 0.00131 (0.00118 to 0.00145) | 355.3 (319.3 to 394.2) | 0.00077 (0.00071 to 0.00083) | 667.0 (617.4 to 719.6) |

Notes:

a. Limited 20-year prevalence was generated in SEER 8 from 2000 to 2019, while limited 10-year prevalence was generated in SEER 17 from 2010 to 2019.

**Supplementary table 13. Age standardized survival rate of primary malignant peripheral nerve sheath tumor among different subgroups during 2000-2019 in 17 SEER registries.**

| **Characteristics** | **No.** | **1-year (%)** | | **3-year (%)** | | **5-year (%)** | |
| --- | --- | --- | --- | --- | --- | --- | --- |
|  |  | Observed | Relative | Observed | Relative | Observed | Relative |
| **Overall** | 1973 | 74.3 | 76.1 | 55.2 | 59.3 | 47.3 | 53.3 |
| **Sex** |  |  |  |  |  |  |  |
| Male | 1,072 | 68.5 | 70.3 | 51.1 | 54.6 | 42.5 | 48.1 |
| Female | 901 | 81.1 | 82.7 | 60.5 | 64.1 | 53.3 | 58.2 |
| **Age** |  |  |  |  |  |  |  |
| 00-19 | 113 | 78.7 | 78.7 | 60.3 | 60.4 | 53.4 | 53.6 |
| 20-39 | 650 | 80.5 | 80.6 | 56.9 | 57.1 | 51.8 | 52.1 |
| 40-59 | 699 | 80.8 | 81.2 | 65.3 | 66.4 | 57.6 | 59.2 |
| ≥60 | 511 | 71.6 | 74.1 | 51.6 | 57.0 | 43.5 | 51.2 |
| **Size ^b^** |  |  |  |  |  |  |  |
| 0-49 mm | 388 | 86.4 | 87.9 | 73.6 | 79.5 | 65.3 | 70.5 |
| ≥50 mm | 864 | 68.5 | 69.9 | 43.6 | 46.0 | 34.3 | 37.5 |
| **Stage** |  |  |  |  |  |  |  |
| Localize | 1,029 | 87.9 | 89.8 | 69.9 | 74.5 | 60.2 | 67.2 |
| Regional | 486 | 70.4 | 72.0 | 45.8 | 48.9 | 37.7 | 42.3 |
| Distant | 272 | 34.3 | 34.8 | 16.9 | 17.3 | 14.7 | 15.3 |
| **Grade** |  |  |  |  |  |  |  |
| I ^c^ | 101 | 94.8 | 95.5 | 87.7 | 89.8 | 80.1 | 83.5 |
| II | 254 | 90.2 | 91.2 | 74.3 | 78.4 | 64.1 | 69.0 |
| III-IV | 292 | 69.7 | 71.2 | 41.5 | 44.0 | 35.2 | 38.0 |
| **Pathology** |  |  |  |  |  |  |  |
| Triton ^c^ | 79 | 59.2 | 59.9 | 36.3 | 38.6 | 31.3 | 32.7 |
| Others | 1894 | 74.8 | 76.7 | 55.9 | 60.1 | 47.9 | 53.9 |

Notes:

a. Analyses were limited in sex and significant factors according to above univariate and multivariate Cox regression.

b. Analyses of tumor size limited from 2004 to 2019 due to the unavailability before 2004.

c. Due to the small count, the survival rates were not adjusted to age and the crude survival rates were shown.

**Supplementary table 14. Baseline of patients at stage I-III stratified by radiotherapy before and after matching.**

|  | Before matching | | | After IPTW | | | After PSM | | |
| --- | --- | --- | --- | --- | --- | --- | --- | --- | --- |
|  | No | Yes | *P* value | No | Yes | P value | No | Yes | *P* value |
| **Patients** | 638 (100) | 571 (100) |  | 1203.5 (100) | 1217 (100) |  | 394 (100) | 394 (100) |  |
| **Diagnosis year** |  |  | 0.002 |  |  | 0.958 |  |  | 0.962 |
| 2000-2004 | 162 (25.4) | 95 (16.6) |  | 260.2 (21.6) | 257.3 (21.1) |  | 82 (20.8) | 82 (20.8) |  |
| 2005-2009 | 171 (26.8) | 155 (27.1) |  | 329.5 (27.4) | 351.6 (28.9) |  | 113 (28.7) | 109 (27.7) |  |
| 2010-2014 | 157 (24.6) | 170 (29.8) |  | 323.7 (26.9) | 326.0 (26.8) |  | 112 (28.4) | 110 (27.9) |  |
| 2015-2019 | 148 (23.2) | 151 (26.4) |  | 290.1 (24.1) | 282.2 (23.2) |  | 87 (22.1) | 93 (23.6) |  |
| **Age** |  |  | 0.55 |  |  | 0.859 |  |  | 0.926 |
| 00-19 years | 68 (10.7) | 67 (11.7) |  | 131.9 (11.0) | 122.5 (10.1) |  | 44 (11.2) | 44 (11.2) |  |
| 20-64 years | 458 (71.8) | 416 (72.9) |  | 878.9 (73.0) | 889.8 (73.1) |  | 288 (73.1) | 284 (72.1) |  |
| ≥ 65 years | 112 (17.6) | 88 (15.4) |  | 192.7 (16.0) | 204.6 (16.8) |  | 62 (15.7) | 66 (16.8) |  |
| **Sex** |  |  | 0.162 |  |  | 0.357 |  |  | >.99 |
| Male | 323 (50.6) | 313 (54.8) |  | 615.9 (51.2) | 658.9 (54.1) |  | 207 (52.5) | 207 (52.5) |  |
| Female | 315 (49.4) | 258 (45.2) |  | 587.6 (48.8) | 558.1 (45.9) |  | 187 (47.5) | 187 (47.5) |  |
| **Race** |  |  | 0.121 |  |  | 0.61 |  |  | 0.532 |
| NHW | 372 (58.3) | 308 (53.9) |  | 685.3 (56.9) | 692.9 (56.9) |  | 215 (54.6) | 219 (55.6) |  |
| NHB | 97 (15.2) | 85 (14.9) |  | 190.4 (15.8) | 167.7 (13.8) |  | 62 (15.7) | 46 (11.7) |  |
| NHAPI | 55 (8.6) | 41 (7.18) |  | 95.8 (8.0) | 83.7 (6.9) |  | 33 (8.4) | 34 (8.6) |  |
| Hispanic | 107 (16.8) | 128 (22.4) |  | 217.9 (18.1) | 249.7 (20.5) |  | 77 (19.5) | 88 (22.3) |  |
| Others | 7 (1.1) | 9 (1.6) |  | 14.1 (1.2) | 23.0 (1.9) |  | 7 (1.8) | 7 (1.8) |  |
| **Married** |  |  | >.99 |  |  | 0.435 |  |  | 0.218 |
| Yes | 375 (58.8) | 335 (58.7) |  | 728.4 (60.5) | 706.6 (58.1) |  | 244 (61.7) | 225 (57.1) |  |
| No | 263 (41.2) | 236 (41.3) |  | 475.1 (39.5) | 510.4 (41.9) |  | 151 (38.3) | 169 (42.9) |  |
| **Income** |  |  | 0.978 |  |  | 0.897 |  |  | 0.603 |
| ≤ $49999 | 82 (12.9) | 74 (13.0) |  | 158.8 (13.2) | 161.9 (13.3) |  | 50 (12.7) | 41 (10.4) |  |
| $50000-74999 | 368 (57.7) | 326 (57.1) |  | 705.6 (58.6) | 696.6 (57.2) |  | 227 (57.6) | 232 (58.9) |  |
| ≥ 75000 | 188 (29.5) | 171 (29.9) |  | 339.0 (28.2) | 358.5 (29.5) |  | 117 (29.7) | 121 (30.7) |  |
| **Location** |  |  | 0.116 |  |  | 0.144 |  |  | 0.129 |
| Urban | 561 (87.9) | 519 (90.9) |  | 1053.5 (87.5) | 1102.1 (90.6) |  | 346 (87.8) | 360 (91.4) |  |
| Rural | 77 (12.1) | 52 (9.1) |  | 150.0 (12.5) | 114.9 (9.4) |  | 48 (12.2) | 34 (8.6) |  |
| **Site** |  |  | <0.001 |  |  | 0.981 |  |  | 0.976 |
| Head and neck | 66 (10.3) | 75 (13.1) |  | 142.2 (11.8) | 145.4 (12.0) |  | 47 (11.9) | 46 (11.7) |  |
| Intracranial | 25 (3.9) | 10 (1.8) |  | 35.5 (2.9) | 37.5 (3.1) |  | 9 (2.3) | 10 (2.5) |  |
| Limb | 236 (37.0) | 261 (45.7) |  | 480.3 (39.9) | 499.0 (41.0) |  | 159 (40.4) | 164 (41.6) |  |
| Others | 311 (48.7) | 225 (39.4) |  | 545.5 (45.3) | 535.0 (44.0) |  | 179 (45.4) | 174 (44.2) |  |
| **Stage** |  |  | <0.001 |  |  | 0.964 |  |  | 0.837 |
| I | 329 (51.6) | 188 (32.9) |  | 518.5 (43.1) | 533.6 (43.8) |  | 156 (39.6) | 163 (41.4) |  |
| II | 116 (18.2) | 88 (15.4) |  | 200.8 (16.7) | 197.3 (16.2) |  | 69 (17.5) | 70 (17.8) |  |
| III | 193 (30.3) | 295 (51.7) |  | 484.2 (40.2) | 486.0 (39.9) |  | 169 (42.9) | 161 (40.9) |  |
| **Grade** |  |  | <0.001 |  |  | 0.971 |  |  | 0.771 |
| G0/GX | 329 (51.6) | 188 (32.9) |  | 518.5 (43.1) | 533.6 (43.8) |  | 156 (39.6) | 163 (41.4) |  |
| G1 | 118 (18.5) | 78 (13.7) |  | 194.8 (16.2) | 194.2 (16.0) |  | 68 (17.3) | 71 (18.0) |  |
| G2 | 191 (29.9) | 305 (53.4) |  | 490.1 (40.7) | 489.2 (40.2) |  | 170 (43.1) | 160 (40.6) |  |
| **Size** |  |  | <0.001 |  |  | 0.998 |  |  | 0.915 |
| 0-50mm | 66 (10.3) | 75 (13.1) |  | 452.4 (37.6) | 460.9 (37.9) |  | 129 (32.7) | 128 (32.5) |  |
| 51-100mm | 25 (3.9) | 10 (1.8) |  | 447.4 (37.2) | 455.0 (37.4) |  | 169 (42.9) | 164 (41.6) |  |
| 101-150mm | 236 (37.0) | 261 (45.7) |  | 185.5 (15.4) | 185.3 (15.2) |  | 54 (13.7) | 61 (15.5) |  |
| > 150mm | 311 (48.7) | 225 (39.4) |  | 118.2 (9.8) | 115.8 (9.5) |  | 42 (10.7) | 41 (10.4) |  |
| **Surgery** |  |  | <0.001 |  |  | 0.946 |  |  | 0.98 |
| Local excision | 335 (52.5) | 255 (44.7) |  | 580.1 (48.2) | 583.4 (47.9) |  | 200 (50.8) | 200 (50.8) |  |
| Radical excision | 206 (32.3) | 274 (48.0) |  | 479.6 (39.9) | 479.7 (39.4) |  | 154 (39.1) | 157 (39.8) |  |
| Amputation of limb | 75 (11.8) | 31 (5.4) |  | 111.7 (9.3) | 125.5 (10.3) |  | 30 (7.6) | 27 (6.9) |  |
| Others | 22 (3.5) | 11 (1.9) |  | 32.0 (2.7) | 28.5 (2.3) |  | 10 (2.5) | 10 (2.5) |  |
| **Chemotherapy** |  |  | <0.001 |  |  | 0.898 |  |  | 0.924 |
| No | 560 (87.8) | 420 (73.6) |  | 977.2 (81.2) | 984.2 (80.9) |  | 328 (83.2) | 326 (82.7) |  |
| Yes | 78 (12.2) | 151 (26.4) |  | 226.3 (18.8) | 232.8 (19.1) |  | 66 (16.8) | 68 (17.3) |  |

**Supplementary table 15. Multivariable analysis for radiotherapy utilization among patients at stage I-III.**

|  | Uni-regression | | Multi-regression | | Adjustment | |
| --- | --- | --- | --- | --- | --- | --- |
|  | OR | *P* value | OR | *P* value | OR | *P* value |
| **Diagnosis year** |  |  |  |  |  |  |
| 2000-2004 | Reference | - | Reference | - | Reference | - |
| 2005-2009 | 1.55 (1.11-2.16) | 0.011 | 1.44 (1.00-2.08) | 0.049 | 1.45 (1.01-2.10) | 0.045 |
| 2010-2014 | 1.85 (1.32-2.58) | < .001 | 1.60 (1.12-2.31) | 0.011 | 1.61 (1.12-2.31) | 0.011 |
| 2015-2019 | 1.74 (1.24-2.44) | 0.001 | 1.45 (1.00-2.0) | 0.052 | 1.42 (0.98-2.06) | 0.062 |
| **Age** |  |  |  |  |  |  |
| 00-19 years | Reference | - |  |  |  |  |
| 20-64 years | 0.92 (0.64-1.32) | 0.66 |  |  |  |  |
| ≥ 65 years | 0.8 (0.51-1.24) | 0.311 |  |  |  |  |
| **Sex** |  |  |  |  |  |  |
| Male | Reference | - |  |  |  |  |
| Female | 0.85 (0.67-1.06) | 0.145 |  |  |  |  |
| **Race** |  |  |  |  |  |  |
| NHW | Reference | - | Reference | - |  |  |
| NHB | 1.06 (0.76-1.47) | 0.735 | 0.87 (0.61-1.25) | 0.44 |  |  |
| NHAPI | 0.9 (0.58-1.39) | 0.634 | 0.90 (0.56-1.45) | 0.672 |  |  |
| Hispanic | 1.44 (1.07-1.95) | 0.015 | 1.13 (0.81-1.57) | 0.46 |  |  |
| Others | 1.55 (0.57-4.22) | 0.388 | 1.46 (0.50-4.32) | 0.489 |  |  |
| **Married** |  |  |  |  |  |  |
| Yes | Reference | - |  |  |  |  |
| No | 1 (0.8-1.26) | 0.97 |  |  |  |  |
| **Income** |  |  |  |  |  |  |
| ≤ $49999 | Reference | - |  |  |  |  |
| $50000-74999 | 0.98 (0.69-1.39) | 0.917 |  |  |  |  |
| ≥ 75000 | 1.01 (0.69-1.47) | 0.967 |  |  |  |  |
| **Location** |  |  |  |  |  |  |
| Urban | Reference | - |  |  | Reference | - |
| Rural | 0.73 (0.5-1.06) | 0.097 |  |  | 0.74 (0.49-1.11) | 0.14 |
| **Site** |  |  |  |  |  |  |
| Head and neck | Reference | - | Reference | - | Reference | - |
| Intracranial | 0.35 (0.16-0.79) | 0.011 | 0.62 (0.26-1.50) | 0.293 | 0.62 (0.26-1.50) | 0.294 |
| Limb | 0.97 (0.67-1.42) | 0.887 | 0.65 (0.42-0.99) | 0.043 | 0.64 (0.42-0.98) | 0.038 |
| Others | 0.64 (0.44-0.92) | 0.018 | 0.42 (0.27-0.63) | < .001 | 0.42 (0.27-0.64) | < .001 |
| **Stage** |  |  |  |  |  |  |
| I | Reference | - | Reference | - | Reference | - |
| II | 1.33 (0.95-1.85) | 0.092 | 2.85 (1.83-4.45) | < .001 | 2.87 (1.84-4.47) | < .001 |
| III | 2.67 (2.07-3.45) | < .001 | 1.70 (1.22-2.37) | 0.002 | 1.72 (1.23-2.40) | 0.003 |
| **Grade** |  |  |  |  |  |  |
| G0/GX | Reference | - | Reference | - | Reference | - |
| G1 | 1.16 (0.83-1.62) | 0.398 | 0.51 (0.35-0.73) | <.001 | 0.5 (0.35-0.72) | < .001 |
| G2 | 2.79 (2.17-3.6) | < .001 | - | - | - | - |
| **Size** |  |  |  |  |  |  |
| 0-50mm | Reference | - | Reference | - | Reference | - |
| 51-100mm | 2.9 (2.22-3.81) | < .001 | 3.32 (2.19-5.04) | < .001 | 3.33 (2.20-5.05) | < .001 |
| 101-150mm | 3.22 (2.26-4.6) | < .001 | 3.51 (2.12-5.81) | < .001 | 3.42 (2.07-5.65) | < .001 |
| > 150mm | 1.64 (1.08-2.47) | 0.019 | 1.76 (1.01-3.06) | 0.042 | 1.76 (1.02-3.07) | 0.044 |
| **Surgery** |  |  |  |  |  |  |
| Local excision | Reference | - | Reference | - | Reference | - |
| Radical excision | 1.75 (1.37-2.23) | < .001 | 1.45 (1.11-1.90) | <.001 | 1.44 (1.11-1.88) | 0.007 |
| Amputation of limb | 0.54 (0.35-0.85) | 0.008 | 0.45 (0.27-0.75) | 0.002 | 0.45 (0.27-0.75) | 0.002 |
| Others | 0.66 (0.31-1.38) | 0.267 | 0.59 (0.27-1.32) | 0.199 | 0.59 (0.27-1.30) | 0.194 |
| **Chemotherapy** |  |  |  |  |  |  |
| No | Reference | - | Reference | - | Reference | - |
| Yes | 2.58 (1.91-3.49) | < .001 | 2.01 (1.44-2.84) | <.001 | 2.05 (1.46-2.88) | < .001 |

**Supplementary table 16. Cox regression of overall survival for patients at stage I-III stratified by radiotherapy.**

|  | Before matching | | | | After IPTW | | | | After PSM | | | |
| --- | --- | --- | --- | --- | --- | --- | --- | --- | --- | --- | --- | --- |
|  | Uni-regression | | Multi-regression | | Uni-regression | | Multi-regression | | Uni-regression | | Multi-regression | |
|  | HR | *P* value | HR | *P* value | HR | *P* value | HR | *P* value | HR | *P* value | HR | *P* value |
| **Diagnosis year** |  |  |  |  |  |  |  |  |  |  |  |  |
| 2000-2004 | Reference | - | Reference | - | Reference | - | Reference | - | Reference | - | Reference | - |
| 2005-2009 | 1.02 (0.77-1.36) | 0.893 | 1.05 (0.82-1.33) | 0.712 | 0.99 (0.76-1.3) | 0.956 | 0.99 (0.76-1.31) | 0.966 |  |  | 1.14 (0.84-1.53) | 0.402 |
| 2010-2014 | 1.22 (0.91-1.63) | 0.189 | 1.17 (0.91-1.5) | 0.218 | 1.23 (0.95-1.6) | 0.12 | 1.12 (0.85-1.48) | 0.41 |  |  | 1.23 (0.91-1.67) | 0.178 |
| 2015-2019 | 1 (0.69-1.44) | 0.992 | 0.95 (0.7-1.28) | 0.733 | 1.04 (0.75-1.45) | 0.818 | 0.92 (0.66-1.28) | 0.609 |  |  | 0.91 (0.62-1.32) | 0.601 |
| **Age** |  |  |  |  |  |  |  |  |  |  |  |  |
| 00-19 years | Reference | - | Reference | - | Reference | - | Reference | - | Reference | - | Reference | - |
| 20-64 years | 1.21 (0.89-1.65) | 0.225 | 1.58 (1.14-2.2) | 0.006 | 1.35 (0.97-1.88) | 0.073 | 1.87 (1.31-2.67) | 0.001 | 1.28 (0.87-1.88) | 0.214 | 1.97 (1.3-2.99) | 0.001 |
| ≥ 65 years | 2.13 (1.51-2.99) | < .001 | 3.45 (2.37-5.01) | < .001 | 2.21 (1.55-3.15) | 0 | 3.82 (2.54-5.73) | < .001 | 2.14 (1.4-3.28) | 0 | 4.12 (2.56-6.62) | < .001 |
| **Sex** |  |  |  |  |  |  |  |  |  |  |  |  |
| Male | Reference | - | Reference | - | Reference | - |  |  | Reference | - | Reference | - |
| Female | 0.82 (0.68-0.97) | 0.023 | 0.89 (0.74-1.06) | 0.201 | 0.77 (0.63-0.94) | 0.01 | 0.82 (0.67-1.01) | 0.063 | 0.76 (0.61-0.94) | 0.012 | 0.8 (0.64-0.99) | 0.041 |
| **Race** |  |  |  |  |  |  |  |  |  |  |  |  |
| NHW | Reference | - |  |  | Reference | - |  |  | Reference | - |  |  |
| NHB | 1.12 (0.87-1.44) | 0.374 |  |  | 1.09 (0.82-1.44) | 0.569 |  |  | 1.29 (0.95-1.76) | 0.1 |  |  |
| NHAPI | 0.93 (0.66-1.32) | 0.697 |  |  | 1.12 (0.77-1.61) | 0.562 |  |  | 1.05 (0.71-1.56) | 0.813 |  |  |
| Hispanic | 1.18 (0.94-1.49) | 0.146 |  |  | 1.21 (0.94-1.56) | 0.146 |  |  | 1.22 (0.94-1.6) | 0.138 |  |  |
| Others | 1.17 (0.55-2.47) | 0.685 |  |  | 1.05 (0.49-2.26) | 0.902 |  |  | 0.98 (0.4-2.38) | 0.959 |  |  |
| **Married** |  |  |  |  |  |  |  |  |  |  |  |  |
| Yes | Reference | - | Reference | - | Reference | - | Reference | - | Reference | - | Reference | - |
| No | 0.78 (0.65-0.93) | 0.006 | 0.71 (0.59-0.86) | 0.001 | 0.79 (0.64-0.96) | 0.017 | 0.71 (0.57-0.87) | 0.001 | 0.79 (0.64-0.99) | 0.037 | 0.7 (0.55-0.88) | 0.002 |
| **Income** |  |  |  |  |  |  |  |  |  |  |  |  |
| ≤ $49999 | Reference | - |  |  | Reference | - |  |  | Reference | - |  |  |
| $50000-74999 | 0.91 (0.7-1.18) | 0.479 |  |  | 0.94 (0.69-1.29) | 0.712 |  |  | 1.17 (0.82-1.65) | 0.385 |  |  |
| ≥ 75000 | 0.78 (0.58-1.04) | 0.089 |  |  | 0.75 (0.53-1.06) | 0.107 |  |  | 0.9 (0.61-1.33) | 0.603 |  |  |
| **Location** |  |  |  |  |  |  |  |  |  |  |  |  |
| Urban | Reference | - |  |  | Reference | - |  |  | Reference | - |  |  |
| Rural | 1.03 (0.78-1.36) | 0.822 |  |  | 0.95 (0.68-1.32) | 0.759 |  |  | 0.81 (0.56-1.18) | 0.274 |  |  |
| **Site** |  |  |  |  |  |  |  |  |  |  |  |  |
| Head and neck | Reference | - | Reference | - | Reference | - | Reference | - | Reference | - | Reference | - |
| Intracranial | 0.73 (0.41-1.31) | 0.29 | 1.08 (0.6-1.95) | 0.801 | 1.06 (0.58-1.93) | 0.859 | 1.46 (0.8-2.65) | 0.217 | 0.83 (0.4-1.73) | 0.625 | 1.12 (0.54-2.35) | 0.758 |
| Limb | 0.66 (0.49-0.87) | 0.004 | 0.44 (0.33-0.6) | 0 | 0.64 (0.47-0.88) | 0.006 | 0.46 (0.33-0.64) | 0 | 0.68 (0.47-0.98) | 0.04 | 0.47 (0.32-0.7) | < .001 |
| Others | 1.03 (0.78-1.36) | 0.829 | 0.71 (0.53-0.95) | 0.02 | 1.04 (0.77-1.41) | 0.778 | 0.76 (0.55-1.05) | 0.098 | 1.22 (0.86-1.72) | 0.274 | 0.83 (0.57-1.21) | 0.341 |
| **Stage** |  |  |  |  |  |  |  |  |  |  |  |  |
| I | Reference | - | Reference | - | Reference | - | Reference | - | Reference | - | Reference | - |
| II | 0.77 (0.58-1.03) | 0.077 | 1.93 (1.34-2.79) | < .001 | 0.7 (0.52-0.95) | 0.022 | 1.58 (1.06-2.37) | 0.026 | 0.75 (0.53-1.05) | 0.095 | 1.9 (1.19-3.03) | 0.007 |
| III | 1.66 (1.37-2) | < .001 | 1.45 (1.15-1.83) | 0.002 | 1.53 (1.23-1.89) | 0 | 1.43 (1.1-1.85) | 0.008 | 1.41 (1.12-1.77) | 0.003 | 1.45 (1.1-1.91) | 0.008 |
| **Grade** |  |  |  |  |  |  |  |  |  |  |  |  |
| G0/GX | Reference | - | Reference | - | Reference | - | Reference | - | Reference | - | Reference | - |
| G1 | 0.71 (0.53-0.95) | 0.023 | 0.45 (0.33-0.6) | < .001 | 0.69 (0.5-0.94) | 0.02 | 0.48 (0.35-0.66) | < .001 | 0.7 (0.5-0.98) | 0.038 | 0.49 (0.35-0.68) | < .001 |
| G2 | 1.69 (1.4-2.04) | < .001 | - | - | 1.54 (1.25-1.9) | 0 |  |  | 1.47 (1.16-1.84) | 0.001 |  |  |
| **Size** |  |  |  |  |  |  |  |  |  |  |  |  |
| 0-50mm | Reference | - | Reference | - | Reference | - | Reference | - | Reference | - | Reference | - |
| 51-100mm | 1.93 (1.55-2.41) | < .001 | 2.18 (1.58-3.01) | < .001 | 1.75 (1.37-2.23) | 0 | 1.8 (1.25-2.59) | 0.002 | 1.59 (1.22-2.09) | 0.001 | 1.88 (1.25-2.84) | 0.003 |
| 101-150mm | 2.65 (2.04-3.45) | < .001 | 2.73 (1.89-3.95) | < .001 | 2.33 (1.72-3.15) | 0 | 2.23 (1.47-3.39) | < .001 | 2.23 (1.6-3.1) | 0 | 2.41 (1.52-3.84) | < .001 |
| > 150mm | 3.35 (2.51-4.47) | < .001 | 3.9 (2.67-5.7) | < .001 | 2.95 (2.11-4.11) | 0 | 3.14 (2.02-4.88) | < .001 | 2.99 (2.1-4.27) | 0 | 3.5 (2.15-5.7) | < .001 |
| **Surgery** |  |  |  |  |  |  |  |  |  |  |  |  |
| Local excision | Reference | - |  |  | Reference | - |  |  | Reference | - |  |  |
| Radical excision | 1.1 (0.92-1.33) | 0.299 |  |  | 1.1 (0.9-1.35) | 0.359 |  |  | 1.02 (0.81-1.27) | 0.879 |  |  |
| Amputation of limb | 1.05 (0.77-1.44) | 0.749 |  |  | 1.06 (0.72-1.55) | 0.782 |  |  | 1.04 (0.7-1.55) | 0.847 |  |  |
| Others | 1.16 (0.7-1.93) | 0.558 |  |  | 1.15 (0.64-2.05) | 0.643 |  |  | 1.25 (0.66-2.37) | 0.487 |  |  |
| **Chemotherapy** |  |  |  |  |  |  |  |  |  |  |  |  |
| No | Reference | - | Reference | - | Reference | - | Reference | - | Reference | - | Reference | - |
| Yes | 1.7 (1.39-2.08) | <.001 | 1.45 (1.15-1.81) | 0.001 | 1.65 (1.31-2.08) | 0 | 1.52 (1.17-1.97) | 0.002 | 1.6 (1.24-2.07) | 0 | 1.6 (1.21-2.13) | 0.001 |
| **Radiotherapy** |  |  |  |  |  |  |  |  |  |  |  |  |
| No | Reference | - | Reference | - | Reference | - | Reference | - | Reference | - | Reference | - |
| Yes | 1.35 (1.14-1.61) | < .001 | 0.99 (0.82-1.19) | 0.881 | 1.09 (0.9-1.33) | 0.384 | 1.01 (0.83-1.23) | 0.914 | 1.04 (0.84-1.29) | 0.701 | 0.99 (0.8-1.22) | 0.93 |

**Supplementary table 17. Restricted mean survival time for patients at stage I-III stratified by radiotherapy.**

|  | **Unadjusted** | | **PSM** | |
| --- | --- | --- | --- | --- |
|  | **No** | **Yes** | **No** | **Yes** |
| **Restricted at 1 year** | | | | |
| RSMT, month | 11.2 (11.0-11.4) | 11.7 (11.5-11.8) | 11.1 (10.9-11.3) | 11.6 (11.5-11.8) |
| RMST difference | 0.4 (0.2-0.7) (*P* <.001) | | 0.6 (0.3-0.8) (*P* <.001) | |
| **Restricted at 3 years** | | | | |
| RSMT, month | 30.2 (29.3-31.0) | 30.2 (29.4-31.0) | 29.0 (27.9-30.2) | 30.5 (29.5-31.5) |
| RMST difference | 0.0 (-1.2-1.2) (*P* = 0.982) | | 1.5 (-0.0 -2.6) (*P* = 0.058) | |
| **Restricted at 5 years** | | | | |
| RSMT, month | 46.7 (45.1-48.4) | 44.6 (42.9-46.3) | 44.3 (42.1-46.5) | 45.4 (43.3-47.4) |
| RMST difference | -2.2 (-4.5-0.2) (P = 0.074) | | 1.1 (-1.9-4.0) (*P*= 0.485) | |
| **Restricted at 10 years** | | | | |
| RSMT, month | 84.3 (80.4-88.1) | 74.8 (70.6-78.9) | 77.7 (72.6-82.7) | 77.4 (72.6-82.3) |
| RMST difference | -9.5 (-15.1-3.9) (*P* = 0.001) | | -0.3 (-7.3-6.7) (*P* = 0.934) | |

**Supplementary table 18. Subgroup analysis for patients at stage I-III with radiotherapy.**

|  | Before matching | | After IPTW | | After PSM | |
| --- | --- | --- | --- | --- | --- | --- |
|  | Hazard ratio | *P* value | Hazard ratio | *P* value | Hazard ratio | *P* value |
| **Diagnosis year** |  | 0.981 |  | 0.875 |  | 0.403 |
| 2000-2004 | 1.03 (0.72-1.47) | 0.869 | 0.87 (0.59-1.27) | 0.468 | 0.66 (0.43-1) | 0.051 |
| 2005-2009 | 1.88 (1.36-2.61) | < .001 | 1.43 (0.99-2.08) | 0.057 | 1.64 (1.11-2.43) | 0.013 |
| 2010-2014 | 1.22 (0.89-1.68) | 0.226 | 1.05 (0.74-1.47) | 0.798 | 0.97 (0.66-1.42) | 0.874 |
| 2015-2019 | 1.19 (0.75-1.91) | 0.462 | 0.98 (0.59-1.64) | 0.938 | 1.02 (0.57-1.83) | 0.943 |
| **Age** |  | 0.101 |  | 0.242 |  | 0.262 |
| 00-19 years | 1.64 (0.9-2.96) | 0.104 | 1.34 (0.71-2.51) | 0.364 | 1.29 (0.62-2.69) | 0.493 |
| 20-64 years | 1.43 (1.15-1.77) | 0.001 | 1.11 (0.87-1.4) | 0.414 | 1.07 (0.83-1.38) | 0.621 |
| ≥ 65 years | 1.08 (0.75-1.55) | 0.675 | 0.91 (0.61-1.34) | 0.62 | 0.82 (0.53-1.29) | 0.395 |
| **Sex** |  | 0.239 |  | 0.031 |  | 0.007 |
| Male | 1.48 (1.17-1.87) | 0.001 | 1.31 (1.01-1.7) | 0.039 | 1.35 (1.02-1.79) | 0.035 |
| Female | 1.2 (0.92-1.56) | 0.186 | 0.85 (0.63-1.15) | 0.288 | 0.75 (0.54-1.04) | 0.081 |
| **Race** |  | 0.554 |  | 0.363 |  | 0.438 |
| NHW | 1.57 (1.18-2.11) | 0.002 | 1.63 (1.04-2.54) | 0.032 | 1.41 (0.93-2.12) | 0.102 |
| NHB | 1.6 (0.94-2.7) | 0.083 | 1.21 (0.6-2.44) | 0.602 | 0.98 (0.52-1.86) | 0.952 |
| NHAPI | 2.18 (1.02-4.65) | 0.045 | 2.88 (1.51-5.49) | 0.001 | 2.19 (0.67-7.2) | 0.196 |
| Hispanic | 1.91 (1.28-2.86) | 0.002 | 1.13 (0.58-2.19) | 0.713 | 1.07 (0.65-1.75) | 0.794 |
| Others | 0.58 (0.07-4.85) | 0.616 | 0.3 (0.03-2.62) | 0.274 | 0.71 (0.04-11.79) | 0.809 |
| **Married** |  | 0.011 |  | 0.063 | 0 (0-0) | 0.403 |
| Yes | 1.12 (0.9-1.4) | 0.322 | 0.94 (0.74-1.21) | 0.647 | 0.92 (0.7-1.21) | 0.551 |
| No | 1.82 (1.37-2.42) | < .001 | 1.39 (1.01-1.9) | 0.042 | 1.29 (0.91-1.83) | 0.149 |
| **Income** |  | 0.995 |  | 0.476 |  | 0.501 |
| ≤ $49999 | 1.48 (0.92-2.36) | 0.105 | 1.23 (0.72-2.1) | 0.455 | 1.32 (0.69-2.51) | 0.4 |
| $50000-74999 | 1.26 (1.01-1.58) | 0.044 | 1.09 (0.85-1.39) | 0.486 | 1 (0.77-1.3) | 0.992 |
| ≥ 75000 | 1.47 (1.04-2.07) | 0.029 | 0.98 (0.66-1.46) | 0.92 | 0.99 (0.65-1.52) | 0.972 |
| **Location** |  | 0.769 |  | 0.9 |  | 0.915 |
| Urban | 1.37 (1.14-1.65) | 0.001 | 1.1 (0.89-1.35) | 0.387 | 1.04 (0.83-1.3) | 0.719 |
| Rural | 1.22 (0.72-2.06) | 0.454 | 1.02 (0.56-1.87) | 0.949 | 0.97 (0.48-1.97) | 0.933 |
| **Site** |  | 0.017 |  | 0.171 |  | 0.785 |
| Head and neck | 2.31 (1.37-3.88) | 0.002 | 1.6 (0.91-2.82) | 0.102 | 1.04 (0.55-1.96) | 0.906 |
| Intracranial | 1.73 (0.56-5.34) | 0.339 | 1.63 (0.64-4.14) | 0.302 | 2.18 (0.52-9.14) | 0.288 |
| Limb | 1.37 (1.01-1.86) | 0.041 | 1 (0.72-1.4) | 0.982 | 1.09 (0.76-1.56) | 0.652 |
| Others | 1.26 (0.99-1.62) | 0.063 | 1.03 (0.78-1.36) | 0.821 | 1.01 (0.75-1.35) | 0.95 |
| **Stage** |  | 0.003 |  | 0.006 | 0 (0-0) | 0.014 |
| I | 1.61 (1.21-2.15) | 0.001 | 1.39 (1.01-1.92) | 0.041 | 1.35 (0.95-1.93) | 0.093 |
| II | 1.88 (1.15-3.08) | 0.013 | 1.6 (0.95-2.7) | 0.077 | 1.41 (0.77-2.57) | 0.264 |
| III | 0.84 (0.66-1.08) | 0.18 | 0.78 (0.6-1.02) | 0.07 | 0.78 (0.58-1.06) | 0.109 |
| **Grade** |  | 0.007 | 0 (0-0) | 0.015 |  | 0.025 |
| G0/GX | 1.61 (1.21-2.15) | 0.001 | 1.39 (1.01-1.92) | 0.041 | 1.35 (0.95-1.93) | 0.093 |
| G1 | 1.3 (0.77-2.18) | 0.322 | 1.18 (0.68-2.05) | 0.553 | 1.12 (0.62-2) | 0.716 |
| G2 | 0.87 (0.68-1.11) | 0.266 | 0.83 (0.64-1.08) | 0.17 | 0.82 (0.61-1.1) | 0.189 |
| **Size** |  | < .001 |  | 0.001 |  | 0.016 |
| 0-50mm | 2.01 (1.43-2.84) | < .001 | 1.87 (1.28-2.72) | 0.001 | 1.7 (1.08-2.66) | 0.021 |
| 51-100mm | 1.06 (0.81-1.41) | 0.664 | 0.97 (0.72-1.31) | 0.856 | 0.95 (0.69-1.3) | 0.734 |
| 101-150mm | 0.8 (0.53-1.2) | 0.274 | 0.74 (0.48-1.15) | 0.179 | 0.78 (0.48-1.28) | 0.33 |
| > 150mm | 0.79 (0.49-1.27) | 0.334 | 0.71 (0.44-1.16) | 0.172 | 0.77 (0.44-1.34) | 0.355 |
| **Surgery** |  | 0.309 |  | 0.372 |  | 0.211 |
| Local excision | 1.57 (1.22-2.02) | 0.001 | 1.26 (0.96-1.67) | 0.098 | 1.23 (0.91-1.66) | 0.189 |
| Radical excision | 1.12 (0.85-1.48) | 0.427 | 0.91 (0.68-1.23) | 0.551 | 0.87 (0.62-1.22) | 0.428 |
| Amputation of limb | 1.33 (0.71-2.46) | 0.37 | 1.08 (0.55-2.13) | 0.824 | 0.95 (0.45-2.01) | 0.889 |
| Others | 1.33 (0.48-3.67) | 0.583 | 1 (0.35-2.91) | 0.995 | 0.79 (0.23-2.76) | 0.714 |
| **Chemotherapy** |  | 0.01 |  | 0.144 |  | 0.718 |
| No | 1.41 (1.15-1.72) | 0.001 | 1.17 (0.94-1.46) | 0.167 | 1.05 (0.83-1.33) | 0.693 |
| Yes | 0.8 (0.56-1.16) | 0.235 | 0.82 (0.55-1.23) | 0.333 | 0.96 (0.61-1.5) | 0.85 |

**Supplementary table 19. Baseline of patients at stage I-III stratified by chemotherapy before and after matching.**

|  | Before matching | | | After IPTW | | | After PSM | | |
| --- | --- | --- | --- | --- | --- | --- | --- | --- | --- |
|  | No | Yes | *P* value | No | Yes | P value | No | Yes | *P* value |
| **Patients** | 980 (100) | 229 (100) |  | 1210 (100) | 1170 (100) |  | 213 (100) | 213 (100) |  |
| **Diagnosis year** |  |  | 0.282 |  |  | 0.063 |  |  | 0.208 |
| 2000-2004 | 211 (21.5) | 46 (20.1) |  | 252.1 (20.8) | 369.9 (31.6) |  | 36 (16.9) | 44 (20.7) |  |
| 2005-2009 | 258 (26.3) | 68 (29.7) |  | 310.4 (25.6) | 267.0 (22.8) |  | 56 (26.3) | 64 (30.0) |  |
| 2010-2014 | 275 (28.1) | 52 (22.7) |  | 353.7 (29.2) | 237.6 (20.3) |  | 67 (31.5) | 48 (22.5) |  |
| 2015-2019 | 236 (24.1) | 63 (27.5) |  | 293.9 (24.3) | 295.5 (25.3) |  | 54 (25.4) | 57 (26.8) |  |
| **Age** |  |  | <0.001 |  |  | 0.445 |  |  | 0.977 |
| 00-19 years | 79 (8.06) | 56 (24.5) |  | 131.0 (10.8) | 114.0 (9.7) |  | 44 (20.7) | 44 (20.7) |  |
| 20-64 years | 713 (72.8) | 161 (70.3) |  | 879.4 (72.7) | 911.2 (77.9) |  | 158 (74.2) | 157 (73.7) |  |
| ≥ 65 years | 188 (19.2) | 12 (5.24) |  | 199.7 (16.5) | 144.8 (12.4) |  | 11 (5.2) | 12 (5.6) |  |
| **Sex** |  |  | 0.012 |  |  | 0.37 |  |  | 0.921 |
| Male | 498 (50.8) | 138 (60.3) |  | 634.9 (52.5) | 558.2 (47.7) |  | 125 (58.7) | 127 (59.6) |  |
| Female | 482 (49.2) | 91 (39.7) |  | 575.1 (47.5) | 611.8 (52.3) |  | 88 (41.3) | 86 (40.4) |  |
| **Race** |  |  | <0.001 |  |  | 0.836 |  |  | 0.955 |
| NHW | 570 (58.2) | 110 (48.0) |  | 680.7 (56.3) | 615.0 (52.6) |  | 100 (46.9) | 102 (47.9) |  |
| NHB | 149 (15.2) | 33 (14.4) |  | 184.8 (15.3) | 191.8 (16.4) |  | 35 (16.4) | 33 (15.5) |  |
| NHAPI | 82 (8.37) | 14 (6.11) |  | 94.8 (7.8) | 87.0 (7.4) |  | 11 (5.2) | 14 (6.6) |  |
| Hispanic | 166 (16.9) | 69 (30.1) |  | 234.2 (19.4) | 267.7 (22.9) |  | 64 (30.0) | 62 (29.1) |  |
| Others | 13 (1.33) | 3 (1.31) |  | 15.5 (1.3) | 8.4 (0.7) |  | 3 (1.4) | 2 (0.9) |  |
| **Married** |  |  | 0.001 |  |  | 0.235 |  |  | 0.611 |
| Yes | 553 (56.4) | 157 (68.6) |  | 705.7 (58.3) | 754.3 (64.5) |  | 136 (63.8) | 142 (66.7) |  |
| No | 427 (43.6) | 72 (31.4) |  | 504.3 (41.7) | 415.7 (35.5) |  | 77 (36.2) | 71 (33.3) |  |
| **Income** |  |  | 0.254 |  |  | 0.653 |  |  | 0.901 |
| ≤ $49999 | 120 (12.2) | 36 (15.7) |  | 158.7 (13.1) | 191.7 (16.4) |  | 36 (16.9) | 33 (15.5) |  |
| $50000-74999 | 572 (58.4) | 122 (53.3) |  | 692.7 (57.2) | 624.0 (53.3) |  | 111 (52.1) | 115 (54.0) |  |
| ≥ 75000 | 288 (29.4) | 71 (31.0) |  | 358.6 (29.6) | 354.2 (30.3) |  | 66 (31.0) | 65 (30.5) |  |
| **Location** |  |  | > .99 |  |  | 0.846 |  |  | 0.757 |
| Urban | 875 (89.3) | 205 (89.5) |  | 1074.7 (88.8) | 1031.1 (88.1) |  | 188 (88.3) | 191 (89.7) |  |
| Rural | 105 (10.7) | 24 (10.5) |  | 135.3 (11.2) | 138.9 (11.9) |  | 25 (11.7) | 22 (10.3) |  |
| **Site** |  |  | 0.002 |  |  | 0.15 |  |  | 0.435 |
| Head and neck | 126 (12.9) | 15 (6.55) |  | 144.9 (12.0) | 82.3 (7.0) |  | 16 (7.5) | 14 (6.6) |  |
| Intracranial | 34 (3.47) | 1 (0.44) |  | 37.8 (3.1) | 7.8 (0.7) |  | 4 (1.9) | 1 (0.5) |  |
| Limb | 398 (40.6) | 99 (43.2) |  | 503.6 (41.6) | 542.6 (46.4) |  | 99 (46.5) | 93 (43.7) |  |
| Others | 422 (43.1) | 114 (49.8) |  | 523.8 (43.3) | 537.2 (45.9) |  | 94 (44.1) | 105 (49.3) |  |
| **Stage** |  |  | <0.001 |  |  | 0.602 |  |  | 0.467 |
| I | 454 (46.3) | 63 (27.5) |  | 509.1 (42.1) | 435.6 (37.2) |  | 51 (23.9) | 61 (28.6) |  |
| II | 186 (19.0) | 18 (7.86) |  | 200.6 (16.6) | 233.4 (20.0) |  | 16 (7.5) | 18 (8.5) |  |
| III | 340 (34.7) | 148 (64.6) |  | 500.2 (41.3) | 500.9 (42.8) |  | 146 (68.5) | 134 (62.9) |  |
| **Grade** |  |  | <0.001 |  |  | 0.642 |  |  | 0.503 |
| G0/GX | 454 (46.3) | 63 (27.5) |  | 509.1 (42.1) | 435.6 (37.2) |  | 51 (23.9) | 61 (28.6) |  |
| G1 | 174 (17.8) | 22 (9.61) |  | 197.1 (16.3) | 221.1 (18.9) |  | 25 (11.7) | 21 (9.9) |  |
| G2 | 352 (35.9) | 144 (62.9) |  | 503.8 (41.6) | 513.3 (43.9) |  | 137 (64.3) | 131 (61.5) |  |
| **Size** |  |  | <0.001 |  |  | 0.808 |  |  | 0.52 |
| 0-50mm | 428 (43.7) | 26 (11.4) |  | 453.7 (37.5) | 393.6 (33.6) |  | 27 (12.7) | 26 (12.2) |  |
| 51-100mm | 348 (35.5) | 106 (46.3) |  | 452.6 (37.4) | 472.0 (40.3) |  | 112 (52.6) | 99 (46.5) |  |
| 101-150mm | 129 (13.2) | 53 (23.1) |  | 181.9 (15.0) | 188.2 (16.1) |  | 44 (20.7) | 49 (23.0) |  |
| > 150mm | 75 (7.65) | 44 (19.2) |  | 121.8 (10.1) | 116.1 (9.9) |  | 30 (14.1) | 39 (18.3) |  |
| **Surgery** |  |  | <0.001 |  |  | 0.771 |  |  | 0.937 |
| Local excision | 507 (51.7) | 83 (36.2) |  | 587.2 (48.5) | 570.7 (48.8) |  | 85 (39.9) | 82 (38.5) |  |
| Radical excision | 372 (38.0) | 108 (47.2) |  | 486.1 (40.2) | 490.7 (41.9) |  | 94 (44.1) | 100 (46.9) |  |
| Amputation of limb | 77 (7.86) | 29 (12.7) |  | 102.8 (8.5) | 73.5 (6.3) |  | 27 (12.7) | 24 (11.3) |  |
| Others | 24 (2.45) | 9 (3.93) |  | 33.9 (2.8) | 35.0 (3.0) |  | 7 (3.3) | 7 (3.3) |  |
| **Radiotherapy** |  |  | < .001 |  |  | 0.575 |  |  | > .99 |
| No | 560 (57.1) | 78 (34.1) |  | 692.3 (52.8) | 581.9 (49.7) |  | 75 (35.2) | 74 (34.7) |  |
| Yes | 420 (42.9) | 151 (65.9) |  | 571.7 (47.2) | 588.1 (50.3) |  | 138 (64.8) | 139 (65.3) |  |

**Supplementary table 20. Analysis for chemotherapy utilization among patients at stage I-III.**

|  | Uni-regression | | Multi-regression | | Matching | |
| --- | --- | --- | --- | --- | --- | --- |
|  | OR | *P* value | OR | *P* value | OR | *P* value |
| **Diagnosis year** |  |  |  |  |  |  |
| 2000-2004 | Reference | - |  |  |  |  |
| 2005-2009 | 1.21 (0.8-1.83) | 0.371 |  |  |  |  |
| 2010-2014 | 0.87 (0.56-1.34) | 0.522 |  |  |  |  |
| 2015-2019 | 1.22 (0.8-1.87) | 0.348 |  |  |  |  |
| **Age** |  |  |  |  |  |  |
| 00-19 years | Reference | - | Reference | - | Reference | - |
| 20-64 years | 0.32 (0.22-0.47) | < .001 | 0.27 (0.17-0.43) | < .001 | 0.24 (0.16-0.38) | < .001 |
| ≥ 65 years | 0.09 (0.05-0.18) | < .001 | 0.09 (0.04-0.2) | < .001 | 0.08 (0.04-0.18) | < .001 |
| **Sex** |  |  |  |  |  |  |
| Male | Reference | - | Reference | - | Reference | - |
| Female | 0.68 (0.51-0.91) | 0.01 | 0.77 (0.55-1.08) | 0.128 | 0.78 (0.56-1.08) | 0.136 |
| **Race** |  |  |  |  |  |  |
| NHW | Reference | - | Reference | - | Reference | - |
| NHB | 1.15 (0.75-1.76) | 0.529 | 0.9 (0.55-1.46) | 0.665 | 0.90 (0.55-1.45) | 0.656 |
| NHAPI | 0.88 (0.48-1.62) | 0.69 | 0.87 (0.45-1.69) | 0.679 | 0.86 (0.44-1.66) | 0.645 |
| Hispanic | 2.15 (1.52-3.05) | < .001 | 1.52 (1.02-2.26) | 0.04 | 1.62 (1.09-2.42) | 0.018 |
| Others | 1.2 (0.34-4.27) | 0.783 | 0.93 (0.22-3.94) | 0.924 | 0.87 (0.21-3.66) | 0.852 |
| **Married** |  |  |  |  |  |  |
| Yes | Reference | - | Reference | - |  |  |
| No | 0.59 (0.44-0.81) | 0.001 | 0.95 (0.66-1.38) | 0.798 |  |  |
| **Income** |  |  |  |  |  |  |
| ≤ $49999 | Reference | - |  |  | Reference | - |
| $50000-74999 | 0.71 (0.47-1.08) | 0.112 |  |  | 0.55 (0.34-0.9) | 0.017 |
| ≥ 75000 | 0.82 (0.52-1.29) | 0.397 |  |  | 0.78 (0.46-1.33) | 0.363 |
| **Location** |  |  |  |  |  |  |
| Urban | Reference | - |  |  |  |  |
| Rural | 0.98 (0.61-1.56) | 0.918 |  |  |  |  |
| **Site** |  |  |  |  |  |  |
| Head and neck | Reference | - | Reference | - |  |  |
| Intracranial | 0.25 (0.03-1.94) | 0.183 | 0.37 (0.04-3.15) | 0.365 |  |  |
| Limb | 2.09 (1.17-3.73) | 0.013 | 1.37 (0.72-2.61) | 0.336 |  |  |
| Others | 2.27 (1.28-4.03) | 0.005 | 1.56 (0.82-2.94) | 0.174 |  |  |
| **Stage** |  |  |  |  |  |  |
| I | Reference | - | Reference | - |  |  |
| II | 0.7 (0.4-1.21) | 0.2 | 3.53 (1.43-8.67) | 0.006 |  |  |
| III | 3.14 (2.26-4.35) | < .001 | 2.03 (1.36-3.03) | 0.001 |  |  |
| **Grade** |  |  |  |  |  |  |
| G0/GX | Reference | - | Reference | - | Reference | - |
| G1 | 0.91 (0.54-1.53) | 0.724 | 0.43 (0.25-0.73) | 0.002 | 1.00 (0.57-1.77) | 0.982 |
| G2 | 2.95 (2.13-4.09) | < .001 | - | - | 2.28 (1.57-3.3) | < .001 |
| **Size** |  |  |  |  |  |  |
| 0-50mm | Reference | - | Reference | - | Reference | - |
| 51-100mm | 5.01 (3.19-7.88) | < .001 | 4.51 (2-10.16) | < .001 | 3.46 (2.13-5.60) | < .001 |
| 101-150mm | 6.76 (4.07-11.25) | < .001 | 5.51 (2.32-13.11) | < .001 | 4.47 (2.56-7.80) | < .001 |
| > 150mm | 9.66 (5.61-16.63) | <.001 | 9.16 (3.81-22.03) | < .001 | 7.60 (4.23-13.68) | < .001 |
| **Surgery** |  |  |  |  |  |  |
| Local excision | Reference | - | Reference | - | Reference | - |
| Radical excision | 1.77 (1.29-2.43) | 0 | 1.33 (0.93-1.9) | 0.112 | 1.38 (0.97-1.97) | 0.074 |
| Amputation of limb | 2.3 (1.42-3.74) | 0.001 | 2.64 (1.48-4.69) | 0.001 | 2.44 (1.40-4.3) | 0.002 |
| Others | 2.29 (1.03-5.1) | 0.042 | 1.92 (0.76-4.87) | 0.169 | 1.81 (0.73-4.52) | 0.204 |
| **Radiotherapy** |  |  |  |  |  |  |
| No | Reference | - | Reference | - | Reference | - |
| Yes | 2.58 (1.91-3.49) | < .001 | 2 (1.41-2.84) | <.001 | 1.95 (1.38-2.77) | < .001 |

**Supplementary table 21. Cox regression of overall survival for patients at stage I-III stratified by chemotherapy.**

|  | Before matching | | | | After IPTW | | | | After PSM | | | |
| --- | --- | --- | --- | --- | --- | --- | --- | --- | --- | --- | --- | --- |
|  | Uni-regression | | Multi-regression | | Uni-regression | | Multi-regression | | Uni-regression | | Multi-regression | |
|  | HR | *P* value | HR | *P* value | HR | *P* value | HR | *P* value | HR | *P* value | HR | *P* value |
| **Diagnosis year** |  |  |  |  |  |  |  |  |  |  |  |  |
| 2000-2004 | Reference | - |  |  | Reference | - | Reference | - | Reference | - | Reference | - |
| 2005-2009 | 1.07 (0.84-1.36) | 0.597 | 1.05 (0.82-1.33) | 0.712 | 1.11 (0.71-1.72) | 0.647 | 1.14 (0.77-1.7) | 0.506 | 1.22 (0.83-1.78) | 0.318 | 1.4 (0.93-2.11) | 0.106 |
| 2010-2014 | 1.31 (1.03-1.67) | 0.028 | 1.17 (0.91-1.5) | 0.218 | 1.29 (0.83-2) | 0.255 | 1.25 (0.84-1.85) | 0.274 | 1.31 (0.89-1.94) | 0.177 | 1.31 (0.86-2.02) | 0.21 |
| 2015-2019 | 1.06 (0.78-1.43) | 0.71 | 0.95 (0.7-1.28) | 0.733 | 0.97 (0.58-1.63) | 0.914 | 0.8 (0.49-1.31) | 0.373 | 1.25 (0.78-1.98) | 0.353 | 1.28 (0.79-2.08) | 0.323 |
| **Age** |  |  |  |  |  |  |  |  |  |  |  |  |
| 00-19 years | Reference | - | Reference | - | Reference | - |  |  | Reference | - | Reference | - |
| 20-64 years | 1.21 (0.89-1.65) | 0.225 | 1.58 (1.14-2.2) | 0.006 | 1.41 (0.96-2.06) | 0.078 | 1.64 (1.12-2.4) | 0.01 | 1.49 (1.03-2.16) | 0.034 | 1.71 (1.15-2.56) | 0.008 |
| ≥ 65 years | 2.13 (1.51-2.99) | < .001 | 3.45 (2.37-5.01) | < .001 | 2.08 (1.21-3.58) | 0.008 | 3.06 (1.81-5.18) | < .001 | 2.46 (1.36-4.46) | 0.003 | 3.14 (1.64-5.98) | 0.001 |
| **Sex** |  |  |  |  |  |  |  |  |  |  |  |  |
| Male | Reference | - | Reference | - | Reference | - |  |  | Reference | - |  |  |
| Female | 0.82 (0.68-0.97) | 0.023 | 0.89 (0.74-1.06) | 0.201 | 0.86 (0.62-1.17) | 0.333 |  |  | 0.96 (0.73-1.27) | 0.791 |  |  |
| **Race** |  |  |  |  |  |  |  |  |  |  |  |  |
| NHW | Reference | - |  |  | Reference | - |  |  |  |  |  |  |
| NHB | 1.12 (0.87-1.44) | 0.374 |  |  | 1.03 (0.64-1.65) | 0.915 |  |  | 1.33 (0.91-1.95) | 0.139 |  |  |
| NHAPI | 0.93 (0.66-1.32) | 0.697 |  |  | 1.2 (0.77-1.89) | 0.42 |  |  | 1.06 (0.6-1.9) | 0.839 |  |  |
| Hispanic | 1.18 (0.94-1.49) | 0.146 |  |  | 1.04 (0.71-1.51) | 0.846 |  |  | 1.26 (0.91-1.73) | 0.159 |  |  |
| Others | 1.17 (0.55-2.47) | 0.685 |  |  | 0.83 (0.31-2.23) | 0.717 |  |  | 1.23 (0.39-3.88) | 0.727 |  |  |
| **Married** |  |  |  |  |  |  |  |  |  |  |  |  |
| Yes | Reference | - | Reference | - | Reference | - |  |  | Reference | - |  |  |
| No | 0.78 (0.65-0.93) | 0.006 | 0.71 (0.59-0.86) | 0.001 | 0.91 (0.66-1.25) | 0.563 |  |  | 0.9 (0.68-1.2) | 0.487 |  |  |
| **Income** |  |  |  |  |  |  |  |  |  |  |  |  |
| ≤ $49999 | Reference | - |  |  | Reference | - |  |  | Reference | - |  |  |
| $50000-74999 | 0.91 (0.7-1.18) | 0.479 |  |  | 0.79 (0.53-1.17) | 0.24 |  |  | 0.77 (0.54-1.11) | 0.166 |  |  |
| ≥ 75000 | 0.78 (0.58-1.04) | 0.089 |  |  | 0.66 (0.41-1.06) | 0.083 |  |  | 0.77 (0.52-1.16) | 0.209 |  |  |
| **Location** |  |  |  |  |  |  |  |  |  |  |  |  |
| Urban | Reference | - |  |  | Reference | - |  |  | Reference | - |  |  |
| Rural | 1.03 (0.78-1.36) | 0.822 |  |  | 1.25 (0.79-1.97) | 0.342 |  |  | 0.92 (0.6-1.41) | 0.708 |  |  |
| **Site** |  |  |  |  |  |  |  |  |  |  |  |  |
| Head and neck | Reference | - | Reference | - | Reference | - | Reference | - | Reference | - | Reference | - |
| Intracranial | 0.73 (0.41-1.31) | 0.29 | 1.08 (0.6-1.95) | 0.801 | 0.73 (0.33-1.62) | 0.439 | 1.1 (0.5-2.41) | 0.82 | 0.47 (0.11-2.03) | 0.311 | 0.69 (0.14-3.48) | 0.655 |
| Limb | 0.66 (0.49-0.87) | 0.004 | 0.44 (0.33-0.6) | < .001 | 0.49 (0.34-0.71) | 0 | 0.34 (0.24-0.5) | < .001 | 0.47 (0.28-0.79) | 0.005 | 0.28 (0.16-0.51) | < .001 |
| Others | 1.03 (0.78-1.36) | 0.829 | 0.71 (0.53-0.95) | 0.02 | 0.87 (0.61-1.24) | 0.436 | 0.66 (0.45-0.97) | 0.033 | 0.95 (0.57-1.57) | 0.826 | 0.6 (0.34-1.04) | 0.066 |
| **Stage** |  |  |  |  |  |  |  |  |  |  |  |  |
| I | Reference | - | Reference | - | Reference | - |  |  | Reference | - | Reference | - |
| II | 0.77 (0.58-1.03) | 0.077 | 1.93 (1.34-2.79) | < .001 | 0.59 (0.33-1.05) | 0.073 |  |  | 0.34 (0.17-0.69) | 0.003 | 0.85 (0.27-2.65) | 0.784 |
| III | 1.66 (1.37-2) | < .001 | 1.45 (1.15-1.83) | 0.002 | 1.33 (0.94-1.87) | 0.106 |  |  | 0.99 (0.73-1.36) | 0.968 | 0.91 (0.63-1.31) | 0.606 |
| **Grade** |  |  |  |  |  |  |  |  |  |  |  |  |
| G0/GX | Reference | - | Reference | - | Reference | - |  |  | Reference | - |  |  |
| G1 | 0.71 (0.53-0.95) | 0.023 | 0.45 (0.33-0.6) | < .001 | 0.83 (0.49-1.4) | 0.485 |  |  | 0.65 (0.39-1.09) | 0.102 |  |  |
| G2 | 1.69 (1.4-2.04) | < .001 | - | - | 1.17 (0.82-1.67) | 0.394 |  |  | 0.95 (0.69-1.3) | 0.741 |  |  |
| **Size** |  |  |  |  |  |  |  |  |  |  |  |  |
| 0-50mm | Reference | - | Reference | - | Reference | - | Reference | - | Reference | - | Reference | - |
| 51-100mm | 1.93 (1.55-2.41) | < .001 | 2.18 (1.58-3.01) | < .001 | 1.94 (1.26-2.98) | 0.002 | 2.14 (1.4-3.28) | < .001 | 2.3 (1.31-4.02) | 0.004 | 2.28 (0.86-6) | 0.097 |
| 101-150mm | 2.65 (2.04-3.45) | < .001 | 2.73 (1.89-3.95) | < .001 | 2.56 (1.61-4.05) | 0 | 3.03 (1.93-4.76) | < .001 | 3.45 (1.91-6.23) | 0 | 3.46 (1.29-9.32) | 0.014 |
| > 150mm | 3.35 (2.51-4.47) | < .001 | 3.9 (2.67-5.7) | < .001 | 2.86 (1.8-4.57) | 0 | 3.36 (2.07-5.46) | < .001 | 4.88 (2.68-8.87) | 0 | 4.79 (1.78-12.91) | 0.002 |
| **Surgery** |  |  |  |  |  |  |  |  |  |  |  |  |
| Local excision | Reference | - |  |  | Reference | - |  |  | Reference | - | Reference | - |
| Radical excision | 1.1 (0.92-1.33) | 0.299 |  |  | 1.01 (0.72-1.43) | 0.944 |  |  | 1.02 (0.75-1.37) | 0.921 | 0.87 (0.64-1.18) | 0.372 |
| Amputation of limb | 1.05 (0.77-1.44) | 0.749 |  |  | 1.1 (0.71-1.69) | 0.677 |  |  | 0.99 (0.63-1.55) | 0.97 | 0.88 (0.54-1.44) | 0.622 |
| Others | 1.16 (0.7-1.93) | 0.558 |  |  | 1.49 (0.76-2.9) | 0.246 |  |  | 2.12 (1.13-3.97) | 0.02 | 1.26 (0.64-2.51) | 0.501 |
| **Chemotherapy** |  |  |  |  |  |  |  |  |  |  |  |  |
| No | Reference | - | Reference | - | Reference | - | Reference | - | Reference | - | Reference | - |
| Yes | 1.7 (1.39-2.08) | <.001 | 1.45 (1.15-1.81) | 0.001 | 1.42 (1.04-1.94) | 0.027 | 1.8 (1.34-2.41) | < .001 | 1.3 (0.99-1.71) | 0.056 | 1.28 (0.96-1.69) | 0.089 |
| **Radiotherapy ^a^** |  |  |  |  |  |  |  |  |  |  |  |  |
| No | Reference | - | Reference | - | Reference | - |  |  | Reference | - |  |  |
| Yes | 1.35 (1.14-1.61) | 0.001 | 0.99 (0.82-1.19) | 0.881 | 1.08 (0.78-1.48) | 0.656 |  |  | 1.09 (0.82-1.46) | 0.547 |  |  |

**Supplementary table 22. Restricted mean survival time for patients at stage I-III stratified by chemotherapy.**

|  | **Unadjusted** | | **PSM** | |
| --- | --- | --- | --- | --- |
|  | **No** | **Yes** | **No** | **Yes** |
| **Restricted at 1 year** | | | | |
| RSMT, month | 11.4 (11.3-11.6) | 11.4 (11.1-11.6) | 11.5 (11.2-11.7) | 11.4 (11.2-11.6) |
| RMST difference | -0.1 (-0.4-0.2) (*P* = 0.483) | | -0.1 (-0.4 - 0.3) (*P* = 0.722) | |
| **Restricted at 3 years** | | | | |
| RSMT, month | 30.7 (30.1-31.4) | 27.6 (26.1-29.1) | 29.7 (28.2-31.1) | 27.8 (26.3-29.3) |
| RMST difference | -3.1 (-4.7 - -1.5) (*P* < .001) | | -1.8 (-3.9 - 0.3) (*P* = 0.086) | |
| **Restricted at 5 years** | | | | |
| RSMT, month | 47.1 (45.8-48.4) | 39.6 (36.7-42.5) | 43.8 (40.9-46.7) | 39.9 (36.9-42.9) |
| RMST difference | -7.5 (-10.7 – 4.3) (P < .001) | | -3.9 (-8.0-0.2) (*P* = 0.065) | |
| **Restricted at 10 years** | | | | |
| RSMT, month | 83.4 (80.4-86.5) | 64.5 (57.9-71.2) | 74.3 (67.4-81.2) | 64.9 (58.0-71.7) |
| RMST difference | -18.9 (-26.2 - -11.6) (*P* < .001) | | -9.4 (-19.1 – 0.36) (*P* = 0.059) | |

**Supplementary table 23. Subgroup analysis for patients at stage I-III with chemotherapy.**

|  | Before matching | | After IPTW | | After PSM | |
| --- | --- | --- | --- | --- | --- | --- |
|  | Hazard ratio | *P* value | Hazard ratio | *P* value | Hazard ratio | *P* value |
| **Diagnosis year** |  | 0.95 |  | 0.964 |  | 0.642 |
| 2000-2004 | 1.47 (0.97-2.22) | 0.068 | 1.25 (0.67-2.33) | 0.483 | 1.29 (0.71-2.34) | 0.408 |
| 2005-2009 | 2.23 (1.58-3.16) | 0 | 1.97 (1.22-3.19) | 0.006 | 1.81 (1.1-2.97) | 0.019 |
| 2010-2014 | 1.27 (0.84-1.92) | 0.251 | 1.33 (0.77-2.3) | 0.315 | 0.86 (0.52-1.42) | 0.549 |
| 2015-2019 | 2.08 (1.25-3.48) | 0.005 | 1.42 (0.72-2.82) | 0.316 | 1.56 (0.77-3.14) | 0.215 |
| **Age** |  | 0.006 |  | 0.226 |  | 0.03 |
| 00-19 years | 3.78 (2.02-7.05) | 0 | 2.54 (1.24-5.24) | 0.011 | 2.78 (1.32-5.83) | 0.007 |
| 20-64 years | 1.86 (1.46-2.38) | 0 | 1.49 (1.05-2.12) | 0.027 | 1.15 (0.85-1.57) | 0.367 |
| ≥ 65 years | 1.2 (0.58-2.47) | 0.623 | 0.93 (0.33-2.61) | 0.894 | 0.88 (0.33-2.37) | 0.804 |
| **Sex** |  | 0.625 |  | 0.93 |  | 0.577 |
| Male | 1.61 (1.24-2.09) | 0 | 1.44 (1.01-2.07) | 0.045 | 1.38 (0.97-1.97) | 0.073 |
| Female | 1.77 (1.28-2.44) | 0 | 1.4 (0.86-2.29) | 0.175 | 1.18 (0.77-1.81) | 0.448 |
| **Race** |  | 0.553 |  | 0.739 |  | 0.578 |
| NHW | 1.57 (1.18-2.11) | 0.002 | 1.47 (0.93-2.3) | 0.097 | 1.26 (0.84-1.89) | 0.272 |
| NHB | 1.6 (0.94-2.7) | 0.083 | 1.11 (0.51-2.41) | 0.786 | 0.96 (0.5-1.84) | 0.909 |
| NHAPI | 2.18 (1.02-4.65) | 0.045 | 2.86 (1.49-5.48) | 0.002 | 2.55 (0.77-8.38) | 0.124 |
| Hispanic | 1.91 (1.28-2.86) | 0.002 | 1.27 (0.72-2.25) | 0.408 | 1.58 (0.96-2.61) | 0.073 |
| Others | 0.58 (0.07-4.85) | 0.616 | 0.35 (0.04-2.84) | 0.327 | 0.44 (0.04-5.13) | 0.512 |
| **Married** |  | 0.263 |  | 0.271 |  | 0.611 |
| Yes | 1.51 (1.18-1.95) | 0.001 | 1.23 (0.82-1.85) | 0.317 | 1.24 (0.88-1.73) | 0.22 |
| No | 1.96 (1.39-2.77) | 0 | 1.75 (1.11-2.75) | 0.016 | 1.44 (0.91-2.27) | 0.121 |
| **Income** |  | 0.433 |  | 0.634 |  | 0.516 |
| ≤ $49999 | 1.82 (1.1-3.01) | 0.021 | 1.71 (0.96-3.04) | 0.067 | 1.19 (0.63-2.23) | 0.592 |
| $50000-74999 | 1.44 (1.1-1.9) | 0.009 | 1.32 (0.89-1.97) | 0.172 | 1.25 (0.86-1.82) | 0.249 |
| ≥ 75000 | 2.22 (1.51-3.25) | 0 | 1.4 (0.73-2.69) | 0.31 | 1.56 (0.94-2.59) | 0.084 |
| **Location** |  | 0.501 |  | 0.194 |  | 0.421 |
| Urban | 1.66 (1.34-2.07) | 0 | 1.33 (0.95-1.86) | 0.097 | 1.25 (0.94-1.67) | 0.125 |
| Rural | 2.01 (1.12-3.6) | 0.019 | 2.14 (1.13-4.07) | 0.02 | 1.96 (0.85-4.55) | 0.11 |
| **Site** |  | 0.968 |  | 0.543 |  | 0.189 |
| Head and neck | 2.4 (1.24-4.66) | 0.009 | 2.77 (1.79-4.29) | 0 | 1.54 (0.58-4.08) | 0.389 |
| Intracranial | 6.21 (0.72-53.18) | 0.096 | 6.15 (2.51-15.04) | 0 | - | - |
| Limb | 1.53 (1.09-2.15) | 0.015 | 1.18 (0.72-1.91) | 0.514 | 1.41 (0.89-2.21) | 0.141 |
| Others | 1.77 (1.34-2.34) | 0 | 1.51 (0.99-2.31) | 0.057 | 1.12 (0.78-1.62) | 0.543 |
| **Stage** |  | 0.048 |  | 0.202 |  | 0.615 |
| I | 2.17 (1.49-3.15) | 0 | 2 (1.08-3.68) | 0.027 | 1.39 (0.8-2.4) | 0.242 |
| II | 1.01 (0.44-2.34) | 0.983 | 1 (0.4-2.52) | 0.995 | 2.15 (0.53-8.63) | 0.282 |
| III | 1.27 (0.98-1.65) | 0.075 | 1.23 (0.91-1.65) | 0.172 | 1.26 (0.91-1.74) | 0.168 |
| **Grade** |  | 0.002 |  | 0.013 |  | 0.214 |
| G0/GX | 2.17 (1.49-3.15) | 0 | 2 (1.08-3.68) | 0.027 | 1.39 (0.8-2.4) | 0.242 |
| G1 | 3.14 (1.69-5.83) | 0 | 2.66 (1.21-5.83) | 0.014 | 3.38 (1.34-8.52) | 0.01 |
| G2 | 1.07 (0.82-1.39) | 0.642 | 0.85 (0.6-1.21) | 0.377 | 1.08 (0.77-1.51) | 0.656 |
| **Size** |  | 0.537 |  | 0.741 |  | 0.799 |
| 0-50mm | 1.31 (0.68-2.49) | 0.418 | 1.38 (0.65-2.9) | 0.402 | 2.8 (0.88-8.92) | 0.083 |
| 51-100mm | 1.16 (0.85-1.59) | 0.357 | 1.38 (0.94-2.03) | 0.104 | 1.14 (0.77-1.71) | 0.507 |
| 101-150mm | 1.37 (0.9-2.09) | 0.142 | 1.52 (0.91-2.53) | 0.114 | 1.16 (0.67-2.02) | 0.593 |
| > 150mm | 1.72 (1.07-2.7) | 0.025 | 1.51 (0.89-2.56) | 0.123 | 1.31 (0.73-2.35) | 0.3 |
| **Surgery** |  | 0.561 |  | 0.737 |  | 0.343 |
| Local excision | 1.75 (1.26-2.43) | 0.001 | 1.45 (0.88-2.39) | 0.149 | 1.44 (0.93-2.23) | 0.1 |
| Radical excision | 1.49 (1.09-2.03) | 0.012 | 1.31 (0.83-2.08) | 0.244 | 1.28 (0.85-1.94) | 0.237 |
| Amputation of limb | 1.99 (1.09-3.62) | 0.025 | 1.77 (0.9-3.45) | 0.096 | 1.11 (0.51-2.43) | 0.797 |
| Others | 2.52 (0.94-6.74) | 0.065 | 1.62 (0.55-4.74) | 0.381 | 0.54 (0.15-1.91) | 0.342 |
| **Radiotherapy** |  | 0.01 |  | 0.157 |  | 0.096 |
| No | 2.18 (1.57-3.01) | 0 | 1.72 (1.06-2.81) | 0.03 | 1.75 (1.08-2.83) | 0.023 |
| Yes | 1.33 (1.02-1.73) | 0.034 | 1.14 (0.79-1.64) | 0.496 | 1.11 (0.8-1.54) | 0.548 |

**Supplementary table 24. Baseline of patients at stage IV stratified by radiotherapy before and after matching.**

|  | Before matching | | | After IPTW | | | After PSM | | |
| --- | --- | --- | --- | --- | --- | --- | --- | --- | --- |
|  | No | Yes | *P* value | No | Yes | *P* value | No | Yes | *P* value |
| **Patients** | 131 (100) | 118 (100) |  | 246.9 (100) | 250.8 (100) |  | 93 (100) | 93 (100) |  |
| **Diagnosis year** |  |  | 0.391 |  |  | 0.498 |  |  | 0.253 |
| 2000-2004 | 26 (19.8) | 18 (15.3) |  | 49.2 (19.9) | 39.5 (15.8) |  | 20 (21.5) | 16 (17.2) |  |
| 2005-2009 | 42 (32.1) | 31 (26.3) |  | 76.6 (31.0) | 64.2 (25.6) |  | 31 (33.3) | 22 (23.7) |  |
| 2010-2014 | 34 (26.0) | 34 (28.8) |  | 68.4 (27.7) | 75.8 (30.2) |  | 24 (25.8) | 28 (30.1) |  |
| 2015-2019 | 29 (22.1) | 35 (29.7) |  | 52.7 (21.3) | 71.2 (28.4) |  | 18 (19.4) | 27 (29.0) |  |
| **Age** |  |  | 0.192 |  |  | 0.992 |  |  | 0.793 |
| 00-19 years | 13 (9.92) | 18 (15.3) |  | 29.0 (11.7) | 30.1 (12.0) |  | 9 (9.7) | 11 (11.8) |  |
| 20-64 years | 97 (74.0) | 75 (63.6) |  | 175.1 (70.9) | 175.9 (70.1) |  | 65 (69.9) | 66 (71.0) |  |
| ≥ 65 years | 21 (16.0) | 25 (21.2) |  | 42.9 (17.4) | 44.8 (17.9) |  | 19 (20.4) | 16 (17.2) |  |
| **Sex** |  |  | 0.227 |  |  | 0.484 |  |  | 0.454 |
| Male | 82 (62.6) | 64 (54.2) |  | 150.5 (61.0) | 141.4 (56.4) |  | 59 (63.4) | 53 (57.0) |  |
| Female | 49 (37.4) | 54 (45.8) |  | 96.4 (39.0) | 109.4 (43.6) |  | 34 (36.6) | 40 (43.0) |  |
| **Race** |  |  | 0.496 |  |  | 0.426 |  |  | 0.559 |
| NHW | 68 (51.9) | 73 (61.9) |  | 124.5 (50.4) | 148.4 (59.2) |  | 49 (52.7) | 55 (59.1) |  |
| NHB | 19 (14.5) | 11 (9.32) |  | 34.7 (14.1) | 32.0 (12.8) |  | 12 (12.9) | 9 (9.7) |  |
| NHAPI | 17 (13.0) | 11 (9.32) |  | 37.3 (15.1) | 21.0 (8.4) |  | 16 (17.2) | 10 (10.8) |  |
| Hispanic | 25 (19.1) | 22 (18.6) |  | 45.9 (18.6) | 47.9 (19.1) |  | 14 (15.1) | 18 (19.4) |  |
| Others | 2 (1.53) | 1 (0.85) |  | 4.5 (1.8) | 1.5 (0.6) |  | 2 (2.2) | 1 (1.1) |  |
| **Married** |  |  | 1 |  |  | 0.985 |  |  | > .99 |
| Yes | 80 (61.1) | 72 (61.0) |  | 152.4 (61.7) | 154.6 (51.6) |  | 55 (59.1) | 54 (58.1) |  |
| No | 51 (38.9) | 46 (39.0) |  | 94.5 (38.3) | 96.2 (38.4) |  | 38 (40.9) | 39 (41.9) | 1 |
| **Income** |  |  | 0.095 |  |  | 0.997 |  |  | 0.958 |
| ≤ $49999 | 17 (13.0) | 10 (8.47) |  | 25.9 (10.5) | 26.8 (10.7) |  | 7 (7.5) | 8 (8.6) |  |
| $50000-74999 | 81 (61.8) | 64 (54.2) |  | 146.8 (59.4) | 147.7 (58.9) |  | 58 (62.4) | 58 (62.4) |  |
| ≥ 75000 | 33 (25.2) | 44 (37.3) |  | 74.3 (30.1) | 76.3 (30.4) |  | 28 (30.1) | 27 (29.0) |  |
| **Location** |  |  | 1 |  |  | 0.629 |  |  | 0.82 |
| Urban | 114 (87.0) | 103 (87.3) |  | 219.4 (88.9) | 217.8 (86.8) |  | 83 (89.2) | 81 (87.1) |  |
| Rural | 17 (13.0) | 15 (12.7) |  | 27.5 (11.1) | 33.0 (13.2) |  | 10 (10.8) | 12 (12.9) |  |
| **Site** |  |  | 0.242 |  |  | 0.212 |  |  | 0.578 |
| Head and neck | 17 (13.0) | 16 (13.6) |  | 31.8 (12.9) | 35.7 (14.3) |  | 14 (15.1) | 12 (12.9) |  |
| Intracranial | 1 (0.76) | 6 (5.08) |  | 1.6 (0.7) | 12.9 (5.1) |  | 1 (1.1) | 4 (4.3) |  |
| Limb | 39 (29.8) | 32 (27.1) |  | 75.6 (30.6) | 70.8 (28.2) |  | 26 (28.0) | 25 (26.9) |  |
| Others | 74 (56.5) | 64 (54.2) |  | 137.9 (55.9) | 131.4 (52.4) |  | 52 (55.9) | 52 (55.9) |  |
| **Grade** |  |  | 0.714 |  |  | 0.838 |  |  | 0.573 |
| G0/GX | 61 (46.6) | 59 (50.0) |  | 114.0 (46.1) | 125.6 (50.1) |  | 40 (43.0) | 47 (50.5) |  |
| G1 | 6 (4.58) | 7 (5.93) |  | 13.5 (5.5) | 12.8 (5.1) |  | 5 (5.4) | 5 (5.4) |  |
| G2 | 64 (48.9) | 52 (44.1) |  | 119.4 (48.4) | 112.4 (44.8) |  | 48 (51.6) | 41 (44.1) |  |
| **Size** |  |  | 0.01 |  |  | 0.999 |  |  | 0.993 |
| 0-50mm | 28 (21.4) | 22 (18.6) |  | 49.4 (20.0) | 49.2 (19.6) |  | 18 (19.4) | 19 (20.4) |  |
| 51-100mm | 49 (37.4) | 37 (31.4) |  | 85.8 (34.7) | 85.8 (34.2) |  | 35 (37.6) | 34 (36.6) |  |
| 101-150mm | 22 (16.8) | 41 (34.7) |  | 61.1 (24.7) | 62.9 (25.1) |  | 22 (23.7) | 23 (24.7) |  |
| > 150mm | 32 (24.4) | 18 (15.3) |  | 50.7 (20.5) | 52.8 (21.1) |  | 18 (19.4) | 17 (18.3) |  |
| **Surgery** |  |  | 0.65 |  |  | 0.741 |  |  | 0.532 |
| No | 42 (32.1) | 42 (35.6) |  | 81.3 (32.9) | 87.9 (35.0) |  | 28 (30.1) | 33 (35.5) |  |
| Yes | 89 (67.9) | 76 (64.4) |  | 165.6 (67.1) | 162.9 (65.0) |  | 65 (69.9) | 60 (64.5) |  |

**Supplementary table 25. Analysis for radiotherapy utilization among patients at stage IV.**

|  | Uni-regression | | Multi-regression | | Matching | |
| --- | --- | --- | --- | --- | --- | --- |
|  | OR | *P* value | OR | *P* value | OR | *P* value |
| **Diagnosis year** |  |  |  |  |  |  |
| 2000-2004 | Reference | - |  |  |  |  |
| 2005-2009 | 1.07 (0.5-2.28) | 0.869 |  |  |  |  |
| 2010-2014 | 1.44 (0.67-3.11) | 0.347 |  |  |  |  |
| 2015-2019 | 1.74 (0.8-3.79) | 0.161 |  |  |  |  |
| **Age** |  |  |  |  |  |  |
| 00-19 years | Reference | - |  |  | Reference | - |
| 20-64 years | 0.56 (0.26-1.21) | 0.14 |  |  | 0.51 (0.23-1.16) | 0.109 |
| ≥ 65 years | 0.86 (0.34-2.16) | 0.747 |  |  | 0.9 (0.34-2.39) | 0.836 |
| **Sex** |  |  |  |  |  |  |
| Male | Reference | - |  |  |  |  |
| Female | 1.41 (0.85-2.34) | 0.182 |  |  |  |  |
| **Race** |  |  |  |  |  |  |
| NHW | Reference | - |  |  |  |  |
| NHB | 0.54 (0.24-1.22) | 0.136 |  |  |  |  |
| NHAPI | 0.6 (0.26-1.38) | 0.23 |  |  |  |  |
| Hispanic | 0.82 (0.42-1.59) | 0.556 |  |  |  |  |
| Others | 0.47 (0.04-5.25) | 0.537 |  |  |  |  |
| **Married** |  |  |  |  |  |  |
| Yes | Reference | - |  |  |  |  |
| No | 1 (0.6-1.67) | 0.993 |  |  |  |  |
| **Income** |  |  |  |  |  |  |
| ≤ $49999 | Reference | - |  |  | Reference | - |
| $50000-74999 | 1.34 (0.58-3.13) | 0.495 |  |  | 1.37 (0.56-3.33) | 0.488 |
| ≥ 75000 | 2.27 (0.92-5.59) | 0.075 |  |  | 2.35 (0.91-6.09) | 0.078 |
| **Location** |  |  |  |  |  |  |
| Urban | Reference | - |  |  |  |  |
| Rural | 0.98 (0.46-2.05) | 0.95 |  |  |  |  |
| **Site** |  |  |  |  |  |  |
| Head and neck | Reference | - |  |  |  |  |
| Intracranial | 6.37 (0.69-58.91) | 0.103 |  |  |  |  |
| Limb | 0.87 (0.38-1.99) | 0.745 |  |  |  |  |
| Others | 0.92 (0.43-1.97) | 0.827 |  |  |  |  |
| **Grade** |  |  |  |  |  |  |
| G0/GX | Reference | - |  |  |  |  |
| G1 | 1.21 (0.38-3.8) | 0.749 |  |  |  |  |
| G2 | 0.84 (0.5-1.4) | 0.504 |  |  |  |  |
| **Size** |  |  |  |  |  |  |
| 0-50mm | Reference | - | Reference | - | Reference | - |
| 51-100mm | 0.96 (0.48-1.94) | 0.912 | 0.96 (0.48-1.94) | 0.912 | 0.88 (0.43-1.81) | 0.729 |
| 101-150mm | 2.37 (1.11-5.08) | 0.026 | 2.37 (1.11-5.08) | 0.026 | 2.31 (1.05-5.07) | 0.036 |
| > 150mm | 0.72 (0.32-1.6) | 0.415 | 0.72 (0.32-1.6) | 0.415 | 0.6 (0.26-1.39) | 0.232 |
| **Surgery** |  |  |  |  |  |  |
| No | Reference | - |  |  |  |  |
| Yes | 0.85 (0.5-1.44) | 0.556 |  |  |  |  |
| **Chemotherapy** |  |  |  |  |  |  |
| No | Reference | - |  |  |  |  |
| Yes | 0.99 (0.6-1.64) | 0.984 |  |  |  |  |

**Supplementary table 26. Cox regression of overall survival for patients at stage IV stratified by radiotherapy.**

|  | Before matching | | | | After IPTW | | | | After PSM | | | |
| --- | --- | --- | --- | --- | --- | --- | --- | --- | --- | --- | --- | --- |
|  | Uni-regression | | Multi-regression | | Uni-regression | | Multi-regression | | Uni-regression | | Multi-regression | |
|  | HR | *P* value | HR | *P* value | HR | *P* value | HR | *P* value | HR | *P* value | HR | *P* value |
| **Diagnosis year** |  |  |  |  |  |  |  |  |  |  |  |  |
| 2000-2004 | Reference | - |  |  | Reference | - | Reference | - | Reference | - | Reference | - |
| 2005-2009 | 0.97 (0.64-1.45) | 0.873 | 0.82 (0.54-1.24) | 0.347 | 0.97 (0.65-1.46) | 0.9 | 0.86 (0.57-1.31) | 0.494 | 1.01 (0.64-1.6) | 0.96 | 0.99 (0.62-1.57) | 0.954 |
| 2010-2014 | 1.38 (0.92-2.08) | 0.118 | 1.14 (0.74-1.74) | 0.552 | 1.52 (1.02-2.28) | 0.04 | 1.35 (0.89-2.04) | 0.157 | 1.65 (1.05-2.61) | 0.031 | 1.55 (0.96-2.5) | 0.07 |
| 2015-2019 | 1.18 (0.76-1.83) | 0.458 | 1.11 (0.7-1.76) | 0.649 | 1.19 (0.77-1.83) | 0.435 | 1.23 (0.79-1.91) | 0.369 | 1.3 (0.79-2.15) | 0.3 | 1.39 (0.82-2.37) | 0.217 |
| **Age** |  |  |  |  |  |  |  |  |  |  |  |  |
| 00-19 years | Reference | - | Reference | - | Reference | - |  |  | Reference | - |  |  |
| 20-64 years | 1.66 (1.05-2.63) | 0.031 | 1.65 (1.04-2.65) | 0.035 | 1.44 (0.86-2.4) | 0.165 |  |  | 1.2 (0.7-2.05) | 0.517 |  |  |
| ≥ 65 years | 2 (1.18-3.39) | 0.01 | 2.49 (1.43-4.32) | 0.001 | 1.75 (0.97-3.15) | 0.063 |  |  | 1.51 (0.81-2.82) | 0.19 |  |  |
| **Sex** |  |  |  |  |  |  |  |  |  |  |  |  |
| Male | Reference | - |  |  | Reference | - |  |  | Reference | - |  |  |
| Female | 0.87 (0.66-1.15) | 0.332 |  |  | 0.85 (0.64-1.14) | 0.282 |  |  | 0.9 (0.65-1.24) | 0.512 |  |  |
| **Race** |  |  |  |  |  |  |  |  |  |  |  |  |
| NHW | Reference | - |  |  | Reference | - |  |  |  |  |  |  |
| NHB | 1.17 (0.77-1.79) | 0.467 |  |  | 1.21 (0.81-1.82) | 0.355 |  |  | 1.1 (0.66-1.83) | 0.707 |  |  |
| NHAPI | 0.97 (0.62-1.51) | 0.886 |  |  | 1.11 (0.66-1.86) | 0.698 |  |  | 0.91 (0.57-1.46) | 0.693 |  |  |
| Hispanic | 0.92 (0.63-1.35) | 0.678 |  |  | 0.92 (0.62-1.37) | 0.686 |  |  | 0.81 (0.51-1.28) | 0.365 |  |  |
| Others | 0.7 (0.17-2.84) | 0.62 |  |  | 0.75 (0.12-4.72) | 0.757 |  |  | 0.67 (0.17-2.72) | 0.575 |  |  |
| **Married** |  |  |  |  |  |  |  |  |  |  |  |  |
| Yes | Reference | - |  |  | Reference | - |  |  | Reference | - |  |  |
| No | 1.05 (0.79-1.39) | 0.732 |  |  | 0.97 (0.72-1.31) | 0.837 |  |  | 0.97 (0.71-1.34) | 0.874 |  |  |
| **Income** |  |  |  |  |  |  |  |  |  |  |  |  |
| ≤ $49999 | Reference | - |  |  | Reference | - |  |  | Reference | - |  |  |
| $50000-74999 | 0.92 (0.57-1.46) | 0.712 |  |  | 1.04 (0.58-1.87) | 0.886 |  |  | 0.88 (0.48-1.6) | 0.667 |  |  |
| ≥ 75000 | 1.07 (0.65-1.74) | 0.798 |  |  | 1.27 (0.71-2.3) | 0.421 |  |  | 1.16 (0.62-2.17) | 0.649 |  |  |
| **Location** |  |  |  |  |  |  |  |  |  |  |  |  |
| Urban | Reference | - |  |  | Reference | - |  |  | Reference | - |  |  |
| Rural | 0.94 (0.62-1.42) | 0.772 |  |  | 0.87 (0.55-1.37) | 0.538 |  |  | 0.96 (0.6-1.56) | 0.88 |  |  |
| **Site** |  |  |  |  |  |  |  |  |  |  |  |  |
| Head and neck | Reference | - |  |  | Reference | - |  |  | Reference | - |  |  |
| Intracranial | 0.59 (0.2-1.72) | 0.337 |  |  | 0.64 (0.24-1.75) | 0.388 |  |  | 0.95 (0.32-2.79) | 0.921 |  |  |
| Limb | 1.38 (0.84-2.24) | 0.201 |  |  | 1.34 (0.78-2.33) | 0.293 |  |  | 1.26 (0.74-2.17) | 0.399 |  |  |
| Others | 1.52 (0.96-2.4) | 0.074 |  |  | 1.46 (0.86-2.48) | 0.16 |  |  | 1.29 (0.78-2.12) | 0.319 |  |  |
| **Grade** |  |  |  |  |  |  |  |  |  |  |  |  |
| G0/GX | Reference | - |  |  | Reference | - | Reference | - | Reference | - |  |  |
| G1 | 0.71 (0.53-0.95) | 0.023 |  |  | 0.55 (0.3-1) | 0.05 | 0.46 (0.22-0.96) | 0.038 | 0.56 (0.24-1.3) | 0.177 |  |  |
| G2 | 1.69 (1.4-2.04) | < .001 |  |  | 1.19 (0.88-1.61) | 0.249 | 1.14 (0.83-1.57) | 0.404 | 1.33 (0.96-1.84) | 0.084 |  |  |
| **Size** |  |  |  |  |  |  |  |  |  |  |  |  |
| 0-50mm | Reference | - | Reference | - | Reference | - | Reference | - | Reference | - | Reference | - |
| 51-100mm | 1.93 (1.28-2.91) | 0.002 | 2.04 (1.34-3.1) | 0.001 | 1.83 (1.21-2.77) | 0.004 | 1.72 (1.12-2.63) | 0.013 | 1.67 (1.06-2.65) | 0.028 | 1.61 (1-2.57) | 0.048 |
| 101-150mm | 1.78 (1.15-2.75) | 0.009 | 2.19 (1.38-3.47) | 0.001 | 1.99 (1.3-3.04) | 0.001 | 1.95 (1.24-3.08) | 0.004 | 1.83 (1.11-3) | 0.017 | 1.67 (1-2.79) | 0.048 |
| > 150mm | 1.91 (1.22-3.01) | 0.005 | 2.07 (1.3-3.28) | 0.002 | 1.87 (1.2-2.93) | 0.006 | 1.78 (1.12-2.82) | 0.014 | 1.77 (1.05-2.97) | 0.031 | 1.73 (1.03-2.9) | 0.04 |
| **Surgery** |  |  |  |  |  |  |  |  |  |  |  |  |
| No | Reference | - | Reference | - | Reference | - | Reference | - | Reference | - | Reference | - |
| Yes | 0.66 (0.49-0.87) | 0.004 | 0.62 (0.46-0.83) | 0.002 | 0.69 (0.5-0.94) | 0.02 | 0.72 (0.52-0.99) | 0.042 | 0.68 (0.48-0.94) | 0.02 | 0.65 (0.46-0.92) | 0.015 |
| **Chemotherapy** |  |  |  |  |  |  |  |  |  |  |  |  |
| No | Reference | - |  |  | Reference | - | Reference | - | Reference | - |  |  |
| Yes | 0.98 (0.74-1.3) | 0.882 |  |  | 0.96 (0.71-1.3) | 0.801 | 1.8 (1.34-2.41) | < .001 | 1.07 (0.77-1.47) | 0.694 |  |  |
| **Radiotherapy** |  |  |  |  |  |  |  |  |  |  |  |  |
| No | Reference | - | Reference | - | Reference | - | Reference | - | Reference | - | Reference | - |
| Yes | 0.63 (0.48-0.83) | 0.001 | 0.57 (0.42-0.77) | < .001 | 0.61 (0.46-0.81) | 0.001 | 0.54 (0.4-0.74) | 0 | 0.65 (0.47-0.89) | 0.007 | 0.59 (0.42-0.82) | 0.002 |

**Supplementary table 27. Restricted mean survival time for patients at stage IV stratified by radiotherapy.**

|  | **Unadjusted** | | **PSM** | |
| --- | --- | --- | --- | --- |
|  | **No** | **Yes** | **No** | **Yes** |
| **Restricted at 1 year** | | | | |
| RSMT, month | 7.3 (6.53-8.06) | 9.41 (8.76-10.06) | 7.21 (6.29-8.13) | 9.37 (8.62-10.11) |
| RMST difference | 2.11 (1.11-3.11) (*P* < .001) | | 2.16 (0.98-3.34) (*P* < .001) | |
| **Restricted at 3 years** | | | | |
| RSMT, month | 11.97 (9.89-14.05) | 18.26 (15.9-20.61) | 11.92 (9.45-14.39) | 18.13 (15.5-20.77) |
| RMST difference | 6.29 (3.15-9.43) (*P* < .001) | | 6.21 (2.6-9.82) (*P* = 0.001) | |
| **Restricted at 5 years** | | | | |
| RSMT, month | 15.61 (12.12-19.11) | 23.97 (19.96-27.98) | 15.48 (11.39-19.56) | 23.64 (19.18-28.09) |
| RMST difference | 8.36 (3.04-13.68) (P = 0.002) | | 8.16 (2.11-14.21) (*P* = 0.008) | |
| **Restricted at 10 years** | | | | |
| RSMT, month | 22.73 (16.08-29.38) | 34.55 (26.68-42.42) | 22.43 (14.73-30.14) | 33.22 (24.63-41.81) |
| RMST difference | 11.82 (1.52-22.12) (*P* = 0.025) | | 10.79 (-0.75-22.33) (*P* = 0.067) | |

**Supplementary table 28. Subgroup analysis for patients at stage IV with radiotherapy.**

|  | Before matching | | After IPTW | | After PSM | |
| --- | --- | --- | --- | --- | --- | --- |
|  | Hazard ratio | *P* value | Hazard ratio | *P* value | Hazard ratio | *P* value |
| **Diagnosis year** |  | 0.417 |  | 0.414 |  | 0.061 |
| 2000-2004 | 0.69 (0.36-1.32) | 0.261 | 0.62 (0.33-1.17) | 0.14 | 0.91 (0.45-1.85) | 0.796 |
| 2005-2009 | 0.57 (0.34-0.96) | 0.035 | 0.54 (0.31-0.92) | 0.023 | 0.49 (0.27-0.92) | 0.025 |
| 2010-2014 | 0.74 (0.44-1.23) | 0.243 | 0.79 (0.47-1.32) | 0.369 | 0.8 (0.45-1.43) | 0.461 |
| 2015-2019 | 0.42 (0.23-0.79) | 0.007 | 0.36 (0.18-0.74) | 0.005 | 0.19 (0.09-0.43) | < .001 |
| **Age** |  | 0.542 |  | 0.765 |  | 0.602 |
| 00-19 years | 0.74 (0.31-1.76) | 0.494 | 0.62 (0.26-1.44) | 0.264 | 0.82 (0.3-2.28) | 0.708 |
| 20-64 years | 0.56 (0.4-0.79) | 0.001 | 0.55 (0.39-0.78) | 0.001 | 0.56 (0.38-0.81) | 0.002 |
| ≥ 65 years | 0.92 (0.49-1.73) | 0.789 | 0.91 (0.48-1.72) | 0.779 | 0.99 (0.48-2.05) | 0.971 |
| **Sex** |  | 0.046 |  | 0.027 |  | 0.034 |
| Male | 0.82 (0.57-1.19) | 0.294 | 0.81 (0.56-1.18) | 0.276 | 0.88 (0.58-1.33) | 0.546 |
| Female | 0.43 (0.28-0.66) | 0 | 0.4 (0.25-0.63) | 0 | 0.4 (0.24-0.67) | 0 |
| **Race** |  | 0.754 |  | 0.98 |  | 0.725 |
| NHW | 0.6 (0.42-0.87) | 0.007 | 0.58 (0.4-0.85) | 0.006 | 0.57 (0.37-0.86) | 0.008 |
| NHB | 0.85 (0.38-1.91) | 0.689 | 0.97 (0.46-2.04) | 0.933 | 1.34 (0.5-3.59) | 0.562 |
| NHAPI | 0.6 (0.26-1.4) | 0.237 | 0.56 (0.25-1.25) | 0.156 | 0.63 (0.26-1.53) | 0.309 |
| Hispanic | 0.59 (0.3-1.16) | 0.123 | 0.51 (0.25-1.03) | 0.059 | 0.55 (0.24-1.24) | 0.148 |
| Others | 1.41 (0.08-23.57) | 0.809 | 1.35 (0.24-7.62) | 0.731 | 1.41 (0.08-23.57) | 0.809 |
| **Married** |  | 0.553 |  | 0.309 |  | 0.743 |
| Yes | 0.58 (0.41-0.84) | 0.003 | 0.59 (0.41-0.85) | 0.005 | 0.67 (0.44-1.01) | 0.053 |
| No | 0.7 (0.45-1.1) | 0.119 | 0.63 (0.39-1.02) | 0.059 | 0.61 (0.37-1.01) | 0.053 |
| **Income** |  | 0.475 |  | 0.714 |  | 0.295 |
| ≤ $49999 | 0.36 (0.13-0.94) | 0.037 | 0.4 (0.16-1.03) | 0.057 | 0.18 (0.05-0.67) | 0.011 |
| $50000-74999 | 0.72 (0.49-1.05) | 0.084 | 0.69 (0.48-1.02) | 0.059 | 0.72 (0.47-1.09) | 0.119 |
| ≥ 75000 | 0.57 (0.35-0.92) | 0.022 | 0.56 (0.34-0.91) | 0.019 | 0.68 (0.4-1.18) | 0.174 |
| **Location** |  | 0.675 |  | 0.458 |  | 0.631 |
| Urban | 0.62 (0.46-0.83) | 0.002 | 0.6 (0.44-0.81) | 0.001 | 0.63 (0.45-0.89) | 0.008 |
| Rural | 0.8 (0.37-1.73) | 0.564 | 0.78 (0.35-1.75) | 0.548 | 0.86 (0.35-2.15) | 0.751 |
| **Site** |  | 0.676 |  | 0.169 |  | 0.645 |
| Head and neck | 0.86 (0.37-1.98) | 0.718 | 0.94 (0.39-2.25) | 0.888 | 0.83 (0.33-2.05) | 0.679 |
| Intracranial | 0.18 (0.01-2.93) | 0.23 | 0.17 (0.03-1.16) | 0.07 | 0.29 (0.02-4.65) | 0.381 |
| Limb | 0.45 (0.26-0.78) | 0.004 | 0.42 (0.24-0.73) | 0.002 | 0.33 (0.17-0.65) | 0.001 |
| Others | 0.71 (0.49-1.03) | 0.069 | 0.67 (0.46-0.99) | 0.043 | 0.75 (0.49-1.14) | 0.176 |
| **Grade** |  | 0.221 |  | 0.171 |  | 0.109 |
| G0/GX | 0.73 (0.48-1.09) | 0.121 | 0.71 (0.47-1.06) | 0.097 | 0.79 (0.49-1.26) | 0.321 |
| G1 | 1.81 (0.42-7.82) | 0.424 | 1.6 (0.42-6.07) | 0.491 | 2.43 (0.43-13.91) | 0.318 |
| G2 | 1.07 (0.82-1.39) | 0.642 | 0.85 (0.6-1.21) | 0.377 | 1.08 (0.77-1.51) | 0.656 |
| **Size** |  | 0.539 |  | 0.816 |  | 0.472 |
| 0-50mm | 0.6 (0.3-1.19) | 0.14 | 0.64 (0.32-1.27) | 0.204 | 0.66 (0.31-1.42) | 0.285 |
| 51-100mm | 0.71 (0.44-1.13) | 0.146 | 0.69 (0.42-1.11) | 0.126 | 0.69 (0.41-1.16) | 0.162 |
| 101-150mm | 0.45 (0.26-0.79) | 0.005 | 0.43 (0.24-0.77) | 0.004 | 0.44 (0.23-0.83) | 0.012 |
| > 150mm | 0.79 (0.42-1.49) | 0.468 | 0.8 (0.42-1.54) | 0.509 | 1.03 (0.51-2.1) | 0.928 |
| **Surgery** |  | 0.83 |  | 0.327 |  | 0.539 |
| Local excision | 0.52 (0.33-0.83) | 0.006 | 0.49 (0.3-0.8) | 0.004 | 0.55 (0.32-0.94) | 0.028 |
| Radical excision | 0.66 (0.47-0.93) | 0.019 | 0.66 (0.46-0.94) | 0.021 | 0.67 (0.45-0.99) | 0.046 |
| **Chemotherapy** |  | 0.609 |  | 0.774 |  | 0.76 |
| No | 0.64 (0.42-0.97) | 0.036 | 0.64 (0.42-0.97) | 0.037 | 0.75 (0.46-1.21) | 0.238 |
| Yes | 0.62 (0.43-0.9) | 0.013 | 0.59 (0.4-0.88) | 0.009 | 0.58 (0.38-0.89) | 0.013 |

**Supplementary table 29. Baseline of patients at stage IV stratified by chemotherapy before and after matching.**

|  | Before matching | | | After IPTW | | | After PSM | | |
| --- | --- | --- | --- | --- | --- | --- | --- | --- | --- |
|  | No | Yes | *P* value | No | Yes | *P* value | No | Yes | *P* value |
| **Patients** | 118 (100) | 131 (100) |  | 264.8 (100) | 250.7 (100) |  | 69 | 69 |  |
| **Diagnosis year** |  |  | 0.233 |  |  | 0.812 |  |  | 0.809 |
| 2000-2004 | 20 (16.9) | 24 (18.3) |  | 43.6 (16.5) | 46.4 (18.5) |  | 12 (17.4) | 15 (21.7) |  |
| 2005-2009 | 28 (23.7) | 45 (34.4) |  | 96.7 (36.5) | 75.3 (30.1) |  | 22 (31.9) | 21 (30.4) |  |
| 2010-2014 | 35 (29.7) | 33 (25.2) |  | 61.7 (23.3) | 57.5 (22.9) |  | 17 (24.6) | 19 (27.5) |  |
| 2015-2019 | 35 (29.7) | 29 (22.1) |  | 62.7 (23.7) | 71.5 (28.5) |  | 18 (26.1) | 14 (20.3) |  |
| **Age** |  |  | <0.001 |  |  | 0.677 |  |  | 0.637 |
| 00-19 years | 7 (5.93) | 24 (18.3) |  | 51.9 (19.6) | 32.8 (13.1) |  | 4 (5.8) | 7 (10.1) |  |
| 20-64 years | 76 (64.4) | 96 (73.3) |  | 166.0 (62.7) | 166.8 (66.5) |  | 54 (78.3) | 52 (75.4) |  |
| ≥ 65 years | 35 (29.7) | 11 (8.40) |  | 46.8 (17.7) | 51.2 (20.4) |  | 11 (15.9) | 10 (14.5) |  |
| **Sex** |  |  | 0.016 |  |  | 0.63 |  |  | > .99 |
| Male | 79 (66.9) | 67 (51.1) |  | 171.7 (64.8) | 152.6 (60.9) |  | 42 (60.9) | 43 (62.3) |  |
| Female | 39 (33.1) | 64 (48.9) |  | 93.1 (35.2) | 98.1 (39.1) |  | 27 (39.1) | 26 (37.7) |  |
| **Race** |  |  | 0.004 |  |  | 0.861 |  |  | 0.87 |
| NHW | 73 (61.9) | 68 (51.9) |  | 139.3 (52.6) | 135.9 (54.2) |  | 40 (58.0) | 42 (60.9) |  |
| NHB | 17 (14.4) | 13 (9.92) |  | 28.0 (10.6) | 26.6 (10.6) |  | 9 (13.0) | 7 (10.1) |  |
| NHAPI | 15 (12.7) | 13 (9.92) |  | 27.9 (10.5) | 37.0 (14.8) |  | 8 (11.6) | 11 (15.9) |  |
| Hispanic | 11 (9.32) | 36 (27.5) |  | 66.4 (25.1) | 47.2 (18.8) |  | 11 (15.9) | 8 (11.6) |  |
| Others | 2 (1.69) | 1 (0.76) |  | 3.2 (1.2) | 4.0 (1.6) |  | 1 (1.4) | 1 (1.4) |  |
| **Married** |  |  | 0.51 |  |  | 0.164 |  |  | > .99 |
| Yes | 69 (58.5) | 83 (63.4) |  | 179.2 (67.7) | 141.2 (56.3) |  | 41 (59.4) | 42 (60.9) |  |
| No | 49 (41.5) | 48 (36.6) |  | 85.6 (32.3) | 109.5 (43.7) |  | 28 (40.6) | 27 (39.1) |  |
| **Income** |  |  | 0.699 |  |  | 0.874 |  |  | 0.211 |
| ≤ $49999 | 12 (10.2) | 15 (11.5) |  | 25.1 (9.5) | 29.5 (11.8) |  | 10 (14.5) | 4 (5.8) |  |
| $50000-74999 | 72 (61.0) | 73 (55.7) |  | 150.1 (56.7) | 142.4 (56.8) |  | 38 (55.1) | 39 (56.5) |  |
| ≥ 75000 | 34 (28.8) | 43 (32.8) |  | 89.6 (33.9) | 78.9 (31.4) |  | 21 (30.4) | 26 (37.7) |  |
| **Location** |  |  | 0.801 |  |  | 0.678 |  |  | > .99 |
| Urban | 104 (88.1) | 113 (86.3) |  | 234.7 (88.6) | 217.2 (86.6) |  | 61 (88.4) | 62 (89.9) |  |
| Rural | 14 (11.9) | 18 (13.7) |  | 30.1 (11.4) | 33.5 (13.4) |  | 8 (11.6) | 7 (10.1) |  |
| **Site** |  |  | 0.258 |  |  | 0.58 |  |  | 0.2 |
| Head and neck | 21 (17.8) | 12 (9.16) |  | 40.1 (15.2) | 24.2 (9.7) |  | 10 (14.5) | 5 (7.2) |  |
| Intracranial | 3 (2.54) | 4 (3.05) |  | 4.3 (1.6) | 6.4 (2.6) |  | 3 (4.3) | 1 (1.4) |  |
| Limb | 32 (27.1) | 39 (29.8) |  | 74.9 (28.3) | 63.6 (25.4) |  | 24 (34.8) | 20 (29.0) |  |
| Others | 62 (52.5) | 76 (58.0) |  | 145.4 (54.9) | 156.5 (62.4) |  | 32 (46.4) | 43 (62.3) |  |
| **Grade** |  |  | 0.003 |  |  | 0.796 |  |  | 0.696 |
| G0/GX | 67 (56.8) | 53 (40.5) |  | 117.3 (44.3) | 123.3 (49.2) |  | 35 (50.7) | 33 (47.8) |  |
| G1 | 9 (7.63) | 4 (3.05) |  | 12.8 (4.8) | 9.9 (4.0) |  | 2 (2.9) | 4 (5.8) |  |
| G2 | 42 (35.6) | 74 (56.5) |  | 134.7 (50.9) | 117.5 (46.9) |  | 32 (46.4) | 32 (46.4) |  |
| **Size** |  |  | 0.007 |  |  | 0.03 |  |  | 0.128 |
| 0-50mm | 32 (27.1) | 18 (13.7) |  | 61.5 (23.2) | 35.4 (14.1) |  | 20 (29.0) | 9 (13.0) |  |
| 51-100mm | 45 (38.1) | 41 (31.3) |  | 123.1 (46.5) | 81.2 (32.4) |  | 22 (31.9) | 25 (36.2) |  |
| 101-150mm | 24 (20.3) | 39 (29.8) |  | 46.3 (17.5) | 68.9 (27.5) |  | 15 (21.7) | 17 (24.6) |  |
| > 150mm | 17 (14.4) | 33 (25.2) |  | 33.9 (12.8) | 65.3 (26.1) |  | 12 (17.4) | 18 (26.1) |  |
| **Surgery** |  |  | 0.248 |  |  | 0.076 |  |  | 0.472 |
| No | 35 (29.7) | 49 (37.4) |  | 61.9 (23.4) | 89.5 (35.7) |  | 21 (30.4) | 26 (37.7) |  |
| Yes | 83 (70.3) | 82 (62.6) |  | 202.9 (76.6) | 161.2 (64.3) |  | 48 (69.6) | 43 (62.3) |  |
| **Radiotherapy** |  |  |  |  |  | 0.712 |  |  | 0.864 |
| No | 62 (52.5) | 69 (52.7) |  | 151.2 (57.1) | 134.9 (53.8) |  | 37 (53.6) | 39 (56.5) |  |
| Yes | 56 (47.5) | 62 (47.3) |  | 113.6 (42.9) | 115.8 (46.2) |  | 32 (46.4) | 30 (43.5) |  |

**Supplementary table 30. Analysis for chemotherapy utilization among patients at stage IV.**

|  | Uni-regression | | Multi-regression | | Matching | |
| --- | --- | --- | --- | --- | --- | --- |
|  | OR | *P* value | OR | *P* value | OR | *P* value |
| **Diagnosis year** |  |  |  |  |  |  |
| 2000-2004 | Reference | - |  |  | Reference | - |
| 2005-2009 | 1.34 (0.63-2.86) | 0.45 |  |  | 2.02 (0.84-4.88) | 0.117 |
| 2010-2014 | 0.79 (0.37-1.68) | 0.534 |  |  | 1.06 (0.45-2.51) | 0.891 |
| 2015-2019 | 0.69 (0.32-1.49) | 0.346 |  |  | 0.67 (0.27-1.67) | 0.391 |
| **Age** |  |  |  |  |  |  |
| 00-19 years | Reference | - | Reference | - | Reference | - |
| 20-64 years | 0.37 (0.15-0.9) | 0.029 | 0.3 (0.1-0.91) | 0.034 | 0.28 (0.09-0.88) | 0.029 |
| ≥ 65 years | 0.09 (0.03-0.27) | < .001 | 0.08 (0.02-0.29) | < .001 | 0.07 (0.02-0.26) | < .001 |
| **Sex** |  |  |  |  |  |  |
| Male | Reference | - | Reference | - | Reference | - |
| Female | 1.93 (1.16-3.24) | 0.012 | 2.08 (1.15-3.78) | 0.016 | 2.12 (1.18-3.84) | 0.013 |
| **Race** |  |  |  |  |  |  |
| NHW | Reference | - | Reference | - | Reference | - |
| NHB | 0.82 (0.37-1.82) | 0.626 | 0.47 (0.18-1.22) | 0.121 | 0.56 (0.23-1.36) | 0.201 |
| NHAPI | 0.93 (0.41-2.1) | 0.862 | 0.67 (0.27-1.68) | 0.392 | 0.88 (0.34-2.27) | 0.798 |
| Hispanic | 3.51 (1.66-7.45) | 0.001 | 2.55 (1.1-5.92) | 0.029 | 3.65 (1.55-8.55) | 0.003 |
| Others | 0.54 (0.05-6.06) | 0.615 | 0.17 (0.01-2.25) | 0.181 | 0.27 (0.02-3.51) | 0.319 |
| **Married** |  |  |  |  |  |  |
| Yes | Reference | - |  |  |  |  |
| No | 0.81 (0.49-1.36) | 0.43 |  |  |  |  |
| **Income** |  |  |  |  |  |  |
| ≤ $49999 | Reference | - |  |  |  |  |
| $50000-74999 | 0.81 (0.36-1.85) | 0.619 |  |  |  |  |
| ≥ 75000 | 1.01 (0.42-2.45) | 0.979 |  |  |  |  |
| **Location** |  |  |  |  |  |  |
| Urban | Reference | - |  |  |  |  |
| Rural | 1.18 (0.56-2.5) | 0.659 |  |  |  |  |
| **Site** |  |  |  |  |  |  |
| Head and neck | Reference | - |  |  |  |  |
| Intracranial | 2.33 (0.45-12.23) | 0.316 |  |  |  |  |
| Limb | 2.13 (0.91-4.99) | 0.081 |  |  |  |  |
| Others | 2.15 (0.98-4.7) | 0.057 |  |  |  |  |
| **Grade** |  |  |  |  |  |  |
| G0/GX | Reference | - | Reference | - | Reference | - |
| G1 | 0.56 (0.16-1.93) | 0.359 | 0.35 (0.09-1.37) | 0.131 | 0.41 (0.1-1.66) | 0.213 |
| G2 | 2.23 (1.32-3.76) | 0.003 | 1.98 (1.09-3.59) | 0.024 | 2.28 (1.25-4.16) | 0.007 |
| **Size** |  |  |  |  |  |  |
| 0-50mm | Reference | - | Reference | - |  |  |
| 51-100mm | 1.62 (0.79-3.31) | 0.187 | 1.56 (0.7-3.43) | 0.274 |  |  |
| 101-150mm | 2.89 (1.34-6.24) | 0.007 | 2.18 (0.93-5.09) | 0.072 |  |  |
| > 150mm | 3.45 (1.52-7.85) | 0.003 | 2.87 (1.11-7.42) | 0.03 |  |  |
| **Surgery** |  |  |  |  |  |  |
| No | Reference | - |  |  |  |  |
| Yes | 0.71 (0.42-1.2) | 0.198 |  |  |  |  |
| **Radiotherapy** |  |  |  |  |  |  |
| No | Reference | - |  |  |  |  |
| Yes | 0.99 (0.6-1.64) | 0.984 |  |  |  |  |

**Supplementary table 31. Cox regression of overall survival for patients at stage I-III stratified by chemotherapy.**

|  | Before matching | | | | After IPTW | | | | After PSM | | | |
| --- | --- | --- | --- | --- | --- | --- | --- | --- | --- | --- | --- | --- |
|  | Uni-regression | | Multi-regression | | Uni-regression | | Multi-regression | | Uni-regression | | Multi-regression | |
|  | HR | *P* value | HR | *P* value | HR | *P* value | HR | *P* value | HR | *P* value | HR | *P* value |
| **Diagnosis year** |  |  |  |  |  |  |  |  |  |  |  |  |
| 2000-2004 | Reference | - |  |  | Reference | - | Reference | - | Reference | - | Reference | - |
| 2005-2009 | 0.97 (0.64-1.45) | 0.873 | 0.83 (0.55-1.27) | 0.398 | 1.04 (0.61-1.78) | 0.892 | 0.89 (0.56-1.44) | 0.645 | 0.98 (0.58-1.65) | 0.944 | 0.81 (0.47-1.4) | 0.454 |
| 2010-2014 | 1.38 (0.92-2.08) | 0.118 | 1.14 (0.74-1.74) | 0.554 | 1.38 (0.86-2.22) | 0.176 | 1.15 (0.7-1.88) | 0.589 | 1.47 (0.87-2.5) | 0.153 | 1.26 (0.72-2.21) | 0.416 |
| 2015-2019 | 1.18 (0.76-1.83) | 0.458 | 1.12 (0.71-1.77) | 0.637 | 1.33 (0.81-2.19) | 0.265 | 1.36 (0.82-2.27) | 0.231 | 1.17 (0.64-2.12) | 0.616 | 0.79 (0.41-1.5) | 0.467 |
| **Age** |  |  |  |  |  |  |  |  |  |  |  |  |
| 00-19 years | Reference | - | Reference | - | Reference | - |  |  | Reference | - |  |  |
| 20-64 years | 1.66 (1.05-2.63) | 0.031 | 1.63 (1.02-2.61) | 0.041 | 1.34 (0.63-2.85) | 0.453 |  |  | 1.65 (0.8-3.41) | 0.176 |  |  |
| ≥ 65 years | 2 (1.18-3.39) | 0.01 | 2.34 (1.3-4.21) | 0.004 | 1.93 (0.86-4.33) | 0.109 |  |  | 1.82 (0.8-4.13) | 0.153 |  |  |
| **Sex** |  |  |  |  |  |  |  |  |  |  |  |  |
| Male | Reference | - |  |  | Reference | - |  |  | Reference | - |  |  |
| Female | 0.87 (0.66-1.15) | 0.332 |  |  | 0.82 (0.58-1.16) | 0.265 |  |  | 1 (0.69-1.45) | 0.989 |  |  |
| **Race** |  |  |  |  |  |  |  |  |  |  |  |  |
| NHW | Reference | - |  |  | Reference | - |  |  |  |  |  |  |
| NHB | 1.17 (0.77-1.79) | 0.467 |  |  | 1.36 (0.89-2.08) | 0.15 |  |  | 1.37 (0.77-2.44) | 0.279 |  |  |
| NHAPI | 0.97 (0.62-1.51) | 0.886 |  |  | 1.23 (0.67-2.24) | 0.506 |  |  | 0.93 (0.53-1.62) | 0.789 |  |  |
| Hispanic | 0.92 (0.63-1.35) | 0.678 |  |  | 1.03 (0.64-1.66) | 0.898 |  |  | 0.68 (0.37-1.26) | 0.221 |  |  |
| Others | 0.7 (0.17-2.84) | 0.62 |  |  | 0.83 (0.3-2.34) | 0.728 |  |  | 1.7 (0.42-6.97) | 0.459 |  |  |
| **Married** |  |  |  |  |  |  |  |  |  |  |  |  |
| Yes | Reference | - |  |  | Reference | - |  |  | Reference | - |  |  |
| No | 1.05 (0.79-1.39) | 0.732 |  |  | 1.12 (0.8-1.57) | 0.516 |  |  | 1.03 (0.71-1.49) | 0.884 |  |  |
| **Income** |  |  |  |  |  |  |  |  |  |  |  |  |
| ≤ $49999 | Reference | - |  |  | Reference | - |  |  | Reference | - |  |  |
| $50000-74999 | 0.92 (0.57-1.46) | 0.712 |  |  | 0.84 (0.52-1.35) | 0.461 |  |  | 0.58 (0.32-1.06) | 0.079 |  |  |
| ≥ 75000 | 1.07 (0.65-1.74) | 0.798 |  |  | 1.09 (0.69-1.73) | 0.712 |  |  | 0.77 (0.41-1.43) | 0.405 |  |  |
| **Location** |  |  |  |  |  |  |  |  |  |  |  |  |
| Urban | Reference | - |  |  | Reference | - |  |  | Reference | - |  |  |
| Rural | 0.94 (0.62-1.42) | 0.772 |  |  | 0.83 (0.51-1.38) | 0.479 |  |  | 0.98 (0.55-1.75) | 0.95 |  |  |
| **Site** |  |  |  |  |  |  |  |  |  |  |  |  |
| Head and neck | Reference | - |  |  | Reference | - |  |  | Reference | - |  |  |
| Intracranial | 0.59 (0.2-1.72) | 0.337 |  |  | 0.8 (0.29-2.21) | 0.662 |  |  | 0.76 (0.21-2.74) | 0.677 |  |  |
| Limb | 1.38 (0.84-2.24) | 0.201 |  |  | 1.73 (0.89-3.36) | 0.107 |  |  | 1.48 (0.76-2.88) | 0.255 |  |  |
| Others | 1.52 (0.96-2.4) | 0.074 |  |  | 1.91 (0.99-3.7) | 0.054 |  |  | 1.53 (0.81-2.91) | 0.193 |  |  |
| **Grade** |  |  |  |  |  |  |  |  |  |  |  |  |
| G0/GX | Reference | - |  |  | Reference | - | Reference | - | Reference | - |  |  |
| G1 | 0.71 (0.53-0.95) | 0.023 |  |  | 0.51 (0.27-0.97) | 0.04 | 0.47 (0.22-1.01) | 0.053 | 0.75 (0.3-1.89) | 0.547 |  |  |
| G2 | 1.69 (1.4-2.04) | < .001 |  |  | 1.29 (0.9-1.84) | 0.16 | 1.32 (0.96-1.83) | 0.091 | 1.44 (0.99-2.09) | 0.058 |  |  |
| **Size** |  |  |  |  |  |  |  |  |  |  |  |  |
| 0-50mm | Reference | - | Reference | - | Reference | - | Reference | - | Reference | - | Reference | - |
| 51-100mm | 1.93 (1.28-2.91) | 0.002 | 2.09 (1.36-3.2) | 0.001 | 2.12 (1.3-3.47) | 0.003 | 2.3 (1.4-3.76) | 0.001 | 2.28 (1.33-3.9) | 0.003 | 3.48 (1.92-6.28) | 0 |
| 101-150mm | 1.78 (1.15-2.75) | 0.009 | 2.24 (1.4-3.6) | 0.001 | 1.98 (1.27-3.09) | 0.003 | 2.57 (1.5-4.4) | 0.001 | 2.04 (1.13-3.67) | 0.018 | 3.25 (1.7-6.23) | 0 |
| > 150mm | 1.91 (1.22-3.01) | 0.005 | 2.12 (1.33-3.4) | 0.002 | 2.41 (1.47-3.95) | 0.001 | 2.46 (1.44-4.23) | 0.001 | 2.25 (1.25-4.04) | 0.007 | 2.8 (1.48-5.28) | 0.001 |
| **Surgery** |  |  |  |  |  |  |  |  |  |  |  |  |
| No | Reference | - | Reference | - | Reference | - | Reference | - | Reference | - | Reference | - |
| Yes | 0.66 (0.49-0.87) | 0.004 | 0.61 (0.45-0.82) | 0.001 | 0.67 (0.47-0.95) | 0.023 | 0.61 (0.43-0.86) | 0.004 | 0.66 (0.45-0.97) | 0.033 | 0.5 (0.33-0.76) | 0.001 |
| **Chemotherapy** |  |  |  |  |  |  |  |  |  |  |  |  |
| No | Reference | - | Reference | - | Reference | - | Reference | - | Reference | - |  |  |
| Yes | 0.98 (0.74-1.3) | 0.882 | 0.91 (0.65-1.26) | 0.55 | 1.07 (0.75-1.52) | 0.729 | 0.74 (0.52-1.05) | 0.089 | 0.89 (0.62-1.29) | 0.547 | 0.52 (0.34-0.8) | 0.002 |
| **Radiotherapy** |  |  |  |  |  |  |  |  |  |  |  |  |
| No | Reference | - | Reference | - | Reference | - | Reference | - | Reference | - | Reference | - |
| Yes | 0.63 (0.48-0.83) | 0.001 | 0.57 (0.42-0.77) | < .001 | 0.55 (0.39-0.77) | 0.001 | 0.45 (0.31-0.66) | 0 | 0.58 (0.4-0.84) | 0.004 | 0.51 (0.33-0.77) | 0.002 |

**Supplementary table 32. Restricted mean survival time for patients at stage IV stratified by chemotherapy.**

|  | **Unadjusted** | | **PSM** | |
| --- | --- | --- | --- | --- |
|  | **No** | **Yes** | **No** | **Yes** |
| **Restricted at 1 year** | | | | |
| RSMT, month | 7.24 (6.4-8.08) | 9.23 (8.63-9.84) | 6.46 (5.36-7.55) | 8.96 (8.1-9.82) |
| RMST difference | 1.99 (0.96-3.03) (*P* < .001) | | 2.5 (1.11-3.89) (*P* < .001) | |
| **Restricted at 3 years** | | | | |
| RSMT, month | 15.2 (12.49-17.91) | 14.9 (12.99-16.8) | 12.64 (9.31-15.98) | 14.03 (11.47-16.58) |
| RMST difference | -0.31 (-3.62- 3) (*P* = 0.856) | | 1.38 (-2.82 - 5.59) (*P* = 0.519) | |
| **Restricted at 5 years** | | | | |
| RSMT, month | 21.08 (16.62-25.54) | 18.56 (15.31-21.81) | 17.24 (11.77-22.71) | 17.46 (13.11-21.81) |
| RMST difference | -2.52 (-8.04-3.01) (P = 0.372) | | 0.22 (-6.77 -7.21) (*P* = 0.951) | |
| **Restricted at 10 years** | | | | |
| RSMT, month | 31.46 (23.18-39.74) | 26.11 (19.63-32.59) | 26.74 (16.22-37.26) | 22.95 (14.96-30.95) |
| RMST difference | -5.35 (-15.86-5.16) (*P* = 0.319) | | -3.79 (-16.99 - 9.42) (*P* = 0.574) | |

**Supplementary table 33. Subgroup analysis for patients at stage IV with chemotherapy.**

|  | Before matching | | After IPTW | | After PSM | |
| --- | --- | --- | --- | --- | --- | --- |
|  | Hazard ratio | *P* value | Hazard ratio | *P* value | Hazard ratio | *P* value |
| **Diagnosis year** |  | 0.417 |  | 0.272 |  | 0.236 |
| 2000-2004 | 1.11 (0.58-2.13) | 0.753 | 1.46 (0.68-3.14) | 0.339 | 0.81 (0.35-1.86) | 0.613 |
| 2005-2009 | 0.97 (0.57-1.64) | 0.911 | 0.94 (0.47-1.89) | 0.873 | 1.21 (0.63-2.34) | 0.571 |
| 2010-2014 | 0.87 (0.52-1.45) | 0.594 | 0.81 (0.49-1.34) | 0.405 | 0.78 (0.4-1.54) | 0.479 |
| 2015-2019 | 1.13 (0.62-2.07) | 0.686 | 1.28 (0.68-2.41) | 0.436 | 0.82 (0.34-1.99) | 0.667 |
| **Age** |  | 0.377 |  | 0.214 |  | 0.417 |
| 00-19 years | 2.02 (0.59-6.91) | 0.262 | 0.95 (0.38-2.38) | 0.911 | 0.51 (0.12-2.17) | 0.361 |
| 20-64 years | 0.94 (0.67-1.33) | 0.739 | 0.97 (0.66-1.43) | 0.875 | 0.88 (0.57-1.33) | 0.537 |
| ≥ 65 years | 1.9 (0.93-3.9) | 0.079 | 1.99 (1.06-3.75) | 0.033 | 1.89 (0.74-4.85) | 0.185 |
| **Sex** |  | 0.993 |  | 0.622 |  | 0.666 |
| Male | 0.97 (0.67-1.4) | 0.867 | 0.98 (0.64-1.5) | 0.924 | 0.87 (0.54-1.41) | 0.581 |
| Female | 1.04 (0.67-1.62) | 0.852 | 1.26 (0.71-2.22) | 0.427 | 1.1 (0.6-1.99) | 0.767 |
| **Race** |  | 0.351 |  | 0.093 |  | 0.718 |
| NHW | 0.86 (0.59-1.24) | 0.412 | 1.04 (0.65-1.66) | 0.863 | 1.04 (0.64-1.67) | 0.885 |
| NHB | 1.56 (0.67-3.62) | 0.304 | 1.38 (0.63-3.05) | 0.425 | 0.55 (0.19-1.58) | 0.264 |
| NHAPI | 0.91 (0.4-2.08) | 0.824 | 1.1 (0.47-2.6) | 0.822 | 0.81 (0.3-2.2) | 0.676 |
| Hispanic | 1.3 (0.56-3.03) | 0.543 | 0.92 (0.41-2.07) | 0.848 | 1.06 (0.32-3.5) | 0.923 |
| Others | - | - | - | - | - | - |
| **Married** |  | 0.902 |  | 0.345 |  | 0.089 |
| Yes | 0.94 (0.66-1.35) | 0.749 | 0.88 (0.55-1.41) | 0.608 | 0.68 (0.42-1.1) | 0.113 |
| No | 1.04 (0.67-1.63) | 0.851 | 1.35 (0.83-2.21) | 0.229 | 1.47 (0.81-2.67) | 0.205 |
| **Income** |  | 0.724 |  | 0.268 |  | 0.728 |
| ≤ $49999 | 0.66 (0.28-1.57) | 0.346 | 0.81 (0.35-1.88) | 0.628 | 1.03 (0.3-3.55) | 0.966 |
| $50000-74999 | 1.03 (0.71-1.5) | 0.866 | 1.25 (0.78-2) | 0.356 | 0.91 (0.55-1.52) | 0.731 |
| ≥$75000 | 0.96 (0.59-1.58) | 0.886 | 0.77 (0.44-1.38) | 0.384 | 0.98 (0.52-1.86) | 0.951 |
| **Location** |  | 0.88 |  | 0.623 |  | 0.625 |
| Urban | 1 (0.74-1.34) | 0.976 | 1.03 (0.7-1.5) | 0.888 | 0.92 (0.62-1.36) | 0.663 |
| Rural | 0.89 (0.41-1.96) | 0.78 | 1.42 (0.56-3.6) | 0.462 | 1.08 (0.35-3.33) | 0.896 |
| **Site** |  | 0.974 |  | 0.722 |  | 0.727 |
| Head and neck | 1.16 (0.5-2.73) | 0.728 | 1.64 (0.61-4.4) | 0.33 | 1.28 (0.36-4.53) | 0.708 |
| Intracranial | 0.71 (0.1-5.1) | 0.731 | 0.86 (0.18-4.03) | 0.844 | 2.45 (0.15-39.72) | 0.529 |
| Limb | 0.88 (0.53-1.48) | 0.635 | 0.81 (0.49-1.36) | 0.432 | 0.69 (0.37-1.31) | 0.262 |
| Others | 0.97 (0.66-1.4) | 0.853 | 1.07 (0.67-1.69) | 0.782 | 0.91 (0.55-1.51) | 0.712 |
| **Grade** |  | 0.03 |  | 0.188 |  | 0.063 |
| G0/GX | 1.16 (0.77-1.75) | 0.487 | 1.49 (0.89-2.49) | 0.128 | 1.32 (0.76-2.28) | 0.328 |
| G1 | 1.7 (0.4-7.33) | 0.474 | 1.72 (0.39-7.46) | 0.472 | 0.7 (0.11-4.42) | 0.708 |
| G2 | 0.6 (0.4-0.9) | 0.014 | 0.72 (0.45-1.16) | 0.175 | 0.66 (0.39-1.11) | 0.116 |
| **Size** |  | 0.021 |  | 0.151 |  | 0.004 |
| 0-50mm | 2.03 (1.02-4.01) | 0.043 | 2.33 (1.16-4.69) | 0.017 | 2.58 (1.02-6.48) | 0.044 |
| 51-100mm | 0.75 (0.47-1.2) | 0.23 | 0.93 (0.53-1.64) | 0.808 | 0.7 (0.38-1.29) | 0.247 |
| 101-150mm | 0.7 (0.39-1.24) | 0.219 | 0.63 (0.32-1.25) | 0.185 | 0.41 (0.19-0.88) | 0.022 |
| > 150mm | 0.66 (0.35-1.24) | 0.199 | 0.78 (0.4-1.54) | 0.477 | 0.44 (0.19-1) | 0.049 |
| **Surgery** |  | 0.156 |  | 0.397 |  | 0.085 |
| Local excision | 0.7 (0.43-1.12) | 0.132 | 0.68 (0.37-1.23) | 0.203 | 0.56 (0.3-1.05) | 0.07 |
| Radical excision | 1.07 (0.75-1.51) | 0.723 | 1.16 (0.74-1.83) | 0.517 | 1.11 (0.7-1.76) | 0.665 |
| **Radiotherapy** |  | 0.511 |  | 0.337 |  | 0.953 |
| No | 0.83 (0.57-1.22) | 0.339 | 0.82 (0.54-1.25) | 0.351 | 0.72 (0.44-1.18) | 0.192 |
| Yes | 1.06 (0.7-1.6) | 0.792 | 1.41 (0.84-2.36) | 0.19 | 1.12 (0.64-1.96) | 0.701 |

**Supplementary table 34. Baseline of training and validation cohorts in the nomogram for survival of primary malignant peripheral nerve sheath tumor.**

| Characteristics | Training | Validation | P value |
| --- | --- | --- | --- |
| No. | 561 | 187 |  |
| Censor, n (%) |  |  | 0.768 |
| Alive | 275 (36.8) | 94 (12.6) |  |
| Death | 286 (38.2) | 93 (12.4) |  |
| OS, median (IQR) | 37 (15, 94) | 34 (14, 100) | 0.898 |
| Sex, n (%) |  |  | 0.310 |
| Male | 303 (54.0) | 93 (49.7) |  |
| Female | 258 (46.0) | 94 (50.3) |  |
| Age, n (%) |  |  | 0.888 |
| 00-19 | 67 (11.9) | 20 (10.7) |  |
| 20-39 | 187 (33.3) | 67 (35.8) |  |
| 40-59 | 194 (34.6) | 61 (32.6) |  |
| ≥ 60 | 113 (20.1) | 39 (20.9) |  |
| Race, n (%) |  |  | 0.095 |
| NHW | 320 (57.0) | 98 (52.4) |  |
| NHB | 91 (16.2) | 23 (12.3) |  |
| NHAPI | 45 (8.0) | 16 (8.6) |  |
| Hispanic | 105 (18.7) | 50 (26.7) |  |
| Income, n (%) |  |  | 0.470 |
| <50000 | 81 (14.4) | 21 (11.2) |  |
| 50000-74999 | 316 (56.3) | 113 (60.4) |  |
| >75000 | 164 (29.2) | 53 (28.3) |  |
| Location, n (%) |  |  | 0.089 |
| Urban | 494 (88.1) | 173 (92.5) |  |
| Rural | 67 (11.9) | 14 (7.5) |  |
| Size, n (%) |  |  | 0.637 |
| 0-49 mm | 157 (28) | 49 (26.2) |  |
| ≥50 mm | 404 (72) | 138 (73.8) |  |
| Site, n (%) |  |  | 0.325 |
| Head and neck | 24 (4.3) | 5 (2.7) |  |
| Others | 537 (95.7) | 182 (97.3) |  |
| Grade, n (%) |  |  | 0.987 |
| I | 43 (7.7) | 15 (8.0) |  |
| II | 123 (21.9) | 41 (21.9) |  |
| III-IV | 395 (70.4) | 131 (70.1) |  |
| Stage, n (%) |  |  | 0.951 |
| Localize | 345 (61.5) | 114 (61) |  |
| Regional | 150 (26.7) | 52 (27.8) |  |
| Distant | 66 (11.8) | 21 (11.2) |  |
| Pathology, n (%) |  |  | 0.115 |
| Triton | 35 (6.2) | 6 (3.2) |  |
| Others | 526 (93.8) | 181 (96.8) |  |
| Total number of tumors, n (%) |  |  | 1.000 |
| 1 | 504 (89.8) | 168 (89.8) |  |
| ≥ 1 | 57 (10.2) | 19 (10.2) |  |

Abbreviations: NHW, non-Hispanic White; NHB, non-Hispanic Black; NHAPI, non-Hispanic Asian and Pacific Islander.

**Supplementary table 35. Univariate and multivariate analysis for malignant peripheral nerve sheath tumor.**

| **Variable** | **Total**  **(No.)** | **Univariate analysis** | | **Multivariate analysis** | |
| --- | --- | --- | --- | --- | --- |
|  |  | **Hazard ratio (95% CI)** | ***P* value** | **Hazard ratio (95% CI)** | ***P* value** |
| **Sex** |  |  | 0.787 |  |  |
| Male | 303 | Reference |  |  |  |
| Female | 258 | 0.969 (0.768 - 1.222) | 0.787 |  |  |
| **Age** |  |  | 0.025 |  |  |
| 00-19 | 67 | Reference |  | Reference |  |
| 20-39 | 187 | 1.499 (0.981 - 2.290) | 0.061 | 1.602 (1.045 - 2.456) | 0.031 |
| 40-59 | 194 | 1.102 (0.716 - 1.695) | 0.658 | 1.301 (0.838 - 2.020) | 0.241 |
| ≥60 | 113 | 1.606 (1.027 - 2.511) | 0.038 | 2.045 (1.300 - 3.217) | 0.002 |
| **Race** |  |  | 0.228 |  |  |
| NHW ^a^ | 320 | Reference |  |  |  |
| NHB ^b^ | 91 | 1.177 (0.851 - 1.627) | 0.326 |  |  |
| NHAPI ^c^ | 45 | 1.152 (0.749 - 1.772) | 0.518 |  |  |
| Hispanic | 105 | 1.367 (1.014 - 1.843) | 0.040 |  |  |
| **Income** |  |  | 0.505 |  |  |
| < 50000 | 81 | Reference |  |  |  |
| 50000-74999 | 316 | 0.820 (0.587 - 1.146) | 0.245 |  |  |
| ≥75000 | 164 | 0.883 (0.613 - 1.271) | 0.503 |  |  |
| **Location** |  |  | 0.875 |  |  |
| Urban | 494 | Reference |  |  |  |
| Rural | 67 | 1.029 (0.719 - 1.473) | 0.874 |  |  |
| **Size** |  |  | < 0.001 |  |  |
| 0-49 mm | 157 | Reference |  | Reference |  |
| ≥50 mm | 404 | 2.835 (2.061 - 3.901) | < 0.001 | 2.110 (1.523 - 2.923) | < 0.001 |
| **Site** | 561 |  | 0.932 |  |  |
| Head and neck | 24 | Reference |  |  |  |
| Others | 537 | 0.976 (0.560 - 1.703) | 0.932 |  |  |
| **Grade** |  |  | < 0.001 |  |  |
| I | 43 | Reference |  | Reference |  |
| II | 123 | 2.653 (1.129 - 6.233) | 0.025 | 2.885 (1.224 - 6.799) | 0.015 |
| III-IV | 395 | 6.181 (2.748 - 13.902) | < 0.001 | 5.001 (2.211 - 11.309) | < 0.001 |
| **Stage** |  |  | < 0.001 |  |  |
| Localize | 345 | Reference |  | Reference |  |
| Regional | 150 | 1.843 (1.415 - 2.402) | < 0.001 | 1.663 (1.274 - 2.173) | < 0.001 |
| Distant | 66 | 4.593 (3.354 - 6.289) | < 0.001 | 3.737 (2.686 - 5.197) | < 0.001 |
| **Pathology** |  |  | 0.007 |  |  |
| Others | 526 | Reference |  | Reference |  |
| Triton tumors | 35 | 1.893 (1.235 - 2.903) | 0.003 | 1.252 (0.810 - 1.934) | 0.312 |
| **Number of tumors** |  |  | 0.866 |  |  |
| 1 | 504 | Reference |  |  |  |
| ≥1 | 57 | 1.031 (0.721 - 1.476) | 0.865 |  |  |

Abbreviations: a. NHW, non-Hispanic White; b. NHB, non-Hispanic Black; c. NHAPI, non-Hispanic Asian and Pacific Islander.

Note: The COX regression was performed in the development cohort. Those with P < .05 in univariate was used for multivariate analysis while sex was used for adjustment.

**Supplementary table 36. Characteristics of survivors diagnosed with primary malignant peripheral nerve sheath tumor in the SEER 17 registries.**

| Characteristics | Survivors with MPNST ^a^ | Patients with MPNST ^b^ |
| --- | --- | --- |
| Overall | 1624 | 2123 |
| Sex, n (%) |  |  |
| Male | 866 (53.3) | 1148 (54.1) |
| Female | 758 (46.7) | 975 (45.9) |
| Age, n (%) |  |  |
| 00-19 | 184 (11.3) | 233 (11.0) |
| 20-39 | 502 (30.9) | 651 (30.7) |
| 40-59 | 561 (34.5) | 705 (33.2) |
| ≥ 60 | 377 (23.2) | 534 (25.2) |
| Race, n (%) |  |  |
| NHW | 922 (56.8) | 1233 (58.1) |
| NHB | 236 (14.5) | 304 (14.3) |
| NHAPI | 135 (8.3) | 182 (8.6) |
| Hispanic | 302 (18.6) | 381 (17.9) |
| Latency, n (%) |  |  |
| 2-11 months ^c^ | 1624 (100.0) | 2123 (100.0) |
| 12-59 months | 1355 (83.4) | 1599 (75.3) |
| 60-119 months | 731 (45.0) | 857 (40.4) |
| ≥ 120 months | 439 (27.0) | 514 (24.2) |
| Receipt of radiotherapy, n (%) |  |  |
| No | 871 (53.6) | 1208 (56.9) |
| Yes | 753 (43.4) | 915 (43.1) |
| Receipt of chemotherapy, n (%) |  |  |
| No | 1290 (79.4) | 1626 (76.6) |
| Yes | 334 (20.6) | 497 (23.4) |

**Supplementary table 37. The risk of developing SPC among survivors diagnosed with primary malignant peripheral nerve sheath tumor during 2000-2019 in 17 SEER registers.**

| SPC type | Observed SPCs,  No. (%) | Expected SPCs,  No. | SIR (95% CI) | Absolute excess incidence per 10,000 person-year | Mean age at SPC diagnosis, yr |
| --- | --- | --- | --- | --- | --- |
| Total | 193 (100) | 74.77 | 2.58 (2.23 to 2.97) | 119.6 (92.05 to 147.14) | 58.47 |
| Total (excluded MPNST) | 154 (79.79) | 74.77 | 2.06 (1.74 to 2.41) | 80.15 (55.55 to 104.75) | .. |
| Head and Neck | 5 (2.59) | 2.61 | 1.92 (0.62 to 4.47) | 2.42 (-2.02 to 6.85) | 60.72 |
| Esophagus | 0 (0) | 0.78 | 0 (0 to 4.76) | -0.78 (-0.78 to -0.78) | .. |
| Stomach | 4 (2.07) | 1.25 | 3.2 (0.87 to 8.2) | 2.78 (-1.18 to 6.75) | 76.56 |
| Small Intestine | 5 (2.59) | 0.38 | 13.21 (4.29 to 30.82) | 4.68 (0.24 to 9.11) | 53.3 |
| Colon and Rectum | 7 (3.63) | 6.83 | 1.03 (0.41 to 2.11) | 0.17 (-5.07 to 5.42) | 76.39 |
| Anus, Anal Canal and Anorectum | 0 (0) | 0.3 | 0 (0 to 12.42) | -0.3 (-0.3 to -0.3) | .. |
| Liver and intrahepatic bile duct | 1 (0.52) | 1.54 | 0.65 (0.02 to 3.62) | -0.55 (-2.53 to 1.44) | 56.34 |
| Gallbladder and other biliary | 1 (0.52) | 0.5 | 1.99 (0.05 to 11.1) | 0.5 (-1.48 to 2.49) | 45.42 |
| Pancreas | 2 (1.04) | 2.14 | 0.94 (0.11 to 3.38) | -0.14 (-2.94 to 2.66) | 77.22 |
| Retroperitoneum, peritoneum and other digestive system | 1 (0.52) | 0.25 | 3.95 (0.1 to 22.03) | 0.76 (-1.23 to 2.74) | 72.76 |
| Lung and Bronchus | 13 (6.74) | 9.37 | 1.39 (0.74 to 2.37) | 3.68 (-3.47 to 10.83) | 57.58 |
| Pleura, trachea and other respiratory | 3 (1.55) | 0.03 | 111.99 (23.09 to 327.27) | 3.01 (-0.43 to 6.44) | 41.36 |
| Bones and Joints | 2 (1.04) | 0.11 | 18.74 (2.27 to 67.69) | 1.92 (-0.89 to 4.72) | 67.72 |
| Soft Tissue including Heart | 52 (26.94) | 0.49 | 105.17 (78.54 to 137.91) | 52.1 (37.8 to 66.39) | 47.88 |
| Soft Tissue including Heart (excluded MPNST) | 13 (6.73) | 0.49 | 26.53 (14.13 to 45.37) | 12.65 (5.5 to 19.8) | 68.86 |
| Melanoma of the Skin | 20 (10.36) | 3.71 | 5.39 (3.29 to 8.32) | 16.47 (7.61 to 25.34) | 62.9 |
| Breast | 14 (7.25) | 9.91 | 1.41 (0.77 to 2.37) | 4.14 (-3.28 to 11.55) | .. |
| Cervix Uteri | 0 (0) | 0.51 | 0 (0 to 7.18) | -1.08 (-1.08 to -1.08) | 47.13 |
| Corpus and Uterus, NOS | 2 (1.04) | 2.1 | 0.95 (0.12 to 3.44) | -0.21 (-6.06 to 5.64) | 54.58 |
| Ovary | 1 (0.52) | 0.88 | 1.14 (0.03 to 6.36) | 0.26 (-3.87 to 4.39) | .. |
| Vagina, vulva and other female genital organs | 0 (0) | 0.36 | 0 (0 to 10.29) | -0.76 (-0.76 to -0.76) | 70.5 |
| Prostate | 10 (5.18) | 11.5 | 0.87 (0.42 to 1.6) | -2.91 (-14.96 to 9.14) | .. |
| Testis | 0 (0) | 0.31 | 0 (0 to 11.94) | -0.6 (-0.6 to -0.6) | .. |
| Penis and other male genital organs | 0 (0) | 0.1 | 0 (0 to 38.69) | -0.19 (-0.19 to -0.19) | 67.09 |
| Urinary Bladder | 4 (2.07) | 3.53 | 1.13 (0.31 to 2.9) | 0.47 (-3.49 to 4.44) | 68.24 |
| Kidney and Renal Pelvis | 7 (3.63) | 2.65 | 2.64 (1.06 to 5.44) | 4.4 (-0.85 to 9.64) | .. |
| Ureter and other urinary organs | 0 (0) | 0.16 | 0 (0 to 23.57) | -0.16 (-0.16 to -0.16) | .. |
| Eye and Orbit | 0 (0) | 0.12 | 0 (0 to 30.47) | -0.12 (-0.12 to -0.12) | 51.64 |
| Brain and Other Nervous System | 8 (4.15) | 0.87 | 9.17 (3.96 to 18.07) | 7.21 (1.6 to 12.82) | 45.31 |
| Thyroid | 9 (4.66) | 1.76 | 5.12 (2.34 to 9.72) | 7.33 (1.38 to 13.27) | .. |
| Other endocrine system | 0 (0) | 0.1 | 0 (0 to 38.25) | -0.1 (-0.1 to -0.1) | .. |
| Hodgkin Lymphoma | 0 (0) | 0.3 | 0 (0 to 12.15) | -0.31 (-0.31 to -0.31) | 66.07 |
| Non-Hodgkin Lymphoma | 6 (3.11) | 3.94 | 1.52 (0.56 to 3.32) | 2.08 (-2.77 to 6.94) | 70.42 |
| Myeloma | 2 (1.04) | 1.16 | 1.73 (0.21 to 6.25) | 0.86 (-1.95 to 3.66) | .. |
| Acute Lymphocytic Leukemia | 0 (0) | 0.12 | 0 (0 to 31.81) | -0.12 (-0.12 to -0.12) | 45.84 |
| Chronic Myeloid Leukemia | 2 (1.04) | 0.28 | 7.1 (0.86 to 25.64) | 1.74 (-1.07 to 4.54) | 33.29 |
| Acute Non-Lymphocytic Leukemia | 2 (1.04) | 0.68 | 2.93 (0.35 to 10.57) | 1.33 (-1.47 to 4.13) | 67.23 |
| Others | 10 (5.18) | 3.16 | 3.17 (1.52 to 5.82) | 6.92 (0.65 to 13.19) | 60.72 |

Abbreviations: SIR, standardized incidence ratio; SPC, subsequent primary cancer

**Supplementary table38-43 were shown in excel.**

**Supplementary table44. The risk of developing SPC among survivors diagnosed with primary malignant peripheral nerve sheath tumor with single outcome during 2000-2019 in 17 SEER registers.**

| SPC type | Observed SPCs,  No. (%) | Expected SPCs,  No. | SIR (95% CI) | Absolute excess incidence per 10,000 person-year | Mean age at SPC diagnosis, yr |
| --- | --- | --- | --- | --- | --- |
| Total | 160 (100) | 66.61 | 2.4 (2.04 to 2.8) | 101.53 (74.58 to 128.48) | 58.28 |
| Total (excluded MPNST) | 129 (80.63) | 66.61 | 1.94 (1.62 to 2.30) | 67.83 (43.63 to 92.03) | .. |
| Head and Neck | 5 (3.13) | 2.34 | 2.14 (0.7 to 4.99) | 2.9 (-1.87 to 7.66) | 60.72 |
| Esophagus | 0 (0) | 0.68 | 0 (0 to 5.4) | -0.74 (-0.74 to -0.74) | .. |
| Stomach | 3 (1.88) | 1.1 | 2.72 (0.56 to 7.96) | 2.06 (-1.63 to 5.75) | 77.44 |
| Small Intestine | 4 (2.5) | 0.34 | 11.95 (3.26 to 30.59) | 3.99 (-0.28 to 8.25) | 46.65 |
| Colon and Rectum | 6 (3.75) | 6.09 | 0.99 (0.36 to 2.14) | -0.1 (-5.32 to 5.12) | 78.97 |
| Anus, Anal Canal and Anorectum | 0 (0) | 0.27 | 0 (0 to 13.77) | -0.29 (-0.29 to -0.29) | .. |
| Liver and intrahepatic bile duct | 1 (0.63) | 1.37 | 0.73 (0.02 to 4.06) | -0.4 (-2.53 to 1.73) | 56.34 |
| Gallbladder and other biliary | 1 (0.63) | 0.44 | 2.26 (0.06 to 12.59) | 0.61 (-1.52 to 2.74) | 45.42 |
| Pancreas | 2 (1.25) | 1.88 | 1.06 (0.13 to 3.84) | 0.13 (-2.88 to 3.14) | 77.22 |
| Retroperitoneum, peritoneum and other digestive system | 1 (0.63) | 0.23 | 4.45 (0.11 to 24.77) | 0.84 (-1.29 to 2.97) | 72.76 |
| Lung and Bronchus | 12 (7.5) | 8.25 | 1.45 (0.75 to 2.54) | 4.07 (-3.31 to 11.45) | 57.4 |
| Pleura, trachea and other respiratory | 3 (1.88) | 0.02 | 123.91 (25.55 to 362.13) | 3.24 (-0.46 to 6.93) | 41.36 |
| Bones and Joints | 2 (1.25) | 0.1 | 20.41 (2.47 to 73.71) | 2.07 (-0.95 to 5.08) | 67.72 |
| Soft Tissue including Heart | 41 (25.63) | 0.44 | 92.46 (66.35 to 125.43) | 44.09 (30.45 to 57.73) | 49.31 |
| Soft Tissue including Heart (excluded MPNST) | 12 | 0.44 | 27.27 (14.09 to 47.64) | 12.56 (5.18 to 19.95) | 69.35 |
| Melanoma of the Skin | 15 (9.38) | 3.32 | 4.52 (2.53 to 7.46) | 12.7 (4.45 to 20.96) | 63.09 |
| Breast | 10 (6.25) | 8.99 | 1.11 (0.53 to 2.05) | 1.1 (-5.64 to 7.84) | .. |
| Cervix Uteri | 0 (0) | 0.47 | 0 (0 to 7.81) | -0.51 (-0.51 to -0.51) | 47.13 |
| Corpus and Uterus, NOS | 2 (1.25) | 1.9 | 1.05 (0.13 to 3.8) | 0.11 (-2.9 to 3.12) | 54.58 |
| Ovary | 1 (0.63) | 0.8 | 1.25 (0.03 to 6.97) | 0.22 (-1.91 to 2.35) | .. |
| Vagina, vulva and other female genital organs | 0 (0) | 0.32 | 0 (0 to 11.4) | -0.35 (-0.35 to -0.35) | 72.32 |
| Prostate | 8 (5) | 10.16 | 0.79 (0.34 to 1.55) | -2.35 (-8.37 to 3.68) | .. |
| Testis | 0 (0) | 0.29 | 0 (0 to 12.55) | -0.32 (-0.32 to -0.32) | .. |
| Penis and other male genital organs | 0 (0) | 0.08 | 0 (0 to 44.31) | -0.09 (-0.09 to -0.09) | 67.09 |
| Urinary Bladder | 4 (2.5) | 3.08 | 1.3 (0.35 to 3.33) | 1 (-3.26 to 5.26) | 64.69 |
| Kidney and Renal Pelvis | 6 (3.75) | 2.36 | 2.54 (0.93 to 5.53) | 3.95 (-1.27 to 9.17) | .. |
| Ureter and other urinary organs | 0 (0) | 0.14 | 0 (0 to 27.24) | -0.15 (-0.15 to -0.15) | .. |
| Eye and Orbit | 0 (0) | 0.11 | 0 (0 to 33.88) | -0.12 (-0.12 to -0.12) | 51.18 |
| Brain and Other Nervous System | 7 (4.38) | 0.79 | 8.86 (3.56 to 18.26) | 6.75 (1.11 to 12.39) | 41.2 |
| Thyroid | 8 (5) | 1.62 | 4.95 (2.14 to 9.76) | 6.94 (0.91 to 12.97) | .. |
| Other endocrine system | 0 (0) | 0.09 | 0 (0 to 42.47) | -0.09 (-0.09 to -0.09) | .. |
| Hodgkin Lymphoma | 0 (0) | 0.28 | 0 (0 to 13.06) | -0.31 (-0.31 to -0.31) | 61 |
| Non-Hodgkin Lymphoma | 4 (2.5) | 3.5 | 1.14 (0.31 to 2.92) | 0.54 (-3.72 to 4.8) | 70.42 |
| Myeloma | 2 (1.25) | 1.01 | 1.98 (0.24 to 7.17) | 1.08 (-1.94 to 4.09) | .. |
| Acute Lymphocytic Leukemia | 0 (0) | 0.11 | 0 (0 to 34.3) | -0.12 (-0.12 to -0.12) | 42.34 |
| Chronic Myeloid Leukemia | 1 (0.63) | 0.25 | 3.99 (0.1 to 22.22) | 0.81 (-1.32 to 2.94) | 33.29 |
| Acute Non-Lymphocytic Leukemia | 2 (1.25) | 0.6 | 3.31 (0.4 to 11.95) | 1.52 (-1.5 to 4.53) | 65.84 |
| Others | 9 (5.63) | 2.78 | 3.23 (1.48 to 6.14) | 6.76 (0.36 to 13.15) | 58.28 |

Abbreviations: SIR, standardized incidence ratio; SPC, subsequent primary cancer;

**Supplementary table 45. The risk of developing SPC among 5-year survivors diagnosed with primary malignant peripheral nerve sheath tumor during 2000-2019 in 17 SEER registers.**

| SPC type | Observed SPCs,  No. (%) | Expected SPCs,  No. | SIR (95% CI) | Absolute excess incidence per 10,000 person-year | Mean age at SPC diagnosis, yr |
| --- | --- | --- | --- | --- | --- |
| Total | 88 (100) | 39.65 | 2.22 (1.78 to 2.73) | 100.99 (62.59 to 139.4) | 62.09 |
| Total (excluded MPNST) | 70 (79.55) | 39.65 | 1.77 (1.38 to 2,23) | 63.39 (29.14, 97.65) | .. |
| Head and Neck | 2 (2.27) | 1.41 | 1.42 (0.17 to 5.14) | 1.24 (-4.55 to 7.03) | 70.88 |
| Esophagus | 0 (0) | 0.42 | 0 (0 to 8.9) | -0.87 (-0.87 to -0.87) | .. |
| Stomach | 1 (1.14) | 0.66 | 1.52 (0.04 to 8.49) | 0.72 (-3.38 to 4.81) | 73.92 |
| Small Intestine | 1 (1.14) | 0.21 | 4.78 (0.12 to 26.6) | 1.65 (-2.44 to 5.75) | 79.92 |
| Colon and Rectum | 5 (5.68) | 3.5 | 1.43 (0.46 to 3.33) | 3.13 (-6.03 to 12.28) | 72.77 |
| Anus, Anal Canal and Anorectum | 0 (0) | 0.16 | 0 (0 to 22.53) | -0.34 (-0.34 to -0.34) | .. |
| Liver and intrahepatic bile duct | 1 (1.14) | 0.86 | 1.16 (0.03 to 6.45) | 0.29 (-3.81 to 4.38) | 56.34 |
| Gallbladder and other biliary | 1 (1.14) | 0.27 | 3.67 (0.09 to 20.45) | 1.52 (-2.57 to 5.61) | 45.42 |
| Pancreas | 2 (2.27) | 1.18 | 1.7 (0.21 to 6.14) | 1.72 (-4.07 to 7.51) | 77.22 |
| Retroperitoneum, peritoneum and other digestive system | 1 (1.14) | 0.14 | 7.21 (0.18 to 40.16) | 1.8 (-2.3 to 5.89) | 72.76 |
| Lung and Bronchus | 4 (4.55) | 4.92 | 0.81 (0.22 to 2.08) | -1.92 (-10.1 to 6.27) | 62.52 |
| Pleura, trachea and other respiratory | 1 (1.14) | 0.01 | 73.62 (1.86 to 410.16) | 2.06 (-2.03 to 6.15) | 44.17 |
| Bones and Joints | 2 (2.27) | 0.05 | 37.17 (4.5 to 134.26) | 4.07 (-1.72 to 9.85) | 67.72 |
| Soft Tissue including Heart | 25 (28.41) | 0.26 | 95.42 (61.75 to 140.86) | 51.67 (31.2 to 72.14) | 45.99 |
| Soft Tissue including Heart (excluded MPNST) | 8 (9.09) | 0.26 | 30.53 (13.18 to 60.16) | 16.16 (4.58 to 27.74) | 67.6 |
| Melanoma of the Skin | 9 (10.23) | 2.05 | 4.4 (2.01 to 8.35) | 14.52 (2.24 to 26.81) | 60.97 |
| Breast | 9 (10.23) | 5.26 | 1.71 (0.78 to 3.25) | 7.81 (-4.48 to 20.09) | .. |
| Cervix Uteri | 0 (0) | 0.25 | 0 (0 to 14.56) | -1.1 (-1.1 to -1.1) | 37.33 |
| Corpus and Uterus, NOS | 1 (1.14) | 1.15 | 0.87 (0.02 to 4.85) | -0.65 (-9.2 to 7.89) | 54.58 |
| Ovary | 1 (1.14) | 0.45 | 2.22 (0.06 to 12.36) | 2.4 (-6.15 to 10.94) | .. |
| Vagina, vulva and other female genital organs | 0 (0) | 0.2 | 0 (0 to 18.28) | -0.88 (-0.88 to -0.88) | 70.34 |
| Prostate | 5 (5.68) | 5.96 | 0.84 (0.27 to 1.96) | -3.86 (-21.44 to 13.72) | .. |
| Testis | 0 (0) | 0.15 | 0 (0 to 25.21) | -0.59 (-0.59 to -0.59) | .. |
| Penis and other male genital organs | 0 (0) | 0.05 | 0 (0 to 70.85) | -0.21 (-0.21 to -0.21) | 75.06 |
| Urinary Bladder | 3 (3.41) | 1.88 | 1.6 (0.33 to 4.66) | 2.34 (-4.75 to 9.43) | 70.75 |
| Kidney and Renal Pelvis | 4 (4.55) | 1.45 | 2.76 (0.75 to 7.05) | 5.32 (-2.87 to 13.51) | .. |
| Ureter and other urinary organs | 0 (0) | 0.09 | 0 (0 to 42.63) | -0.18 (-0.18 to -0.18) | .. |
| Eye and Orbit | 0 (0) | 0.07 | 0 (0 to 56.88) | -0.14 (-0.14 to -0.14) | .. |
| Brain and Other Nervous System | 0 (0) | 0.45 | 0 (0 to 8.13) | -0.95 (-0.95 to -0.95) | 78.17 |
| Thyroid | 1 (1.14) | 0.94 | 1.07 (0.03 to 5.94) | 0.13 (-3.97 to 4.22) | .. |
| Other endocrine system | 0 (0) | 0.05 | 0 (0 to 73.46) | -0.11 (-0.11 to -0.11) | .. |
| Hodgkin Lymphoma | 0 (0) | 0.15 | 0 (0 to 25.26) | -0.31 (-0.31 to -0.31) | 77.64 |
| Non-Hodgkin Lymphoma | 3 (3.41) | 2.11 | 1.42 (0.29 to 4.15) | 1.86 (-5.23 to 8.95) | 84.34 |
| Myeloma | 1 (1.14) | 0.64 | 1.57 (0.04 to 8.75) | 0.76 (-3.34 to 4.85) | .. |
| Acute Lymphocytic Leukemia | 0 (0) | 0.06 | 0 (0 to 65.66) | -0.12 (-0.12 to -0.12) | .. |
| Chronic Myeloid Leukemia | 0 (0) | 0.15 | 0 (0 to 24.33) | -0.32 (-0.32 to -0.32) | .. |
| Acute Non-Lymphocytic Leukemia | 0 (0) | 0.37 | 0 (0 to 9.95) | -0.77 (-0.77 to -0.77) | 78.43 |
| Others | 5 (5.68) | 1.68 | 2.99 (0.97 to 6.97) | 6.95 (-2.21 to 16.1) | 62.09 |

Abbreviations: SIR, standardized incidence ratio; SPC, subsequent primary cancer;

**Supplementary table 46. The risk of developing SPC among survivors diagnosed with primary malignant peripheral nerve sheath tumor (including distant stage at diagnosis) during 2000-2019 in 17 SEER registers.**

| SPC type | Observed SPCs,  No. (%) | Expected SPCs,  No. | SIR (95% CI) | Absolute excess incidence per 10,000 person-year | Mean age at SPC diagnosis, yr |
| --- | --- | --- | --- | --- | --- |
| Total | 222 (100) | 91.66 | 2.42 (2.11 to 2.76) | 110.79 (85.96 to 135.61) | 59.09 |
| Total (excluded MPNST) | 178 (80.18) | 91.66 | 1.94 (1.67 to 2.25) | 73.39 (51.16 to 95.61) | .. |
| Head and Neck | 5 (2.25) | 3.18 | 1.57 (0.51 to 3.67) | 1.54 (-2.18 to 5.27) | 60.72 |
| Esophagus | 0 (0) | 0.95 | 0 (0 to 3.89) | -0.81 (-0.81 to -0.81) | .. |
| Stomach | 4 (1.8) | 1.51 | 2.66 (0.72 to 6.8) | 2.12 (-1.21 to 5.45) | 76.56 |
| Small Intestine | 5 (2.25) | 0.46 | 10.83 (3.52 to 25.28) | 3.86 (0.13 to 7.58) | 53.3 |
| Colon and Rectum | 9 (4.05) | 8.41 | 1.07 (0.49 to 2.03) | 0.5 (-4.49 to 5.5) | 76.21 |
| Anus, Anal Canal and Anorectum | 0 (0) | 0.36 | 0 (0 to 10.19) | -0.31 (-0.31 to -0.31) | .. |
| Liver and intrahepatic bile duct | 1 (0.45) | 1.86 | 0.54 (0.01 to 3) | -0.73 (-2.39 to 0.94) | 56.34 |
| Gallbladder and other biliary | 1 (0.45) | 0.62 | 1.62 (0.04 to 9.02) | 0.33 (-1.34 to 1.99) | 45.42 |
| Pancreas | 4 (1.8) | 2.64 | 1.52 (0.41 to 3.88) | 1.16 (-2.17 to 4.49) | 66.11 |
| Retroperitoneum, peritoneum and other digestive system | 1 (0.45) | 0.31 | 3.19 (0.08 to 17.78) | 0.58 (-1.08 to 2.25) | 72.76 |
| Lung and Bronchus | 16 (7.21) | 11.62 | 1.38 (0.79 to 2.24) | 3.73 (-2.94 to 10.39) | 57.75 |
| Pleura, trachea and other respiratory | 3 (1.35) | 0.03 | 92.16 (19.01 to 269.32) | 2.52 (-0.36 to 5.41) | 41.36 |
| Bones and Joints | 2 (0.9) | 0.13 | 15.59 (1.89 to 56.32) | 1.59 (-0.76 to 3.95) | 67.72 |
| Soft Tissue including Heart | 60 (27.03) | 0.6 | 100.13 (76.41 to 128.88) | 50.49 (37.58 to 63.39) | 48.35 |
| Soft Tissue including Heart (excluded MPNST) | 18 (8.11) | 0.6 | 30.49 (17.81 to 47.05) | 14.79 (7.72 to 21.86) | 69.3 |
| Melanoma of the Skin | 22 (9.91) | 4.55 | 4.84 (3.03 to 7.33) | 14.83 (7.02 to 22.65) | 64.16 |
| Breast | 16 (7.21) | 12.22 | 1.31 (0.75 to 2.13) | 3.21 (-3.45 to 9.87) | .. |
| Cervix Uteri | 0 (0) | 0.61 | 0 (0 to 6.01) | -1.09 (-1.09 to -1.09) | 47.13 |
| Corpus and Uterus, NOS | 2 (0.9) | 2.58 | 0.78 (0.09 to 2.81) | -1.02 (-5.92 to 3.89) | 67.24 |
| Ovary | 2 (0.9) | 1.1 | 1.82 (0.22 to 6.57) | 1.59 (-3.31 to 6.5) | .. |
| Vagina, vulva and other female genital organs | 0 (0) | 0.45 | 0 (0 to 8.19) | -0.8 (-0.8 to -0.8) | 69.34 |
| Prostate | 12 (5.41) | 13.96 | 0.86 (0.44 to 1.5) | -3.2 (-14.3 to 7.91) | .. |
| Testis | 0 (0) | 0.36 | 0 (0 to 10.12) | -0.6 (-0.6 to -0.6) | .. |
| Penis and other male genital organs | 0 (0) | 0.11 | 0 (0 to 32.3) | -0.19 (-0.19 to -0.19) | 67.09 |
| Urinary Bladder | 4 (1.8) | 4.33 | 0.93 (0.25 to 2.37) | -0.28 (-3.61 to 3.05) | 68.24 |
| Kidney and Renal Pelvis | 7 (3.15) | 3.23 | 2.17 (0.87 to 4.46) | 3.2 (-1.21 to 7.61) | .. |
| Ureter and other urinary organs | 0 (0) | 0.19 | 0 (0 to 19.2) | -0.16 (-0.16 to -0.16) | .. |
| Eye and Orbit | 0 (0) | 0.15 | 0 (0 to 24.71) | -0.13 (-0.13 to -0.13) | 51.64 |
| Brain and Other Nervous System | 8 (3.6) | 1.07 | 7.5 (3.24 to 14.78) | 5.89 (1.18 to 10.6) | 45.31 |
| Thyroid | 9 (4.05) | 2.09 | 4.3 (1.97 to 8.16) | 5.87 (0.87 to 10.87) | .. |
| Other endocrine system | 0 (0) | 0.12 | 0 (0 to 31.77) | -0.1 (-0.1 to -0.1) | .. |
| Hodgkin Lymphoma | 0 (0) | 0.36 | 0 (0 to 10.16) | -0.31 (-0.31 to -0.31) | 69.16 |
| Non-Hodgkin Lymphoma | 10 (4.5) | 4.86 | 2.06 (0.99 to 3.78) | 4.37 (-0.9 to 9.64) | 70.42 |
| Myeloma | 2 (0.9) | 1.41 | 1.42 (0.17 to 5.12) | 0.5 (-1.85 to 2.86) | 17.42 |
| Acute Lymphocytic Leukemia | 1 (0.45) | 0.14 | 7.16 (0.18 to 39.91) | 0.73 (-0.93 to 2.4) | 45.84 |
| Chronic Myeloid Leukemia | 2 (0.9) | 0.34 | 5.82 (0.71 to 21.02) | 1.41 (-0.95 to 3.76) | 33.29 |
| Acute Non-Lymphocytic Leukemia | 2 (0.9) | 0.84 | 2.39 (0.29 to 8.62) | 0.99 (-1.37 to 3.34) | 70.38 |
| Others | 12 (5.41) | 3.91 | 3.07 (1.59 to 5.37) | 6.88 (1.11 to 12.65) | 60.72 |

Abbreviations: SIR, standardized incidence ratio; SPC, subsequent primary cancer;

**Supplementary table 47. The risk of developing SPC among survivors diagnosed with primary malignant peripheral nerve sheath tumor (aged less than 89 years) during 2000-2019 in 17 SEER registers.**

| SPC type | Observed SPCs,  No. (%) | Expected SPCs,  No. | SIR (95% CI) | Absolute excess incidence per 10,000 person-year | Mean age at SPC diagnosis, yr |
| --- | --- | --- | --- | --- | --- |
| Total | 187 (100) | 71.34 | 2.62 (2.26 to 3.03) | 118.82 (91.28 to 146.35) | 57.42 |
| Total (excluded MPNST) | 148 (79.14) | 71.34 | 2.07 (1.75 to 2.44) | 78.75 (54.26 to103.25) |  |
| Head and Neck | 5 (2.67) | 2.52 | 1.98 (0.64 to 4.63) | 2.55 (-1.96 to 7.05) | 60.72 |
| Esophagus | 0 (0) | 0.74 | 0 (0 to 5) | -0.76 (-0.76 to -0.76) | .. |
| Stomach | 4 (2.14) | 1.16 | 3.44 (0.94 to 8.81) | 2.92 (-1.11 to 6.94) | 76.56 |
| Small Intestine | 5 (2.67) | 0.36 | 13.79 (4.48 to 32.17) | 4.76 (0.26 to 9.27) | 53.3 |
| Colon and Rectum | 6 (3.21) | 6.4 | 0.94 (0.34 to 2.04) | -0.41 (-5.34 to 4.52) | 73.83 |
| Anus, Anal Canal and Anorectum | 0 (0) | 0.29 | 0 (0 to 12.86) | -0.3 (-0.3 to -0.3) | .. |
| Liver and intrahepatic bile duct | 1 (0.53) | 1.49 | 0.67 (0.02 to 3.75) | -0.5 (-2.51 to 1.52) | 56.34 |
| Gallbladder and other biliary | 1 (0.53) | 0.46 | 2.16 (0.06 to 12.02) | 0.55 (-1.46 to 2.56) | 45.42 |
| Pancreas | 2 (1.07) | 1.98 | 1.01 (0.12 to 3.64) | 0.02 (-2.83 to 2.87) | 77.22 |
| Retroperitoneum, peritoneum and other digestive system | 1 (0.53) | 0.24 | 4.2 (0.11 to 23.39) | 0.78 (-1.23 to 2.8) | 72.76 |
| Lung and Bronchus | 13 (6.95) | 8.88 | 1.46 (0.78 to 2.5) | 4.23 (-3.03 to 11.49) | 57.58 |
| Pleura, trachea and other respiratory | 3 (1.6) | 0.03 | 117.27 (24.18 to 342.7) | 3.06 (-0.43 to 6.54) | 41.36 |
| Bones and Joints | 1 (0.53) | 0.1 | 9.71 (0.25 to 54.09) | 0.92 (-1.09 to 2.93) | 41.02 |
| Soft Tissue including Heart | 52 (27.81) | 0.47 | 110.66 (82.65 to 145.11) | 52.93 (38.42 to 67.45) | 47.88 |
| Soft Tissue including Heart (excluded MPNST) | 15 (8.02) | 0.47 | 31.91 (17.86 to 52.64) | 14.93 (7.13 to 22.72) | 66.54 |
| Melanoma of the Skin | 18 (9.63) | 3.54 | 5.09 (3.01 to 8.04) | 14.85 (6.31 to 23.4) | 62.9 |
| Breast | 14 (7.49) | 9.61 | 1.46 (0.8 to 2.44) | 4.51 (-3.03 to 12.04) | .. |
| Cervix Uteri | 0 (0) | 0.5 | 0 (0 to 7.32) | -1.08 (-1.08 to -1.08) | 47.13 |
| Corpus and Uterus, NOS | 2 (1.07) | 2.06 | 0.97 (0.12 to 3.52) | -0.12 (-6.07 to 5.84) | 54.58 |
| Ovary | 1 (0.53) | 0.84 | 1.19 (0.03 to 6.64) | 0.35 (-3.87 to 4.56) | .. |
| Vagina, vulva and other female genital organs | 0 (0) | 0.34 | 0 (0 to 10.99) | -0.72 (-0.72 to -0.72) | 68.17 |
| Prostate | 9 (4.81) | 11.21 | 0.8 (0.37 to 1.52) | -4.35 (-15.92 to 7.22) | .. |
| Testis | 0 (0) | 0.31 | 0 (0 to 11.96) | -0.61 (-0.61 to -0.61) | .. |
| Penis and other male genital organs | 0 (0) | 0.09 | 0 (0 to 41.4) | -0.18 (-0.18 to -0.18) | 67.09 |
| Urinary Bladder | 4 (2.14) | 3.25 | 1.23 (0.34 to 3.16) | 0.77 (-3.25 to 4.8) | 64.69 |
| Kidney and Renal Pelvis | 6 (3.21) | 2.56 | 2.34 (0.86 to 5.09) | 3.53 (-1.4 to 8.46) | .. |
| Ureter and other urinary organs | 0 (0) | 0.14 | 0 (0 to 25.9) | -0.15 (-0.15 to -0.15) | .. |
| Eye and Orbit | 0 (0) | 0.12 | 0 (0 to 31.66) | -0.12 (-0.12 to -0.12) | 51.64 |
| Brain and Other Nervous System | 8 (4.28) | 0.84 | 9.47 (4.09 to 18.67) | 7.35 (1.66 to 13.05) | 45.31 |
| Thyroid | 9 (4.81) | 1.74 | 5.17 (2.36 to 9.81) | 7.46 (1.42 to 13.5) | .. |
| Other endocrine system | 0 (0) | 0.09 | 0 (0 to 39.16) | -0.1 (-0.1 to -0.1) | .. |
| Hodgkin Lymphoma | 0 (0) | 0.3 | 0 (0 to 12.35) | -0.31 (-0.31 to -0.31) | 66.07 |
| Non-Hodgkin Lymphoma | 6 (3.21) | 3.72 | 1.61 (0.59 to 3.51) | 2.35 (-2.59 to 7.28) | 70.42 |
| Myeloma | 2 (1.07) | 1.09 | 1.83 (0.22 to 6.61) | 0.93 (-1.92 to 3.78) | .. |
| Acute Lymphocytic Leukemia | 0 (0) | 0.11 | 0 (0 to 32.5) | -0.12 (-0.12 to -0.12) | 45.84 |
| Chronic Myeloid Leukemia | 2 (1.07) | 0.27 | 7.53 (0.91 to 27.19) | 1.78 (-1.07 to 4.63) | 33.29 |
| Acute Non-Lymphocytic Leukemia | 2 (1.07) | 0.64 | 3.13 (0.38 to 11.31) | 1.4 (-1.45 to 4.25) | 67.23 |
| Others | 10 (5.35) | 2.85 | 3.51 (1.68 to 6.45) | 7.34 (0.98 to 13.71) | 57.42 |

Abbreviations: SIR, standardized incidence ratio; SPC, subsequent primary cancer;

**Supplementary table 48. The risk of developing SPC among survivors diagnosed with primary malignant peripheral nerve sheath tumor during 1975-2019 in 8 SEER registers.**

| SPC type | Observed SPCs,  No. (%) | Expected SPCs,  No. | SIR (95% CI) | Absolute excess incidence per 10,000 person-year | Mean age at SPC diagnosis, yr |
| --- | --- | --- | --- | --- | --- |
| Total | 171 (100) | 91.65 | 1.87 (1.6 to 2.17) | 78.56 (53.18 to 103.93) | 59.34 |
| Total (excluded MPNST) | 152 (88.89) | 91.65 | 1.66 (1.41 to 1.94) | 59.75 (35.83 to 83.67) | .. |
| Head and Neck | 2 (1.17) | 3.2 | 0.63 (0.08 to 2.26) | -1.19 (-3.93 to 1.55) | 34.34 |
| Esophagus | 1 (0.58) | 0.99 | 1.02 (0.03 to 5.66) | 0.02 (-1.93 to 1.96) | 74.16 |
| Stomach | 2 (1.17) | 1.56 | 1.28 (0.16 to 4.63) | 0.44 (-2.31 to 3.18) | 62.38 |
| Small Intestine | 5 (2.92) | 0.4 | 12.39 (4.02 to 28.92) | 4.55 (0.21 to 8.89) | 61.15 |
| Colon and Rectum | 5 (2.92) | 9.41 | 0.53 (0.17 to 1.24) | -4.37 (-8.71 to -0.03) | 60.47 |
| Anus, Anal Canal and Anorectum | 0 (0) | 0.3 | 0 (0 to 12.39) | -0.3 (-0.3 to -0.3) | .. |
| Liver and intrahepatic bile duct | 0 (0) | 1.46 | 0 (0 to 2.52) | -1.45 (-1.45 to -1.45) | .. |
| Gallbladder and other biliary | 2 (1.17) | 0.59 | 3.4 (0.41 to 12.3) | 1.4 (-1.35 to 4.14) | 56.84 |
| Pancreas | 1 (0.58) | 2.5 | 0.4 (0.01 to 2.23) | -1.49 (-3.43 to 0.45) | 86.25 |
| Retroperitoneum, peritoneum and other digestive system | 1 (0.58) | 0.29 | 3.48 (0.09 to 19.37) | 0.71 (-1.24 to 2.65) | 34.56 |
| Lung and Bronchus | 11 (6.43) | 12.12 | 0.91 (0.45 to 1.62) | -1.11 (-7.54 to 5.33) | 64.1 |
| Pleura, trachea and other respiratory | 0 (0) | 0.03 | 0 (0 to 110.56) | -0.03 (-0.03 to -0.03) | .. |
| Bones and Joints | 0 (0) | 0.12 | 0 (0 to 32.16) | -0.11 (-0.11 to -0.11) | .. |
| Soft Tissue including Heart | 40 (23.39) | 0.51 | 77.76 (55.55 to 105.89) | 39.09 (26.82 to 51.36) | 50.55 |
| Soft Tissue including Heart (excluded MPNST) | 22 (12.87) | 0.51 | 43.14 (27.03 to 65.31) | 21.27 (12.17 to 30.37) | 63.23 |
| Melanoma of the Skin | 7 (4.09) | 4.23 | 1.66 (0.67 to 3.41) | 2.75 (-2.39 to 7.88) | 65.59 |
| Breast | 21 (12.28) | 11.81 | 1.78 (1.1 to 2.72) | 9.1 (0.21 to 17.99) | .. |
| Cervix Uteri | 0 (0) | 0.53 | 0 (0 to 7.01) | -0.52 (-0.52 to -0.52) | 42.29 |
| Corpus and Uterus, NOS | 2 (1.17) | 2.45 | 0.82 (0.1 to 2.95) | -0.45 (-3.19 to 2.3) | 71.67 |
| Ovary | 1 (0.58) | 1.18 | 0.85 (0.02 to 4.74) | -0.17 (-2.11 to 1.77) | .. |
| Vagina, vulva and other female genital organs | 0 (0) | 0.42 | 0 (0 to 8.89) | -0.41 (-0.41 to -0.41) | 68.8 |
| Prostate | 17 (9.94) | 15.31 | 1.11 (0.65 to 1.78) | 1.68 (-6.32 to 9.68) | .. |
| Testis | 0 (0) | 0.29 | 0 (0 to 12.96) | -0.28 (-0.28 to -0.28) | .. |
| Penis and other male genital organs | 0 (0) | 0.11 | 0 (0 to 32.85) | -0.11 (-0.11 to -0.11) | 69.84 |
| Urinary Bladder | 9 (5.26) | 4.67 | 1.93 (0.88 to 3.66) | 4.29 (-1.53 to 10.11) | 65.75 |
| Kidney and Renal Pelvis | 4 (2.34) | 2.71 | 1.48 (0.4 to 3.79) | 1.28 (-2.6 to 5.16) | .. |
| Ureter and other urinary organs | 0 (0) | 0.2 | 0 (0 to 18.14) | -0.2 (-0.2 to -0.2) | 63.92 |
| Eye and Orbit | 1 (0.58) | 0.16 | 6.46 (0.16 to 36) | 0.84 (-1.1 to 2.78) | 48.89 |
| Brain and Other Nervous System | 11 (6.43) | 1.07 | 10.33 (5.16 to 18.48) | 9.84 (3.4 to 16.27) | 53.1 |
| Thyroid | 6 (3.51) | 1.46 | 4.11 (1.51 to 8.95) | 4.5 (-0.26 to 9.25) | .. |
| Other endocrine system | 0 (0) | 0.1 | 0 (0 to 37.4) | -0.1 (-0.1 to -0.1) | .. |
| Hodgkin Lymphoma | 0 (0) | 0.34 | 0 (0 to 10.9) | -0.34 (-0.34 to -0.34) | 68.13 |
| Non-Hodgkin Lymphoma | 7 (4.09) | 4.94 | 1.42 (0.57 to 2.92) | 2.04 (-3.09 to 7.17) | 56.88 |
| Myeloma | 2 (1.17) | 1.28 | 1.56 (0.19 to 5.63) | 0.71 (-2.03 to 3.45) | 55.17 |
| Acute Lymphocytic Leukemia | 1 (0.58) | 0.1 | 9.76 (0.25 to 54.36) | 0.89 (-1.05 to 2.83) | 49.34 |
| Chronic Myeloid Leukemia | 1 (0.58) | 0.34 | 2.97 (0.08 to 16.57) | 0.66 (-1.28 to 2.6) | 44.51 |
| Acute Non-Lymphocytic Leukemia | 1 (0.58) | 0.82 | 1.22 (0.03 to 6.8) | 0.18 (-1.76 to 2.12) | 61.16 |
| Others | 10 (5.85) | 3.69 | 2.71 (1.3 to 4.99) | 6.25 (0.11 to 12.39) | 59.34 |

Abbreviations: SIR, standardized incidence ratio; SPC, subsequent primary cancer;

**Supplementary table 49. Causes of death among survivors diagnosed with primary malignant peripheral nerve sheath tumor during 2000-2019 in 17 SEER registers.**

| COD type | Observed,  No. (%) | Expected  No. | SMR (95% CI) | Absolute excess incidence per 10,000 person-year | Mean age at COD,  yr |
| --- | --- | --- | --- | --- | --- |
| All Cause of Death | 1063 (100) | 152.96 | 6.95 (6.54 to 7.38) | 758.15 (704.91 to 811.38) | 51.48 |
| All malignant cancers | 790 (74.32) | 34.31 | 23.02 (21.45 to 24.69) | 629.55 (583.66 to 675.45) | 48.21 |
| Head and Neck | 9 (0.85) | 0.81 | 11.11 (5.08 to 21.09) | 6.82 (1.92 to 11.72) | 55.09 |
| Esophagus | 0 (0) | 0.9 | 0 (0 to 4.1) | -0.75 (-0.75 to -0.75) | .. |
| Stomach | 5 (0.47) | 0.72 | 6.99 (2.27 to 16.31) | 3.57 (-0.08 to 7.22) | 68.18 |
| Small Intestine | 0 (0) | 0.08 | 0 (0 to 45.19) | -0.07 (-0.07 to -0.07) | .. |
| Colon and Rectum | 5 (0.47) | 3.15 | 1.59 (0.52 to 3.71) | 1.54 (-2.11 to 5.19) | 73.15 |
| Anus, Anal Canal and Anorectum | 0 (0) | 0.05 | 0 (0 to 72.77) | -0.04 (-0.04 to -0.04) | .. |
| Liver and intrahepatic bile duct | 5 (0.47) | 1.4 | 3.57 (1.16 to 8.34) | 3 (-0.65 to 6.65) | 74.84 |
| Gallbladder and other biliary | 0 (0) | 0.22 | 0 (0 to 16.56) | -0.19 (-0.19 to -0.19) | .. |
| Pancreas | 3 (0.28) | 2.32 | 1.29 (0.27 to 3.78) | 0.57 (-2.26 to 3.4) | 79.76 |
| Retroperitoneum, peritoneum and other digestive system | 5 (0.47) | 0.06 | 78.48 (25.48 to 183.15) | 4.11 (0.46 to 7.76) | 52.15 |
| Lung and Bronchus | 55 (5.17) | 9.13 | 6.03 (4.54 to 7.85) | 38.22 (26.11 to 50.33) | 56.71 |
| Pleura, trachea and other respiratory | 2 (0.19) | 0.03 | 65.22 (7.9 to 235.59) | 1.64 (-0.67 to 3.95) | 60.84 |
| Bones and Joints | 19 (1.79) | 0.08 | 251.44 (151.38 to 392.66) | 15.77 (8.65 to 22.88) | 48.3 |
| Soft Tissue including Heart | 512 (48.17) | 0.25 | 2070.31 (1894.85 to 2257.66) | 426.34 (389.39 to 463.28) | 44.61 |
| Melanoma of the Skin | 15 (1.41) | 0.5 | 29.96 (16.77 to 49.41) | 12.08 (5.75 to 18.4) | 61.41 |
| Breast | 0 (0) | 2.22 | 0 (0 to 1.66) | -1.85 (-1.85 to -1.85) | .. |
| Cervix Uteri | 1 (0.09) | 0.21 | 4.72 (0.12 to 26.32) | 4.72 (0.12 to 26.32) | 44 |
| Corpus and Uterus, NOS | 1 (0.09) | 0.5 | 2.01 (0.05 to 11.2) | 2.01 (0.05 to 11.2) | 74.42 |
| Ovary | 2 (0.19) | 0.75 | 2.66 (0.32 to 9.6) | 2.66 (0.32 to 9.6) | 72.53 |
| Vagina, vulva and other female genital organs | 0 (0) | 0.11 | 0 (0 to 33.46) | 0 (0 to 33.46) | .. |
| Prostate | 1 (0.09) | 1.98 | 0.51 (0.01 to 2.82) | -0.82 (-4.72 to 1.57) | 62.09 |
| Testis | 0 (0) | 0.02 | 0 (0 to 197.98) | -0.02 (-0.03 to -0.03) | .. |
| Penis and other male genital organs | 0 (0) | 0.02 | 0 (0 to 169.04) | -0.02 (-0.04 to -0.04) | .. |
| Urinary Bladder | 3 (0.28) | 0.96 | 3.14 (0.65 to 9.17) | 1.7 (-1.13 to 4.53) | 66.75 |
| Kidney and Renal Pelvis | 2 (0.19) | 0.81 | 2.46 (0.3 to 8.9) | 0.99 (-1.32 to 3.3) | 47.96 |
| Ureter and other urinary organs | 0 (0) | 0.05 | 0 (0 to 74.27) | -0.04 (-0.04 to -0.04) | .. |
| Eye and Orbit | 1 (0.09) | 0.02 | 59.52 (1.51 to 331.64) | 0.82 (-0.81 to 2.45) | 53.92 |
| Brain and Other Nervous System | 29 (2.73) | 0.83 | 34.87 (23.35 to 50.08) | 23.47 (14.67 to 32.26) | 51.32 |
| Thyroid | 0 (0) | 0.11 | 0 (0 to 34.92) | -0.09 (-0.09 to -0.09) | .. |
| Other endocrine system | 4 (0.38) | 0.05 | 84.3 (22.97 to 215.83) | 3.29 (0.03 to 6.56) | 61.73 |
| Hodgkin Lymphoma | 0 (0) | 0.06 | 0 (0 to 59.08) | -0.05 (-0.05 to -0.05) | .. |
| Non-Hodgkin Lymphoma | 8 (0.75) | 1.49 | 5.37 (2.32 to 10.58) | 5.42 (0.8 to 10.04) | 55.28 |
| Myeloma | 1 (0.09) | 0.7 | 1.42 (0.04 to 7.92) | 0.25 (-1.39 to 1.88) | 56.59 |
| Acute Lymphocytic Leukemia | 0 (0) | 0.07 | 0 (0 to 54.1) | -0.06 (-0.06 to -0.06) | .. |
| Acute Non-Lymphocytic Leukemia | 0 (0) | 0.67 | 0 (0 to 5.53) | -0.56 (-0.56 to -0.56) | .. |
| Chronic Myeloid Leukemia | 0 (0) | 0.07 | 0 (0 to 56.99) | -0.05 (-0.05 to -0.05) | .. |
| Others | 102 (9.6) | 3.01 | 33.85 (27.6 to 41.09) | 82.46 (65.97 to 98.95) | 50.75 |
| In situ, benign or unknown behavior neoplasm | 60 (5.64) | 0.9 | 66.61 (50.83 to 85.74) | 49.24 (36.59 to 61.88) | 46.26 |
| Septicemia | 4 (0.38) | 2.23 | 1.8 (0.49 to 4.6) | 1.48 (-1.79 to 4.74) | 82.61 |
| Other Infectious and Parasitic Diseases including HIV | 4 (0.38) | 1.61 | 2.49 (0.68 to 6.38) | 2 (-1.27 to 5.26) | 44.61 |
| Diabetes Mellitus | 4 (0.38) | 4.68 | 0.86 (0.23 to 2.19) | -0.56 (-3.83 to 2.7) | 74.06 |
| Alzheimer’s (ICD-9 and 10 only) | 8 (0.75) | 5.65 | 1.42 (0.61 to 2.79) | 1.96 (-2.66 to 6.58) | 84.66 |
| CVD | 70 (6.59) | 50.68 | 1.38 (1.08 to 1.75) | 16.1 (2.44 to 29.76) | 73.91 |
| Diseases of Heart | 45 (4.23) | 38.63 | 1.17 (0.85 to 1.56) | 5.31 (-5.65 to 16.26) | 73.18 |
| Hypertension without Heart Disease | 4 (0.38) | 1.83 | 2.18 (0.59 to 5.59) | 1.81 (-1.46 to 5.07) | 80.73 |
| Cerebrovascular Diseases | 18 (1.69) | 8.54 | 2.11 (1.25 to 3.33) | 7.89 (0.96 to 14.81) | 72.71 |
| Atherosclerosis | 1 (0.09) | 0.45 | 2.24 (0.06 to 12.48) | 0.46 (-1.17 to 2.09) | 85.83 |
| Aortic Aneurysm and Dissection | 2 (0.19) | 0.66 | 3.03 (0.37 to 10.95) | 1.12 (-1.19 to 3.43) | 81.42 |
| Other Diseases of Arteries, Arterioles, Capillaries | 0 (0) | 0.57 | 0 (0 to 6.45) | -0.48 (-0.48 to -0.48) | .. |
| Pneumonia and Influenza | 11 (1.03) | 3.42 | 3.22 (1.61 to 5.76) | 6.32 (0.9 to 11.73) | 77.62 |
| Chronic Obstructive Pulmonary Disease and Allied Cond | 7 (0.66) | 8.56 | 0.82 (0.33 to 1.69) | -1.3 (-5.62 to 3.02) | 65.61 |
| Chronic Liver Disease and Cirrhosis | 2 (0.19) | 2.03 | 0.99 (0.12 to 3.57) | -0.02 (-2.33 to 2.29) | 72.58 |
| Nephritis, Nephrotic Syndrome and Nephrosis | 4 (0.38) | 2.96 | 1.35 (0.37 to 3.46) | 0.87 (-2.4 to 4.14) | 81.96 |
| Symptoms, Signs and Ill-Defined Conditions | 6 (0.56) | 1.84 | 3.26 (1.2 to 7.1) | 3.47 (-0.53 to 7.46) | 48.76 |
| Accidents and Adverse Effects | 6 (0.56) | 6.83 | 0.88 (0.32 to 1.91) | -0.69 (-4.69 to 3.31) | 55.92 |
| Suicide and Self-Inflicted Injury | 3 (0.28) | 1.96 | 1.53 (0.32 to 4.48) | 0.87 (-1.96 to 3.7) | 45.14 |
| Other COD | 83 (7.81) | 25.12 | 3.3 (2.63 to 4.1) | 48.22 (33.34 to 63.09) | 55.16 |
| Syphilis | 0 (0) | 0 | 0 (0 to 1435.84) | 0 (0 to 0) | .. |
| Tuberculosis | 0 (0) | 0.04 | 0 (0 to 96.02) | -0.03 (-0.03 to -0.03) | .. |
| Stomach and Duodenal Ulcers | 0 (0) | 0.2 | 0 (0 to 18.84) | -0.16 (-0.16 to -0.16) | .. |
| Puerperium | 0 (0) | 0.03 | 0 (0 to 145.93) | -0.02 (-0.02 to -0.02) | .. |
| Congenital Anomalies | 44 (4.14) | 0.22 | 197.21 (143.3 to 264.75) | 36.47 (25.64 to 47.3) | 44.56 |
| Certain Conditions Originating in. Perinatal Period | 0 (0) | 0.03 | 0 (0 to 134.37) | -0.02 (-0.02 to -0.02) | .. |
| Homicide and Legal Intervention | 0 (0) | 0.76 | 0 (0 to 4.89) | -0.63 (-0.63 to -0.63) | .. |
| Other Cause of Death | 39 (3.67) | 23.85 | 1.64 (1.16 to 2.24) | 12.62 (2.42 to 22.82) | 67.12 |

Abbreviations: SMR, standardized mortality ratio; COD, cause of death.

**Supplementary table50-S55 were shown in excel.**

**Supplementary table 56. Causes of death among survivors diagnosed with primary malignant peripheral nerve sheath tumor during 1975-2019 in 8 SEER registers.**

| COD type | Observed,  No. (%) | Expected  No.^c^ | SMR (95% CI)^d^ | Absolute excess incidence per 10,000 person-year^e^ | Mean age at COD,  yr |
| --- | --- | --- | --- | --- | --- |
| All Cause of Death | 950 (100) | 219.02 | 4.34 (4.07 to 4.62) | 570.22 (523.1 to 892116.17) | 55.71 |
| All malignant cancers | 674 (70.95) | 49.9 | 13.51 (12.51 to 14.57) | 486.85 (447.15 to 760893.43) | 50.35 |
| Head and Neck | 7 (0.74) | 1.18 | 5.95 (2.39 to 12.26) | 4.54 (0.5 to 12410.86) | 65.11 |
| Esophagus | 1 (0.11) | 1.2 | 0.84 (0.02 to 4.66) | -0.15 (-1.68 to 1988.5) | 74.92 |
| Stomach | 6 (0.63) | 1.17 | 5.12 (1.88 to 11.15) | 3.77 (0.02 to 10855.6) | 58.07 |
| Small Intestine | 2 (0.21) | 0.11 | 19.01 (2.3 to 68.66) | 1.48 (-0.68 to 5260.8) | 80.42 |
| Colon and Rectum | 5 (0.53) | 5.03 | 0.99 (0.32 to 2.32) | -0.02 (-3.44 to 4905.53) | 65.72 |
| Anus, Anal Canal and Anorectum | 0 (0) | 0.05 | 0 (0 to 69.33) | -0.04 (-0.04 to -59.75) | .. |
| Liver and intrahepatic bile duct | 2 (0.21) | 1.56 | 1.28 (0.16 to 4.63) | 0.34 (-1.82 to 3619.5) | 61.67 |
| Gallbladder and other biliary | 0 (0) | 0.36 | 0 (0 to 10.34) | -0.28 (-0.28 to -402.43) | .. |
| Pancreas | 5 (0.53) | 3.01 | 1.66 (0.54 to 3.87) | 1.55 (-1.87 to 7180.36) | 74.82 |
| Retroperitoneum, peritoneum and other digestive system | 12 (1.26) | 0.08 | 151.94 (78.51 to 265.41) | 9.3 (4 to 21091.92) | 52.23 |
| Lung and Bronchus | 34 (3.58) | 13.95 | 2.44 (1.69 to 3.41) | 15.64 (6.72 to 35480.4) | 58.14 |
| Pleura, trachea and other respiratory | 6 (0.63) | 0.06 | 104.92 (38.5 to 228.36) | 4.64 (0.89 to 12111.37) | 57.14 |
| Bones and Joints | 10 (1.05) | 0.09 | 106.01 (50.83 to 194.95) | 7.73 (2.89 to 18153.61) | 49.27 |
| Soft Tissue including Heart | 450 (47.37) | 0.32 | 1427 (1298.18 to 1565.14) | 350.79 (318.35 to 553785.23) | 47.12 |
| Melanoma of the Skin | 13 (1.37) | 0.7 | 18.69 (9.95 to 31.96) | 9.6 (4.09 to 21836.19) | 61.66 |
| Breast | 4 (0.42) | 3.17 | 1.26 (0.34 to 3.23) | 0.65 (-2.41 to 5352.27) | 59.94 |
| Cervix Uteri | 0 (0) | 0.28 | 0 (0 to 13.41) | -0.44 (-0.44 to -293.3) | .. |
| Corpus and Uterus, NOS | 1 (0.11) | 0.58 | 1.72 (0.04 to 9.56) | 0.67 (-2.47 to 2535.2) | 74.42 |
| Ovary | 2 (0.21) | 1.08 | 1.85 (0.22 to 6.67) | 1.47 (-2.97 to 3933.3) | 71.88 |
| Vagina, vulva and other female genital organs | 1 (0.11) | 0.14 | 7.18 (0.18 to 40.01) | 1.38 (-1.76 to 3008.75) | 67.67 |
| Prostate | 5 (0.53) | 3.12 | 1.6 (0.52 to 3.74) | 2.86 (-3.81, 7418.76) | 75.22 |
| Testis | 0 (0) | 0.02 | 0 (0 to 171.06) | -0.03 (-0.03 to -26.07) | .. |
| Penis and other male genital organs | 0 (0) | 0.03 | 0 (0 to 128.85) | -0.04 (-0.04 to -34.36) | .. |
| Urinary Bladder | 2 (0.21) | 1.35 | 1.48 (0.18 to 5.34) | 0.51 (-1.66 to 3855.1) | 74.59 |
| Kidney and Renal Pelvis | 1 (0.11) | 1.12 | 0.89 (0.02 to 4.96) | -0.1 (-1.63 to 2069.67) | 50.09 |
| Ureter and other urinary organs | 0 (0) | 0.07 | 0 (0 to 55.18) | -0.05 (-0.05 to -75.53) | .. |
| Eye and Orbit | 3 (0.32) | 0.02 | 123.19 (25.41 to 360.02) | 2.32 (-0.33 to 7181.63) | 66.03 |
| Brain and Other Nervous System | 21 (2.21) | 1.09 | 19.22 (11.9 to 29.38) | 15.53 (8.52 to 32565.49) | 48.47 |
| Thyroid | 1 (0.11) | 0.14 | 7.36 (0.19 to 41.01) | 0.67 (-0.85 to 3183.41) | 87.34 |
| Other endocrine system | 4 (0.42) | 0.06 | 67.15 (18.3 to 171.94) | 3.07 (0.02 to 8860.33) | 61.17 |
| Hodgkin Lymphoma | 1 (0.11) | 0.11 | 9.53 (0.24 to 53.12) | 0.7 (-0.83 to 3218.35) | 89.42 |
| Non-Hodgkin Lymphoma | 4 (0.42) | 2.32 | 1.72 (0.47 to 4.41) | 1.31 (-1.75 to 6309.32) | 62.09 |
| Myeloma | 3 (0.32) | 0.96 | 3.12 (0.64 to 9.1) | 1.59 (-1.06 to 6123.12) | 77.7 |
| Acute Lymphocytic Leukemia | 0 (0) | 0.09 | 0 (0 to 42.45) | -0.07 (-0.07 to -98.07) | .. |
| Acute Non-Lymphocytic Leukemia | 2 (0.21) | 0.93 | 2.14 (0.26 to 7.74) | 0.83 (-1.33 to 4326.3) | 83.46 |
| Chronic Myeloid Leukemia | 1 (0.11) | 0.14 | 7.28 (0.18 to 40.55) | 0.67 (-0.86 to 3182.28) | 51.23 |
| Others | 64 (6.74) | 4.39 | 14.57 (11.22 to 18.61) | 46.5 (34.27 to 84868.67) | 49.1 |
| In situ, benign or unknown behavior neoplasm | 48 (5.05) | 1.15 | 41.92 (30.91 to 55.58) | 36.55 (25.96 to 68125.67) | 51.42 |
| Septicemia | 5 (0.53) | 2.67 | 1.88 (0.61 to 4.38) | 1.82 (-1.6 to 7570.39) | 74.79 |
| Other Infectious and Parasitic Diseases including HIV | 2 (0.21) | 2.06 | 0.97 (0.12 to 3.51) | -0.05 (-2.21 to 3058.12) | 32.92 |
| Diabetes Mellitus | 2 (0.21) | 5.94 | 0.34 (0.04 to 1.22) | -3.07 (-5.23 to -1313.43) | 57.42 |
| Alzheimer’s (ICD-9 and 10 only) | 5 (0.53) | 6.23 | 0.8 (0.26 to 1.87) | -0.96 (-4.38 to 3551.68) | 88.9 |
| CVD | 107 (11.26) | 84.59 | 1.27 (1.04 to 1.53) | 17.49 (1.67 to 48121.29) | 78.38 |
| Diseases of Heart | 74 (7.79) | 65.04 | 1.14 (0.89 to 1.43) | 6.99 (-6.17 to 29102.2) | 78.45 |
| Hypertension without Heart Disease | 4 (0.42) | 1.98 | 2.02 (0.55 to 5.17) | 1.58 (-1.48 to 6695.98) | 90.11 |
| Cerebrovascular Diseases | 24 (2.53) | 14.12 | 1.7 (1.09 to 2.53) | 7.7 (0.21 to 21956.94) | 76.63 |
| Atherosclerosis | 3 (0.32) | 1.26 | 2.38 (0.49 to 6.96) | 1.36 (-1.29 to 5788.32) | 73.75 |
| Aortic Aneurysm and Dissection | 2 (0.21) | 1.31 | 1.52 (0.18 to 5.5) | 0.54 (-1.63 to 3899.06) | 80.5 |
| Other Diseases of Arteries, Arterioles, Capillaries | 0 (0) | 0.86 | 0 (0 to 4.27) | -0.67 (-0.67 to -973.96) | .. |
| Pneumonia and Influenza | 12 (1.26) | 6.06 | 1.98 (1.02 to 3.46) | 4.63 (-0.66 to 14347.47) | 73.37 |
| Chronic Obstructive Pulmonary Disease and Allied Cond | 12 (1.26) | 11.94 | 1.01 (0.52 to 1.76) | 0.05 (-5.25 to 7718) | 73.32 |
| Chronic Liver Disease and Cirrhosis | 2 (0.21) | 2.49 | 0.8 (0.1 to 2.9) | -0.39 (-2.55 to 2568.89) | 68.79 |
| Nephritis, Nephrotic Syndrome and Nephrosis | 3 (0.32) | 3.54 | 0.85 (0.18 to 2.48) | -0.42 (-3.07 to 3218.15) | 65.81 |
| Symptoms, Signs and Ill-Defined Conditions | 5 (0.53) | 2.42 | 2.07 (0.67 to 4.83) | 2.02 (-1.4 to 7852.21) | 74.5 |
| Accidents and Adverse Effects | 7 (0.74) | 7.42 | 0.94 (0.38 to 1.94) | -0.33 (-4.37 to 5372.19) | 56.24 |
| Suicide and Self-Inflicted Injury | 3 (0.32) | 2.13 | 1.41 (0.29 to 4.12) | 0.68 (-1.97 to 4810.98) | 53.33 |
| Other COD | 58 (6.11) | 30.22 | 1.92 (1.46 to 2.48) | 21.67 (10.03 to 48145.55) | 65.98 |
| Syphilis | 0 (0) | 0 | 0 (0 to 961.33) | 0 (0 to -4.51) | .. |
| Tuberculosis | 0 (0) | 0.09 | 0 (0 to 39.04) | -0.07 (-0.07 to -105.96) | .. |
| Stomach and Duodenal Ulcers | 1 (0.11) | 0.42 | 2.41 (0.06 to 13.4) | 0.46 (-1.07 to 2867.77) | 85.5 |
| Puerperium | 0 (0) | 0.02 | 0 (0 to 207.35) | -0.01 (-0.01 to -20.29) | .. |
| Congenital Anomalies | 16 (1.68) | 0.28 | 57.94 (33.12 to 94.09) | 12.27 (6.15 to 26562.96) | 51.14 |
| Certain Conditions Originating in. Perinatal Period | 0 (0) | 0.03 | 0 (0 to 145.73) | -0.02 (-0.02 to -28.18) | .. |
| Homicide and Legal Intervention | 0 (0) | 0.74 | 0 (0 to 4.96) | -0.58 (-0.58 to -837.56) | .. |
| Other Cause of Death | 41 (4.32) | 28.64 | 1.43 (1.03 to 1.94) | 9.64 (-0.15 to 28079.27) | 71.3 |

Abbreviations: SMR, standardized mortality ratio; COD, cause of death.

**Supplementary table 57. Comparison of patients with malignant peripheral nerve sheath tumor from the Chinese multicenter and the SEER database.**

|  | Multicenter | SEER | *P* value |
| --- | --- | --- | --- |
| Patients | 85 (100) | 157 (100) |  |
| Age |  |  | 0.002 |
| 00-19 years | 10 (11.8) | 175 (11.1) |  |
| 20-64 years | 72 (84.7) | 1118 (70.8) |  |
| ≥ 65 years | 3 (3.53) | 285 (18.1) |  |
| Sex |  |  | 0.418 |
| Male | 41 (48.2) | 842 (53.4) |  |
| Female | 44 (51.8) | 736 (46.6) |  |
| Married |  |  | 0.015 |
| Yes | 62 (72.9) | 931 (59.0) |  |
| No | 23 (27.1) | 647 (41.0) |  |
| Location |  |  | <0.001 |
| Urban | 56 (65.9) | 1406 (89.1) |  |
| Rural | 29 (34.1) | 172 (10.9) |  |
| Site |  |  | 0.001 |
| Head and neck | 22 (25.9) | 185 (11.7) |  |
| Intracranial | 6 (7.06) | 67 (4.25) |  |
| Limb | 29 (34.1) | 594 (37.6) |  |
| Others | 28 (32.9) | 732 (46.4) |  |
| Stage |  |  | 0.021 |
| I-III | 80 (94.1) | 1329 (84.2) |  |
| IV | 5 (5.88) | 249 (15.8) |  |
| Surgery |  |  | 0.082 |
| No | 5 (5.88) | 204 (12.9) |  |
| Yes | 80 (94.1) | 1374 (87.1) |  |
| Radiotherapy |  |  | <0.001 |
| No | 74 (87.1) | 841 (53.3) |  |
| Yes | 11 (12.9) | 737 (46.7) |  |
| Chemotherapy |  |  | 0.526 |
| No | 67 (78.8) | 1186 (75.2) |  |
| Yes | 18 (21.2) | 392 (24.8) |  |
| Other treatment |  |  |  |
| No | 72 (84.7) |  |  |
| Yes | 13 (15.3) |  |  |
| Recurrence |  |  |  |
| No | 51 (60.0) |  |  |
| Yes | 34 (40.0) |  |  |
| Hypertension |  |  |  |
| No | 75 (90.4) |  |  |
| Yes | 8 (9.64) |  |  |
| Diabetes |  |  |  |
| No | 80 (94.1) |  |  |
| Yes | 5 (5.9) |  |  |
| Cardiovascular disease |  |  |  |
| No | 82 (96.5) |  |  |
| Yes | 3 (3.53) |  |  |
| Asthma |  |  |  |
| No | 85 (100) |  |  |
| Yes | 0 (0) |  |  |
| COPD |  |  |  |
| No | 85 (100) |  |  |
| Yes | 0 (0) |  |  |
| Subsequence primary cancer |  |  |  |
| No | 79 (92.9) |  |  |
| Yes | 6 (7.1) |  |  |

**Supplementary figure 1.** Flowchart of study.


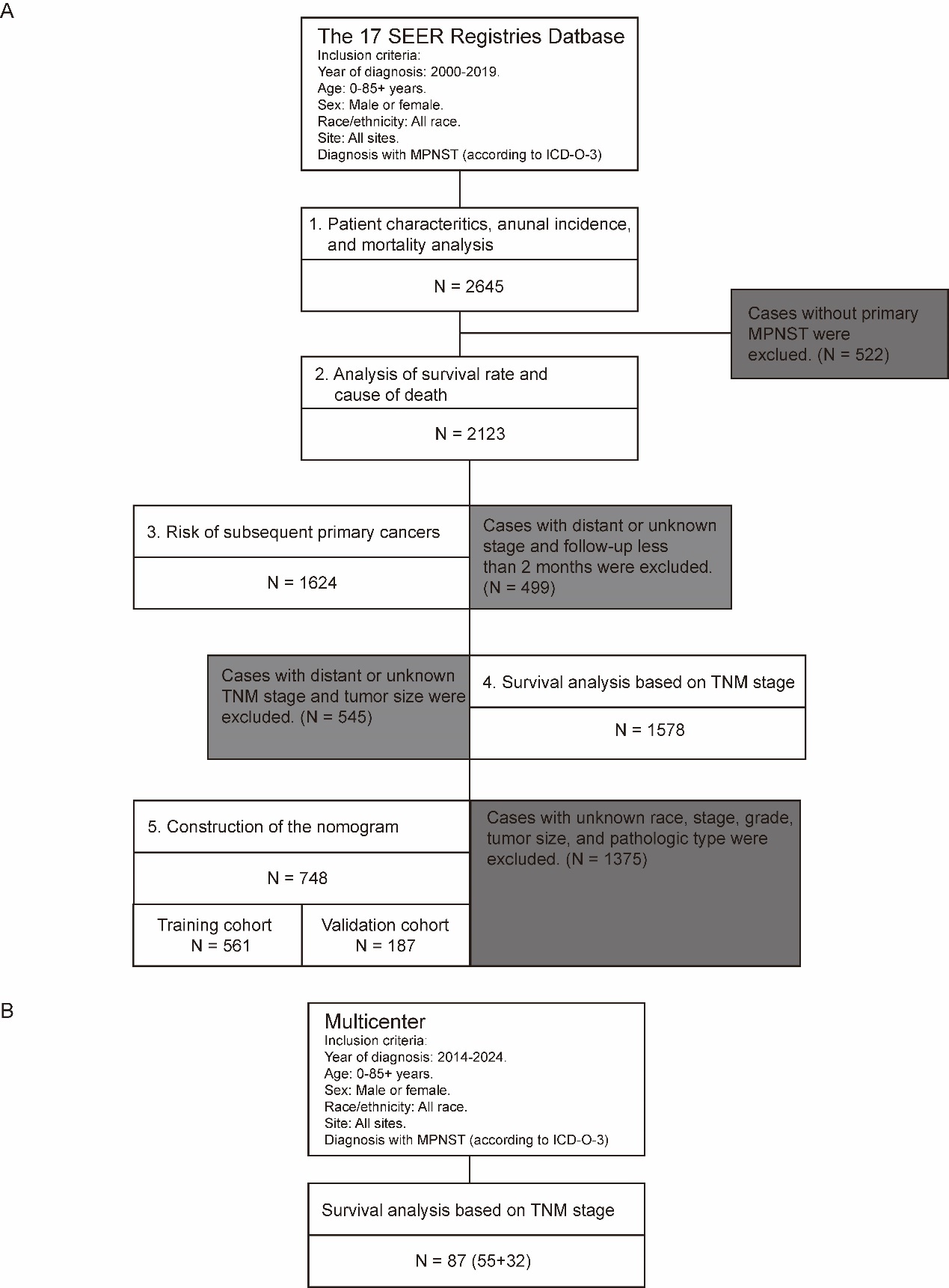


**Supplementary figure 2. Incidence and prevalence of malignant peripheral nerve sheath tumor.**


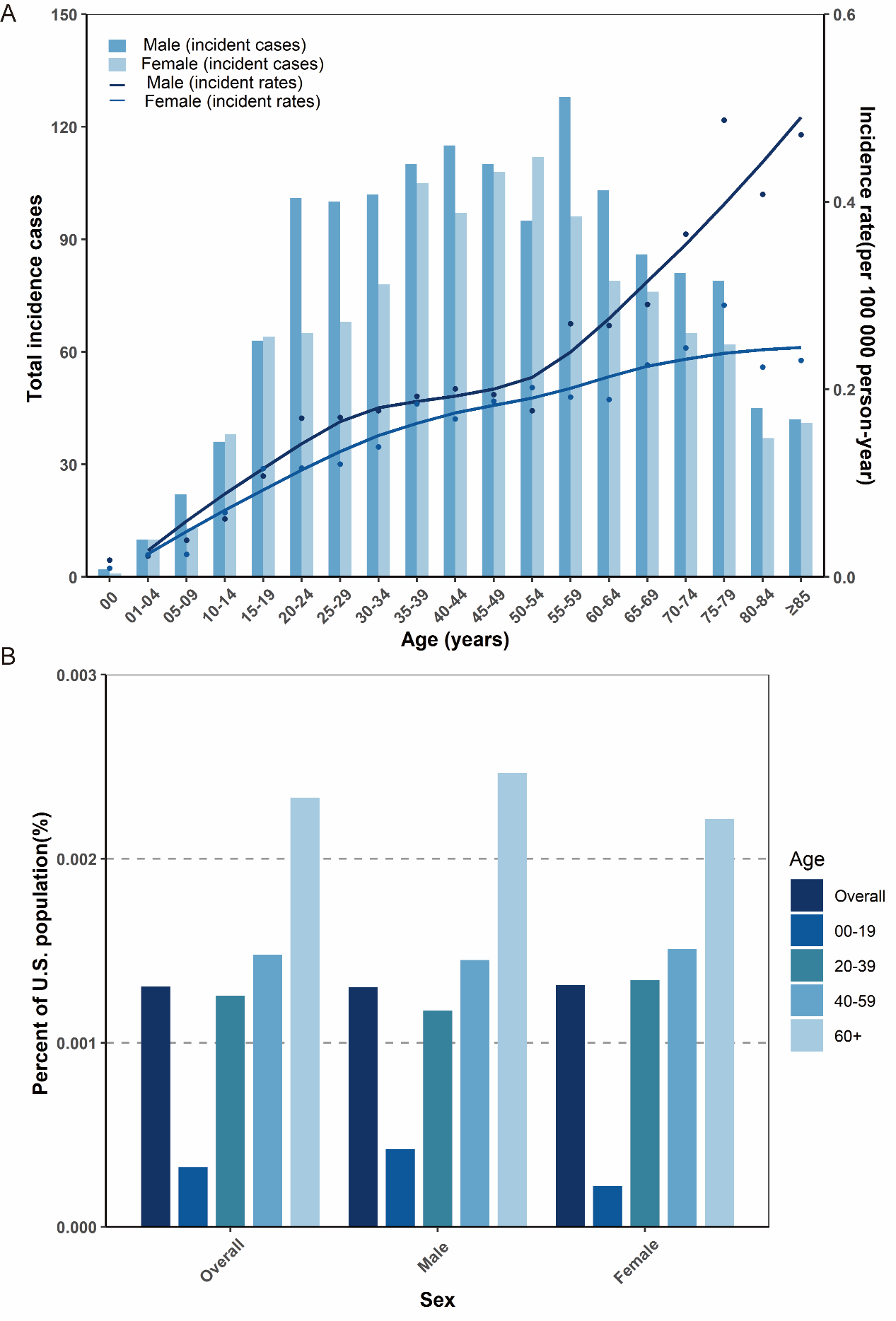


**Supplementary figure 3.** Survival curve for patients diagnosed with malignant peripheral nerve sheath tumor among different subgroups.

**
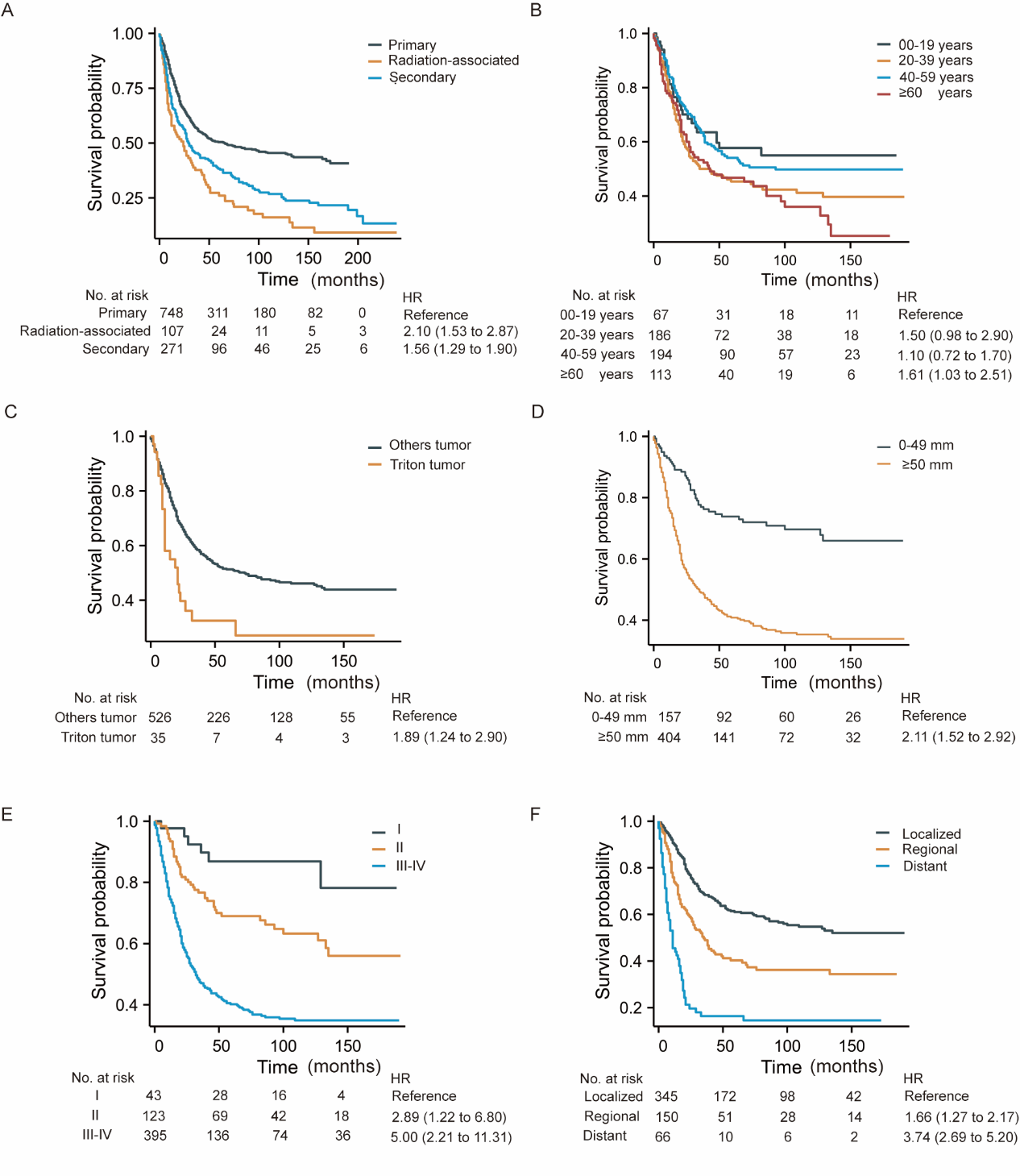
**

**Supplementary figure 4**. Forest plot for patients diagnosed with malignant peripheral nerve sheath tumor among different subgroups.


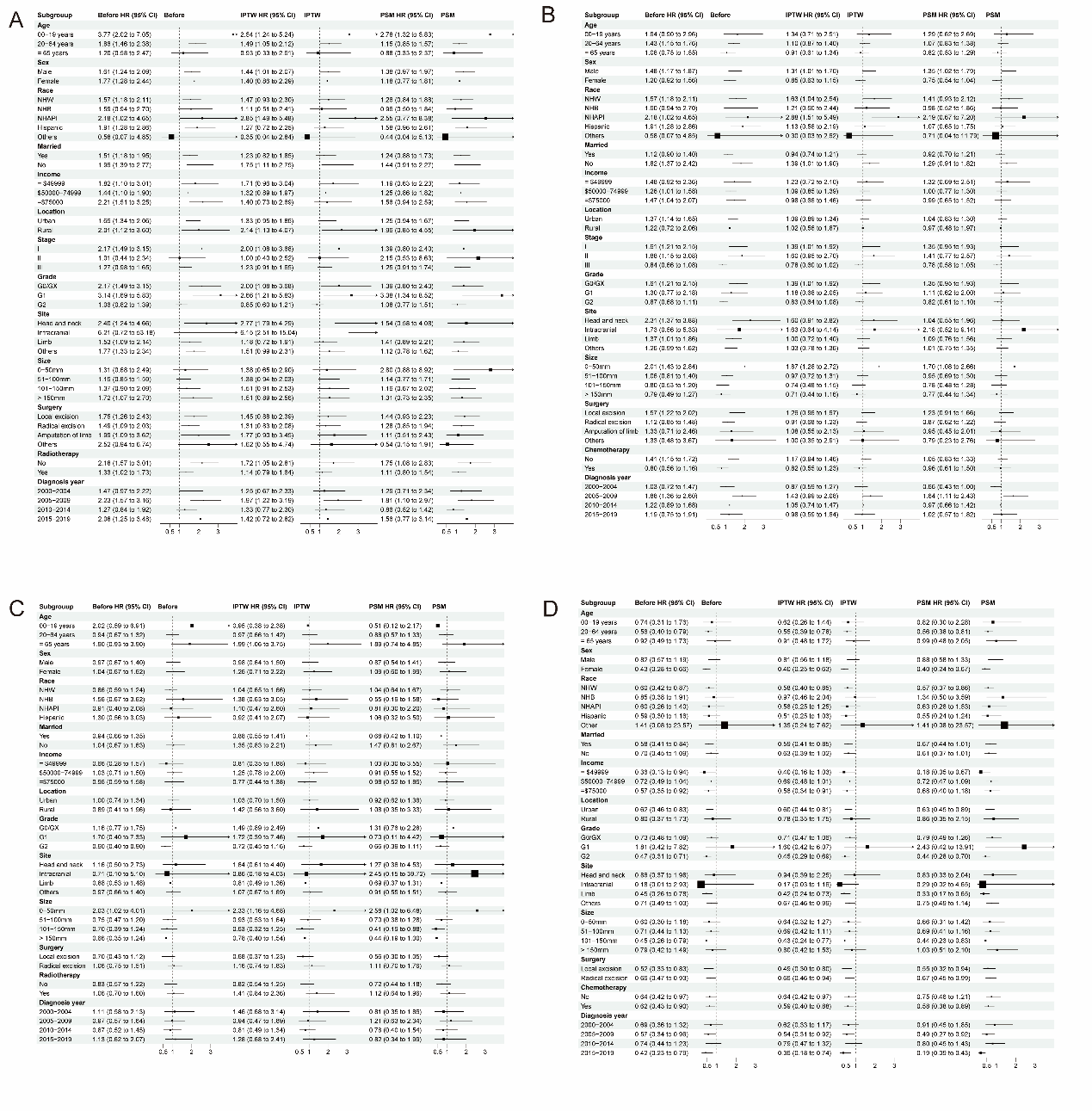


**Supplementary figure 5. Nomogram to predict the survival probabilities of patients with malignant peripheral nerve sheath tumor and the ROC curve of the nomogram using the training and validation sets.**

**
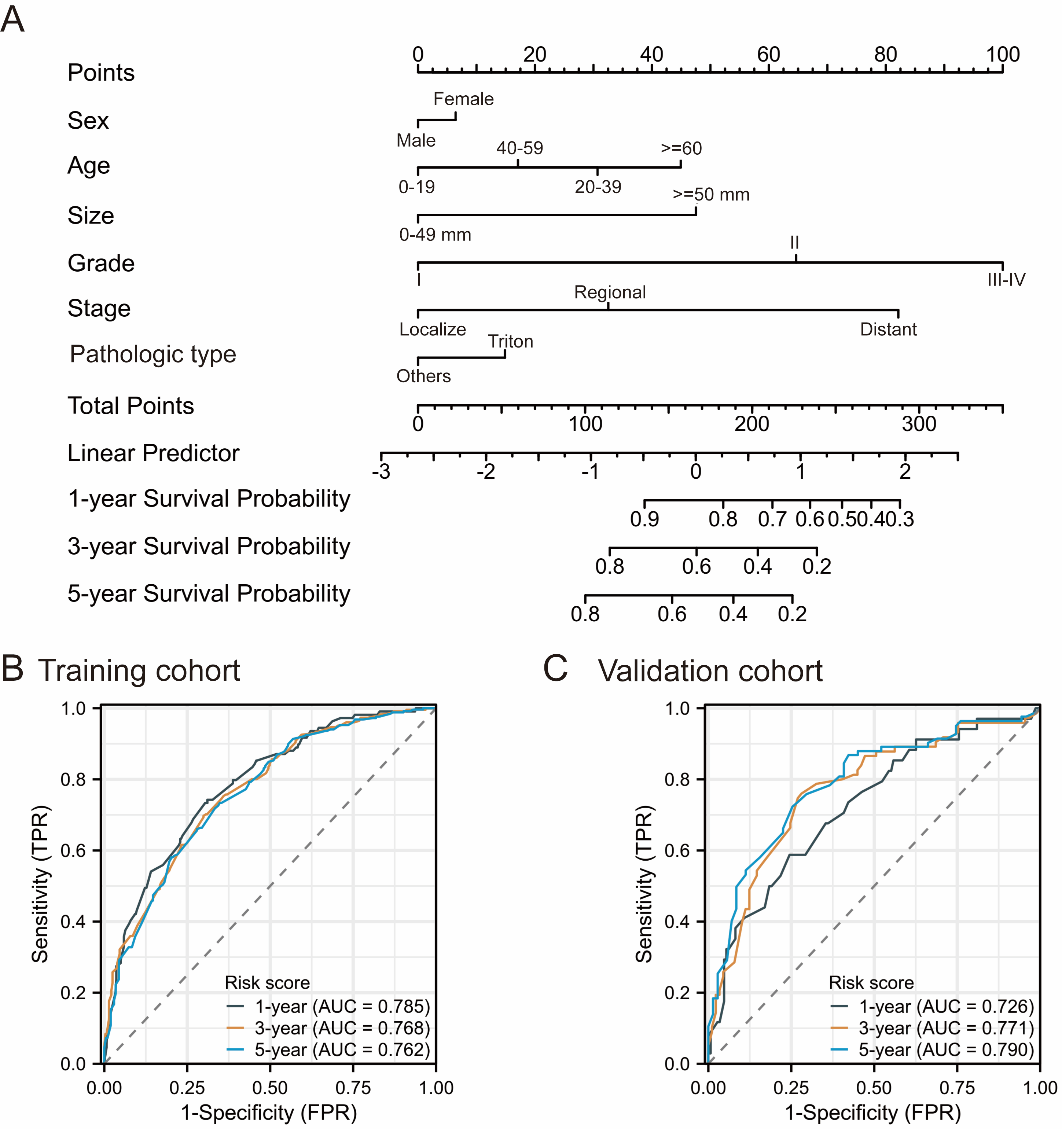
**

**Supplementary figure 6.** Calibration plot and decision curve analysis for the nomogram.

**
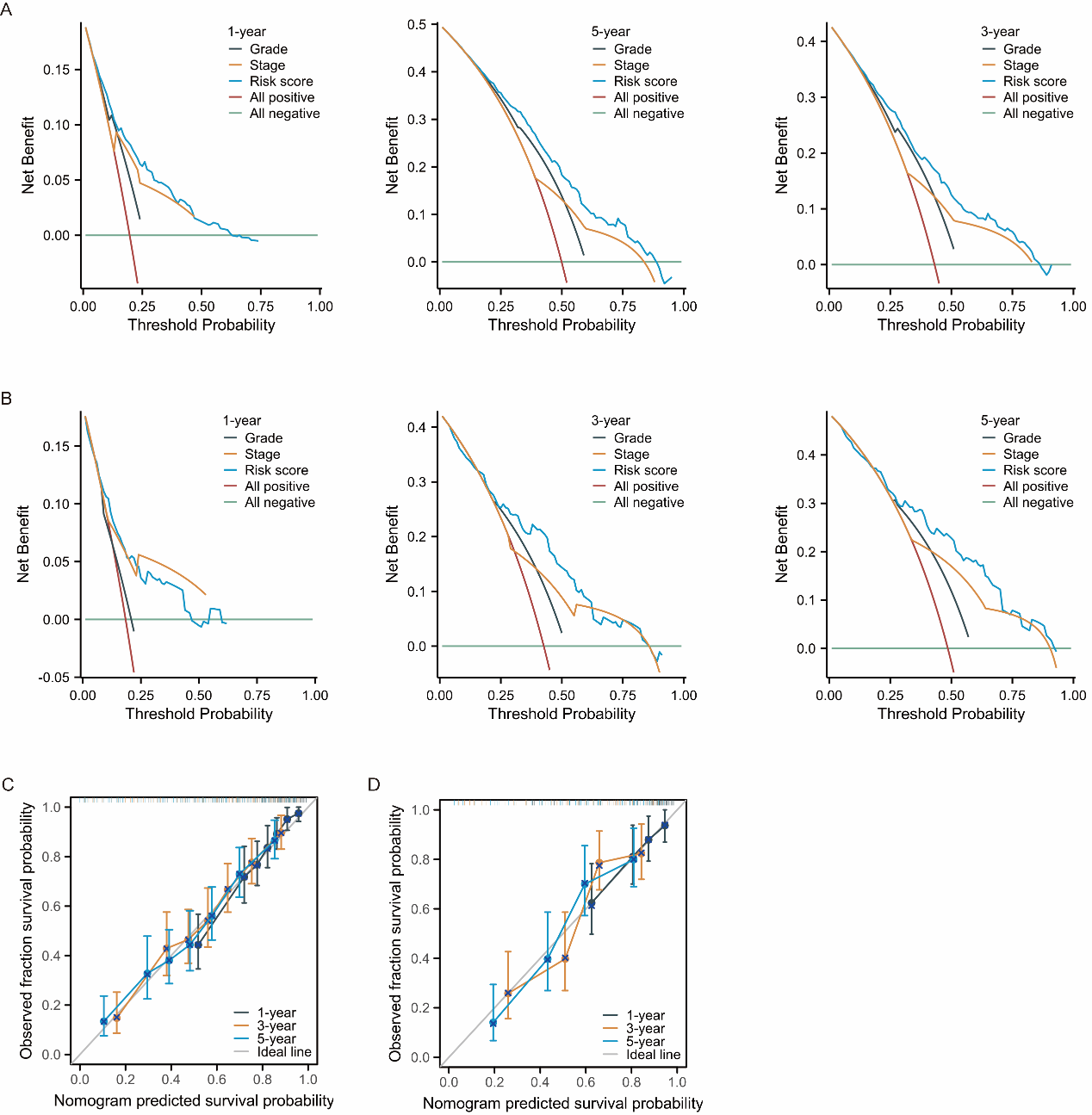
**

**Supplementary figure 7. Risk of developing subsequent primary cancers and cause of death among patients with malignant peripheral nerve sheath tumor**


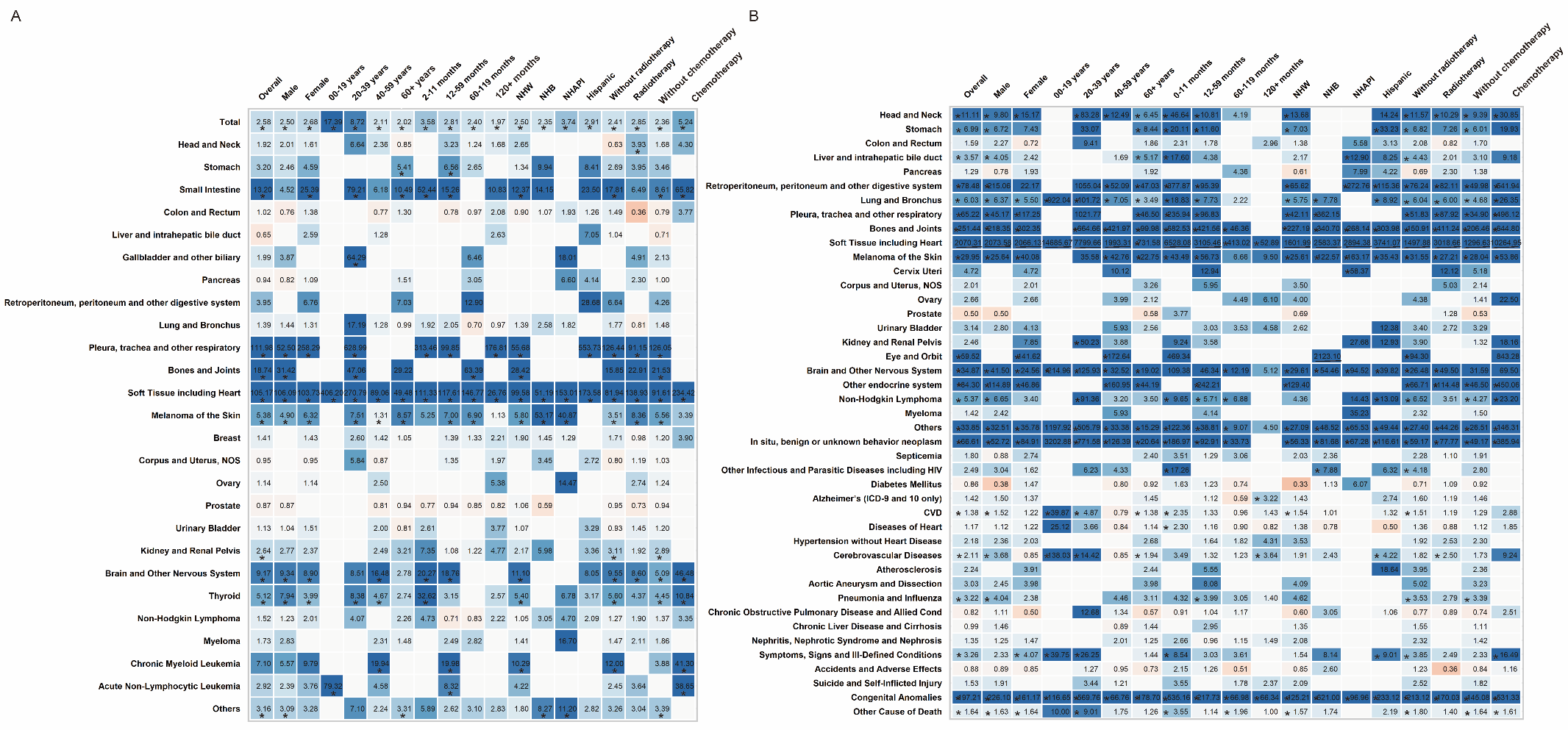


**Figure legend**

**Supplementary figure 1. Flowchart of study inclusion and exclusion for the nomogram.**

**Supplementary figure 2. Incidence and prevalence of malignant peripheral nerve sheath tumor. (A)** Incidence rates by age and sex are calculated from the 17 SEER registries. Filled circles represent observed age-adjusted rates while filled bars represent observed count. The curve was fitted by the incidence and age groups, where those aged 00 years were not included due to the small sample size. **(B)** 20-year limited-duration prevalence by sex and age during 2000 to 2019 was estimated in the 8 SEER registries.

**Supplementary figure 3**. **Forest plot for patients diagnosed with malignant peripheral nerve sheath tumor among different subgroups.** (A) Chemotherapy for patients at stage I-III. (B) Radiotherapy for patients at stage I-III. (C) Chemotherapy for patients at stage IV. (D) Radiotherapy for patients at stage IV.

**Supplementary figure 4. Survival curve for patients diagnosed with malignant peripheral nerve sheath tumor among different subgroups.** (A) Sequence of tumor. (B) Age. (C) Pathologic type. (D) Tumor size. (E) Tumor stage. (F) Tumor grade. HR: Hazard ratio.

**Supplementary figure 5. Nomogram to predict the survival probabilities of patients with malignant peripheral nerve sheath tumor and the ROC curve of the nomogram using the training and validation sets. (A)** Points for sex, age, tumor size, tumor grade, stage and histologic type are obtained by drawing a line upward from the corresponding values to the “Points” line. The sum of the points of these factors is located on the “Total points” line, and a line projected down to the bottom scales determine the probabilities of 1-year, 3-year and 5-year overall survival. Receiver operating characteristics (ROC) curve for the Nomogram in the training cohort **(B)** and the validation cohort **(C)**. Area under the curves of the models to predict 1-year, 3year and 5-year survival rates was estimated respectively.

**Supplementary figure 6. Calibration plot and decision curve analysis for the nomogram.** (A) Calibration curve of the nomogram for 1-year, 3-year and 5-year survival in training cohort. (B) Calibration curve of the nomogram for 1-year, 3-year and 5-year survival in validation cohort. (C) Decision curve analysis of the nomogram in training cohort. (D) Decision curve analysis of the nomogram in validation cohort.

**Supplementary figure 7. Risk of developing subsequent primary cancers and cause of death among patients with malignant peripheral nerve sheath tumor. (A)** demonstrated the standardized incidence ratio (SIR) of developing subsequent primary cancers among non-metastasis MPNST. **(B)** showed the standardized mortality ratio (SMR) of death by cause among patients with malignant peripheral nerve sheath tumor.

**Abbreviation:** NHW, non-Hispanic White; NHB, non-Hispanic Black; NHAPI, non-Hispanic Asian and Pacific Islander.

Notes:

The analyses of race limited in non-Hispanic White, non-Hispanic Black, non-Hispanic Asian and Pacific Islander and Hispanic. Participants with non-Hispanic American Indian/Alaska Native and non-Hispanic unknown were excluded. 12 out of 39 SPCs and 22 out of 67 COD were not shown in the figure due to the non-observed event. “*” and “_” indicated the SIR or SMR was significantly elevated.
