# Supplementary material for: Epidemiologic and survival analysis of malignant peripheral nerve sheath tumors: a retrospective cohort study
Source: Int J Surg. 2024 Jun 27;110(12):8210–4. doi: 10.1097/JS9.0000000000001756 (PMC11634120; doi:10.1097/JS9.0000000000001756)
Supplement: SUPPLEMENTARY MATERIAL [file js9-110-8210-s003.pdf]

eTable38. The risk of developing SPC among survivors diagnosed with primary malignant peripheral nerve sheath tumor by sex during 2000-2019 in 17 SEER registers.

SPC	Male				Female			
	Obs.	SIR (95% CI)	Absolute excess incidence per 10,000 person-year	Relative risk (95%CI)	Obs.	SIR (95% CI)	Absolute excess incidence per 10,000 person-year	Relative risk (95%CI)
Total	107 (100)	2.51 (2.05, 3.03)	124.94 (85.53, 164.35)	1 (Reference)	86 (100)	2.68 (2.15, 3.31)	113.79 (75.46, 152.13)	0.9 (0.67, 1.2)
Total (excluded MPNST)	81 (75.70)	1.92 (1.53, 2.38)	74.4 (40.12, 108.69)	1 (Reference)	72 (83.72)	2.25 (1.76, 2.83)	(116.78, 186.93)	..
Head and Neck	4 (3.74)	2.01 (0.55, 5.15)	3.91 (-3.71, 11.53)	1 (Reference)	1 (1.16)	1.61 (0.04, 8.97)	0.8 (-3.33, 4.93)	0.74 (0.08, 6.71)
Esophagus	0 (0)	0 (0, 5.83)	-1.23 (-1.23, -1.23)	1 (Reference)	0 (0)	0 (0, 25.99)	-0.3 (-0.3, -0.3)	..
Stomach	2 (1.87)	2.46 (0.3, 8.89)	2.31 (-3.08, 7.69)	1 (Reference)	2 (2.33)	4.59 (0.56, 16.57)	3.3 (-2.55, 9.14)	1.94 (0.27, 13.78)
Small Intestine	1 (0.93)	4.52 (0.11, 25.2)	1.51 (-2.3, 5.32)	1 (Reference)	4 (4.65)	25.39 (6.92, 65.01)	8.1 (-0.16, 16.37)	5 (0.56, 45.02)
Colon and Rectum	3 (2.8)	0.76 (0.16, 2.23)	-1.82 (-8.42, 4.78)	1 (Reference)	4 (4.65)	1.38 (0.38, 3.54)	2.34 (-5.93, 10.6)	1.81 (0.41, 8.11)
Anus, Anal Canal and Anorectum	0 (0)	0 (0, 29.31)	-0.25 (-0.25, -0.25)	1 (Reference)	0 (0)	0 (0, 21.56)	-0.36 (-0.36, -0.36)	..
Liver and intrahepatic bile duct	0 (0)	0 (0, 3.2)	-2.24 (-2.24, -2.24)	1 (Reference)	1 (1.16)	2.59 (0.07, 14.45)	1.3 (-2.84, 5.43)	..
Gallbladder and other biliary	1 (0.93)	3.87 (0.1, 21.58)	1.44 (-2.37, 5.25)	1 (Reference)	0 (0)	0 (0, 15.12)	-0.51 (-0.51, -0.51)	..
Pancreas	1 (0.93)	0.82 (0.02, 4.58)	-0.42 (-4.23, 3.39)	1 (Reference)	1 (1.16)	1.09 (0.03, 6.05)	0.17 (-3.97, 4.3)	1.29 (0.08, 20.62)
Retroperitoneum, peritoneum and other digestive system	0 (0)	0 (0, 35.14)	-0.2 (-0.2, -0.2)	1 (Reference)	1 (1.16)	6.76 (0.17, 37.67)	1.8 (-2.34, 5.93)	..
Lung and Bronchus	8 (7.48)	1.44 (0.62, 2.84)	4.78 (-6, 15.55)	1 (Reference)	5 (5.81)	1.31 (0.43, 3.05)	2.49 (-6.76, 11.73)	0.78 (0.25, 2.42)
Pleura, trachea and other respiratory	1 (0.93)	52.5 (1.33, 292.53)	1.91 (-1.9, 5.72)	1 (Reference)	2 (2.33)	258.29 (31.28, 933.05)	4.2 (-1.64, 10.05)	256.16 (22.53, 2912.28)
Bones and Joints	2 (1.87)	31.42 (3.81, 113.49)	3.76 (-1.62, 9.15)	1 (Reference)	0 (0)	0 (0, 85.65)	-0.09 (-0.09, -0.09)	..
Soft Tissue including Heart	32 (29.91)	106.09 (72.56, 149.76)	61.61 (40.06, 83.16)	1 (Reference)	20 (23.26)	103.73 (63.36, 160.21)	41.78 (23.29, 60.26)	0.85 (0.49, 1.49)
Soft Tissue including Heart (excluded MPNST)	7 (6.54)	23.18 (9.32, 47.76)	13.02 (2.94, 23.1)	1 (Reference)	7 (8.14)	36.27 (14.58, 74.73)	14.76 (3.83, 25.7)	..
Melanoma of the Skin	12 (11.21)	4.9 (2.53, 8.57)	18.57 (5.37, 31.76)	1 (Reference)	8 (9.3)	6.32 (2.73, 12.44)	14.2 (2.51, 25.89)	1.43 (0.57, 3.62)
Breast	0 (0)	0 (0, 37.47)	-0.19 (-0.19, -0.19)	1 (Reference)	14 (16.28)	1.43 (0.78, 2.39)	8.83 (-6.64, 24.3)	..
Cervix Uteri	..	..	..	1 (Reference)	0 (0)	0 (0, 7.18)	-1.08 (-1.08, -1.08)	..
Corpus and Uterus, NOS	..	..	..	1 (Reference)	2 (2.33)	0.95 (0.12, 3.44)	-0.21 (-6.06, 5.64)	..
Ovary	..	..	..	1 (Reference)	1 (1.16)	1.14 (0.03, 6.36)	0.26 (-3.87, 4.39)	..
Vagina, vulva and other female genital organs	..	..	..	1 (Reference)	0 (0)	0 (0, 10.29)	-0.76 (-0.76, -0.76)	..
Prostate	10 (9.35)	0.87 (0.42, 1.6)	-2.91 (-14.96, 9.14)	1 (Reference)	..	..	..	..
Testis	0 (0)	0 (0, 11.94)	-0.6 (-0.6, -0.6)	1 (Reference)	..	..	..	..
Penis and other male genital organs	0 (0)	0 (0, 38.69)	-0.19 (-0.19, -0.19)	1 (Reference)	..	..	..	..
Urinary Bladder	3 (2.8)	1.05 (0.22, 3.06)	0.25 (-6.35, 6.85)	1 (Reference)	1 (1.16)	1.51 (0.04, 8.41)	0.71 (-3.42, 4.84)	1.41 (0.15, 13.54)
Kidney and Renal Pelvis	5 (4.67)	2.77 (0.9, 6.46)	6.21 (-2.31, 14.73)	1 (Reference)	2 (2.33)	2.37 (0.29, 8.55)	2.44 (-3.41, 8.28)	0.88 (0.17, 4.54)
Ureter and other urinary organs	0 (0)	0 (0, 33.37)	-0.22 (-0.22, -0.22)	1 (Reference)	0 (0)	0 (0, 80.23)	-0.1 (-0.1, -0.1)	..
Eye and Orbit	0 (0)	0 (0, 49.54)	-0.15 (-0.15, -0.15)	1 (Reference)	0 (0)	0 (0, 79.16)	-0.1 (-0.1, -0.1)	..
Brain and Other Nervous System	5 (4.67)	9.34 (3.03, 21.79)	8.68 (0.16, 17.2)	1 (Reference)	3 (3.49)	8.9 (1.84, 26.02)	5.62 (-1.54, 12.78)	0.97 (0.23, 4.07)
Thyroid	4 (3.74)	7.94 (2.16, 20.32)	6.8 (-0.82, 14.41)	1 (Reference)	5 (5.81)	3.99 (1.3, 9.31)	7.9 (-1.34, 17.15)	0.42 (0.11, 1.59)
Other endocrine system	0 (0)	0 (0, 69.03)	-0.1 (-0.1, -0.1)	1 (Reference)	0 (0)	0 (0, 85.8)	-0.09 (-0.09, -0.09)	..
Hodgkin Lymphoma	0 (0)	0 (0, 20.28)	-0.35 (-0.35, -0.35)	1 (Reference)	0 (0)	0 (0, 30.34)	-0.26 (-0.26, -0.26)	..
Non-Hodgkin Lymphoma	3 (2.8)	1.23 (0.25, 3.58)	1.08 (-5.52, 7.68)	1 (Reference)	3 (3.49)	2.01 (0.41, 5.87)	3.18 (-3.98, 10.34)	1.61 (0.32, 7.96)
Myeloma	2 (1.87)	2.83 (0.34, 10.23)	2.52 (-2.87, 7.9)	1 (Reference)	0 (0)	0 (0, 8.21)	-0.95 (-0.95, -0.95)	..
Acute Lymphocytic Leukemia	0 (0)	0 (0, 53.68)	-0.13 (-0.13, -0.13)	1 (Reference)	0 (0)	0 (0, 78.08)	-0.1 (-0.1, -0.1)	..
Chronic Myeloid Leukemia	1 (0.93)	5.57 (0.14, 31.03)	1.6 (-2.21, 5.4)	1 (Reference)	1 (1.16)	9.79 (0.25, 54.54)	1.89 (-2.24, 6.03)	1.58 (0.1, 25.31)
Acute Non-Lymphocytic Leukemia	1 (0.93)	2.39 (0.06, 13.34)	1.13 (-2.68, 4.94)	1 (Reference)	1 (1.16)	3.76 (0.1, 20.94)	1.55 (-2.59, 5.68)	1.16 (0.07, 18.69)
Others	6 (5.61)	3.09 (1.14, 6.73)	7.89 (-1.44, 17.22)	1 (Reference)	4 (4.65)	3.28 (0.89, 8.4)	5.86 (-2.4, 14.13)	1.05 (0.3, 3.74)

eTable39. The risk of developing SPC among survivors diagnosed with primary malignant peripheral nerve sheath tumor by age during 2000-2019 in 17 SEER registers.

SPC	0-19 years				20-39 years				40-59 years				≥ 60 years			
	Obs.	SIR (95% CI)	Absolute excess incidence per 10,000 person-year	Relative risk (95%CI)	Obs.	SIR (95% CI)	Absolute excess incidence per 10,000 person-year	Relative risk (95%CI)	Obs.	SIR (95% CI)	Absolute excess incidence per 10,000 person-year	Relative risk (95%CI)	Obs.	SIR (95% CI)	Absolute excess incidence per 10,000 person-year	Relative risk (95%CI)
Total	7 (100)	17.39 (6.99, 35.83)	54.83 (11.73, 97.92)	1 (Reference)	43 (100)	8.72 (6.31, 11.75)	126.79 (83.99, 169.6)	0.51 (0.23, 1.13)	64 (100)	2.11 (1.62, 2.69)	88.5 (47.25, 129.75)	0.12 (0.05, 0.25)	79 (100)	2.02 (1.6, 2.52)	212.48 (119.77, 305.19)	0.1 (0.05, 0.22)
Total (excluded MPNST)	4 (57.14)	9.93 (2.70, 25.41)	29.89 (-2.68, 62.47)	1 (Reference)	25 (58.14)	4.67 (2.96, 7.00)	66.84 (34.2, 99.48)	..	51 (79.69)	1.68 (1.25, 2.21)	54.3 (17.48, 91.12)	..	74 (93.67)	1.89 (1.49, 2.38)	185.87 (96.14, 275.6)	..
Head and Neck	0 (0)	0 (0, 449.96)	-0.07 (-0.07, -0.07)	1 (Reference)	1 (2.33)	6.64 (0.17, 36.99)	2.83 (-3.7, 9.36)	..	3 (4.69)	2.36 (0.49, 6.89)	4.55 (-4.38, 13.48)	..	1 (1.27)	0.85 (0.02, 4.72)	-0.96 (-11.39, 9.47)	..
Esophagus	0 (0)	0 (0, 12556.3)	0 (0, 0)	1 (Reference)	0 (0)	0 (0, 176.97)	-0.07 (-0.07, -0.07)	..	0 (0)	0 (0, 11.83)	-0.82 (-0.82, -0.82)	..	0 (0)	0 (0, 8.35)	-2.35 (-2.35, -2.35)	..
Stomach	0 (0)	0 (0, 1520.02)	-0.02 (-0.02, -0.02)	1 (Reference)	0 (0)	0 (0, 56.39)	-0.22 (-0.22, -0.22)	..	0 (0)	0 (0, 8.36)	-1.16 (-1.16, -1.16)	..	4 (5.06)	5.41 (1.47, 13.84)	17.35 (-3.51, 38.21)	..
Small Intestine	0 (0)	0 (0, 4258.75)	-0.01 (-0.01, -0.01)	1 (Reference)	2 (4.65)	79.21 (9.59, 286.14)	6.58 (-2.65, 15.81)	..	1 (1.56)	6.18 (0.16, 34.42)	2.21 (-2.95, 7.36)	..	2 (2.53)	10.49 (1.27, 37.89)	9.63 (-5.12, 24.38)	..
Colon and Rectum	0 (0)	0 (0, 220.53)	-0.14 (-0.14, -0.14)	1 (Reference)	0 (0)	0 (0, 9.42)	-1.3 (-1.3, -1.3)	..	2 (3.13)	0.77 (0.09, 2.79)	-1.55 (-8.85, 5.74)	..	5 (6.33)	1.31 (0.42, 3.05)	6.23 (-17.1, 29.55)	..
Anus, Anal Canal and Anorectum	0 (0)	0 (0, 8474.96)	0 (0, 0)	1 (Reference)	0 (0)	0 (0, 148.47)	-0.08 (-0.08, -0.08)	..	0 (0)	0 (0, 23.57)	-0.41 (-0.41, -0.41)	..	0 (0)	0 (0, 32.02)	-0.61 (-0.61, -0.61)	..
Liver and intrahepatic bile duct	0 (0)	0 (0, 1462.07)	-0.02 (-0.02, -0.02)	1 (Reference)	0 (0)	0 (0, 80.33)	-0.15 (-0.15, -0.15)	..	1 (1.56)	1.28 (0.03, 7.13)	0.58 (-4.58, 5.73)	..	0 (0)	0 (0, 5.2)	-3.78 (-3.78, -3.78)	..
Gallbladder and other biliary	0 (0)	0 (0, 13231.62)	0 (0, 0)	1 (Reference)	1 (2.33)	64.29 (1.63, 358.2)	3.28 (-3.25, 9.81)	..	0 (0)	0 (0, 22.26)	-0.44 (-0.44, -0.44)	..	0 (0)	0 (0, 11.51)	-1.71 (-1.71, -1.71)	..
Pancreas	0 (0)	0 (0, 1832.54)	-0.02 (-0.02, -0.02)	1 (Reference)	0 (0)	0 (0, 60.58)	-0.2 (-0.2, -0.2)	..	0 (0)	0 (0, 4.94)	-1.97 (-1.97, -1.97)	..	2 (2.53)	1.51 (0.18, 5.44)	3.58 (-11.17, 18.33)	..
Retroperitoneum, peritoneum and other digestive system	0 (0)	0 (0, 3465.76)	-0.01 (-0.01, -0.01)	1 (Reference)	0 (0)	0 (0, 279.57)	-0.04 (-0.04, -0.04)	..	0 (0)	0 (0, 38.29)	-0.25 (-0.25, -0.25)	..	1 (1.27)	7.03 (0.18, 39.16)	4.57 (-5.87, 15)	..
Lung and Bronchus	0 (0)	0 (0, 1135.14)	-0.03 (-0.03, -0.03)	1 (Reference)	3 (6.98)	17.19 (3.55, 50.23)	9.41 (-1.9, 20.72)	..	4 (6.25)	1.28 (0.35, 3.28)	2.3 (-8.01, 12.61)	..	6 (7.59)	0.99 (0.36, 2.16)	-0.33 (-25.87, 25.22)	..
Pleura, trachea and other respiratory	0 (0)	0 (0, 2388.11)	-0.01 (-0.01, -0.01)	1 (Reference)	3 (6.98)	628.99 (129.71, 1838.18)	9.98 (-1.33, 21.28)	..	0 (0)	0 (0, 409.58)	-0.02 (-0.02, -0.02)	..	0 (0)	0 (0, 321.65)	-0.06 (-0.06, -0.06)	..
Bones and Joints	0 (0)	0 (0, 286.36)	-0.11 (-0.11, -0.11)	1 (Reference)	1 (2.33)	47.06 (1.19, 262.21)	3.26 (-3.27, 9.79)	..	0 (0)	0 (0, 96.13)	-0.1 (-0.1, -0.1)	..	1 (1.27)	29.22 (0.74, 162.81)	5.14 (-5.29, 15.57)	..
Soft Tissue including Heart	6 (85.71)	406.2 (149.07, 884.13)	49.74 (9.84, 89.64)	1 (Reference)	18 (41.86)	270.79 (160.48, 427.96)	59.73 (32.03, 87.42)	0.68 (0.27, 1.71)	17 (26.56)	89.06 (51.88, 142.6)	44.22 (22.96, 65.48)	0.22 (0.09, 0.55)	11 (13.92)	49.48 (24.7, 88.53)	57.36 (22.76, 91.95)	0.11 (0.04, 0.3)
Soft Tissue including Heart (excluded MPNST)	3 (42.86)	200 (41.24, 584.48)	24.81 (-3.41, 53.02)	1 (Reference)	2 (4.65)	30.30 (3.67, 109.47)	6.44 (-2.79, 15.67)	..	4 (6.25)	20.94 (5.71, 53.62)	10.02 (-0.29, 20.33)	..	6 (7.59)	27.03 (9.92, 58.83)	30.75 (5.2, 56.3)	..
Melanoma of the Skin	0 (0)	0 (0, 112.91)	-0.27 (-0.27, -0.27)	1 (Reference)	3 (6.98)	7.51 (1.55, 21.94)	8.66 (-2.65, 19.97)	..	2 (3.13)	1.31 (0.16, 4.72)	1.23 (-6.06, 8.52)	..	15 (18.99)	8.57 (4.8, 14.14)	70.51 (30.12, 110.91)	..
Breast	0 (0)	0 (0, 208.51)	-0.15 (-0.15, -0.15)	1 (Reference)	3 (6.98)	2.6 (0.54, 7.6)	6.15 (-5.15, 17.46)	..	7 (10.94)	1.42 (0.57, 2.92)	5.43 (-8.21, 19.07)	..	4 (5.06)	1.05 (0.29, 2.69)	1.03 (-19.83, 21.89)	..
Cervix Uteri	0 (0)	0 (0, 424.75)	-0.15 (-0.15, -0.15)	1 (Reference)	0 (0)	0 (0, 22.4)	-1.17 (-1.17, -1.17)	..	0 (0)	0 (0, 15.57)	-1.28 (-1.28, -1.28)	..	0 (0)	0 (0, 35.65)	-1.14 (-1.14, -1.14)	..
Corpus and Uterus, NOS	0 (0)	0 (0, 968.24)	-0.07 (-0.07, -0.07)	1 (Reference)	1 (2.33)	5.84 (0.15, 32.52)	5.87 (-8.02, 19.76)	..	1 (1.56)	0.87 (0.02, 4.84)	-0.82 (-11.4, 9.77)	..	0 (0)	0 (0, 4.77)	-8.54 (-8.54, -8.54)	..
Ovary	0 (0)	0 (0, 406.38)	-0.16 (-0.16, -0.16)	1 (Reference)	0 (0)	0 (0, 39.78)	-0.66 (-0.66, -0.66)	..	1 (1.56)	2.5 (0.06, 13.93)	3.24 (-7.34, 13.82)	..	0 (0)	0 (0, 9.84)	-4.14 (-4.14, -4.14)	..
Vagina, vulva and other female genital organs	0 (0)	0 (0, 2404.13)	-0.03 (-0.03, -0.03)	1 (Reference)	0 (0)	0 (0, 123.99)	-0.21 (-0.21, -0.21)	..	0 (0)	0 (0, 24.58)	-0.81 (-0.81, -0.81)	..	0 (0)	0 (0, 20.8)	-1.96 (-1.96, -1.96)	..
Prostate	0 (0)	0 (0, 19008.24)	0 (0, 0)	1 (Reference)	0 (0)	0 (0, 26.58)	-0.87 (-0.87, -0.87)	..	4 (6.25)	0.81 (0.22, 2.07)	-4.87 (-24.98, 15.25)	..	6 (7.59)	0.94 (0.34, 2.04)	-4.2 (-53.56, 45.16)	..
Testis	0 (0)	0 (0, 88.99)	-0.66 (-0.66, -0.66)	1 (Reference)	0 (0)	0 (0, 20.62)	-1.12 (-1.12, -1.12)	..	0 (0)	0 (0, 47.93)	-0.4 (-0.4, -0.4)	..	0 (0)	0 (0, 315.28)	-0.12 (-0.12, -0.12)	..
Penis and other male genital organs	0 (0)	0 (0, 12994.96)	0 (0, 0)	1 (Reference)	0 (0)	0 (0, 715.74)	-0.03 (-0.03, -0.03)	..	0 (0)	0 (0, 108.16)	-0.18 (-0.18, -0.18)	..	0 (0)	0 (0, 66.1)	-0.57 (-0.57, -0.57)	..
Urinary Bladder	0 (0)	0 (0 to 1973.1)	-0.02 (-0.02, -0.02)	1 (Reference)	0 (0)	0 (0, 57.56)	-0.21 (-0.21, -0.21)	..	2 (3.13)	2 (0.24, 7.21)	2.63 (-4.67, 9.92)	..	2 (2.53)	0.81 (0.1, 2.93)	-2.47 (-17.22, 12.28)	..
Kidney and Renal Pelvis	0 (0)	0 (0 to 579.22)	-0.06 (-0.06, -0.06)	1 (Reference)	0 (0)	0 (0, 19.37)	-0.63 (-0.63, -0.63)	..	3 (4.69)	2.49 (0.51, 7.27)	4.72 (-4.21, 13.65)	..	4 (5.06)	3.21 (0.87, 8.21)	14.65 (-6.21, 35.51)	..
Ureter and other urinary organs	0 (0)	0 (0 to 95850.23)	0 (0, 0)	1 (Reference)	0 (0)	0 (0, 1770.99)	-0.01 (-0.01, -0.01)	..	0 (0)	0 (0, 92.02)	-0.11 (-0.11, -0.11)	..	0 (0)	0 (0, 32.27)	-0.61 (-0.61, -0.61)	..
Eye and Orbit	0 (0)	0 (0 to 3656.87)	-0.01 (-0.01, -0.01)	1 (Reference)	0 (0)	0 (0, 342.94)	-0.04 (-0.04, -0.04)	..	0 (0)	0 (0, 70.23)	-0.14 (-0.14, -0.14)	..	0 (0)	0 (0, 65.52)	-0.3 (-0.3, -0.3)	..
Brain and Other Nervous System	0 (0)	0 (0 to 157.39)	-0.26 (-0.26, -0.26)	1 (Reference)	1 (2.33)	8.51 (0.22, 47.44)	2.94 (-3.59, 9.47)	..	6 (9.38)	16.48 (6.05, 35.86)	14.83 (2.2, 27.46)	..	1 (1.27)	2.78 (0.07, 15.5)	3.41 (-7.02, 13.84)	..
Thyroid	0 (0)	0 (0 to 73.35)	-0.48 (-0.48, -0.48)	1 (Reference)	4 (9.3)	8.38 (2.28, 21.45)	11.73 (-1.32, 24.79)	..	4 (6.25)	4.67 (1.27, 11.96)	8.27 (-2.04, 18.58)	..	1 (1.27)	2.74 (0.07, 15.27)	3.38 (-7.05, 13.81)	..
Other endocrine system	0 (0)	0 (0 to 1398.48)	-0.03 (-0.03, -0.03)	1 (Reference)	0 (0)	0 (0, 284.89)	-0.04 (-0.04, -0.04)	..	0 (0)	0 (0, 84.29)	-0.12 (-0.12, -0.12)	..	0 (0)	0 (0, 102.47)	-0.19 (-0.19, -0.19)	..
Hodgkin Lymphoma	0 (0)	0 (0 to 125.24)	-0.3 (-0.3, -0.3)	1 (Reference)	0 (0)	0 (0, 37.99)	-0.32 (-0.32, -0.32)	..	0 (0)	0 (0, 37.34)	-0.26 (-0.26, -0.26)	..	0 (0)	0 (0, 51.47)	-0.38 (-0.38, -0.38)	..
Non-Hodgkin Lymphoma	0 (0)	0 (0 to 164.59)	-0.23 (-0.23, -0.23)	1 (Reference)	1 (2.33)	4.07 (0.1, 22.66)	2.51 (-4.02, 9.04)	..	0 (0)	0 (0, 2.53)	-3.83 (-3.83, -3.83)	..	5 (6.33)	2.26 (0.74, 5.28)	14.85 (-8.47, 38.18)	..
Myeloma	0 (0)	0 (0 to 6241.7)	-0.01 (-0.01, -0.01)	1 (Reference)	0 (0)	0 (0, 84.95)	-0.15 (-0.15, -0.15)	..	1 (1.56)	2.31 (0.06, 12.86)	1.49 (-3.67, 6.65)	..	1 (1.27)	1.48 (0.04, 8.22)	1.72 (-8.72, 12.15)	..
Acute Lymphocytic Leukemia	0 (0)	0 (0 to 252.17)	-0.18 (-0.18, -0.18)	1 (Reference)	0 (0)	0 (0, 150.64)	-0.08 (-0.08, -0.08)	..	0 (0)	0 (0, 91.9)	-0.11 (-0.11, -0.11)	..	0 (0)	0 (0, 125.25)	-0.16 (-0.16, -0.16)	..
Chronic Myeloid Leukemia	0 (0)	0 (0 to 966.88)	-0.04 (-0.04, -0.04)	1 (Reference)	0 (0)	0 (0, 109.04)	-0.11 (-0.11, -0.11)	..	2 (3.13)	19.94 (2.42, 72.03)	5 (-2.29, 12.29)	..	0 (0)	0 (0, 25.77)	-0.76 (-0.76, -0.76)	..
Acute Non-Lymphocytic Leukemia	1 (14.29)	101.81 (2.58 to 567.27)	8.21 (-8.08, 24.49)	1 (Reference)	0 (0)	0 (0, 67.56)	-0.18 (-0.18, -0.18)	..	1 (1.56)	4.58 (0.12, 25.5)	2.06 (-3.1, 7.21)	0.07 (0, 1.07)	0 (0)	0 (0, 9.27)	-2.12 (-2.12, -2.12)	..
Others	0 (0)	0 (0 to 343.97)	-0.11 (-0.11, -0.11)	1 (Reference)	1 (2.33)	7.1 (0.18, 39.57)	2.86 (-3.67, 9.39)		2 (3.13)	2.24 (0.27, 8.08)	2.91 (-4.38, 10.2)	..	7 (8.86)	3.31 (1.33, 6.83)	26.01 (-1.58, 53.61)	

eTable40. The risk of developing SPC among survivors diagnosed with primary malignant peripheral nerve sheath tumor by latency during 2000-2019 in 17 SEER registers.

SPC	2-11 months				12-59 months				60-119 months				≥ 120 months			
	Obs.	SIR (95% CI)	Absolute excess incidence per 10,000 person-year	Relative risk (95%CI)	Obs.	SIR (95% CI)	Absolute excess incidence per 10,000 person-year	Relative risk (95%CI)	Obs.	SIR (95% CI)	Absolute excess incidence per 10,000 person-year	Relative risk (95%CI)	Obs.	SIR (95% CI)	Absolute excess incidence per 10,000 person-year	Relative risk (95%CI)
Total	29 (100)	3.58 (2.4, 5.14)	168.42 (83.34, 253.49)	1 (Reference)	76 (100)	2.81 (2.22, 3.52)	126.98 (82.69, 171.27)	0.76 (0.5, 1.17)	55 (100)	2.41 (1.81, 3.13)	112.24 (61.47, 163)	0.61 (0.38, 0.95)	33 (100)	1.97 (1.35, 2.76)	84.26 (25.75, 142.78)	0.42 (0.25, 0.7)
Total (excluded MPNST)	26 (89.66)	3.21 (2.10, 4.70)	144.23 (63.68, 224.79)	1 (Reference)	58 (76.32)	2.15 (1.63, 2.78)	80.32 (41.63, 119.01)		39 (70.91)	1.71 (1.21, 2.33)	56.35 (13.6, 99.1)		31 (93.93)	1.85 (1.25, 2.62)	73.87 (17.16, 130.58)	..
Head and Neck	0 (0)	0 (0, 13.27)	-2.24 (-2.24, -2.24)	1 (Reference)	3 (3.95)	3.23 (0.67, 9.45)	5.37 (-3.43, 14.17)	..	1 (1.82)	1.24 (0.03, 6.89)	0.67 (-6.18, 7.51)	..	1 (3.03)	1.68 (0.04, 9.35)	2.1 (-8.09, 12.28)	..
Esophagus	0 (0)	0 (0, 44.34)	-0.67 (-0.67, -0.67)	1 (Reference)	0 (0)	0 (0, 13.33)	-0.72 (-0.72, -0.72)	..	0 (0)	0 (0, 15.46)	-0.83 (-0.83, -0.83)	..	0 (0)	0 (0, 20.96)	-0.91 (-0.91, -0.91)	..
Stomach	0 (0)	0 (0, 27.26)	-1.09 (-1.09, -1.09)	1 (Reference)	3 (3.95)	6.56 (1.35, 19.16)	6.59 (-2.21, 15.39)	..	1 (1.82)	2.65 (0.07, 14.79)	2.18 (-4.67, 9.02)	..	0 (0)	0 (0, 13.22)	-1.45 (-1.45, -1.45)	..
Small Intestine	2 (6.9)	52.44 (6.35, 189.43)	15.81 (-6.53, 38.16)	1 (Reference)	2 (2.63)	15.26 (1.85, 55.12)	4.84 (-2.34, 12.03)	0.3 (0.04, 2.16)	0 (0)	0 (0, 31.51)	-0.41 (-0.41, -0.41)	..	1 (3.03)	10.83 (0.27, 60.33)	4.72 (-5.47, 14.9)	0.14 (0.01, 1.7)
Colon and Rectum	0 (0)	0 (0, 4.75)	-6.27 (-6.27, -6.27)	1 (Reference)	2 (2.63)	0.79 (0.1, 2.83)	-1.42 (-8.61, 5.76)	..	2 (3.64)	0.97 (0.12, 3.51)	-0.21 (-9.89, 9.47)	..	3 (9.09)	2.08 (0.43, 6.07)	8.09 (-9.56, 25.73)	..
Anus, Anal Canal and Anorectum	0 (0)	0 (0, 122.31)	-0.24 (-0.24, -0.24)	1 (Reference)	0 (0)	0 (0, 35.79)	-0.27 (-0.27, -0.27)	..	0 (0)	0 (0, 40.18)	-0.32 (-0.32, -0.32)	..	0 (0)	0 (0, 51.27)	-0.37 (-0.37, -0.37)	..
Liver and intrahepatic bile duct	0 (0)	0 (0, 24.44)	-1.22 (-1.22, -1.22)	1 (Reference)	0 (0)	0 (0, 7.02)	-1.36 (-1.36, -1.36)	..	0 (0)	0 (0, 7.63)	-1.69 (-1.69, -1.69)	..	1 (3.03)	2.63 (0.07, 14.67)	3.22 (-6.96, 13.41)	..
Gallbladder and other biliary	0 (0)	0 (0, 70.74)	-0.42 (-0.42, -0.42)	1 (Reference)	0 (0)	0 (0, 20.78)	-0.46 (-0.46, -0.46)	..	1 (1.82)	6.46 (0.16, 36.01)	2.95 (-3.89, 9.8)	..	0 (0)	0 (0, 31.32)	-0.61 (-0.61, -0.61)	..
Pancreas	0 (0)	0 (0, 16.96)	-1.75 (-1.75, -1.75)	1 (Reference)	0 (0)	0 (0, 4.97)	-1.93 (-1.93, -1.93)	..	2 (3.64)	3.05 (0.37, 11.01)	4.69 (-4.99, 14.37)	..	0 (0)	0 (0, 7.08)	-2.71 (-2.71, -2.71)	..
Retroperitoneum, peritoneum and other digestive system	0 (0)	0 (0, 142.01)	-0.21 (-0.21, -0.21)	1 (Reference)	0 (0)	0 (0, 41.83)	-0.23 (-0.23, -0.23)	..	1 (1.82)	12.9 (0.33, 71.89)	3.22 (-3.62, 10.07)	..	0 (0)	0 (0, 60.24)	-0.32 (-0.32, -0.32)	..
Lung and Bronchus	2 (6.9)	1.92 (0.23, 6.95)	7.74 (-14.6, 30.09)	1 (Reference)	7 (9.21)	2.05 (0.83, 4.23)	9.31 (-4.13, 22.75)	0.98 (0.2, 4.72)	2 (3.64)	0.7 (0.09, 2.54)	-2.97 (-12.65, 6.71)	0.28 (0.04, 2.01)	2 (6.06)	0.97 (0.12, 3.5)	-0.35 (-14.76, 14.06)	0.25 (0.03, 1.95)
Pleura, trachea and other respiratory	1 (3.45)	313.46 (7.94, 1746.51)	8.04 (-7.76, 23.83)	1 (Reference)	1 (1.32)	99.85 (2.53, 556.32)	2.57 (-2.51, 7.65)	0.78 (0.05, 12.82)	0 (0)	0 (0, 465.28)	-0.03 (-0.03, -0.03)	..	1 (3.03)	176.81 (4.48, 985.13)	5.17 (-5.02, 15.35)	1 (0.06, 15.99)
Bones and Joints	0 (0)	0 (0, 288.27)	-0.1 (-0.1, -0.1)	1 (Reference)	0 (0)	0 (0, 91.95)	-0.1 (-0.1, -0.1)	..	2 (3.64)	63.39 (7.68, 228.99)	6.88 (-2.81, 16.56)	..	0 (0)	0 (0, 165.71)	-0.12 (-0.12, -0.12)	..
Soft Tissue including Heart	6 (20.69)	111.33 (40.86, 242.33)	47.93 (9.23, 86.63)	1 (Reference)	21 (27.63)	117.61 (72.8, 179.78)	53.97 (30.69, 77.25)	1.13 (0.46, 2.8)	22 (40)	146.77 (91.98, 222.21)	76.31 (44.21, 108.42)	1.32 (0.54, 3.27)	3 (9.09)	26.76 (5.52, 78.21)	15.01 (-2.63, 32.65)	0.21 (0.05, 0.84)
Soft Tissue including Heart (excluded MPNST)	3 (10.34)	55.56 (11.46, 162.36)	23.75 (-3.62, 51.11)	1 (Reference)	4 (5.26)	22.35 (6.09, 57.22)	9.9 (-0.26, 20.06)	..	6 (10.91)	40.00 (14.68, 87.06)	20.43 (3.66, 37.2)	..	2 (6.06)	17.86 (2.16, 64.51)	9.81 (-4.59, 24.22)	..
Melanoma of the Skin	2 (6.9)	5.25 (0.64, 18.95)	13.05 (-9.29, 35.39)	1 (Reference)	9 (11.84)	7 (3.2, 13.29)	19.99 (4.75, 35.23)	1.39 (0.3, 6.44)	8 (14.55)	6.9 (2.98, 13.6)	23.89 (4.53, 43.25)	1.55 (0.33, 7.3)	1 (3.03)	1.13 (0.03, 6.28)	0.59 (-9.6, 10.77)	0.28 (0.03, 3.13)
Breast	0 (0)	0 (0, 3.48)	-8.54 (-8.54, -8.54)	1 (Reference)	5 (6.58)	1.39 (0.45, 3.25)	3.66 (-7.7, 15.02)	..	4 (7.27)	1.34 (0.36, 3.42)	3.51 (-10.19, 17.2)	..	5 (15.15)	2.21 (0.72, 5.15)	14.2 (-8.57, 36.98)	..
Cervix Uteri	0 (0)	0 (0, 60.41)	-1.05 (-1.05, -1.05)	1 (Reference)	0 (0)	0 (0, 18.5)	-1.07 (-1.07, -1.07)	..	0 (0)	0 (0, 24.42)	-1.09 (-1.09, -1.09)	..	0 (0)	0 (0, 36.09)	-1.13 (-1.13, -1.13)	..
Corpus and Uterus, NOS	0 (0)	0 (0, 17.5)	-3.62 (-3.62, -3.62)	1 (Reference)	1 (1.32)	1.35 (0.03, 7.53)	1.4 (-9.11, 11.91)	..	0 (0)	0 (0, 5.74)	-4.64 (-4.64, -4.64)	..	1 (3.03)	1.97 (0.05, 10.98)	5.42 (-16.15, 27)	..
Ovary	0 (0)	0 (0, 37.43)	-1.69 (-1.69, -1.69)	1 (Reference)	0 (0)	0 (0, 11.26)	-1.76 (-1.76, -1.76)	..	0 (0)	0 (0, 13.94)	-1.91 (-1.91, -1.91)	..	1 (3.03)	5.38 (0.14, 29.95)	8.96 (-12.62, 30.54)	..
Vagina, vulva and other female genital organs	0 (0)	0 (0, 104.39)	-0.61 (-0.61, -0.61)	1 (Reference)	0 (0)	0 (0, 30.36)	-0.65 (-0.65, -0.65)	..	0 (0)	0 (0, 33.76)	-0.79 (-0.79, -0.79)	..	0 (0)	0 (0, 39.87)	-1.02 (-1.02, -1.02)	..
Prostate	1 (3.45)	0.77 (0.02, 4.3)	-4.5 (-34.27, 25.27)	1 (Reference)	4 (5.26)	0.94 (0.26, 2.42)	-1.19 (-20.86, 18.47)	1.24 (0.14, 11.13)	3 (5.45)	0.85 (0.18, 2.48)	-3.63 (-26.61, 19.35)	1.15 (0.12, 11.36)	2 (6.06)	0.82 (0.1, 2.98)	-4.2 (-31.49, 23.09)	1.19 (0.1, 14.06)
Testis	0 (0)	0 (0, 88.22)	-0.64 (-0.64, -0.64)	1 (Reference)	0 (0)	0 (0, 30.53)	-0.61 (-0.61, -0.61)	..	0 (0)	0 (0, 43.7)	-0.57 (-0.57, -0.57)	..	0 (0)	0 (0, 59.56)	-0.61 (-0.61, -0.61)	..
Penis and other male genital organs	0 (0)	0 (0, 370.71)	-0.15 (-0.15, -0.15)	1 (Reference)	0 (0)	0 (0, 110.65)	-0.17 (-0.17, -0.17)	..	0 (0)	0 (0, 123.86)	-0.2 (-0.2, -0.2)	..	0 (0)	0 (0, 165.55)	-0.22 (-0.22, -0.22)	..
Urinary Bladder	1 (3.45)	2.61 (0.07, 14.57)	4.98 (-10.82, 20.78)	1 (Reference)	0 (0)	0 (0, 2.91)	-3.29 (-3.29, -3.29)	..	0 (0)	0 (0, 3.4)	-3.79 (-3.79, -3.79)	..	3 (9.09)	3.77 (0.78, 11.02)	11.46 (-6.19, 29.1)	1.26 (0.12, 13.53)
Kidney and Renal Pelvis	2 (6.9)	7.35 (0.89, 26.54)	13.93 (-8.42, 36.27)	1 (Reference)	1 (1.32)	1.08 (0.03, 6.02)	0.19 (-4.89, 5.27)	0.15 (0.01, 1.67)	1 (1.82)	1.22 (0.03, 6.77)	0.62 (-6.23, 7.46)	0.18 (0.02, 2.05)	3 (9.09)	4.77 (0.98, 13.93)	12.32 (-5.32, 29.96)	0.81 (0.13, 5.14)
Ureter and other urinary organs	0 (0)	0 (0, 232.2)	-0.13 (-0.13, -0.13)	1 (Reference)	0 (0)	0 (0, 68.16)	-0.14 (-0.14, -0.14)	..	0 (0)	0 (0, 76.98)	-0.17 (-0.17, -0.17)	..	0 (0)	0 (0, 95.54)	-0.2 (-0.2, -0.2)	..
Eye and Orbit	0 (0)	0 (0, 280.29)	-0.11 (-0.11, -0.11)	1 (Reference)	0 (0)	0 (0, 85.68)	-0.11 (-0.11, -0.11)	..	0 (0)	0 (0, 98.6)	-0.13 (-0.13, -0.13)	..	0 (0)	0 (0, 134.43)	-0.14 (-0.14, -0.14)	..
Brain and Other Nervous System	2 (6.9)	20.27 (2.45, 73.21)	15.33 (-7.02, 37.67)	1 (Reference)	6 (7.89)	18.76 (6.89, 40.84)	14.72 (2.28, 27.17)	0.87 (0.18, 4.31)	0 (0)	0 (0, 13.94)	-0.92 (-0.92, -0.92)	..	0 (0)	0 (0, 19.48)	-0.98 (-0.98, -0.98)	..
Thyroid	6 (20.69)	32.62 (11.97, 71.01)	46.88 (8.18, 85.58)	1 (Reference)	2 (2.63)	3.15 (0.38, 11.38)	3.54 (-3.65, 10.72)	0.1 (0.02, 0.47)	0 (0)	0 (0, 6.72)	-1.92 (-1.92, -1.92)	..	1 (3.03)	2.57 (0.07, 14.32)	3.18 (-7.01, 13.36)	0.07 (0.01, 0.55)
Other endocrine system	0 (0)	0 (0, 345.38)	-0.09 (-0.09, -0.09)	1 (Reference)	0 (0)	0 (0, 103.8)	-0.09 (-0.09, -0.09)	..	0 (0)	0 (0, 128.53)	-0.1 (-0.1, -0.1)	..	0 (0)	0 (0, 171.44)	-0.11 (-0.11, -0.11)	..
Hodgkin Lymphoma	0 (0)	0 (0, 96.04)	-0.31 (-0.31, -0.31)	1 (Reference)	0 (0)	0 (0, 30.98)	-0.31 (-0.31, -0.31)	..	0 (0)	0 (0, 41.78)	-0.31 (-0.31, -0.31)	..	0 (0)	0 (0, 63.88)	-0.3 (-0.3, -0.3)	..
Non-Hodgkin Lymphoma	2 (6.9)	4.73 (0.57, 17.07)	12.71 (-9.63, 35.05)	1 (Reference)	1 (1.32)	0.71 (0.02, 3.96)	-1.05 (-6.13, 4.03)	0.16 (0.01, 1.77)	1 (1.82)	0.83 (0.02, 4.6)	-0.74 (-7.58, 6.11)	0.21 (0.02, 2.37)	2 (6.06)	2.22 (0.27, 8.03)	5.72 (-8.69, 20.12)	0.65 (0.09, 4.83)
Myeloma	0 (0)	0 (0, 31.42)	-0.95 (-0.95, -0.95)	1 (Reference)	1 (1.32)	2.49 (0.06, 13.89)	1.55 (-3.53, 6.63)	..	1 (1.82)	2.82 (0.07, 15.71)	2.25 (-4.59, 9.1)	..	0 (0)	0 (0, 13.09)	-1.47 (-1.47, -1.47)	..
Acute Lymphocytic Leukemia	0 (0)	0 (0, 249.04)	-0.12 (-0.12, -0.12)	1 (Reference)	0 (0)	0 (0, 82.04)	-0.12 (-0.12, -0.12)	..	0 (0)	0 (0, 111.8)	-0.12 (-0.12, -0.12)	..	0 (0)	0 (0, 159.09)	-0.12 (-0.12, -0.12)	..
Chronic Myeloid Leukemia	0 (0)	0 (0, 122.9)	-0.24 (-0.24, -0.24)	1 (Reference)	2 (2.63)	19.98 (2.42, 72.19)	4.92 (-2.26, 12.11)	..	0 (0)	0 (0, 42.87)	-0.3 (-0.3, -0.3)	..	0 (0)	0 (0, 56.23)	-0.34 (-0.34, -0.34)	..
Acute Non-Lymphocytic Leukemia	0 (0)	0 (0, 50.75)	-0.59 (-0.59, -0.59)	1 (Reference)	2 (2.63)	8.32 (1.01, 30.06)	4.56 (-2.62, 11.75)	..	0 (0)	0 (0, 17.57)	-0.73 (-0.73, -0.73)	..	0 (0)	0 (0, 22.94)	-0.84 (-0.84, -0.84)	..
Others	2 (6.9)	5.89 (0.71, 21.27)	13.38 (-8.96, 35.73)	1 (Reference)	3 (3.95)	2.62 (0.54, 7.66)	4.81 (-3.99, 13.61)	0.45 (0.08, 2.69)	3 (5.45)	3.1 (0.64, 9.05)	7.1 (-4.76, 18.95)	0.54 (0.09, 3.25)	2 (6.06)	2.83 (0.34, 10.22)	6.72 (-7.68, 21.13)	0.49 (0.07, 3.59)

eTable41. The risk of developing SPC among survivors diagnosed with primary malignant peripheral nerve sheath tumor by radiotherapy during 2000-2019 in 17 SEER registers.

SPC	NHW				NHB				NHAPI				Hispanic				[illegible]r heterogen[illegible]
	Obs.	SIR (95% CI)	Absolute excess incidence per 10,000 person-year	Relative risk (95%CI)	Obs.	SIR (95% CI)	Absolute excess incidence per 10,000 person-year	Relative risk (95%CI)	Obs.	SIR (95% CI)	Absolute excess incidence per 10,000 person-year	Relative risk (95%CI)	Obs.	SIR (95% CI)	Absolute excess incidence per 10,000 person-year	Relative risk (95%CI)	
Total	128 (100)	2.5 (2.09, 2.97)	132.34 (94.14, 170.53)	1 (Reference)	21 (100)	2.35 (1.45, 3.59)	91.52 (23.29, 159.74)	0.92 (0.58, 1.47)	17 (100)	3.74 (2.18, 5.99)	146.29 (51.39, 241.2)	1.53 (0.92, 2.55)	26 (100)	2.91 (1.9, 4.27)	100.11 (41.52, 158.7)	0.96 (0.63, 1.48)	0.608
Total (exclude MPNST)	106 (82.81)	2.07 (1.70, 2.51)	94.44 (59.68, 129.2)	1 (Reference)	18 (85.71)	2.01 (1.19, 3.18)	68.73 (5.56, 131.89)	..	12 (70.59)	2.6 (1.36, 4.61)	87.58 (7.84, 167.31)	..	17 (65.38)	1.90 (1.11, 3.05)	47.35 (-0.03, 94.72)	..	..
Head and Neck	5 (3.91)	2.66 (0.86, 6.2)	5.37 (-2.18, 12.92)	1 (Reference)	0 (0)	0 (0, 13.57)	-2.07 (-2.07, -2.07)	..	0 (0)	0 (0, 25.96)	-1.67 (-1.67, -1.67)	..	0 (0)	0 (0, 13.47)	-1.61 (-1.61, -1.61)	..	0.066
Esophagus	0 (0)	0 (0, 6.42)	-0.99 (-0.99, -0.99)	1 (Reference)	0 (0)	0 (0, 43.84)	-0.64 (-0.64, -0.64)	..	0 (0)	0 (0, 103.76)	-0.42 (-0.42, -0.42)	..	0 (0)	0 (0, 53.75)	-0.4 (-0.4, -0.4)	..	>.99
Stomach	1 (0.78)	1.34 (0.03, 7.45)	0.43 (-2.94, 3.81)	1 (Reference)	2 (9.52)	9.94 (1.2, 35.89)	13.66 (-7.39, 34.72)	7.33 (0.64, 84.06)	0 (0)	0 (0, 23.39)	-1.85 (-1.85, -1.85)	..	1 (3.85)	8.41 (0.21, 46.86)	5.17 (-6.33, 16.66)	6.67 (0.37, 121.15)	0.378
Small Intestine	3 (2.34)	12.37 (2.55, 36.16)	4.75 (-1.1, 10.6)	1 (Reference)	1 (4.76)	14.15 (0.36, 78.83)	7.06 (-7.83, 21.95)	0.83 (0.08, 8.3)	0 (0)	0 (0, 205.69)	-0.21 (-0.21, -0.21)	..	1 (3.85)	23.5 (0.6, 130.91)	5.61 (-5.88, 17.1)	0.88 (0.09, 8.87)	0.9
Colon and Rectum	4 (3.13)	0.9 (0.24, 2.29)	-0.81 (-7.56, 5.95)	1 (Reference)	1 (4.76)	1.07 (0.03, 5.96)	0.5 (-14.39, 15.39)	1.15 (0.13, 10.34)	1 (5.88)	1.93 (0.05, 10.76)	5.66 (-17.36, 28.68)	2.14 (0.24, 19.31)	1 (3.85)	1.26 (0.03, 7)	1.19 (-10.3, 12.68)	1.18 (0.12, 11.24)	0.714
Anus, Anal Canal and Anorectum	0 (0)	0 (0, 18.06)	-0.35 (-0.35, -0.35)	1 (Reference)	0 (0)	0 (0, 101.66)	-0.28 (-0.28, -0.28)	..	0 (0)	0 (0, 454.04)	-0.1 (-0.1, -0.1)	..	0 (0)	0 (0, 82.53)	-0.26 (-0.26, -0.26)	..	>.99
Liver and intrahepatic bile duct	0 (0)	0 (0, 3.82)	-1.67 (-1.67, -1.67)	1 (Reference)	0 (0)	0 (0, 18.82)	-1.49 (-1.49, -1.49)	..	0 (0)	0 (0, 18.04)	-2.4 (-2.4, -2.4)	..	1 (3.85)	7.05 (0.18, 39.29)	5.03 (-6.46, 16.52)	..	0.059
Gallbladder and other biliary	0 (0)	0 (0, 11.59)	-0.55 (-0.55, -0.55)	1 (Reference)	0 (0)	0 (0, 59.75)	-0.47 (-0.47, -0.47)	..	1 (5.88)	18.01 (0.46, 100.36)	11.09 (-11.93, 34.11)	..	0 (0)	0 (0, 63.13)	-0.34 (-0.34, -0.34)	..	0.24
Pancreas	0 (0)	0 (0, 2.6)	-2.45 (-2.45, -2.45)	1 (Reference)	0 (0)	0 (0, 12.5)	-2.24 (-2.24, -2.24)	..	1 (5.88)	6.6 (0.17, 36.76)	9.96 (-13.05, 32.98)	..	1 (3.85)	4.14 (0.11, 23.06)	4.45 (-7.04, 15.94)	..	0.023
Retroperitoneum, peritoneum and other digestive system	0 (0)	0 (0, 22.37)	-0.28 (-0.28, -0.28)	1 (Reference)	0 (0)	0 (0, 126.96)	-0.22 (-0.22, -0.22)	..	0 (0)	0 (0, 182.63)	-0.24 (-0.24, -0.24)	..	1 (3.85)	28.68 (0.73, 159.79)	5.66 (-5.83, 17.15)	..	0.084
Lung and Bronchus	9 (7.03)	1.39 (0.63, 2.63)	4.32 (-5.81, 14.44)	1 (Reference)	3 (14.29)	2.58 (0.53, 7.53)	13.95 (-11.84, 39.73)	1.64 (0.44, 6.12)	1 (5.88)	1.82 (0.05, 10.14)	5.29 (-17.73, 28.31)	1.3 (0.16, 10.32)	0 (0)	0 (0, 3.61)	-5.99 (-5.99, -5.99)	..	0.42
Pleura, trachea and other respiratory	1 (0.78)	55.68 (1.41, 310.22)	1.69 (-1.68, 5.07)	1 (Reference)	0 (0)	0 (0, 1323.23)	-0.02 (-0.02, -0.02)	..	0 (0)	0 (0, 1910)	-0.02 (-0.02, -0.02)	..	2 (7.69)	553.73 (67.06, 2000.26)	11.7 (-4.55, 27.95)	6.02 (0.26, 139.59)	0.032
Bones and Joints	2 (1.56)	28.42 (3.44, 102.67)	3.32 (-1.45, 8.1)	1 (Reference)	0 (0)	0 (0, 335.31)	-0.08 (-0.08, -0.08)	..	0 (0)	0 (0, 603.01)	-0.07 (-0.07, -0.07)	..	0 (0)	0 (0, 209.39)	-0.1 (-0.1, -0.1)	..	0.348
Soft Tissue including Heart	33 (25.78)	99.58 (68.55, 139.85)	56.27 (36.88, 75.67)	1 (Reference)	3 (14.29)	51.19 (10.56, 149.59)	22.34 (-3.44, 48.13)	0.47 (0.14, 1.55)	5 (29.41)	153.01 (49.68, 357.07)	58.34 (6.87, 109.81)	1.66 (0.65, 4.28)	11 (42.31)	173.58 (86.65, 310.59)	64.12 (26.01, 102.23)	1.13 (0.55, 2.31)	0.583
Soft Tissue including Heart (excluded MPNST)	12 (9.38)	36.25 (18.73, 63.33)	20.1 (8.4, 31.8)	1 (Reference)	0 (0)	0 (0,62.52)	-0.45 (-0.45, -0.45)	..	0 (0)	0 (0, 111.78)	-0.39 (-0.39, -0.39)	..	3 (11.54)	47.62 (9.82, 139.16)	17.22 (-2.68, 37.12)	..	<.001
Melanoma of the Skin	18 (14.06)	5.8 (3.44, 9.17)	25.66 (11.34, 39.99)	1 (Reference)	1 (4.76)	53.17 (1.35, 296.24)	7.45 (-7.43, 22.34)	9.26 (1.21, 71.07)	1 (5.88)	40.87 (1.04, 227.73)	11.46 (-11.56, 34.48)	7.34 (0.97, 55.82)	0 (0)	0 (0, 7.09)	-3.05 (-3.05, -3.05)	..	0.156
Breast	11 (8.59)	1.9 (0.95, 3.4)	8.99 (-2.21, 20.19)	1 (Reference)	2 (9.52)	1.45 (0.18, 5.23)	4.69 (-16.37, 25.74)	0.74 (0.16, 3.37)	1 (5.88)	1.29 (0.03, 7.21)	2.67 (-20.35, 25.69)	0.64 (0.08, 4.99)	0 (0)	0 (0, 2.06)	-10.52 (-10.52, -10.52)	..	0.035
Cervix Uteri	0 (0)	0 (0, 13.36)	-1.07 (-1.07, -1.07)	1 (Reference)	0 (0)	0 (0, 42.36)	-1.26 (-1.26, -1.26)	..	0 (0)	0 (0, 87.43)	-1.01 (-1.01, -1.01)	..	0 (0)	0 (0, 38)	-1.02 (-1.02, -1.02)	..	>.99
Corpus and Uterus, NOS	0 (0)	0 (0, 3.01)	-4.75 (-4.75, -4.75)	1 (Reference)	1 (4.76)	3.45 (0.09, 19.22)	10.28 (-18.09, 38.65)	..	0 (0)	0 (0, 21.09)	-4.18 (-4.18, -4.18)	..	1 (3.85)	2.72 (0.07, 15.17)	6.61 (-13.88, 27.11)	..	0.208
Ovary	0 (0)	0 (0, 7)	-2.04 (-2.04, -2.04)	1 (Reference)	0 (0)	0 (0, 37.56)	-1.42 (-1.42, -1.42)	..	1 (5.88)	14.47 (0.37, 80.63)	22.23 (-24.58, 69.04)	..	0 (0)	0 (0, 22.07)	-1.75 (-1.75, -1.75)	..	0.405
Vagina, vulva and other female genital organs	0 (0)	0 (0, 16.64)	-0.86 (-0.86, -0.86)	1 (Reference)	0 (0)	0 (0, 84.36)	-0.63 (-0.63, -0.63)	..	0 (0)	0 (0, 182.84)	-0.48 (-0.48, -0.48)	..	0 (0)	0 (0, 53.57)	-0.72 (-0.72, -0.72)	..	>.99
Prostate	9 (7.03)	1.06 (0.49, 2.02)	1.64 (-16.6, 19.89)	1 (Reference)	1 (4.76)	0.59 (0.02, 3.29)	-11.07 (-42.39, 20.26)	0.56 (0.07, 4.44)	0 (0)	0 (0, 7.42)	-11.48 (-11.48, -11.48)	..	0 (0)	0 (0, 5.4)	-9.13 (-9.13, -9.13)	..	0.139
Testis	0 (0)	0 (0, 17.38)	-0.66 (-0.66, -0.66)	1 (Reference)	0 (0)	0 (0, 315.17)	-0.19 (-0.19, -0.19)	..	0 (0)	0 (0, 332.03)	-0.26 (-0.26, -0.26)	..	0 (0)	0 (0, 54.07)	-0.91 (-0.91, -0.91)	..	>.99
Penis and other male genital organs	0 (0)	0 (0, 51.43)	-0.22 (-0.22, -0.22)	1 (Reference)	0 (0)	0 (0, 373.95)	-0.16 (-0.16, -0.16)	..	0 (0)	0 (0, 684.62)	-0.13 (-0.13, -0.13)	..	0 (0)	0 (0, 545.79)	-0.09 (-0.09, -0.09)	..	>.99
Urinary Bladder	3 (2.34)	1.07 (0.22, 3.13)	0.35 (-5.5, 6.19)	1 (Reference)	0 (0)	0 (0, 15.97)	-1.76 (-1.76, -1.76)	..	0 (0)	0 (0, 26.16)	-1.66 (-1.66, -1.66)	..	1 (3.85)	3.29 (0.08, 18.32)	4.08 (-7.41, 15.57)	2.8 (0.26, 30.31)	0.553
Kidney and Renal Pelvis	4 (3.13)	2.17 (0.59, 5.55)	3.71 (-3.04, 10.47)	1 (Reference)	2 (9.52)	5.98 (0.72, 21.6)	12.65 (-8.4, 33.7)	2.99 (0.54, 16.52)	0 (0)	0 (0, 27.32)	-1.59 (-1.59, -1.59)	..	1 (3.85)	3.36 (0.09, 18.74)	4.12 (-7.37, 15.61)	1.78 (0.19, 16.63)	0.653
Ureter and other urinary organs	0 (0)	0 (0, 32.16)	-0.2 (-0.2, -0.2)	1 (Reference)	0 (0)	0 (0, 271.92)	-0.1 (-0.1, -0.1)	..	0 (0)	0 (0, 345.82)	-0.13 (-0.13, -0.13)	..	0 (0)	0 (0, 245.78)	-0.09 (-0.09, -0.09)	..	>.99
Eye and Orbit	0 (0)	0 (0, 38.22)	-0.17 (-0.17, -0.17)	1 (Reference)	0 (0)	0 (0, 1294.55)	-0.02 (-0.02, -0.02)	..	0 (0)	0 (0, 1307.96)	-0.03 (-0.03, -0.03)	..	0 (0)	0 (0, 214.71)	-0.1 (-0.1, -0.1)	..	>.99
Brain and Other Nervous System	7 (5.47)	11.1 (4.46, 22.88)	10.97 (2.04, 19.9)	1 (Reference)	0 (0)	0 (0, 58.76)	-0.48 (-0.48, -0.48)	..	0 (0)	0 (0, 88.78)	-0.49 (-0.49, -0.49)	..	1 (3.85)	8.05 (0.2, 44.86)	5.13 (-6.36, 16.62)	0.74 (0.09, 6.21)	0.42
Thyroid	6 (4.69)	5.4 (1.98, 11.75)	8.42 (0.15, 16.69)	1 (Reference)	0 (0)	0 (0, 25.66)	-1.09 (-1.09, -1.09)	..	1 (5.88)	6.78 (0.17, 37.76)	10.01 (-13.01, 33.03)	1.25 (0.15, 10.35)	1 (3.85)	3.18 (0.08, 17.69)	4.02 (-7.47, 15.51)	0.51 (0.06, 4.35)	0.582
Other endocrine system	0 (0)	0 (0, 65.56)	-0.1 (-0.1, -0.1)	1 (Reference)	0 (0)	0 (0, 235.78)	-0.12 (-0.12, -0.12)	..	0 (0)	0 (0, 355.84)	-0.12 (-0.12, -0.12)	..	0 (0)	0 (0, 305.02)	-0.07 (-0.07, -0.07)	..	>.99
Hodgkin Lymphoma	0 (0)	0 (0, 18.92)	-0.34 (-0.34, -0.34)	1 (Reference)	0 (0)	0 (0, 93.19)	-0.3 (-0.3, -0.3)	..	0 (0)	0 (0, 312.34)	-0.14 (-0.14, -0.14)	..	0 (0)	0 (0, 69.75)	-0.31 (-0.31, -0.31)	..	>.99
Non-Hodgkin Lymphoma	3 (2.34)	1.05 (0.22, 3.07)	0.24 (-5.61, 6.09)	1 (Reference)	1 (4.76)	3.05 (0.08, 16.97)	5.1 (-9.79, 19.99)	3.01 (0.31, 29.7)	1 (5.88)	4.7 (0.12, 26.16)	9.24 (-13.78, 32.26)	4.97 (0.52, 48.02)	1 (3.85)	2.09 (0.05, 11.63)	3.06 (-8.44, 14.55)	1.81 (0.18, 18.7)	0.376
Myeloma	1 (0.78)	1.41 (0.04, 7.84)	0.5 (-2.88, 3.88)	1 (Reference)	0 (0)	0 (0, 14.38)	-1.95 (-1.95, -1.95)	..	1 (5.88)	16.7 (0.42, 93.07)	11.04 (-11.98, 34.06)	15.84 (0.98, 255.65)	0 (0)	0 (0, 32.72)	-0.66 (-0.66, -0.66)	..	0.447
Acute Lymphocytic Leukemia	0 (0)	0 (0, 48.97)	-0.13 (-0.13, -0.13)	1 (Reference)	0 (0)	0 (0, 384.18)	-0.07 (-0.07, -0.07)	..	0 (0)	0 (0, 450.59)	-0.1 (-0.1, -0.1)	..	0 (0)	0 (0, 176.47)	-0.12 (-0.12, -0.12)	..	>.99
Chronic Myeloid Leukemia	2 (1.56)	10.29 (1.25, 37.19)	3.11 (-1.66, 7.88)	1 (Reference)	0 (0)	0 (0, 116.25)	-0.24 (-0.24, -0.24)	..	0 (0)	0 (0, 228.56)	-0.19 (-0.19, -0.19)	..	0 (0)	0 (0, 105.95)	-0.2 (-0.2, -0.2)	..	0.192
Acute Non-Lymphocytic Leukemia	2 (1.56)	4.22 (0.51, 15.24)	2.63 (-2.15, 7.4)	1 (Reference)	0 (0)	0 (0, 54.33)	-0.52 (-0.52, -0.52)	..	0 (0)	0 (0, 76.51)	-0.57 (-0.57, -0.57)	..	0 (0)	0 (0, 44.9)	-0.48 (-0.48, -0.48)	..	0.135
Others	4 (3.13)	1.8 (0.49, 4.62)	3.07 (-3.68, 9.82)	1 (Reference)	3 (14.29)	8.27 (1.71, 24.18)	20.03 (-5.75, 45.82)	4.64 (1.01, 21.25)	2 (11.76)	11.21 (1.36, 40.48)	21.39 (-11.16, 53.94)	6.47 (1.18, 35.6)	1 (3.85)	2.82 (0.07, 15.68)	3.78 (-7.71, 15.27)	1.62 (0.17, 15.19)	0.214

eTable42. The risk of developing SPC among survivors diagnosed with primary malignant peripheral nerve sheath tumor by radiotherapy during 2000-2019 in 17 SEER registers.

SPC	No				Yes				r heterogen
	Obs.	SIR (95% CI)	Absolute excess incidence per 10,000 person-year	Relative risk (95%CI)	Obs.	SIR (95% CI)	Absolute excess incidence per 10,000 person-year	Relative risk (95%CI)	
Total	109 (100)	2.41 (1.98, 2.91)	110.03 (74.72, 145.34)	1 (Reference)	84 (100)	2.85 (2.27, 3.52)	133.14 (89.23, 177.05)	1.15 (0.87, 1.53)	0.328
Total (excluded MPNST)	92 (84.4)	2.03 (1.64, 2.49)	80.69 (48.25, 113.14)	1 (Reference)	62 (73.81)	2.13 (1.64, 2.73)	79.37 (41.65, 117.09)	..	..
Head and Neck	1 (0.92)	0.63 (0.02, 3.5)	-1.02 (-4.41, 2.36)	1 (Reference)	4 (4.76)	3.93 (1.07, 10.06)	7.29 (-2.29, 16.87)	5.83 (0.65, 52.35)	0.073
Esophagus	0 (0)	0 (0, 7.75)	-0.82 (-0.82, -0.82)	1 (Reference)	0 (0)	0 (0, 12.34)	-0.73 (-0.73, -0.73)	..	>.99
Stomach	2 (1.83)	2.69 (0.33, 9.72)	2.17 (-2.61, 6.95)	1 (Reference)	2 (2.38)	3.95 (0.48, 14.28)	3.65 (-3.12, 10.43)	1.37 (0.19, 9.89)	0.753
Small Intestine	4 (3.67)	17.81 (4.85, 45.6)	6.52 (-0.25, 13.28)	1 (Reference)	1 (1.19)	6.49 (0.16, 36.17)	2.07 (-2.72, 6.86)	0.35 (0.04, 3.18)	0.31
Colon and Rectum	6 (5.5)	1.49 (0.55, 3.25)	3.42 (-4.86, 11.71)	1 (Reference)	1 (1.19)	0.36 (0.01, 1.98)	-4.43 (-9.22, 0.36)	0.22 (0.03, 1.87)	0.105
Anus, Anal Canal and Anorectum	0 (0)	0 (0, 21.04)	-0.3 (-0.3, -0.3)	1 (Reference)	0 (0)	0 (0, 30.31)	-0.3 (-0.3, -0.3)	..	>.99
Liver and intrahepatic bile duct	1 (0.92)	1.04 (0.03, 5.82)	0.07 (-3.31, 3.45)	1 (Reference)	0 (0)	0 (0, 6.34)	-1.42 (-1.42, -1.42)	..	0.325
Gallbladder and other biliary	0 (0)	0 (0, 12.36)	-0.52 (-0.52, -0.52)	1 (Reference)	1 (1.19)	4.91 (0.12, 27.36)	1.95 (-2.84, 6.74)	..	0.239
Pancreas	0 (0)	0 (0, 2.91)	-2.19 (-2.19, -2.19)	1 (Reference)	2 (2.38)	2.3 (0.28, 8.31)	2.76 (-4.01, 9.54)	..	0.06
Retroperitoneum, peritoneum and other digestive system	1 (0.92)	6.65 (0.17, 37.03)	1.47 (-1.92, 4.85)	1 (Reference)	0 (0)	0 (0, 36.02)	-0.25 (-0.25, -0.25)	..	0.254
Lung and Bronchus	10 (9.17)	1.77 (0.85, 3.25)	7.48 (-3.21, 18.18)	1 (Reference)	3 (3.57)	0.81 (0.17, 2.37)	-1.72 (-10.01, 6.58)	0.43 (0.12, 1.57)	0.175
Pleura, trachea and other respiratory	2 (1.83)	126.44 (15.31, 456.73)	3.42 (-1.36, 8.21)	1 (Reference)	1 (1.19)	91.15 (2.31, 507.85)	2.42 (-2.37, 7.21)	0.5 (0.05, 5.51)	0.56
Bones and Joints	1 (0.92)	15.85 (0.4, 88.32)	1.62 (-1.77, 5)	1 (Reference)	1 (1.19)	22.91 (0.58, 127.66)	2.34 (-2.45, 7.13)	2 (0.13, 31.98)	0.627
Soft Tissue including Heart	24 (22.02)	81.94 (52.5, 121.92)	40.91 (24.34, 57.48)	1 (Reference)	28 (33.33)	138.93 (92.32, 200.79)	67.95 (42.6, 93.3)	1.61 (0.93, 2.78)	0.087
Soft Tissue including Heart (excluded MPNST)	7 (6.42)	23.89 (9.61, 49.22)	11.57 (2.63, 20.52)	1 (Reference)	7 (8.33)	34.65 (13.93, 71.40)	16.62 (3.94, 29.29)	..	..
Melanoma of the Skin	8 (7.34)	3.51 (1.52, 6.92)	9.87 (0.31, 19.44)	1 (Reference)	12 (14.29)	8.36 (4.32, 14.6)	25.82 (9.23, 42.42)	2.21 (0.9, 5.41)	0.08
Breast	10 (9.17)	1.71 (0.82, 3.15)	7.18 (-3.52, 17.87)	1 (Reference)	4 (4.76)	0.98 (0.27, 2.52)	-0.17 (-9.75, 9.41)	0.6 (0.19, 1.93)	0.377
Cervix Uteri	0 (0)	0 (0, 11.95)	-0.53 (-0.53, -0.53)	1 (Reference)	0 (0)	0 (0, 18)	-0.5 (-0.5, -0.5)	..	>.99
Corpus and Uterus, NOS	1 (0.92)	0.8 (0.02, 4.44)	-0.44 (-3.82, 2.94)	1 (Reference)	1 (1.19)	1.19 (0.03, 6.6)	0.38 (-4.41, 5.17)	1.79 (0.11, 28.63)	0.682
Ovary	0 (0)	0 (0, 7.21)	-0.88 (-0.88, -0.88)	1 (Reference)	1 (1.19)	2.74 (0.07, 15.28)	1.55 (-3.24, 6.34)	..	0.153
Vagina, vulva and other female genital organs	0 (0)	0 (0, 17.87)	-0.36 (-0.36, -0.36)	1 (Reference)	0 (0)	0 (0, 24.23)	-0.37 (-0.37, -0.37)	..	>.99
Prostate	7 (6.42)	0.95 (0.38, 1.95)	-0.66 (-9.61, 8.29)	1 (Reference)	3 (3.57)	0.73 (0.15, 2.13)	-2.72 (-11.02, 5.58)	0.79 (0.2, 3.07)	0.725
Testis	0 (0)	0 (0, 22.69)	-0.28 (-0.28, -0.28)	1 (Reference)	0 (0)	0 (0, 25.19)	-0.36 (-0.36, -0.36)	..	>.99
Penis and other male genital organs	0 (0)	0 (0, 63.49)	-0.1 (-0.1, -0.1)	1 (Reference)	0 (0)	0 (0, 99.04)	-0.09 (-0.09, -0.09)	..	>.99
Urinary Bladder	2 (1.83)	0.93 (0.11, 3.35)	-0.27 (-5.05, 4.51)	1 (Reference)	2 (2.38)	1.45 (0.18, 5.25)	1.52 (-5.25, 8.3)	1.69 (0.23, 12.21)	0.606
Kidney and Renal Pelvis	5 (4.59)	3.11 (1.01, 7.26)	5.86 (-1.71, 13.42)	1 (Reference)	2 (2.38)	1.92 (0.23, 6.92)	2.34 (-4.44, 9.11)	0.63 (0.12, 3.27)	0.569
Ureter and other urinary organs	0 (0)	0 (0, 38.92)	-0.16 (-0.16, -0.16)	1 (Reference)	0 (0)	0 (0, 59.73)	-0.15 (-0.15, -0.15)	..	>.99
Eye and Orbit	0 (0)	0 (0, 50.14)	-0.13 (-0.13, -0.13)	1 (Reference)	0 (0)	0 (0, 77.66)	-0.12 (-0.12, -0.12)	..	>.99
Brain and Other Nervous System	5 (4.59)	9.55 (3.1, 22.29)	7.73 (0.16, 15.29)	1 (Reference)	3 (3.57)	8.6 (1.77, 25.12)	6.48 (-1.82, 14.78)	0.79 (0.19, 3.34)	0.75
Thyroid	6 (5.5)	5.6 (2.06, 12.19)	8.51 (0.22, 16.79)	1 (Reference)	3 (3.57)	4.37 (0.9, 12.78)	5.66 (-2.64, 13.95)	0.62 (0.15, 2.51)	0.498
Other endocrine system	0 (0)	0 (0, 63.57)	-0.1 (-0.1, -0.1)	1 (Reference)	0 (0)	0 (0, 96.05)	-0.09 (-0.09, -0.09)	..	>.99
Hodgkin Lymphoma	0 (0)	0 (0, 20.97)	-0.3 (-0.3, -0.3)	1 (Reference)	0 (0)	0 (0, 28.9)	-0.31 (-0.31, -0.31)	..	>.99
Non-Hodgkin Lymphoma	3 (2.75)	1.27 (0.26, 3.71)	1.1 (-4.76, 6.96)	1 (Reference)	3 (3.57)	1.9 (0.39, 5.56)	3.48 (-4.82, 11.78)	1.39 (0.28, 7)	0.688
Myeloma	1 (0.92)	1.47 (0.04, 8.17)	0.55 (-2.83, 3.93)	1 (Reference)	1 (1.19)	2.11 (0.05, 11.78)	1.29 (-3.5, 6.08)	1.58 (0.1, 25.48)	0.747
Acute Lymphocytic Leukemia	0 (0)	0 (0, 53.08)	-0.12 (-0.12, -0.12)	1 (Reference)	0 (0)	0 (0, 79.4)	-0.11 (-0.11, -0.11)	..	>.99
Chronic Myeloid Leukemia	2 (1.83)	12 (1.45, 43.34)	3.16 (-1.62, 7.95)	1 (Reference)	0 (0)	0 (0, 32.07)	-0.28 (-0.28, -0.28)	..	0.124
Acute Non-Lymphocytic Leukemia	1 (0.92)	2.45 (0.06, 13.63)	1.02 (-2.36, 4.4)	1 (Reference)	1 (1.19)	3.64 (0.09, 20.26)	1.77 (-3.02, 6.56)	1.2 (0.07, 19.5)	0.896
Others	6 (5.5)	3.26 (1.2, 7.09)	7.17 (-1.11, 15.46)	1 (Reference)	4 (4.76)	3.04 (0.83, 7.78)	6.56 (-3.02, 16.14)	0.9 (0.25, 3.21)	0.874

eTable43. The risk of developing SPC among survivors diagnosed with primary malignant peripheral nerve sheath tumor by chemotherapy during 2000-2019 in 17 SEER registers.

SPC	No				Yes				r heterogen
	Obs.	SIR (95% CI)	Absolute excess incidence per 10,000 person-year	Relative risk (95%CI)	Obs.	SIR (95% CI)	Absolute excess incidence per 10,000 person-year	Relative risk (95%CI)	
Total	163 (100)	2.36 (2.01, 2.75)	111.32 (81.67, 140.96)	1 (Reference)	30 (100)	5.24 (3.53, 7.48)	167.93 (93.66, 242.21)	1.85 (1.24, 2.76)	0.005
Total (excluded MPNST)	132 (80.98)	1.91 (1.60, 2.27)	74.59 (47.91, 101.27)	1 (Reference)	23 (76.67)	4.02 (2.55, 6.03)	119.5 (54.47, 184.54)	..	..
Head and Neck	4 (2.45)	1.68 (0.46, 4.31)	1.92 (-2.72, 6.56)	1 (Reference)	1 (3.33)	4.3 (0.11, 23.96)	5.31 (-8.25, 18.87)	2.08 (0.23, 19.17)	0.548
Esophagus	0 (0)	0 (0, 5.15)	-0.85 (-0.85, -0.85)	1 (Reference)	0 (0)	0 (0, 63.99)	-0.4 (-0.4, -0.4)	..	>.99
Stomach	4 (2.45)	3.46 (0.94, 8.85)	3.37 (-1.28, 8.01)	1 (Reference)	0 (0)	0 (0, 40.16)	-0.64 (-0.64, -0.64)	..	0.576
Small Intestine	3 (1.84)	8.61 (1.78, 25.17)	3.14 (-0.88, 7.16)	1 (Reference)	2 (6.67)	65.82 (7.97, 237.78)	13.63 (-5.55, 32.81)	21.41 (2.36, 194.24)	0.013
Colon and Rectum	5 (3.07)	0.79 (0.26, 1.85)	-1.54 (-6.73, 3.65)	1 (Reference)	2 (6.67)	3.77 (0.46, 13.6)	10.16 (-9.02, 29.34)	6.35 (1.16, 34.85)	0.065
Anus, Anal Canal and Anorectum	0 (0)	0 (0, 13.6)	-0.32 (-0.32, -0.32)	1 (Reference)	0 (0)	0 (0, 143.11)	-0.18 (-0.18, -0.18)	..	>.99
Liver and intrahepatic bile duct	1 (0.61)	0.71 (0.02, 3.95)	-0.49 (-2.81, 1.83)	1 (Reference)	0 (0)	0 (0, 28.9)	-0.88 (-0.88, -0.88)	..	0.546
Gallbladder and other biliary	1 (0.61)	2.13 (0.05, 11.87)	0.63 (-1.69, 2.95)	1 (Reference)	0 (0)	0 (0, 112.09)	-0.23 (-0.23, -0.23)	..	0.92
Pancreas	2 (1.23)	1 (0.12, 3.62)	0.01 (-3.28, 3.29)	1 (Reference)	0 (0)	0 (0, 25.67)	-0.99 (-0.99, -0.99)	..	0.698
Retroperitoneum, peritoneum and other digestive system	1 (0.61)	4.26 (0.11, 23.74)	0.91 (-1.42, 3.23)	1 (Reference)	0 (0)	0 (0, 202.45)	-0.13 (-0.13, -0.13)	..	0.766
Lung and Bronchus	13 (7.98)	1.48 (0.79, 2.53)	5.01 (-3.37, 13.38)	1 (Reference)	0 (0)	0 (0, 6.24)	-4.09 (-4.09, -4.09)	..	0.146
Pleura, trachea and other respiratory	3 (1.84)	126.05 (26, 368.38)	3.53 (-0.5, 7.55)	1 (Reference)	0 (0)	0 (0, 1233.75)	-0.02 (-0.02, -0.02)	..	0.094
Bones and Joints	2 (1.23)	21.53 (2.61, 77.77)	2.26 (-1.02, 5.54)	1 (Reference)	0 (0)	0 (0, 266.79)	-0.1 (-0.1, -0.1)	..	0.447
Soft Tissue including Heart	41 (25.15)	91.62 (65.75, 124.29)	48.04 (33.17, 62.91)	1 (Reference)	11 (36.67)	234.42 (117.02, 419.45)	75.78 (30.81, 120.76)	1.74 (0.86, 3.51)	0.141
Soft Tissue including Heart (excluded MPNST)	11 (6.75)	24.55 (12.26, 43.93)	12.5 (4.8, 20.2)	1 (Reference)	4 (13.33)	85.11 (23.19, 217.91)	27.35 (0.23, 54.47)	..	..
Melanoma of the Skin	19 (11.66)	5.56 (3.35, 8.68)	18.46 (8.34, 28.58)	1 (Reference)	1 (3.33)	3.39 (0.09, 18.88)	4.88 (-8.68, 18.44)	0.92 (0.12, 7.03)	0.931
Breast	11 (6.75)	1.2 (0.6, 2.15)	2.2 (-5.5, 9.9)	1 (Reference)	3 (10)	3.91 (0.81, 11.41)	15.44 (-8.05, 38.93)	3.35 (0.89, 12.52)	0.108
Cervix Uteri	0 (0)	0 (0, 7.92)	-0.55 (-0.55, -0.55)	1 (Reference)	0 (0)	0 (0, 76.62)	-0.33 (-0.33, -0.33)	..	>.99
Corpus and Uterus, NOS	2 (1.23)	1.03 (0.13, 3.73)	0.07 (-3.21, 3.36)	1 (Reference)	0 (0)	0 (0, 22.78)	-1.12 (-1.12, -1.12)	..	0.531
Ovary	1 (0.61)	1.24 (0.03, 6.89)	0.23 (-2.1, 2.55)	1 (Reference)	0 (0)	0 (0, 54.42)	-0.47 (-0.47, -0.47)	..	0.569
Vagina, vulva and other female genital organs	0 (0)	0 (0, 11.04)	-0.4 (-0.4, -0.4)	1 (Reference)	0 (0)	0 (0, 150.53)	-0.17 (-0.17, -0.17)	..	>.99
Prostate	10 (6.13)	0.94 (0.45, 1.73)	-0.73 (-8.07, 6.62)	1 (Reference)	0 (0)	0 (0, 4.17)	-6.12 (-6.12, -6.12)	..	0.215
Testis	0 (0)	0 (0, 15.31)	-0.29 (-0.29, -0.29)	1 (Reference)	0 (0)	0 (0, 54.27)	-0.47 (-0.47, -0.47)	..	>.99
Penis and other male genital organs	0 (0)	0 (0, 42.01)	-0.1 (-0.1, -0.1)	1 (Reference)	0 (0)	0 (0, 489.21)	-0.05 (-0.05, -0.05)	..	>.99
Urinary Bladder	4 (2.45)	1.2 (0.33, 3.08)	0.8 (-3.85, 5.44)	1 (Reference)	0 (0)	0 (0, 18.08)	-1.41 (-1.41, -1.41)	..	0.402
Kidney and Renal Pelvis	7 (4.29)	2.89 (1.16, 5.95)	5.42 (-0.72, 11.56)	1 (Reference)	0 (0)	0 (0, 16.34)	-1.56 (-1.56, -1.56)	..	0.264
Ureter and other urinary organs	0 (0)	0 (0, 24.98)	-0.18 (-0.18, -0.18)	1 (Reference)	0 (0)	0 (0, 416.55)	-0.06 (-0.06, -0.06)	..	>.99
Eye and Orbit	0 (0)	0 (0, 33.21)	-0.13 (-0.13, -0.13)	1 (Reference)	0 (0)	0 (0, 368.62)	-0.07 (-0.07, -0.07)	..	>.99
Brain and Other Nervous System	4 (2.45)	5.09 (1.39, 13.02)	3.81 (-0.84, 8.45)	1 (Reference)	4 (13.33)	46.48 (12.66, 119)	27.08 (-0.04, 54.2)	7.09 (1.7, 29.64)	0.011
Thyroid	7 (4.29)	4.45 (1.79, 9.17)	6.43 (0.29, 12.57)	1 (Reference)	2 (6.67)	10.84 (1.31, 39.14)	12.56 (-6.62, 31.74)	1.59 (0.32, 7.85)	0.586
Other endocrine system	0 (0)	0 (0, 42.75)	-0.1 (-0.1, -0.1)	1 (Reference)	0 (0)	0 (0, 363.29)	-0.07 (-0.07, -0.07)	..	>.99
Hodgkin Lymphoma	0 (0)	0 (0, 14.25)	-0.31 (-0.31, -0.31)	1 (Reference)	0 (0)	0 (0, 82.52)	-0.31 (-0.31, -0.31)	..	>.99
Non-Hodgkin Lymphoma	5 (3.07)	1.37 (0.45, 3.21)	1.61 (-3.58, 6.8)	1 (Reference)	1 (3.33)	3.35 (0.09, 18.64)	4.85 (-8.71, 18.41)	3.34 (0.37, 30.48)	0.346
Myeloma	2 (1.23)	1.87 (0.23, 6.74)	1.1 (-2.18, 4.38)	1 (Reference)	0 (0)	0 (0, 44.51)	-0.57 (-0.57, -0.57)	..	0.567
Acute Lymphocytic Leukemia	0 (0)	0 (0, 37.23)	-0.12 (-0.12, -0.12)	1 (Reference)	0 (0)	0 (0, 218.42)	-0.12 (-0.12, -0.12)	..	>.99
Chronic Myeloid Leukemia	1 (0.61)	3.88 (0.1, 21.64)	0.88 (-1.44, 3.2)	1 (Reference)	1 (3.33)	41.3 (1.05, 230.11)	6.75 (-6.81, 20.31)	7.66 (0.46, 128.16)	0.186
Acute Non-Lymphocytic Leukemia	0 (0)	0 (0, 5.84)	-0.75 (-0.75, -0.75)	1 (Reference)	2 (6.67)	38.65 (4.68, 139.62)	13.48 (-5.7, 32.66)	..	0.017
Others	10 (6.13)	3.39 (1.62, 6.23)	8.35 (1.01, 15.69)	1 (Reference)	0 (0)	0 (0, 17.82)	-1.43 (-1.43, -1.43)	..	0.234

eTable50. Causes of death among survivors diagnosed with primary malignant peripheral nerve sheath tumor by sex during 2000-2019 in 17 SEER registers.

COD	Male				Female			
	Obs.	SMR (95% CI)	Absolute excess incidence per 10,000 person-year	Relative risk (95%CI)	Obs.	SMR (95% CI)	Absolute excess incidence per 10,000 person-year	Relative risk (95%CI)
All Cause of Death	606 (100)	7.33 (6.76 to 7.94)	758.15 (762.52, 917.4)	1 (Reference)	457 (100)	6.5 (5.92 to 7.13)	669.86 (597.28, 742.43)	1.24 (1.1, 1.4)
All malignant cancers	457 (75.41)	23.34 (21.25 to 25.58)	629.55 (634.81, 769.31)	1 (Reference)	333 (72.87)	22.61 (20.24 to 25.17)	551.3 (489.34, 613.25)	0.85 (0.73, 0.97)
Head and Neck	6 (0.99)	9.8 (3.6 to 21.32)	6.82 (0.94, 16.35)	1 (Reference)	3 (0.66)	15.17 (3.13 to 44.33)	4.85 (-1.03, 10.73)	1.99 (0.49, 8.12)
Esophagus	0 (0)	0 (0 to 4.99)	-0.75 (-1.19, -1.19)	1 (Reference)	0 (0)	0 (0 to 22.98)	-0.28 (-0.28, -0.28)	..
Stomach	3 (0.5)	6.72 (1.39 to 19.64)	3.57 (-1.35, 9.55)	1 (Reference)	2 (0.44)	7.43 (0.9 to 26.86)	3 (-1.8, 7.8)	1.01 (0.17, 6.07)
Small Intestine	0 (0)	0 (0 to 77.31)	-0.07 (-0.08, -0.08)	1 (Reference)	0 (0)	0 (0 to 108.76)	-0.06 (-0.06, -0.06)	..
Colon and Rectum	4 (0.66)	2.27 (0.62 to 5.82)	1.54 (-2.69, 9.89)	1 (Reference)	1 (0.22)	0.72 (0.02 to 4.01)	-0.67 (-4.07, 2.72)	0.29 (0.03, 2.58)
Anus, Anal Canal and Anorectum	0 (0)	0 (0 to 167.13)	-0.04 (-0.04, -0.04)	1 (Reference)	0 (0)	0 (0 to 128.88)	-0.05 (-0.05, -0.05)	..
Liver and intrahepatic bile duct	4 (0.66)	4.05 (1.1 to 10.38)	3 (-1.46, 11.13)	1 (Reference)	1 (0.22)	2.42 (0.06 to 13.5)	1.02 (-2.38, 4.41)	0.58 (0.06, 5.23)
Gallbladder and other biliary	0 (0)	0 (0 to 37.73)	-0.19 (-0.16, -0.16)	1 (Reference)	0 (0)	0 (0 to 29.52)	-0.22 (-0.22, -0.22)	..
Pancreas	1 (0.17)	0.78 (0.02 to 4.34)	0.57 (-3.6, 2.69)	1 (Reference)	2 (0.44)	1.93 (0.23 to 6.98)	1.67 (-3.13, 6.47)	2.14 (0.19, 23.58)
Retroperitoneum, peritoneum and other digestive system	4 (0.66)	215.06 (58.6 to 550.64)	4.11 (0.1, 12.68)	1 (Reference)	1 (0.22)	22.17 (0.56 to 123.51)	1.65 (-1.74, 5.05)	0.15 (0.02, 1.43)
Lung and Bronchus	35 (5.78)	6.37 (4.44 to 8.86)	38.22 (28.75, 65.97)	1 (Reference)	20 (4.38)	5.5 (3.36 to 8.5)	28.35 (13.17, 43.53)	0.81 (0.47, 1.41)
Pleura, trachea and other respiratory	1 (0.17)	45.17 (1.14 to 251.68)	1.64 (-1.58, 4.72)	1 (Reference)	1 (0.22)	117.25 (2.97 to 653.29)	1.72 (-1.68, 5.11)	1.73 (0.1, 30.76)
Bones and Joints	10 (1.65)	218.35 (104.71 to 401.56)	15.77 (6.03, 25.92)	1 (Reference)	9 (1.97)	302.35 (138.25 to 573.96)	15.54 (5.35, 25.72)	1.82 (0.73, 4.57)
Soft Tissue including Heart	288 (47.52)	2073.58 (1840.99 to 2327.42)	426.34 (408.64, 515.41)	1 (Reference)	224 (49.02)	2066.13 (1804.4 to 2355.16)	387.82 (337.01, 438.63)	1.03 (0.86, 1.22)
Melanoma of the Skin	9 (1.49)	25.64 (11.72 to 48.67)	12.08 (4.44, 23.32)	1 (Reference)	6 (1.31)	40.08 (14.71 to 87.24)	10.13 (1.82, 18.45)	1.59 (0.56, 4.47)
Breast	0 (0)	0 (0 to 130.14)	-1.85 (-0.04, -0.04)	1 (Reference)	0 (0)	0 (0 to 1.68)	-3.8 (-3.8, -3.8)	..
Cervix Uteri	0 (0)	0 (0 to 0)	0.66 (0, 0)	1 (Reference)	1 (0.22)	4.72 (0.12 to 26.32)	1.37 (-2.03, 4.76)	..
Corpus and Uterus, NOS	0 (0)	0 (0 to 0)	0.42 (0, 0)	1 (Reference)	1 (0.22)	2.01 (0.05 to 11.2)	0.87 (-2.52, 4.27)	..
Ovary	0 (0)	0 (0 to 0)	1.04 (0, 0)	1 (Reference)	2 (0.44)	2.66 (0.32 to 9.6)	2.16 (-2.64, 6.96)	..
Vagina, vulva and other female genital organs	0 (0)	0 (0 to 0)	-0.09 (0, 0)	1 (Reference)	0 (0)	0 (0 to 33.46)	-0.19 (-0.19, -0.19)	..
Prostate	1 (0.17)	0.51 (0.01 to 2.82)	-0.82 (-4.72, 1.57)	1 (Reference)	0 (0)	0 (0 to 0)	0 (0, 0)	..
Testis	0 (0)	0 (0 to 197.98)	-0.02 (-0.03, -0.03)	1 (Reference)	0 (0)	0 (0 to 0)	0 (0, 0)	..
Penis and other male genital organs	0 (0)	0 (0 to 169.04)	-0.02 (-0.04, -0.04)	1 (Reference)	0 (0)	0 (0 to 0)	0 (0, 0)	..
Urinary Bladder	2 (0.33)	2.8 (0.34 to 10.11)	1.7 (-2.38, 6.51)	1 (Reference)	1 (0.22)	4.13 (0.11 to 23)	1.31 (-2.08, 4.71)	1.51 (0.14, 16.72)
Kidney and Renal Pelvis	0 (0)	0 (0 to 6.62)	0.99 (-0.89, -0.89)	1 (Reference)	2 (0.44)	7.85 (0.95 to 28.35)	3.02 (-1.78, 7.82)	..
Ureter and other urinary organs	0 (0)	0 (0 to 117.77)	-0.04 (-0.05, -0.05)	1 (Reference)	0 (0)	0 (0 to 201.05)	-0.03 (-0.03, -0.03)	..
Eye and Orbit	0 (0)	0 (0 to 378.77)	0.82 (-0.02, -0.02)	1 (Reference)	1 (0.22)	141.62 (3.59 to 789.04)	1.72 (-1.68, 5.12)	..
Brain and Other Nervous System	21 (3.47)	41.5 (25.69 to 63.44)	23.47 (18.48, 47.31)	1 (Reference)	8 (1.75)	24.56 (10.6 to 48.4)	13.29 (3.69, 22.9)	0.62 (0.27, 1.4)
Thyroid	0 (0)	0 (0 to 71.89)	-0.09 (-0.08, -0.08)	1 (Reference)	0 (0)	0 (0 to 67.92)	-0.09 (-0.09, -0.09)	..
Other endocrine system	3 (0.5)	114.89 (23.69 to 335.76)	3.29 (-0.68, 10.22)	1 (Reference)	1 (0.22)	46.86 (1.19 to 261.1)	1.7 (-1.7, 5.09)	0.35 (0.04, 3.39)
Hodgkin Lymphoma	0 (0)	0 (0 to 96.31)	-0.05 (-0.06, -0.06)	1 (Reference)	0 (0)	0 (0 to 152.84)	-0.04 (-0.04, -0.04)	..
Non-Hodgkin Lymphoma	6 (0.99)	6.65 (2.44 to 14.47)	5.42 (0.48, 15.89)	1 (Reference)	2 (0.44)	3.4 (0.41 to 12.29)	2.45 (-2.36, 7.25)	0.55 (0.11, 2.74)
Myeloma	1 (0.17)	2.42 (0.06 to 13.51)	0.25 (-2.2, 4.09)	1 (Reference)	0 (0)	0 (0 to 12.66)	-0.51 (-0.5, -0.5)	..
Acute Lymphocytic Leukemia	0 (0)	0 (0 to 91.25)	-0.06 (-0.06, -0.06)	1 (Reference)	0 (0)	0 (0 to 132.91)	-0.05 (-0.05, -0.05)	..
Acute Non-Lymphocytic Leukemia	0 (0)	0 (0 to 8.97)	-0.56 (-0.66, -0.66)	1 (Reference)	0 (0)	0 (0 to 14.38)	-0.44 (-0.44, -0.44)	..
Chronic Myeloid Leukemia	0 (0)	0 (0 to 94.96)	-0.05 (-0.06, -0.06)	1 (Reference)	0 (0)	0 (0 to 142.56)	-0.05 (-0.05, -0.05)	..
Others	58 (9.57)	32.51 (24.69 to 42.03)	82.46 (66.27, 114.19)	1 (Reference)	44 (9.63)	35.78 (26 to 48.03)	74.09 (51.56, 96.61)	1.07 (0.72, 1.59)
In situ, benign or unknown behavior neoplasm	27 (4.46)	52.72 (34.74 to 76.7)	49.24 (26.17, 58.86)	1 (Reference)	33 (7.22)	84.91 (58.45 to 119.25)	56.49 (36.98, 75.99)	2.32 (1.32, 4.07)
Septicemia	1 (0.17)	0.88 (0.02 to 4.93)	1.48 (-3.36, 2.94)	1 (Reference)	3 (0.66)	2.74 (0.57 to 8)	3.3 (-2.58, 9.18)	2.96 (0.31, 28.46)
Other Infectious and Parasitic Diseases including HIV	3 (0.5)	3.04 (0.63 to 8.87)	2 (-2.22, 8.68)	1 (Reference)	1 (0.22)	1.62 (0.04 to 9.03)	0.66 (-2.73, 4.06)	0.85 (0.09, 8.23)
Diabetes Mellitus	1 (0.17)	0.38 (0.01 to 2.12)	-0.56 (-5.76, 0.53)	1 (Reference)	3 (0.66)	1.47 (0.3 to 4.29)	1.65 (-4.23, 7.53)	3.85 (0.4, 37.33)
Alzheimer's (ICD-9 and 10 only)	3 (0.5)	1.5 (0.31 to 4.37)	1.96 (-3.85, 7.04)	1 (Reference)	5 (1.09)	1.37 (0.45 to 3.2)	2.35 (-5.24, 9.94)	0.8 (0.19, 3.37)
CVD	41 (6.77)	1.52 (1.09 to 2.06)	16.1 (2.36, 42.65)	1 (Reference)	29 (6.35)	1.22 (0.82 to 1.76)	9.19 (-9.1, 27.47)	0.81 (0.5, 1.31)
Diseases of Heart	24 (3.96)	1.12 (0.72 to 1.67)	5.31 (-11.31, 19.51)	1 (Reference)	21 (4.6)	1.22 (0.76 to 1.87)	6.61 (-8.95, 22.17)	1.14 (0.63, 2.06)
Hypertension without Heart Disease	2 (0.33)	2.36 (0.29 to 8.52)	1.81 (-2.6, 6.3)	1 (Reference)	2 (0.44)	2.03 (0.25 to 7.33)	1.76 (-3.04, 6.56)	0.69 (0.1, 4.92)
Cerebrovascular Diseases	14 (2.31)	3.68 (2.01 to 6.17)	7.89 (4.59, 28.13)	1 (Reference)	4 (0.88)	0.85 (0.23 to 2.17)	-1.26 (-8.05, 5.53)	0.23 (0.08, 0.7)
Atherosclerosis	0 (0)	0 (0 to 19.32)	0.46 (-0.31, -0.31)	1 (Reference)	1 (0.22)	3.91 (0.1 to 21.8)	1.29 (-2.11, 4.68)	..
Aortic Aneurysm and Dissection	1 (0.17)	2.45 (0.06 to 13.64)	1.12 (-2.2, 4.1)	1 (Reference)	1 (0.22)	3.98 (0.1 to 22.16)	1.3 (-2.1, 4.69)	1.48 (0.09, 23.59)
Other Diseases of Arteries, Arterioles, Capillaries	0 (0)	0 (0 to 13.05)	-0.48 (-0.45, -0.45)	1 (Reference)	0 (0)	0 (0 to 12.75)	-0.5 (-0.5, -0.5)	..
Pneumonia and Influenza	7 (1.16)	4.04 (1.62 to 8.32)	6.32 (0.13, 16.78)	1 (Reference)	4 (0.88)	2.38 (0.65 to 6.09)	4.01 (-2.78, 10.8)	0.61 (0.18, 2.11)
Chronic Obstructive Pulmonary Disease and Allied Cond	5 (0.83)	1.11 (0.36 to 2.59)	-1.3 (-6.25, 7.81)	1 (Reference)	2 (0.44)	0.5 (0.06 to 1.79)	-3.54 (-8.34, 1.26)	0.46 (0.09, 2.38)
Chronic Liver Disease and Cirrhosis	2 (0.33)	1.46 (0.18 to 5.27)	-0.02 (-3.44, 5.46)	1 (Reference)	0 (0)	0 (0 to 5.63)	-1.14 (-1.13, -1.13)	..
Nephritis, Nephrotic Syndrome and Nephrosis	2 (0.33)	1.25 (0.15 to 4.52)	0.87 (-3.8, 5.1)	1 (Reference)	2 (0.44)	1.47 (0.18 to 5.32)	1.11 (-3.69, 5.91)	1.21 (0.17, 8.6)
Symptoms, Signs and Ill-Defined Conditions	2 (0.33)	2.33 (0.28 to 8.43)	3.47 (-2.61, 6.28)	1 (Reference)	4 (0.88)	4.07 (1.11 to 10.42)	5.23 (-1.56, 12.02)	2.9 (0.51, 16.39)
Accidents and Adverse Effects	4 (0.66)	0.89 (0.24 to 2.29)	-0.69 (-7.06, 5.52)	1 (Reference)	2 (0.44)	0.85 (0.1 to 3.08)	-0.6 (-5.4, 4.21)	1.1 (0.2, 6.12)
Suicide and Self-Inflicted Injury	3 (0.5)	1.91 (0.39 to 5.57)	0.87 (-3.16, 7.74)	1 (Reference)	0 (0)	0 (0 to 9.64)	-0.66 (-0.66, -0.66)	..
Other COD	47 (7.76)	3.74 (2.75 to 4.98)	48.22 (33.72, 76.85)	1 (Reference)	36 (7.88)	2.87 (2.01 to 3.97)	40.59 (20.22, 60.96)	1.07 (0.69, 1.67)
Syphilis	0 (0)	0 (0 to 2298.79)	0 (0, 0)	1 (Reference)	0 (0)	0 (0 to 3824.89)	0 (0, 0)	..
Tuberculosis	0 (0)	0 (0 to 149.74)	-0.03 (-0.04, -0.04)	1 (Reference)	0 (0)	0 (0 to 267.66)	-0.02 (-0.02, -0.02)	..
Stomach and Duodenal Ulcers	0 (0)	0 (0 to 35.26)	-0.16 (-0.17, -0.17)	1 (Reference)	0 (0)	0 (0 to 40.45)	-0.16 (-0.16, -0.16)	..
Puerperium	0 (0)	0 (0 to 0)	-0.02 (0, 0)	1 (Reference)	0 (0)	0 (0 to 145.93)	-0.04 (-0.04, -0.04)	..
Congenital Anomalies	28 (4.62)	226.11 (150.25 to 326.78)	36.47 (28.1, 61.39)	1 (Reference)	16 (3.5)	161.17 (92.12 to 261.74)	27.54 (13.96, 41.12)	0.75 (0.41, 1.4)
Certain Conditions Originating in. Perinatal Period	0 (0)	0 (0 to 234.62)	-0.02 (-0.03, -0.03)	1 (Reference)	0 (0)	0 (0 to 314.45)	-0.02 (-0.02, -0.02)	..
Homicide and Legal Intervention	0 (0)	0 (0 to 5.96)	-0.63 (-0.99, -0.99)	1 (Reference)	0 (0)	0 (0 to 27.11)	-0.24 (-0.24, -0.24)	..
Other Cause of Death	19 (3.14)	1.63 (0.98 to 2.54)	12.62 (-1.94, 25.48)	1 (Reference)	20 (4.38)	1.64 (1 to 2.53)	13.53 (-1.65, 28.71)	1.13 (0.6, 2.13)

eTable51. Causes of death among survivors diagnosed with primary malignant peripheral nerve sheath tumor by age during 2000-2019 in 17 SEER registers.

COD	0-19 years				20-39 years				40-59 years				⩾ 60 years			
	Obs.	SMR (95% CI)	Absolute excess incidence per 10,000 person-year	Relative risk (95%CI	Obs.	SMR (95% CI)	Absolute excess incidence per 10,000 person-year	Relative risk (95%CI	Obs.	SMR (95% CI)	Absolute excess incidence per 10,000 person-year	Relative risk (95%CI	Obs.	SMR (95% CI)	Absolute excess incidence per 10,000 person-year	Relative risk (95%CI)
All Cause of Death	91 (100)	90.16 (72.59 to 110.69)	621.72 (492.55, 750.89)	1 (Reference)	311 (100)	49.52 (44.17 to 55.34)	852.61 (755.89, 949.32)	0.52 (0.41, 0.65)	315 (100)	9.58 (8.55 to 10.7)	618.87 (542.56, 695.18)	0.11 (0.09, 0.14)	346 (100)	3.07 (2.75 to 3.41)	962.28 (811.86, 1112.71)	0.03 (0.02, 0.03)
All malignant cancers	74 (81.32	1332.78 (1046.52 to 1673.18)	510.86 (394.37, 627.34)	1 (Reference)	251 (80.71	261.68 (230.31 to 296.14)	699.62 (612.73, 786.5)	0.25 (0.19, 0.32)	263 (83.49	26.06 (23 to 29.41)	554.81 (485.08, 624.54)	0.02 (0.02, 0.03)	202 (58.38	8.71 (7.55 to 9.99)	737.69 (622.76, 852.63)	0 (0, 0.01)
Head and Neck	0 (0)	0 (0 to 4606.98)	-0.01 (-0.01, -0.01)	1 (Reference)	2 (0.64)	83.28 (10.09 to 300.85)	5.53 (-2.23, 13.28)	..	4 (1.27)	12.49 (3.4 to 31.99)	8.07 (-0.53, 16.67)	..	3 (0.87)	6.45 (1.33 to 18.85)	10.46 (-3.55, 24.47)	..
Esophagus	0 (0)	0 (0 to 12032.41)	0 (0, 0)	1 (Reference)	0 (0)	0 (0 to 183.6)	-0.06 (-0.06, -0.06)	..	0 (0)	0 (0 to 11.03)	-0.73 (-0.73, -0.73)	..	0 (0)	0 (0 to 6.76)	-2.25 (-2.25, -2.25)	..
Stomach	0 (0)	0 (0 to 3625.02)	-0.01 (-0.01, -0.01)	1 (Reference)	1 (0.32)	33.07 (0.84 to 184.27)	2.71 (-2.77, 8.2)	..	0 (0)	0 (0 to 17.57)	-0.46 (-0.46, -0.46)	..	4 (1.16)	8.44 (2.3 to 21.6)	14.55 (-1.63, 30.72)	..
Small Intestine	0 (0)	0 (0 to 51890.88)	0 (0, 0)	1 (Reference)	0 (0)	0 (0 to 1350.9)	-0.01 (-0.01, -0.01)	..	0 (0)	0 (0 to 147.63)	-0.06 (-0.05, -0.05)	..	0 (0)	0 (0 to 68.51)	-0.22 (-0.22, -0.22)	..
Colon and Rectum	0 (0)	0 (0 to 1467.18)	-0.02 (-0.02, -0.02)	1 (Reference)	1 (0.32)	9.41 (0.24 to 52.42)	2.5 (-2.98, 7.99)	..	0 (0)	0 (0 to 4.14)	-1.96 (-1.96, -1.96)	..	4 (1.16)	1.86 (0.51 to 4.77)	7.65 (-8.53, 23.82)	..
Anus, Anal Canal and Anorectum	0 (0)	0 (0 to 79149.08)	0 (0, 0)	1 (Reference)	0 (0)	0 (0 to 1163.82)	-0.01 (-0.01, -0.01)	..	0 (0)	0 (0 to 166.43)	-0.05 (-0.05, -0.05)	..	0 (0)	0 (0 to 145.73)	-0.1 (-0.1, -0.1)	..
Liver and intrahepatic bile duct	0 (0)	0 (0 to 2744.98)	-0.01 (-0.01, -0.01)	1 (Reference)	0 (0)	0 (0 to 113.95)	-0.09 (-0.09, -0.09)	..	1 (0.32)	1.69 (0.04 to 9.42)	0.9 (-3.4, 5.19)	..	4 (1.16)	5.17 (1.41 to 13.23)	13.31 (-2.86, 29.48)	..
Gallbladder and other biliary	0 (0)	0 (0 to 50539.26)	0 (0, 0)	1 (Reference)	0 (0)	0 (0 to 813.11)	-0.01 (-0.01, -0.01)	..	0 (0)	0 (0 to 61.75)	-0.13 (-0.13, -0.13)	..	0 (0)	0 (0 to 23.29)	-0.65 (-0.65, -0.65)	..
Pancreas	0 (0)	0 (0 to 8559.38)	0 (0, 0)	1 (Reference)	0 (0)	0 (0 to 84.51)	-0.12 (-0.12, -0.12)	..	0 (0)	0 (0 to 5.19)	-1.56 (-1.56, -1.56)	..	3 (0.87)	1.92 (0.4 to 5.61)	5.93 (-8.08, 19.94)	..
Retroperitoneum, peritoneum and other digestive system	0 (0)	0 (0 to 40487.01)	0 (0, 0)	1 (Reference)	2 (0.64)	1055.04 (127.77 to 3811.16)	5.59 (-2.17, 13.35)	..	1 (0.32)	52.09 (1.32 to 290.25)	2.15 (-2.15, 6.45)	..	2 (0.58)	47.03 (5.7 to 169.89)	8.08 (-3.36, 19.51)	..
Lung and Bronchus	1 (1.1)	922.04 (23.34 to 5137.3)	6.9 (-6.64, 20.44)	1 (Reference)	13 (4.18)	101.72 (54.16 to 173.94)	36.02 (16.24, 55.79)	0.03 (0, 0.19)	19 (6.03)	7.05 (4.24 to 11.01)	35.77 (17.03, 54.51)	0 (0, 0.01)	22 (6.36)	3.49 (2.19 to 5.29)	64.77 (26.84, 102.7)	0 (0, 0)
Pleura, trachea and other respiratory	0 (0)	0 (0 to 25444.12)	0 (0, 0)	1 (Reference)	1 (0.32)	1021.77 (25.87 to 5692.95)	2.8 (-2.69, 8.28)	..	0 (0)	0 (0 to 458.91)	-0.02 (-0.02, -0.02)	..	1 (0.29)	46.5 (1.18 to 259.09)	4.04 (-4.05, 12.12)	..
Bones and Joints	0 (0)	0 (0 to 709.52)	-0.04 (-0.03, -0.03)	1 (Reference)	6 (1.93)	664.66 (243.92 to 1446.68)	16.76 (3.33, 30.2)	..	9 (2.86)	421.97 (192.95 to 801.03)	19.7 (6.8, 32.6)	..	4 (1.16)	99.98 (27.24 to 255.98)	16.34 (0.17, 32.51)	..
Soft Tissue including Heart	64 (70.33	14685.67 (11309.76 to 18753.28)	442.13 (333.8, 550.46)	1 (Reference)	176 (56.59	7799.66 (6689.89 to 9040.94)	492.39 (419.63, 565.14)	0.41 (0.3, 0.55)	175 (55.56	1993.31 (1708.91 to 2311.51)	383.71 (326.83, 440.59)	0.12 (0.09, 0.16)	97 (28.03)	731.59 (593.27 to 892.47)	399.67 (320.02, 479.31)	0.03 (0.02, 0.04)
Melanoma of the Skin	0 (0)	0 (0 to 3123.58)	-0.01 (-0.01, -0.01)	1 (Reference)	1 (0.32)	35.58 (0.9 to 198.21)	2.72 (-2.76, 8.2)	..	7 (2.22)	42.77 (17.19 to 88.11)	15 (3.62, 26.37)	..	7 (2.02)	22.75 (9.14 to 46.86)	27.61 (6.22, 49.01)	..
Breast	0 (0)	0 (0 to 2517)	-0.01 (-0.01, -0.01)	1 (Reference)	0 (0)	0 (0 to 25.91)	-0.4 (-0.4, -0.4)	..	0 (0)	0 (0 to 4.59)	-1.77 (-1.77, -1.77)	..	0 (0)	0 (0 to 2.89)	-5.26 (-5.26, -5.26)	..
Cervix Uteri	0 (0)	0 (0 to 3561.33)	-0.02 (-0.02, -0.02)	1 (Reference)	0 (0)	0 (0 to 99.6)	-0.22 (-0.22, -0.22)	..	1 (0.32)	10.12 (0.26 to 56.38)	4.14 (-4.87, 13.16)	..	0 (0)	0 (0 to 49.34)	-0.61 (-0.61, -0.61)	..
Corpus and Uterus, NOS	0 (0)	0 (0 to 17656.92)	0 (0, 0)	1 (Reference)	0 (0)	0 (0 to 238.68)	-0.09 (-0.09, -0.09)	..	0 (0)	0 (0 to 21.04)	-0.81 (-0.8, -0.8)	..	1 (0.29)	3.26 (0.08 to 18.18)	5.65 (-10.32, 21.63)	..
Ovary	0 (0)	0 (0 to 4466.02)	-0.01 (-0.02, -0.02)	1 (Reference)	0 (0)	0 (0 to 123.9)	-0.17 (-0.18, -0.18)	..	1 (0.32)	3.99 (0.1 to 22.21)	3.45 (-5.57, 12.46)	..	1 (0.29)	2.12 (0.05 to 11.83)	4.31 (-11.66, 20.28)	..
Vagina, vulva and other female genital organs	0 (0)	0 (0 to 42637.22)	0 (0, 0)	1 (Reference)	0 (0)	0 (0 to 980.16)	-0.02 (-0.02, -0.02)	..	0 (0)	0 (0 to 124.76)	-0.14 (-0.14, -0.14)	..	0 (0)	0 (0 to 48.02)	-0.63 (-0.63, -0.63)	..
Prostate	0 (0)	0 (0 to 96178.9)	0 (0, 0)	1 (Reference)	0 (0)	0 (0 to 860.25)	-0.02 (-0.02, -0.02)	..	0 (0)	0 (0 to 14.43)	-1.07 (-1.07, -1.07)	..	1 (0.29)	0.58 (0.02 to 3.24)	-6.01 (-22.39, 10.37)	..
Testis	0 (0)	0 (0 to 2436.41)	-0.02 (-0.03, -0.03)	1 (Reference)	0 (0)	0 (0 to 511.03)	-0.04 (-0.04, -0.04)	..	0 (0)	0 (0 to 563.31)	-0.03 (-0.03, -0.03)	..	0 (0)	0 (0 to 1100.79)	-0.03 (-0.03, -0.03)	..
Penis and other male genital organs	0 (0)	0 (0 to 126350.66)	0 (0, 0)	1 (Reference)	0 (0)	0 (0 to 4352.33)	-0.01 (-0.01, -0.01)	..	0 (0)	0 (0 to 577.91)	-0.03 (-0.03, -0.03)	..	0 (0)	0 (0 to 253.31)	-0.12 (-0.13, -0.13)	..
Urinary Bladder	0 (0)	0 (0 to 37038.62)	0 (0, 0)	1 (Reference)	0 (0)	0 (0 to 477.58)	-0.02 (-0.02, -0.02)	..	1 (0.32)	5.93 (0.15 to 33.05)	1.82 (-2.48, 6.12)	..	2 (0.58)	2.56 (0.31 to 9.26)	5.03 (-6.4, 16.47)	..
Kidney and Renal Pelvis	0 (0)	0 (0 to 3535.03)	-0.01 (-0.01, -0.01)	1 (Reference)	1 (0.32)	50.23 (1.27 to 279.87)	2.74 (-2.74, 8.23)	..	1 (0.32)	3.88 (0.1 to 21.62)	1.63 (-2.67, 5.93)	..	0 (0)	0 (0 to 6.92)	-2.2 (-2.2, -2.2)	..
Ureter and other urinary organs	0 (0)	0 (0 to 707563.92)	0 (0, 0)	1 (Reference)	0 (0)	0 (0 to 7043.92)	0 (0, 0)	..	0 (0)	0 (0 to 403.17)	-0.02 (-0.02, -0.02)	..	0 (0)	0 (0 to 92.24)	-0.17 (-0.17, -0.17)	..
Eye and Orbit	0 (0)	0 (0 to 53836.56)	0 (0, 0)	1 (Reference)	0 (0)	0 (0 to 4431.17)	0 (0, 0)	..	1 (0.32)	172.64 (4.37 to 961.89)	2.18 (-2.12, 6.48)	..	0 (0)	0 (0 to 364.98)	-0.04 (-0.04, -0.04)	..
Brain and Other Nervous System	2 (2.2)	214.96 (26.03 to 776.51)	13.75 (-5.39, 32.91)	1 (Reference)	8 (2.57)	125.93 (54.37 to 248.14)	22.21 (6.69, 37.72)	0.87 (0.18, 4.1)	11 (3.49)	32.53 (16.24 to 58.2)	23.39 (9.13, 37.65)	0.22 (0.05, 1.01)	8 (2.31)	19.02 (8.21 to 37.47)	31.27 (8.4, 54.14)	0.09 (0.02, 0.43)
Thyroid	0 (0)	0 (0 to 26987.52)	0 (0, 0)	1 (Reference)	0 (0)	0 (0 to 1239.63)	-0.01 (-0.01, -0.01)	..	0 (0)	0 (0 to 123.05)	-0.07 (-0.07, -0.07)	..	0 (0)	0 (0 to 50.86)	-0.3 (-0.3, -0.3)	..
Other endocrine system	0 (0)	0 (0 to 2572.91)	-0.01 (-0.01, -0.01)	1 (Reference)	0 (0)	0 (0 to 777.21)	-0.01 (-0.01, -0.01)	..	3 (0.95)	160.95 (33.19 to 470.38)	6.54 (-0.91, 13.99)	..	1 (0.29)	44.19 (1.12 to 246.18)	4.03 (-4.06, 12.12)	..
Hodgkin Lymphoma	0 (0)	0 (0 to 3166.6)	-0.01 (-0.01, -0.01)	1 (Reference)	0 (0)	0 (0 to 471.93)	-0.02 (-0.02, -0.02)	..	0 (0)	0 (0 to 199.56)	-0.04 (-0.04, -0.04)	..	0 (0)	0 (0 to 105.48)	-0.14 (-0.14, -0.14)	..
Non-Hodgkin Lymphoma	0 (0)	0 (0 to 1172.78)	-0.02 (-0.02, -0.02)	1 (Reference)	3 (0.96)	91.36 (18.84 to 267)	8.3 (-1.2, 17.8)	..	1 (0.32)	3.2 (0.08 to 17.81)	1.51 (-2.79, 5.81)	..	4 (1.16)	3.51 (0.96 to 8.97)	11.8 (-4.38, 27.97)	..
Myeloma	0 (0)	0 (0 to 58416.84)	0 (0, 0)	1 (Reference)	0 (0)	0 (0 to 410.7)	-0.03 (-0.03, -0.03)	..	1 (0.32)	5.94 (0.15 to 33.07)	1.82 (-2.47, 6.12)	..	0 (0)	0 (0 to 7.01)	-2.17 (-2.17, -2.17)	..
Acute Lymphocytic Leukemia	0 (0)	0 (0 to 768.65)	-0.03 (-0.03, -0.03)	1 (Reference)	0 (0)	0 (0 to 326)	-0.03 (-0.03, -0.03)	..	0 (0)	0 (0 to 166.35)	-0.05 (-0.05, -0.05)	..	0 (0)	0 (0 to 123.41)	-0.12 (-0.12, -0.12)	..
Acute Non-Lymphocytic Leukemia	0 (0)	0 (0 to 771.65)	-0.03 (-0.03, -0.03)	1 (Reference)	0 (0)	0 (0 to 136.47)	-0.08 (-0.08, -0.08)	..	0 (0)	0 (0 to 22.04)	-0.37 (-0.37, -0.37)	..	0 (0)	0 (0 to 7.87)	-1.93 (-1.93, -1.93)	..
Chronic Myeloid Leukemia	0 (0)	0 (0 to 11453.69)	0 (0, 0)	1 (Reference)	0 (0)	0 (0 to 1077.27)	-0.01 (-0.01, -0.01)	..	0 (0)	0 (0 to 266.33)	-0.03 (-0.03, -0.03)	..	0 (0)	0 (0 to 78.28)	-0.19 (-0.19, -0.19)	..
Others	7 (7.69)	1197.93 (481.63 to 2468.18)	48.32 (12.49, 84.15)	1 (Reference)	36 (11.58)	505.79 (354.25 to 700.23)	100.53 (67.63, 133.43)	0.31 (0.13, 0.7)	26 (8.25)	33.38 (21.81 to 48.91)	55.33 (33.4, 77.25)	0.02 (0.01, 0.04)	33 (9.54)	15.29 (10.53 to 21.48)	127.25 (80.8, 173.71)	0.01 (0, 0.01)
In situ, benign or unknown behavior neoplasm	10 (10.99	3202.88 (1535.9 to 5890.2)	69.07 (26.25, 111.89)	1 (Reference)	16 (5.14)	771.58 (441.03 to 1253)	44.71 (22.77, 66.65)	0.07 (0.03, 0.18)	19 (6.03)	126.39 (76.1 to 197.38)	41.35 (22.61, 60.09)	0.01 (0.01, 0.04)	15 (4.34)	20.64 (11.55 to 34.05)	58.89 (27.57, 90.21)	0 (0, 0)
Septicemia	0 (0)	0 (0 to 664.03)	-0.04 (-0.04, -0.04)	1 (Reference)	0 (0)	0 (0 to 56.16)	-0.18 (-0.18, -0.18)	..	0 (0)	0 (0 to 7.5)	-1.08 (-1.08, -1.08)	..	4 (1.16)	2.4 (0.66 to 6.16)	9.64 (-6.54, 25.81)	..
Other Infectious and Parasitic Diseases including HIV	0 (0)	0 (0 to 374.59)	-0.07 (-0.07, -0.07)	1 (Reference)	1 (0.32)	6.23 (0.16 to 34.72)	2.35 (-3.13, 7.83)	..	3 (0.95)	4.33 (0.89 to 12.65)	5.06 (-2.39, 12.51)	..	0 (0)	0 (0 to 4.98)	-3.06 (-3.06, -3.06)	..
Diabetes Mellitus	0 (0)	0 (0 to 466.11)	-0.06 (-0.06, -0.06)	1 (Reference)	0 (0)	0 (0 to 23.73)	-0.44 (-0.43, -0.43)	..	1 (0.32)	0.8 (0.02 to 4.43)	-0.56 (-4.86, 3.74)	..	3 (0.87)	0.92 (0.19 to 2.69)	-1.05 (-15.06, 12.95)	..
Alzheimer's (ICD-9 and 10 only)	0 (0)	0 (0 to 461022.24)	0 (0, 0)	1 (Reference)	0 (0)	0 (0 to 2536.95)	0 (0, 0)	..	0 (0)	0 (0 to 27.65)	-0.29 (-0.29, -0.29)	..	8 (2.31)	1.45 (0.63 to 2.86)	10.25 (-12.62, 33.12)	..
CVD	2 (2.2)	39.87 (4.83 to 144.04)	13.47 (-5.68, 32.62)	1 (Reference)	5 (1.61)	4.87 (1.58 to 11.37)	11.12 (-1.14, 23.38)	0.12 (0.02, 0.62)	7 (2.22)	0.79 (0.32 to 1.63)	-4.09 (-15.46, 7.29)	0.02 (0, 0.09)	56 (16.18)	1.38 (1.04 to 1.79)	62.98 (2.46, 123.49)	0.03 (0.01, 0.14)
Diseases of Heart	1 (1.1)	25.12 (0.64 to 139.94)	6.63 (-6.91, 20.17)	1 (Reference)	3 (0.96)	3.66 (0.75 to 10.68)	6.1 (-3.4, 15.6)	0.14 (0.02, 1.39)	6 (1.9)	0.84 (0.31 to 1.84)	-2.45 (-12.98, 8.08)	0.03 (0, 0.27)	35 (10.12)	1.14 (0.8 to 1.59)	17.94 (-29.9, 65.79)	0.04 (0.01, 0.28)
Hypertension without Heart Disease	0 (0)	0 (0 to 3759.81)	-0.01 (-0.01, -0.01)	1 (Reference)	0 (0)	0 (0 to 110.73)	-0.09 (-0.09, -0.09)	..	0 (0)	0 (0 to 12.06)	-0.67 (-0.67, -0.67)	..	4 (1.16)	2.68 (0.73 to 6.86)	10.34 (-5.83, 26.52)	..
Cerebrovascular Diseases	1 (1.1)	138.03 (3.5 to 769.07)	6.86 (-6.68, 20.4)	1 (Reference)	2 (0.64)	14.42 (1.75 to 52.08)	5.21 (-2.55, 12.96)	0.1 (0.01, 1.16)	1 (0.32)	0.85 (0.02 to 4.73)	-0.39 (-4.69, 3.91)	0.01 (0, 0.1)	14 (4.05)	1.94 (1.06 to 3.26)	28.01 (-2.25, 58.26)	0.02 (0, 0.15)
Atherosclerosis	0 (0)	0 (0 to 60475.18)	0 (0, 0)	1 (Reference)	0 (0)	0 (0 to 1899.5)	-0.01 (-0.01, -0.01)	..	0 (0)	0 (0 to 105.7)	-0.08 (-0.08, -0.08)	..	1 (0.29)	2.44 (0.06 to 13.6)	2.44 (-5.65, 10.52)	..
Aortic Aneurysm and Dissection	0 (0)	0 (0 to 2741.69)	-0.01 (-0.01, -0.01)	1 (Reference)	0 (0)	0 (0 to 167.1)	-0.06 (-0.06, -0.06)	..	0 (0)	0 (0 to 27.45)	-0.3 (-0.29, -0.29)	..	2 (0.58)	3.98 (0.48 to 14.39)	6.18 (-5.26, 17.62)	..
Other Diseases of Arteries, Arterioles, Capillaries	0 (0)	0 (0 to 5191.88)	-0.01 (-0.01, -0.01)	1 (Reference)	0 (0)	0 (0 to 388.84)	-0.03 (-0.03, -0.03)	..	0 (0)	0 (0 to 39.53)	-0.21 (-0.2, -0.2)	..	0 (0)	0 (0 to 7.87)	-1.93 (-1.93, -1.93)	..
Pneumonia and Influenza	0 (0)	0 (0 to 478.88)	-0.05 (-0.06, -0.06)	1 (Reference)	0 (0)	0 (0 to 53.25)	-0.19 (-0.19, -0.19)	..	2 (0.63)	4.46 (0.54 to 16.12)	3.4 (-2.68, 9.49)	..	9 (2.6)	3.11 (1.42 to 5.91)	25.21 (0.94, 49.47)	..
Chronic Obstructive Pulmonary Disease and Allied Cond	0 (0)	0 (0 to 594.85)	-0.04 (-0.04, -0.04)	1 (Reference)	1 (0.32)	12.68 (0.32 to 70.63)	2.58 (-2.91, 8.06)	..	2 (0.63)	1.34 (0.16 to 4.85)	1.12 (-4.96, 7.2)	..	4 (1.16)	0.57 (0.16 to 1.47)	-12.31 (-28.48, 3.87)	..
Chronic Liver Disease and Cirrhosis	0 (0)	0 (0 to 751.97)	-0.03 (-0.03, -0.03)	1 (Reference)	0 (0)	0 (0 to 18.36)	-0.56 (-0.56, -0.56)	..	1 (0.32)	0.89 (0.02 to 4.96)	-0.27 (-4.57, 4.03)	..	1 (0.29)	1.44 (0.04 to 7.99)	1.25 (-6.84, 9.34)	..
Nephritis, Nephrotic Syndrome and Nephrosis	0 (0)	0 (0 to 1232.23)	-0.02 (-0.02, -0.02)	1 (Reference)	0 (0)	0 (0 to 66.67)	-0.16 (-0.15, -0.15)	..	1 (0.32)	2.01 (0.05 to 11.18)	1.1 (-3.2, 5.4)	..	3 (0.87)	1.25 (0.26 to 3.65)	2.48 (-11.53, 16.49)	..
Symptoms, Signs and Ill-Defined Conditions	1 (1.1)	39.75 (1.01 to 221.49)	6.74 (-6.81, 20.28)	1 (Reference)	3 (0.96)	26.25 (5.41 to 76.7)	8.07 (-1.42, 17.57)	0.74 (0.08, 7.22)	0 (0)	0 (0 to 11.71)	-0.69 (-0.69, -0.69)	..	2 (0.58)	1.44 (0.18 to 5.21)	2.53 (-8.9, 13.97)	0.03 (0, 0.36)
Accidents and Adverse Effects	0 (0)	0 (0 to 9.18)	-2.78 (-2.78, -2.78)	1 (Reference)	2 (0.64)	1.27 (0.15 to 4.59)	1.19 (-6.56, 8.95)	..	2 (0.63)	0.95 (0.12 to 3.42)	-0.25 (-6.33, 5.83)	..	2 (0.58)	0.73 (0.09 to 2.64)	-3.03 (-14.47, 8.4)	..
Suicide and Self-Inflicted Injury	0 (0)	0 (0 to 26.18)	-0.97 (-0.97, -0.97)	1 (Reference)	2 (0.64)	3.44 (0.42 to 12.43)	3.97 (-3.79, 11.73)	..	1 (0.32)	1.21 (0.03 to 6.76)	0.39 (-3.91, 4.69)	..	0 (0)	0 (0 to 8.99)	-1.69 (-1.7, -1.7)	..
Other COD	4 (4.4)	13.92 (3.79 to 35.65)	25.65 (-1.43, 52.73)	1 (Reference)	30 (9.65)	24.7 (16.67 to 35.26)	80.54 (50.5, 110.58)	1.79 (0.63, 5.07)	13 (4.13)	2.99 (1.59 to 5.11)	18.98 (3.48, 34.48)	0.24 (0.08, 0.75)	36 (10.4)	1.87 (1.31 to 2.59)	69.02 (20.5, 117.54)	0.13 (0.05, 0.37)
Syphilis	0 (0)	0 (0 to 345694.12)	0 (0, 0)	1 (Reference)	0 (0)	0 (0 to 38072.53)	0 (0, 0)	..	0 (0)	0 (0 to 5415.8)	0 (0, 0)	..	0 (0)	0 (0 to 2071.88)	-0.01 (-0.01, -0.01)	..
Tuberculosis	0 (0)	0 (0 to 20429.6)	0 (0, 0)	1 (Reference)	0 (0)	0 (0 to 1973.96)	-0.01 (-0.01, -0.01)	..	0 (0)	0 (0 to 338.01)	-0.02 (-0.02, -0.02)	..	0 (0)	0 (0 to 144.92)	-0.11 (-0.1, -0.1)	..
Stomach and Duodenal Ulcers	0 (0)	0 (0 to 12729.89)	0 (0, 0)	1 (Reference)	0 (0)	0 (0 to 636.39)	-0.02 (-0.02, -0.02)	..	0 (0)	0 (0 to 80.81)	-0.1 (-0.1, -0.1)	..	0 (0)	0 (0 to 25.6)	-0.6 (-0.59, -0.59)	..
Puerperium	0 (0)	0 (0 to 1104.4)	-0.02 (-0.02, -0.02)	1 (Reference)	0 (0)	0 (0 to 212.23)	-0.05 (-0.05, -0.05)	..	0 (0)	0 (0 to 813.07)	-0.01 (-0.01, -0.01)	..	0 (0)	0 (0 to 181108.44)	0 (0, 0)	..
Congenital Anomalies	3 (3.3)	116.65 (24.06 to 340.9)	20.55 (-2.91, 44)	1 (Reference)	23 (7.4)	569.76 (361.18 to 854.92)	64.24 (37.94, 90.54)	6.74 (2.02, 22.56)	6 (1.9)	66.76 (24.5 to 145.32)	12.97 (2.43, 23.5)	0.86 (0.21, 3.48)	12 (3.47)	178.7 (92.34 to 312.15)	49.23 (21.22, 77.25)	1.94 (0.55, 6.9)
Certain Conditions Originating in. Perinatal Period	0 (0)	0 (0 to 140.56)	-0.18 (-0.18, -0.18)	1 (Reference)	0 (0)	0 (0 to 7368.33)	0 (0, 0)	..	0 (0)	0 (0 to 7954.89)	0 (0, 0)	..	0 (0)	0 (0 to 14993.04)	0 (0, 0)	..
Homicide and Legal Intervention	0 (0)	0 (0 to 28.06)	-0.91 (-0.91, -0.91)	1 (Reference)	0 (0)	0 (0 to 9.93)	-1.04 (-1.04, -1.04)	..	0 (0)	0 (0 to 18.7)	-0.43 (-0.43, -0.43)	..	0 (0)	0 (0 to 67.34)	-0.23 (-0.23, -0.23)	..
Other Cause of Death	1 (1.1)	10 (0.25 to 55.69)	6.22 (-7.32, 19.76)	1 (Reference)	7 (2.25)	9.01 (3.62 to 18.56)	17.41 (2.9, 31.92)	0.86 (0.11, 7)	7 (2.22)	1.75 (0.7 to 3.61)	6.58 (-4.79, 17.96)	0.17 (0.02, 1.39)	24 (6.94)	1.27 (0.81 to 1.88)	20.72 (-18.9, 60.34)	0.11 (0.02, 0.83)

eTable32. Causes of death among survivors diagnosed with primary malignant peripheral nerve sheath tumor by latency during 2000-2019 in 17 SEER registers.

COD	0-11 months				12-59 months				60-119 months				≥ 120 months			
	Obs.	SMR (95% CI)	Absolute excess incidence per 10,000 person-year	Relative risk (95%CI	Obs.	SMR (95% CI)	Absolute excess incidence per 10,000 person-year	Relative risk (95%CI	Obs.	SMR (95% CI)	Absolute excess incidence per 10,000 person-year	Relative risk (95%CI	Obs.	SMR (95% CI)	Absolute excess incidence per 10,000 person-year	Relative risk (95%CI)
All Cause of Death	413 (100)	20.65 (18.71 to 22.75)	2130.14 (1914.25, 2346.04)	1 (Reference)	491 (100)	9.24 (8.44 to 10.09)	977.29 (880.35, 1074.23)	0.43 (0.38, 0.5)	114 (100)	2.55 (2.1 to 3.06)	206.27 (143.96, 268.58)	0.11 (0.09, 0.13)	45 (100)	1.28 (0.94 to 1.72)	42.75 (-13.92, 99.43)	0.04 (0.03, 0.06)
All malignant cancers	331 (80.15)	71.7 (64.18 to 79.85)	1769.05 (1575.77, 1962.32)	1 (Reference)	389 (79.23)	32.5 (29.35 to 35.89)	841.54 (755.26, 927.83)	0.4 (0.34, 0.46)	58 (50.88)	5.74 (4.36 to 7.42)	142.62 (98.18, 187.06)	0.06 (0.04, 0.07)	12 (26.67)	1.57 (0.81 to 2.75)	18.85 (-10.42, 48.12)	0.01 (0.01, 0.02)
Head and Neck	5 (1.21)	46.64 (15.14 to 108.84)	26.52 (2.77, 50.28)	1 (Reference)	3 (0.61)	10.81 (2.23 to 31.6)	6.08 (-1.5, 13.66)	0.19 (0.04, 0.79)	1 (0.88)	4.19 (0.11 to 23.35)	2.27 (-3.57, 8.1)	0.05 (0.01, 0.48)	0 (0)	0 (0 to 19.73)	-0.81 (-0.81, -0.81)	..
Esophagus	0 (0)	0 (0 to 30.72)	-0.65 (-0.65, -0.65)	1 (Reference)	0 (0)	0 (0 to 11.93)	-0.69 (-0.69, -0.69)	..	0 (0)	0 (0 to 13.84)	-0.79 (-0.79, -0.79)	..	0 (0)	0 (0 to 18)	-0.88 (-0.88, -0.88)	..
Stomach	2 (0.48)	20.11 (2.44 to 72.63)	10.3 (-4.72, 25.33)	1 (Reference)	3 (0.61)	11.6 (2.39 to 33.91)	6.12 (-1.46, 13.7)	0.57 (0.1, 3.44)	0 (0)	0 (0 to 17.85)	-0.62 (-0.62, -0.62)	..	0 (0)	0 (0 to 24.48)	-0.65 (-0.65, -0.65)	..
Small Intestine	0 (0)	0 (0 to 361.34)	-0.06 (-0.05, -0.05)	1 (Reference)	0 (0)	0 (0 to 135.71)	-0.06 (-0.06, -0.06)	..	0 (0)	0 (0 to 155.13)	-0.07 (-0.07, -0.07)	..	0 (0)	0 (0 to 180.31)	-0.09 (-0.09, -0.09)	..
Colon and Rectum	1 (0.24)	2.31 (0.06 to 12.84)	3.07 (-7.56, 13.69)	1 (Reference)	2 (0.41)	1.78 (0.22 to 6.45)	1.96 (-4.22, 8.15)	0.83 (0.08, 9.17)	0 (0)	0 (0 to 4.02)	-2.73 (-2.73, -2.73)	..	2 (4.44)	2.96 (0.36 to 10.7)	5.71 (-6.24, 17.66)	1.46 (0.12, 17.7)
Anus, Anal Canal and Anorectum	0 (0)	0 (0 to 618.17)	-0.03 (-0.03, -0.03)	1 (Reference)	0 (0)	0 (0 to 228.75)	-0.04 (-0.04, -0.04)	..	0 (0)	0 (0 to 243.07)	-0.05 (-0.04, -0.04)	..	0 (0)	0 (0 to 274.77)	-0.06 (-0.06, -0.06)	..
Liver and intrahepatic bile duct	3 (0.73)	17.6 (3.63 to 51.43)	15.34 (-3.06, 33.74)	1 (Reference)	2 (0.41)	4.38 (0.53 to 15.82)	3.45 (-2.74, 9.63)	0.26 (0.04, 1.57)	0 (0)	0 (0 to 8.77)	-1.25 (-1.25, -1.25)	..	0 (0)	0 (0 to 10.49)	-1.52 (-1.52, -1.52)	..
Gallbladder and other biliary	0 (0)	0 (0 to 126.68)	-0.16 (-0.16, -0.16)	1 (Reference)	0 (0)	0 (0 to 48.24)	-0.17 (-0.17, -0.17)	..	0 (0)	0 (0 to 56.15)	-0.2 (-0.2, -0.2)	..	0 (0)	0 (0 to 71.71)	-0.22 (-0.22, -0.22)	..
Pancreas	0 (0)	0 (0 to 12.61)	-1.59 (-1.59, -1.59)	1 (Reference)	0 (0)	0 (0 to 4.75)	-1.73 (-1.73, -1.73)	..	3 (2.63)	4.36 (0.9 to 12.74)	6.88 (-3.22, 16.99)	..	0 (0)	0 (0 to 6.59)	-2.42 (-2.41, -2.41)	..
Retroperitoneum, peritoneum and other digestive system	3 (0.73)	377.87 (77.93 to 1104.28)	16.22 (-2.18, 34.62)	1 (Reference)	2 (0.41)	95.4 (11.55 to 344.6)	4.42 (-1.77, 10.6)	0.42 (0.07, 2.6)	0 (0)	0 (0 to 195.72)	-0.06 (-0.06, -0.06)	..	0 (0)	0 (0 to 231.18)	-0.07 (-0.07, -0.07)	..
Lung and Bronchus	24 (5.81)	18.83 (12.07 to 28.02)	123.18 (71.13, 175.22)	1 (Reference)	25 (5.09)	7.73 (5 to 11.41)	48.58 (26.71, 70.45)	0.35 (0.2, 0.62)	6 (5.26)	2.22 (0.82 to 4.84)	9.83 (-4.47, 24.12)	0.07 (0.03, 0.18)	0 (0)	0 (0 to 1.92)	-8.27 (-8.26, -8.26)	..
Pleura, trachea and other respiratory	1 (0.24)	235.94 (5.97 to 1314.6)	5.4 (-5.23, 16.02)	1 (Reference)	1 (0.2)	96.83 (2.45 to 539.52)	2.21 (-2.17, 6.58)	0.71 (0.04, 11.79)	0 (0)	0 (0 to 431.78)	-0.03 (-0.03, -0.03)	..	0 (0)	0 (0 to 488.07)	-0.03 (-0.03, -0.03)	..
Bones and Joints	7 (1.69)	682.53 (274.41 to 1406.28)	37.89 (9.78, 65.99)	1 (Reference)	11 (2.24)	421.56 (210.44 to 754.28)	24.49 (9.98, 39)	0.53 (0.2, 1.37)	1 (0.88)	46.36 (1.17 to 258.31)	2.91 (-2.92, 8.75)	0.06 (0.01, 0.46)	0 (0)	0 (0 to 209.06)	-0.08 (-0.08, -0.08)	..
Soft Tissue including Heart	215 (52.06)	6528.08 (5684.58 to 7461.46)	1165.16 (1009.38, 1320.93)	1 (Reference)	264 (53.77)	3105.46 (2742.12 to 3503.55)	589.07 (517.99, 660.15)	0.38 (0.32, 0.46)	30 (26.32)	413.02 (278.66 to 589.61)	89.11 (57.14, 121.07)	0.05 (0.03, 0.07)	3 (6.67)	52.89 (10.91 to 154.56)	12.69 (-1.95, 27.32)	0.01 (0, 0.02)
Melanoma of the Skin	3 (0.73)	43.49 (8.97 to 127.1)	15.89 (-2.51, 34.29)	1 (Reference)	10 (2.04)	56.73 (27.2 to 104.32)	21.93 (8.09, 35.76)	1.23 (0.34, 4.47)	1 (0.88)	6.66 (0.17 to 37.1)	2.53 (-3.3, 8.37)	0.14 (0.01, 1.31)	1 (2.22)	9.5 (0.24 to 52.91)	3.86 (-4.59, 12.31)	0.18 (0.02, 1.74)
Breast	0 (0)	0 (0 to 12.64)	-1.58 (-1.58, -1.58)	1 (Reference)	0 (0)	0 (0 to 4.68)	-1.76 (-1.76, -1.76)	..	0 (0)	0 (0 to 5.68)	-1.93 (-1.93, -1.93)	..	0 (0)	0 (0 to 7.45)	-2.14 (-2.13, -2.13)	..
Cervix Uteri	0 (0)	0 (0 to 126.45)	-0.34 (-0.34, -0.34)	1 (Reference)	1 (0.2)	12.94 (0.33 to 72.09)	4.25 (-4.78, 13.29)	..	0 (0)	0 (0 to 60.42)	-0.37 (-0.37, -0.37)	..	0 (0)	0 (0 to 83.53)	-0.4 (-0.4, -0.4)	..
Corpus and Uterus, NOS	0 (0)	0 (0 to 61.52)	-0.7 (-0.7, -0.7)	1 (Reference)	1 (0.2)	5.95 (0.15 to 33.15)	3.84 (-5.2, 12.87)	..	0 (0)	0 (0 to 25.42)	-0.89 (-0.88, -0.88)	..	0 (0)	0 (0 to 29.68)	-1.13 (-1.13, -1.13)	..
Ovary	0 (0)	0 (0 to 37.14)	-1.15 (-1.15, -1.15)	1 (Reference)	0 (0)	0 (0 to 13.83)	-1.23 (-1.23, -1.23)	..	1 (0.88)	4.49 (0.11 to 25.02)	4.74 (-7.21, 16.69)	..	1 (2.22)	6.1 (0.15 to 33.99)	7.6 (-10.21, 25.41)	..
Vagina, vulva and other female genital organs	0 (0)	0 (0 to 286.47)	-0.15 (-0.15, -0.15)	1 (Reference)	0 (0)	0 (0 to 104.36)	-0.16 (-0.16, -0.16)	..	0 (0)	0 (0 to 112.43)	-0.2 (-0.2, -0.2)	..	0 (0)	0 (0 to 126.32)	-0.27 (-0.26, -0.26)	..
Prostate	1 (0.24)	3.77 (0.1 to 20.98)	7.48 (-12.49, 27.44)	1 (Reference)	0 (0)	0 (0 to 5.32)	-3 (-3, -3)	..	0 (0)	0 (0 to 6.4)	-3.35 (-3.35, -3.35)	..	0 (0)	0 (0 to 8.31)	-3.64 (-3.64, -3.64)	..
Testis	0 (0)	0 (0 to 1284.98)	-0.03 (-0.03, -0.03)	1 (Reference)	0 (0)	0 (0 to 544.8)	-0.03 (-0.03, -0.03)	..	0 (0)	0 (0 to 719.34)	-0.03 (-0.03, -0.03)	..	0 (0)	0 (0 to 955.09)	-0.03 (-0.03, -0.03)	..
Penis and other male genital organs	0 (0)	0 (0 to 1327.38)	-0.03 (-0.03, -0.03)	1 (Reference)	0 (0)	0 (0 to 508.79)	-0.03 (-0.03, -0.03)	..	0 (0)	0 (0 to 567.64)	-0.04 (-0.03, -0.03)	..	0 (0)	0 (0 to 696.73)	-0.04 (-0.04, -0.04)	..
Urinary Bladder	0 (0)	0 (0 to 29.54)	-0.68 (-0.68, -0.68)	1 (Reference)	1 (0.2)	3.03 (0.08 to 16.87)	1.5 (-2.88, 5.87)	..	1 (0.88)	3.53 (0.09 to 19.67)	2.13 (-3.7, 7.97)	..	1 (2.22)	4.58 (0.12 to 25.53)	3.37 (-5.08, 11.82)	..
Kidney and Renal Pelvis	1 (0.24)	9.24 (0.23 to 51.47)	4.83 (-5.79, 15.46)	1 (Reference)	1 (0.2)	3.58 (0.09 to 19.94)	1.61 (-2.77, 5.98)	0.05 (0, 2.48)	0 (0)	0 (0 to 15.33)	-0.72 (-0.72, -0.72)	..	0 (0)	0 (0 to 20.11)	-0.79 (-0.79, -0.79)	..
Ureter and other urinary organs	0 (0)	0 (0 to 590.69)	-0.03 (-0.03, -0.03)	1 (Reference)	0 (0)	0 (0 to 220.16)	-0.04 (-0.04, -0.04)	..	0 (0)	0 (0 to 247.84)	-0.04 (-0.04, -0.04)	..	0 (0)	0 (0 to 312.95)	-0.05 (-0.05, -0.05)	..
Eye and Orbit	1 (0.24)	469.34 (11.88 to 2614.98)	5.41 (-5.21, 16.03)	1 (Reference)	0 (0)	0 (0 to 669.8)	-0.01 (-0.01, -0.01)	..	0 (0)	0 (0 to 728.04)	-0.02 (-0.01, -0.01)	..	0 (0)	0 (0 to 900.72)	-0.02 (-0.02, -0.02)	..
Brain and Other Nervous System	12 (2.91)	109.38 (56.52 to 191.07)	64.45 (27.64, 101.25)	1 (Reference)	13 (2.65)	46.34 (24.68 to 79.25)	28.39 (12.62, 44.16)	0.4 (0.18, 0.89)	3 (2.63)	12.19 (2.51 to 35.61)	8.2 (-1.91, 18.31)	0.1 (0.03, 0.36)	1 (2.22)	5.12 (0.13 to 28.53)	3.47 (-4.98, 11.92)	0.03 (0, 0.27)
Thyroid	0 (0)	0 (0 to 279.7)	-0.07 (-0.07, -0.07)	1 (Reference)	0 (0)	0 (0 to 104.46)	-0.08 (-0.08, -0.08)	..	0 (0)	0 (0 to 116.37)	-0.09 (-0.1, -0.1)	..	0 (0)	0 (0 to 145.08)	-0.11 (-0.11, -0.11)	..
Other endocrine system	0 (0)	0 (0 to 574.38)	-0.04 (-0.03, -0.03)	1 (Reference)	4 (0.81)	242.21 (65.99 to 620.15)	8.89 (0.14, 17.64)	..	0 (0)	0 (0 to 268.35)	-0.04 (-0.04, -0.04)	..	0 (0)	0 (0 to 342.58)	-0.05 (-0.05, -0.05)	..
Hodgkin Lymphoma	0 (0)	0 (0 to 394.49)	-0.05 (-0.05, -0.05)	1 (Reference)	0 (0)	0 (0 to 161.04)	-0.05 (-0.05, -0.05)	..	0 (0)	0 (0 to 207.76)	-0.05 (-0.05, -0.05)	..	0 (0)	0 (0 to 296.85)	-0.05 (-0.05, -0.05)	..
Non-Hodgkin Lymphoma	2 (0.48)	9.65 (1.17 to 34.87)	9.72 (-5.31, 24.74)	1 (Reference)	3 (0.61)	5.71 (1.18 to 16.69)	5.52 (-2.05, 13.1)	0.56 (0.09, 3.36)	3 (2.63)	6.88 (1.42 to 20.12)	7.64 (-2.47, 17.74)	0.61 (0.1, 3.71)	0 (0)	0 (0 to 11.47)	-1.39 (-1.39, -1.39)	..
Myeloma	0 (0)	0 (0 to 39.7)	-0.5 (-0.5, -0.5)	1 (Reference)	1 (0.2)	4.14 (0.11 to 23.04)	1.69 (-2.68, 6.07)	..	0 (0)	0 (0 to 17.92)	-0.61 (-0.61, -0.61)	..	0 (0)	0 (0 to 22.6)	-0.7 (-0.7, -0.7)	..
Acute Lymphocytic Leukemia	0 (0)	0 (0 to 375.95)	-0.05 (-0.05, -0.05)	1 (Reference)	0 (0)	0 (0 to 152.36)	-0.05 (-0.05, -0.05)	..	0 (0)	0 (0 to 187.77)	-0.06 (-0.06, -0.06)	..	0 (0)	0 (0 to 254.17)	-0.06 (-0.06, -0.06)	..
Acute Non-Lymphocytic Leukemia	0 (0)	0 (0 to 41.01)	-0.49 (-0.49, -0.49)	1 (Reference)	0 (0)	0 (0 to 16.05)	-0.51 (-0.51, -0.51)	..	0 (0)	0 (0 to 18.73)	-0.59 (-0.59, -0.59)	..	0 (0)	0 (0 to 24.44)	-0.65 (-0.65, -0.65)	..
Chronic Myeloid Leukemia	0 (0)	0 (0 to 397.41)	-0.05 (-0.05, -0.05)	1 (Reference)	0 (0)	0 (0 to 162.93)	-0.05 (-0.05, -0.05)	..	0 (0)	0 (0 to 200.84)	-0.06 (-0.05, -0.05)	..	0 (0)	0 (0 to 255.58)	-0.06 (-0.06, -0.06)	..
Others	50 (12.11)	122.36 (90.82 to 161.32)	268.79 (193.67, 343.91)	1 (Reference)	41 (8.35)	38.81 (27.85 to 52.65)	89.16 (61.14, 117.17)	0.24 (0.16, 0.36)	8 (7.02)	9.07 (3.92 to 17.87)	21.19 (4.69, 37.7)	0.05 (0.02, 0.1)	3 (6.67)	4.5 (0.93 to 13.15)	10.06 (-4.58, 24.69)	0.02 (0, 0.05)
In situ, benign or unknown behavior neoplasm	22 (5.33)	186.97 (117.18 to 283.08)	118.61 (68.78, 168.43)	1 (Reference)	29 (5.91)	92.91 (62.22 to 133.44)	64.03 (40.47, 87.59)	0.33 (0.18, 0.59)	9 (7.89)	33.73 (15.43 to 64.03)	26 (8.49, 43.51)	0.14 (0.07, 0.31)	0 (0)	0 (0 to 18.06)	-0.88 (-0.88, -0.88)	..
Septicemia	1 (0.24)	3.51 (0.09 to 19.58)	3.88 (-6.75, 14.5)	1 (Reference)	1 (0.2)	1.3 (0.03 to 7.22)	0.51 (-3.87, 4.88)	0.37 (0.02, 5.98)	2 (1.75)	3.06 (0.37 to 11.04)	4.01 (-4.25, 12.26)	0.97 (0.09, 10.68)	0 (0)	0 (0 to 7.16)	-2.22 (-2.22, -2.22)	..
Other Infectious and Parasitic Diseases including HIV	4 (0.97)	17.26 (4.7 to 44.2)	20.43 (-0.82, 41.67)	1 (Reference)	0 (0)	0 (0 to 6.24)	-1.32 (-1.32, -1.32)	..	0 (0)	0 (0 to 7.91)	-1.39 (-1.39, -1.39)	..	0 (0)	0 (0 to 11.67)	-1.36 (-1.36, -1.36)	..
Diabetes Mellitus	1 (0.24)	1.63 (0.04 to 9.07)	2.09 (-8.53, 12.72)	1 (Reference)	2 (0.41)	1.23 (0.15 to 4.42)	0.82 (-5.37, 7.01)	0.73 (0.07, 8.09)	1 (0.88)	0.74 (0.02 to 4.12)	-1.05 (-6.89, 4.78)	0.45 (0.03, 7.25)	0 (0)	0 (0 to 3.44)	-4.63 (-4.63, -4.63)	..
Alzheimer's (ICD-9 and 10 only)	0 (0)	0 (0 to 6.04)	-3.31 (-3.31, -3.31)	1 (Reference)	2 (0.41)	1.12 (0.14 to 4.04)	0.48 (-5.71, 6.66)	..	1 (0.88)	0.59 (0.02 to 3.28)	-2.09 (-7.92, 3.75)	..	5 (11.11)	3.22 (1.05 to 7.52)	14.87 (-4.02, 33.76)	..
CVD	16 (3.87)	2.35 (1.34 to 3.81)	49.79 (7.3, 92.28)	1 (Reference)	24 (4.89)	1.33 (0.85 to 1.98)	13.22 (-8.21, 34.65)	0.57 (0.3, 1.07)	14 (12.28)	0.96 (0.53 to 1.61)	-1.68 (-23.51, 20.16)	0.41 (0.2, 0.84)	16 (35.56)	1.43 (0.82 to 2.32)	20.6 (-13.2, 54.4)	0.58 (0.29, 1.18)
Diseases of Heart	12 (2.91)	2.3 (1.19 to 4.02)	36.8 (0, 73.6)	1 (Reference)	16 (3.26)	1.16 (0.66 to 1.88)	4.89 (-12.61, 22.39)	0.5 (0.24, 1.06)	10 (8.77)	0.9 (0.43 to 1.66)	-3.3 (-21.75, 15.16)	0.38 (0.16, 0.89)	7 (15.56)	0.82 (0.33 to 1.7)	-6.46 (-28.82, 15.89)	0.33 (0.13, 0.84)
Hypertension without Heart Disease	0 (0)	0 (0 to 17.61)	-1.14 (-1.14, -1.14)	1 (Reference)	1 (0.2)	1.64 (0.04 to 9.11)	0.87 (-3.51, 5.24)	..	1 (0.88)	1.82 (0.05 to 10.16)	1.34 (-4.49, 7.18)	..	2 (4.44)	4.31 (0.52 to 15.57)	6.62 (-5.33, 18.57)	..
Cerebrovascular Diseases	4 (0.97)	3.49 (0.95 to 8.94)	15.47 (-5.78, 36.72)	1 (Reference)	4 (0.81)	1.32 (0.36 to 3.38)	2.17 (-6.58, 10.92)	0.37 (0.09, 1.5)	3 (2.63)	1.23 (0.25 to 3.59)	1.67 (-8.44, 11.77)	0.35 (0.08, 1.57)	7 (15.56)	3.64 (1.46 to 7.5)	21.89 (-0.46, 44.24)	0.99 (0.28, 3.52)
Atherosclerosis	0 (0)	0 (0 to 52.32)	-0.38 (-0.38, -0.38)	1 (Reference)	1 (0.2)	5.55 (0.14 to 30.91)	1.83 (-2.54, 6.21)	..	0 (0)	0 (0 to 30.83)	-0.36 (-0.36, -0.36)	..	0 (0)	0 (0 to 48.48)	-0.33 (-0.33, -0.33)	..
Aortic Aneurysm and Dissection	0 (0)	0 (0 to 36.37)	-0.55 (-0.55, -0.55)	1 (Reference)	2 (0.41)	8.08 (0.98 to 29.2)	3.91 (-2.27, 10.1)	..	0 (0)	0 (0 to 20.14)	-0.55 (-0.54, -0.54)	..	0 (0)	0 (0 to 28.84)	-0.55 (-0.55, -0.55)	..
Other Diseases of Arteries, Arterioles, Capillaries	0 (0)	0 (0 to 48.83)	-0.41 (-0.41, -0.41)	1 (Reference)	0 (0)	0 (0 to 18.27)	-0.45 (-0.45, -0.45)	..	0 (0)	0 (0 to 22.54)	-0.49 (-0.49, -0.49)	..	0 (0)	0 (0 to 28.19)	-0.56 (-0.56, -0.56)	..
Pneumonia and Influenza	2 (0.48)	4.32 (0.52 to 15.59)	8.33 (-6.69, 23.35)	1 (Reference)	5 (1.02)	3.99 (1.3 to 9.31)	8.36 (-1.42, 18.15)	0.93 (0.18, 4.78)	3 (2.63)	3.05 (0.63 to 8.9)	6 (-4.11, 16.11)	0.69 (0.12, 4.17)	1 (2.22)	1.4 (0.04 to 7.8)	1.23 (-7.22, 9.68)	0.31 (0.03, 3.54)
Chronic Obstructive Pulmonary Disease and Allied Cond	1 (0.24)	0.91 (0.02 to 5.05)	-0.56 (-11.18, 10.07)	1 (Reference)	3 (0.61)	1.04 (0.22 to 3.04)	0.26 (-7.32, 7.84)	1.1 (0.11, 10.58)	3 (2.63)	1.17 (0.24 to 3.43)	1.32 (-8.78, 11.43)	1.05 (0.11, 10.38)	0 (0)	0 (0 to 1.83)	-8.69 (-8.69, -8.69)	..
Chronic Liver Disease and Cirrhosis	0 (0)	0 (0 to 14.18)	-1.41 (-1.41, -1.41)	1 (Reference)	2 (0.41)	2.95 (0.36 to 10.64)	2.95 (-3.24, 9.14)	..	0 (0)	0 (0 to 6.15)	-1.79 (-1.78, -1.78)	..	0 (0)	0 (0 to 7.56)	-2.1 (-2.1, -2.1)	..
Nephritis, Nephrotic Syndrome and Nephrosis	1 (0.24)	2.66 (0.07 to 14.84)	3.39 (-7.24, 14.01)	1 (Reference)	1 (0.2)	0.96 (0.02 to 5.36)	-0.09 (-4.46, 4.29)	0.35 (0.02, 5.68)	1 (0.88)	1.15 (0.03 to 6.4)	0.38 (-5.45, 6.22)	0.41 (0.03, 6.65)	1 (2.22)	1.49 (0.04 to 8.31)	1.42 (-7.03, 9.87)	0.51 (0.03, 8.64)
Symptoms, Signs and Ill-Defined Conditions	2 (0.48)	8.54 (1.03 to 30.83)	9.57 (-5.45, 24.6)	1 (Reference)	2 (0.41)	3.03 (0.37 to 10.95)	2.99 (-3.2, 9.18)	0.42 (0.06, 2.99)	2 (1.75)	3.61 (0.44 to 13.04)	4.31 (-3.95, 12.56)	0.5 (0.07, 3.61)	0 (0)	0 (0 to 9.39)	-1.69 (-1.69, -1.69)	..
Accidents and Adverse Effects	2 (0.48)	2.15 (0.26 to 7.77)	5.8 (-9.22, 20.83)	1 (Reference)	3 (0.61)	1.26 (0.26 to 3.69)	1.4 (-6.18, 8.98)	0.6 (0.1, 3.57)	1 (0.88)	0.51 (0.01 to 2.85)	-2.84 (-8.67, 3)	0.24 (0.02, 2.7)	0 (0)	0 (0 to 2.35)	-6.77 (-6.76, -6.76)	..
Suicide and Self-Inflicted Injury	1 (0.24)	3.55 (0.09 to 19.79)	3.89 (-6.73, 14.52)	1 (Reference)	0 (0)	0 (0 to 5.33)	-1.55 (-1.54, -1.54)	..	1 (0.88)	1.78 (0.05 to 9.94)	1.31 (-4.53, 7.14)	0.54 (0.03, 8.66)	1 (2.22)	2.37 (0.06 to 13.19)	2.49 (-5.96, 10.94)	0.69 (0.04, 11.08)
Other COD	29 (7.02)	9.56 (6.4 to 13.73)	140.74 (83.54, 197.95)	1 (Reference)	27 (5.5)	3.23 (2.13 to 4.7)	41.61 (18.87, 64.34)	0.36 (0.21, 0.61)	18 (15.79)	2.41 (1.43 to 3.8)	31.33 (6.57, 56.09)	0.27 (0.15, 0.48)	9 (20)	1.44 (0.66 to 2.73)	11.85 (-13.5, 37.2)	0.14 (0.07, 0.31)
Syphilis	0 (0)	0 (0 to 11409.89)	0 (0, 0)	1 (Reference)	0 (0)	0 (0 to 4255.59)	0 (0, 0)	..	0 (0)	0 (0 to 4823.01)	0 (0, 0)	..	0 (0)	0 (0 to 6006.42)	0 (0, 0)	..
Tuberculosis	0 (0)	0 (0 to 639.86)	-0.03 (-0.03, -0.03)	1 (Reference)	0 (0)	0 (0 to 249.19)	-0.03 (-0.03, -0.03)	..	0 (0)	0 (0 to 338.69)	-0.03 (-0.03, -0.03)	..	0 (0)	0 (0 to 530.24)	-0.03 (-0.03, -0.03)	..
Stomach and Duodenal Ulcers	0 (0)	0 (0 to 131.69)	-0.15 (-0.15, -0.15)	1 (Reference)	0 (0)	0 (0 to 52.15)	-0.16 (-0.16, -0.16)	..	0 (0)	0 (0 to 67.05)	-0.16 (-0.16, -0.16)	..	0 (0)	0 (0 to 87.72)	-0.18 (-0.18, -0.18)	..
Puerperium	0 (0)	0 (0 to 940.27)	-0.02 (-0.02, -0.02)	1 (Reference)	0 (0)	0 (0 to 391.71)	-0.02 (-0.02, -0.02)	..	0 (0)	0 (0 to 504.54)	-0.02 (-0.02, -0.02)	..	0 (0)	0 (0 to 797.26)	-0.02 (-0.02, -0.02)	..
Congenital Anomalies	19 (4.6)	535.16 (322.2 to 835.73)	102.79 (56.48, 149.09)	1 (Reference)	18 (3.67)	217.73 (129.04 to 344.11)	39.99 (21.43, 58.55)	0.42 (0.22, 0.79)	4 (3.51)	66.98 (18.25 to 171.5)	11.73 (0.06, 23.4)	0.13 (0.04, 0.37)	3 (6.67)	66.35 (13.68 to 193.89)	12.74 (-1.9, 27.37)	0.12 (0.03, 0.4)
Certain Conditions Originating in. Perinatal Period	0 (0)	0 (0 to 318.5)	-0.06 (-0.07, -0.07)	1 (Reference)	0 (0)	0 (0 to 243.95)	-0.03 (-0.03, -0.03)	..	0 (0)	0 (0 to 7764.08)	0 (0, 0)	..	0 (0)	0 (0 to 13422.89)	0 (0, 0)	..
Homicide and Legal Intervention	0 (0)	0 (0 to 27.78)	-0.72 (-0.72, -0.72)	1 (Reference)	0 (0)	0 (0 to 12.73)	-0.65 (-0.65, -0.65)	..	0 (0)	0 (0 to 18.49)	-0.59 (-0.59, -0.59)	..	0 (0)	0 (0 to 27.8)	-0.57 (-0.57, -0.57)	..
Other Cause of Death	10 (2.42)	3.55 (1.7 to 6.53)	38.94 (5.35, 72.54)	1 (Reference)	9 (1.83)	1.14 (0.52 to 2.17)	2.51 (-10.62, 15.63)	0.32 (0.13, 0.79)	14 (12.28)	1.96 (1.07 to 3.29)	20.41 (-1.42, 42.25)	0.53 (0.24, 1.2)	6 (13.33)	1 (0.37 to 2.17)	-0.08 (-20.78, 20.61)	0.25 (0.09, 0.68)

eTable33. Causes of death among survivors diagnosed with primary malignant peripheral nerve sheath tumor by race during 2000-2019 in 17 SEER registers.

COD	NHW Obs.	NHW SMR (95% CI)	NHW Absolute excess incidence per 10,000 person-year	NHW Relative risk (95%CI)	NHB Obs.	NHB SMR (95% CI)	NHB Absolute excess incidence per 10,000 person-year	NHB Relative risk (95%CI)
All Cause of Death	633 (100)	5.84 (5.39 to 6.31)	717.73 (650.25, 785.21)	1 (Reference)	161 (100)	8.11 (6.91 to 9.47)	924.24 (761.4, 1087.08)	0.9 (0.75, 1.07)
All malignant cancers	453 (71.56)	18.4 (16.74 to 20.17)	586.17 (529.09, 643.25)	1 (Reference)	115 (71.43)	26.61 (21.97 to 31.94)	724.69 (587.06, 862.31)	1.13 (0.92, 1.39)
Head and Neck	8 (1.26)	13.68 (5.91 to 26.95)	10.15 (2.56, 17.73)	1 (Reference)	0 (0)	0 (0 to 33.89)	-0.71 (-0.71, -0.71)	..
Esophagus	0 (0)	0 (0 to 5.21)	-0.97 (-0.97, -0.97)	1 (Reference)	0 (0)	0 (0 to 44.01)	-0.55 (-0.55, -0.55)	..
Stomach	3 (0.47)	7.03 (1.45 to 20.55)	3.52 (-1.12, 8.17)	1 (Reference)	0 (0)	0 (0 to 28.04)	-0.86 (-0.86, -0.86)	..
Small Intestine	0 (0)	0 (0 to 67.45)	-0.08 (-0.08, -0.08)	1 (Reference)	0 (0)	0 (0 to 251.95)	-0.1 (-0.1, -0.1)	..
Colon and Rectum	3 (0.47)	1.38 (0.28 to 4.02)	1.12 (-3.53, 5.76)	1 (Reference)	0 (0)	0 (0 to 8.1)	-2.98 (-2.99, -2.99)	..
Anus, Anal Canal and Anorectum	0 (0)	0 (0 to 102.45)	-0.05 (-0.05, -0.05)	1 (Reference)	0 (0)	0 (0 to 568.87)	-0.04 (-0.04, -0.04)	..
Liver and intrahepatic bile duct	2 (0.32)	2.17 (0.26 to 7.83)	1.47 (-2.32, 5.27)	1 (Reference)	0 (0)	0 (0 to 19.45)	-1.24 (-1.24, -1.24)	..
Gallbladder and other biliary	0 (0)	0 (0 to 24.88)	-0.2 (-0.2, -0.2)	1 (Reference)	0 (0)	0 (0 to 127.96)	-0.19 (-0.19, -0.19)	..
Pancreas	1 (0.16)	0.61 (0.02 to 3.39)	-0.88 (-3.56, 1.8)	1 (Reference)	0 (0)	0 (0 to 12.11)	-1.99 (-2, -2)	..
Retroperitoneum, peritoneum and other digestive system	3 (0.47)	65.63 (13.53 to 191.78)	4.04 (-0.6, 8.69)	1 (Reference)	0 (0)	0 (0 to 686.61)	-0.04 (-0.03, -0.03)	..
Lung and Bronchus	39 (6.16)	5.75 (4.09 to 7.86)	44.09 (27.34, 60.84)	1 (Reference)	8 (4.97)	7.78 (3.36 to 15.33)	45.65 (9.35, 81.95)	1.23 (0.57, 2.67)
Pleura, trachea and other respiratory	1 (0.16)	42.11 (1.07 to 234.6)	1.34 (-1.35, 4.02)	1 (Reference)	1 (0.62)	362.15 (9.17 to 2017.76)	6.53 (-6.31, 19.36)	14.62 (0.05, 4262.16)
Bones and Joints	12 (1.9)	227.19 (117.39 to 396.85)	16.35 (7.06, 25.64)	1 (Reference)	3 (1.86)	340.7 (70.26 to 995.66)	19.59 (-2.64, 41.81)	3.1 (0.81, 11.77)
Soft Tissue including Heart	276 (43.6)	1601.99 (1418.55 to 1802.57)	377.43 (332.87, 421.98)	1 (Reference)	81 (50.31)	2583.37 (2051.57 to 3210.89)	530.16 (414.66, 645.66)	1.11 (0.86, 1.43)
Melanoma of the Skin	11 (1.74)	25.61 (12.78 to 45.82)	14.46 (5.57, 23.36)	1 (Reference)	1 (0.62)	122.57 (3.1 to 682.91)	6.49 (-6.34, 19.33)	12.42 (1.45, 106.2)
Breast	0 (0)	0 (0 to 2.69)	-1.88 (-1.87, -1.87)	1 (Reference)	0 (0)	0 (0 to 9.56)	-2.53 (-2.53, -2.53)	..
Cervix Uteri	0 (0)	0 (0 to 32.55)	-0.16 (-0.15, -0.15)	1 (Reference)	0 (0)	0 (0 to 80.25)	-0.3 (-0.3, -0.3)	..
Corpus and Uterus, NOS	1 (0.16)	3.5 (0.09 to 19.5)	0.98 (-1.7, 3.66)	1 (Reference)	0 (0)	0 (0 to 33.79)	-0.72 (-0.71, -0.71)	..
Ovary	2 (0.32)	4 (0.48 to 14.43)	2.05 (-1.74, 5.84)	1 (Reference)	0 (0)	0 (0 to 45.2)	-0.53 (-0.54, -0.54)	..
Vagina, vulva and other female genital organs	0 (0)	0 (0 to 49.51)	-0.1 (-0.1, -0.1)	1 (Reference)	0 (0)	0 (0 to 289.31)	-0.08 (-0.09, -0.09)	..
Prostate	1 (0.16)	0.69 (0.02 to 3.85)	-0.61 (-3.29, 2.07)	1 (Reference)	0 (0)	0 (0 to 10.29)	-2.35 (-2.34, -2.34)	..
Testis	0 (0)	0 (0 to 271.07)	-0.02 (-0.02, -0.02)	1 (Reference)	0 (0)	0 (0 to 2964.43)	-0.01 (-0.01, -0.01)	..
Penis and other male genital organs	0 (0)	0 (0 to 223.79)	-0.02 (-0.02, -0.02)	1 (Reference)	0 (0)	0 (0 to 1340.72)	-0.02 (-0.02, -0.02)	..
Urinary Bladder	2 (0.32)	2.62 (0.32 to 9.48)	1.69 (-2.1, 5.49)	1 (Reference)	0 (0)	0 (0 to 45.73)	-0.53 (-0.53, -0.53)	..
Kidney and Renal Pelvis	0 (0)	0 (0 to 6)	-0.84 (-0.84, -0.84)	1 (Reference)	0 (0)	0 (0 to 45.55)	-0.53 (-0.53, -0.53)	..
Ureter and other urinary organs	0 (0)	0 (0 to 96.93)	-0.05 (-0.05, -0.05)	1 (Reference)	0 (0)	0 (0 to 884.95)	-0.03 (-0.03, -0.03)	..
Eye and Orbit	0 (0)	0 (0 to 268.54)	-0.02 (-0.02, -0.02)	1 (Reference)	1 (0.62)	2123.11 (53.75 to 11829.18)	6.55 (-6.29, 19.38)	..
Brain and Other Nervous System	19 (3)	29.62 (17.83 to 46.25)	25.12 (13.43, 36.81)	1 (Reference)	3 (1.86)	54.46 (11.23 to 159.15)	19.28 (-2.95, 41.51)	2.01 (0.59, 6.85)
Thyroid	0 (0)	0 (0 to 50.59)	-0.1 (-0.1, -0.1)	1 (Reference)	0 (0)	0 (0 to 343.01)	-0.07 (-0.07, -0.07)	..
Other endocrine system	4 (0.63)	129.4 (35.26 to 331.32)	5.43 (0.07, 10.79)	1 (Reference)	0 (0)	0 (0 to 531.92)	-0.05 (-0.05, -0.05)	..
Hodgkin Lymphoma	0 (0)	0 (0 to 80.23)	-0.06 (-0.06, -0.06)	1 (Reference)	0 (0)	0 (0 to 590.63)	-0.04 (-0.04, -0.04)	..
Non-Hodgkin Lymphoma	5 (0.79)	4.36 (1.42 to 10.17)	5.27 (-0.72, 11.27)	1 (Reference)	0 (0)	0 (0 to 31.31)	-0.77 (-0.77, -0.77)	..
Myeloma	0 (0)	0 (0 to 7.77)	-0.65 (-0.65, -0.65)	1 (Reference)	0 (0)	0 (0 to 27.18)	-0.89 (-0.89, -0.89)	..
Acute Lymphocytic Leukemia	0 (0)	0 (0 to 76.69)	-0.07 (-0.07, -0.07)	1 (Reference)	0 (0)	0 (0 to 621.56)	-0.04 (-0.04, -0.04)	..
Acute Non-Lymphocytic Leukemia	0 (0)	0 (0 to 7.31)	-0.69 (-0.69, -0.69)	1 (Reference)	0 (0)	0 (0 to 66.03)	-0.37 (-0.37, -0.37)	..
Chronic Myeloid Leukemia	0 (0)	0 (0 to 77.74)	-0.07 (-0.06, -0.06)	1 (Reference)	0 (0)	0 (0 to 516.56)	-0.05 (-0.05, -0.05)	..
Others	60 (9.48)	27.09 (20.67 to 34.87)	79.07 (58.3, 99.84)	1 (Reference)	17 (10.56)	48.52 (28.26 to 77.68)	109.02 (56.11, 161.93)	1.17 (0.67, 2.04)
In situ, benign or unknown behavior neoplasm	38 (6)	56.33 (39.86 to 77.32)	51.07 (34.54, 67.61)	1 (Reference)	7 (4.35)	81.68 (32.84 to 168.29)	45.27 (11.32, 79.23)	0.58 (0.25, 1.34)
Septicemia	3 (0.47)	2.03 (0.42 to 5.94)	2.08 (-2.56, 6.73)	1 (Reference)	1 (0.62)	2.36 (0.06 to 13.14)	3.77 (-9.06, 16.6)	1.19 (0.12, 11.55)
Other Infectious and Parasitic Diseases including HIV	0 (0)	0 (0 to 3.76)	-1.34 (-1.34, -1.34)	1 (Reference)	3 (1.86)	7.89 (1.63 to 23.04)	17.15 (-5.07, 39.38)	..
Diabetes Mellitus	1 (0.16)	0.33 (0.01 to 1.85)	-2.74 (-5.43, -0.06)	1 (Reference)	1 (0.62)	1.13 (0.03 to 6.3)	0.76 (-12.08, 13.59)	2.75 (0.17, 45.27)
Alzheimer's (ICD-9 and 10 only)	6 (0.95)	1.43 (0.53 to 3.12)	2.48 (-4.09, 9.05)	1 (Reference)	0 (0)	0 (0 to 6.92)	-3.49 (-3.49, -3.49)	..
CVD	55 (8.69)	1.54 (1.16 to 2.01)	26.42 (6.53, 46.31)	1 (Reference)	7 (4.35)	1.01 (0.4 to 2.07)	0.27 (-33.69, 34.22)	0.63 (0.29, 1.4)
Diseases of Heart	38 (6)	1.38 (0.98 to 1.89)	14.24 (-2.29, 30.78)	1 (Reference)	4 (2.48)	0.78 (0.21 to 2)	-7.4 (-33.06, 18.27)	0.52 (0.18, 1.48)
Hypertension without Heart Disease	4 (0.63)	3.53 (0.96 to 9.03)	3.92 (-1.44, 9.29)	1 (Reference)	0 (0)	0 (0 to 9.59)	-2.52 (-2.52, -2.52)	..
Cerebrovascular Diseases	11 (1.74)	1.91 (0.95 to 3.42)	7.18 (-1.72, 16.07)	1 (Reference)	3 (1.86)	2.43 (0.5 to 7.1)	11.56 (-10.67, 33.78)	1.32 (0.36, 4.84)
Atherosclerosis	0 (0)	0 (0 to 11.37)	-0.44 (-0.44, -0.44)	1 (Reference)	0 (0)	0 (0 to 78.11)	-0.31 (-0.31, -0.31)	..
Aortic Aneurysm and Dissection	2 (0.32)	4.09 (0.5 to 14.79)	2.07 (-1.73, 5.86)	1 (Reference)	0 (0)	0 (0 to 53.33)	-0.45 (-0.45, -0.45)	..
Other Diseases of Arteries, Arterioles, Capillaries	0 (0)	0 (0 to 9.31)	-0.54 (-0.54, -0.54)	1 (Reference)	0 (0)	0 (0 to 39.3)	-0.62 (-0.62, -0.62)	..
Pneumonia and Influenza	11 (1.74)	4.62 (2.3 to 8.26)	11.79 (2.89, 20.68)	1 (Reference)	0 (0)	0 (0 to 9.4)	-2.57 (-2.57, -2.57)	..
Chronic Obstructive Pulmonary Disease and Allied Cond	4 (0.63)	0.6 (0.16 to 1.53)	-3.66 (-9.03, 1.7)	1 (Reference)	2 (1.24)	3.05 (0.37 to 11.01)	8.8 (-9.35, 26.95)	3.83 (0.64, 23.05)
Chronic Liver Disease and Cirrhosis	2 (0.32)	1.35 (0.16 to 4.87)	0.71 (-3.09, 4.5)	1 (Reference)	0 (0)	0 (0 to 21.37)	-1.13 (-1.13, -1.13)	..
Nephritis, Nephrotic Syndrome and Nephrosis	4 (0.63)	2.08 (0.57 to 5.33)	2.84 (-2.52, 8.21)	1 (Reference)	0 (0)	0 (0 to 6.14)	-3.93 (-3.93, -3.93)	..
Symptoms, Signs and Ill-Defined Conditions	2 (0.32)	1.54 (0.19 to 5.56)	0.96 (-2.84, 4.75)	1 (Reference)	2 (1.24)	8.14 (0.99 to 29.39)	11.49 (-6.66, 29.63)	3.41 (0.46, 25.25)
Accidents and Adverse Effects	4 (0.63)	0.85 (0.23 to 2.18)	-0.96 (-6.32, 4.4)	1 (Reference)	2 (1.24)	2.6 (0.32 to 9.4)	8.06 (-10.09, 26.21)	2.82 (0.5, 15.9)
Suicide and Self-Inflicted Injury	3 (0.47)	2.09 (0.43 to 6.11)	2.14 (-2.5, 6.79)	1 (Reference)	0 (0)	0 (0 to 33.45)	-0.72 (-0.72, -0.72)	..
Other COD	46 (7.27)	2.59 (1.9 to 3.45)	38.64 (20.45, 56.83)	1 (Reference)	21 (13.04)	6.37 (3.94 to 9.73)	115.9 (57.09, 174.71)	1.32 (0.77, 2.29)
Syphilis	0 (0)	0 (0 to 3759.95)	0 (0, 0)	1 (Reference)	0 (0)	0 (0 to 2812.47)	-0.01 (-0.01, -0.01)	..
Tuberculosis	0 (0)	0 (0 to 224.85)	-0.02 (-0.02, -0.02)	1 (Reference)	0 (0)	0 (0 to 548.99)	-0.04 (-0.05, -0.05)	..
Stomach and Duodenal Ulcers	0 (0)	0 (0 to 26.81)	-0.19 (-0.19, -0.19)	1 (Reference)	0 (0)	0 (0 to 177.66)	-0.14 (-0.14, -0.14)	..
Puerperium	0 (0)	0 (0 to 316.94)	-0.02 (-0.02, -0.02)	1 (Reference)	0 (0)	0 (0 to 505.61)	-0.05 (-0.05, -0.05)	..
Congenital Anomalies	19 (3)	125.21 (75.39 to 195.54)	25.79 (14.1, 37.48)	1 (Reference)	16 (9.94)	621.01 (354.96 to 1008.47)	104.6 (53.26, 155.93)	4.09 (2.08, 8.03)
Certain Conditions Originating in. Perinatal Period	0 (0)	0 (0 to 266.96)	-0.02 (-0.02, -0.02)	1 (Reference)	0 (0)	0 (0 to 782.22)	-0.03 (-0.03, -0.03)	..
Homicide and Legal Intervention	0 (0)	0 (0 to 13.85)	-0.36 (-0.36, -0.36)	1 (Reference)	0 (0)	0 (0 to 10.3)	-2.34 (-2.34, -2.34)	..
Other Cause of Death	27 (4.27)	1.57 (1.04 to 2.29)	13.46 (-0.48, 27.39)	1 (Reference)	5 (3.11)	1.74 (0.57 to 4.06)	13.92 (-14.78, 42.62)	0.93 (0.35, 2.45)

COD	NHAPI Obs.	NHAPI SIR (95% CI)	NHAPI Absolute excess incidence per 10,000 person-year	NHAPI Relative risk (95%CI	Hispanic Obs.	Hispanic SMR (95% CI)	Hispanic Absolute excess incidence per 10,000 person-year	Hispanic Relative risk (95%CI	r heteroge
All Cause of Death	69 (100)	9.1 (7.08 to 11.52)	584.29 (429.4, 739.17)	1.31 (1.02, 1.68)	188 (100)	11.26 (9.71 to 12.99)	867.33 (731.26, 1003.4)	1.27 (1.07, 1.5)	0.002
All malignant cancers	63 (91.3)	35.75 (27.47 to 45.74)	582.57 (434.57, 730.57)	1.59 (1.22, 2.07)	150 (79.79)	42.91 (36.31 to 50.35)	741.78 (620.24, 863.33)	1.39 (1.15, 1.68)	<.001
Head and Neck	0 (0)	0 (0 to 85.86)	-0.41 (-0.41, -0.41)	..	1 (0.53)	14.24 (0.36 to 79.32)	4.71 (-5.22, 14.63)	0.71 (0.09, 5.77)	0.24
Esophagus	0 (0)	0 (0 to 118)	-0.3 (-0.29, -0.29)	..	0 (0)	0 (0 to 49.25)	-0.38 (-0.38, -0.38)	..	>.99
Stomach	0 (0)	0 (0 to 40.3)	-0.87 (-0.88, -0.88)	..	2 (1.06)	33.23 (4.02 to 120.03)	9.82 (-4.21, 23.86)	4.46 (0.68, 29.26)	0.139
Small Intestine	0 (0)	0 (0 to 885.79)	-0.04 (-0.04, -0.04)	..	0 (0)	0 (0 to 469.13)	-0.04 (-0.04, -0.04)	..	>.99
Colon and Rectum	1 (1.45)	5.58 (0.14 to 31.06)	7.81 (-10.84, 26.46)	5.33 (0.54, 52.19)	1 (0.53)	3.13 (0.08 to 17.46)	3.45 (-6.48, 13.37)	3.11 (0.29, 32.96)	0.307
Anus, Anal Canal and Anorectum	0 (0)	0 (0 to 2463.14)	-0.01 (-0.01, -0.01)	..	0 (0)	0 (0 to 558.52)	-0.03 (-0.04, -0.04)	..	>.99
Liver and intrahepatic bile duct	2 (2.9)	12.91 (1.56 to 46.62)	17.55 (-8.82, 43.92)	5.54 (0.78, 39.41)	1 (0.53)	8.25 (0.21 to 45.98)	4.45 (-5.47, 14.37)	4.27 (0.38, 47.99)	0.209
Gallbladder and other biliary	0 (0)	0 (0 to 181.81)	-0.19 (-0.19, -0.19)	..	0 (0)	0 (0 to 152.2)	-0.12 (-0.12, -0.12)	..	>.99
Pancreas	1 (1.45)	7.99 (0.2 to 44.53)	8.32 (-10.32, 26.97)	13.59 (0.85, 217.44)	1 (0.53)	4.22 (0.11 to 23.51)	3.86 (-6.06, 13.79)	5.13 (0.3, 87.52)	0.256
Retroperitoneum, peritoneum and other digestive system	1 (1.45)	272.76 (6.91 to 1519.73)	9.48 (-9.17, 28.12)	2.23 (0.12, 41.36)	1 (0.53)	115.37 (2.92 to 642.77)	5.02 (-4.91, 14.94)	1.89 (0.12, 30)	0.658
Lung and Bronchus	0 (0)	0 (0 to 9.25)	-3.79 (-3.8, -3.8)	..	8 (4.26)	8.92 (3.85 to 17.57)	35.96 (7.9, 64.03)	1.13 (0.51, 2.49)	0.098
Pleura, trachea and other respiratory	0 (0)	0 (0 to 3154.79)	-0.01 (-0.01, -0.01)	..	0 (0)	0 (0 to 1265.8)	-0.02 (-0.02, -0.02)	..	0.469
Bones and Joints	1 (1.45)	268.15 (6.79 to 1494.01)	9.48 (-9.17, 28.12)	5.5 (0.66, 45.55)	3 (1.6)	303.98 (62.69 to 888.36)	15.14 (-2.05, 32.33)	9.34 (2.4, 36.33)	0.017
Soft Tissue including Heart	40 (57.97)	2894.38 (2067.79 to 3941.32)	380.4 (262.47, 498.32)	1.59 (1.11, 2.27)	107 (56.91)	3741.07 (3065.89 to 4520.7)	541.62 (438.97, 644.27)	1.38 (1.09, 1.74)	0.014
Melanoma of the Skin	1 (1.45)	163.17 (4.13 to 909.1)	9.46 (-9.19, 28.1)	13.75 (1.61, 117.68)	2 (1.06)	35.43 (4.29 to 127.98)	9.84 (-4.19, 23.88)	1.2 (0.26, 5.54)	0.113
Breast	0 (0)	0 (0 to 32.69)	-1.07 (-1.07, -1.07)	..	0 (0)	0 (0 to 10.75)	-1.74 (-1.74, -1.74)	..	>.99
Cervix Uteri	1 (1.45)	58.37 (1.48 to 325.21)	9.35 (-9.29, 28)	..	0 (0)	0 (0 to 110.45)	-0.17 (-0.17, -0.17)	..	0.178
Corpus and Uterus, NOS	0 (0)	0 (0 to 126.47)	-0.28 (-0.28, -0.28)	..	0 (0)	0 (0 to 52.23)	-0.36 (-0.36, -0.36)	..	0.781
Ovary	0 (0)	0 (0 to 82.87)	-0.42 (-0.43, -0.43)	..	0 (0)	0 (0 to 30.35)	-0.62 (-0.62, -0.62)	..	0.638
Vagina, vulva and other female genital organs	0 (0)	0 (0 to 801.88)	-0.04 (-0.05, -0.05)	..	0 (0)	0 (0 to 204.06)	-0.09 (-0.09, -0.09)	..	>.99
Prostate	0 (0)	0 (0 to 46.39)	-0.76 (-0.76, -0.76)	..	0 (0)	0 (0 to 40.05)	-0.47 (-0.47, -0.47)	..	0.934
Testis	0 (0)	0 (0 to 5135.44)	-0.01 (-0.01, -0.01)	..	0 (0)	0 (0 to 1249.9)	-0.02 (-0.02, -0.02)	..	>.99
Penis and other male genital organs	0 (0)	0 (0 to 3344.17)	-0.01 (-0.01, -0.01)	..	0 (0)	0 (0 to 2575.77)	-0.01 (-0.01, -0.01)	..	>.99
Urinary Bladder	0 (0)	0 (0 to 114.97)	-0.31 (-0.3, -0.3)	..	1 (0.53)	12.38 (0.31 to 68.98)	4.65 (-5.27, 14.58)	4.46 (0.36, 55.49)	0.579
Kidney and Renal Pelvis	1 (1.45)	27.68 (0.7 to 154.21)	9.17 (-9.48, 27.82)	..	1 (0.53)	12.93 (0.33 to 72.02)	4.67 (-5.25, 14.6)	..	0.037
Ureter and other urinary organs	0 (0)	0 (0 to 1475.52)	-0.02 (-0.03, -0.03)	..	0 (0)	0 (0 to 759.08)	-0.03 (-0.03, -0.03)	..	>.99
Eye and Orbit	0 (0)	0 (0 to 7335.83)	-0.01 (-0.01, -0.01)	..	0 (0)	0 (0 to 1791.91)	-0.01 (-0.01, -0.01)	..	0.428
Brain and Other Nervous System	3 (4.35)	94.52 (19.49 to 276.22)	28.24 (-4.06, 60.53)	3.3 (0.97, 11.21)	4 (2.13)	39.82 (10.85 to 101.96)	19.74 (-0.1, 39.59)	1.11 (0.37, 3.38)	0.313
Thyroid	0 (0)	0 (0 to 379.76)	-0.09 (-0.1, -0.1)	..	0 (0)	0 (0 to 315.12)	-0.06 (-0.06, -0.06)	..	>.99
Other endocrine system	0 (0)	0 (0 to 994.25)	-0.04 (-0.04, -0.04)	..	0 (0)	0 (0 to 663.58)	-0.03 (-0.03, -0.03)	..	0.625
Hodgkin Lymphoma	0 (0)	0 (0 to 1455.85)	-0.02 (-0.03, -0.03)	..	0 (0)	0 (0 to 490.79)	-0.04 (-0.04, -0.04)	..	>.99
Non-Hodgkin Lymphoma	1 (1.45)	14.43 (0.37 to 80.41)	8.85 (-9.79, 27.5)	4.93 (0.55, 44.54)	2 (1.06)	13.09 (1.59 to 47.28)	9.35 (-4.68, 23.39)	2.39 (0.43, 13.12)	0.197
Myeloma	1 (1.45)	35.23 (0.89 to 196.27)	9.24 (-9.4, 27.89)	..	0 (0)	0 (0 to 58.35)	-0.32 (-0.32, -0.32)	..	0.092
Acute Lymphocytic Leukemia	0 (0)	0 (0 to 924.38)	-0.04 (-0.04, -0.04)	..	0 (0)	0 (0 to 377.66)	-0.05 (-0.05, -0.05)	..	>.99
Acute Non-Lymphocytic Leukemia	0 (0)	0 (0 to 104.94)	-0.33 (-0.33, -0.33)	..	0 (0)	0 (0 to 52.77)	-0.35 (-0.35, -0.35)	..	>.99
Chronic Myeloid Leukemia	0 (0)	0 (0 to 1213.7)	-0.03 (-0.03, -0.03)	..	0 (0)	0 (0 to 535.51)	-0.04 (-0.04, -0.04)	..	>.99
Others	9 (13.04)	65.53 (29.97 to 124.4)	84.31 (28.38, 140.25)	2.29 (1.14, 4.63)	15 (7.98)	49.44 (27.67 to 81.55)	74.41 (35.98, 112.85)	1.06 (0.59, 1.91)	0.214
In situ, benign or unknown behavior neoplasm	3 (4.35)	67.28 (13.88 to 196.62)	28.12 (-4.18, 60.41)	0.59 (0.17, 2.06)	11 (5.85)	116.61 (58.21 to 208.65)	55.22 (22.31, 88.13)	0.89 (0.44, 1.79)	0.535
Septicemia	0 (0)	0 (0 to 38.62)	-0.91 (-0.91, -0.91)	..	0 (0)	0 (0 to 16.35)	-1.14 (-1.14, -1.14)	..	0.68
Other Infectious and Parasitic Diseases including HIV	0 (0)	0 (0 to 46.59)	-0.75 (-0.75, -0.75)	..	1 (0.53)	6.32 (0.16 to 35.21)	4.26 (-5.66, 14.19)	..	0.137
Diabetes Mellitus	2 (2.9)	6.07 (0.74 to 21.92)	15.89 (-10.48, 42.26)	15.86 (1.43, 175.99)	0 (0)	0 (0 to 8.39)	-2.23 (-2.23, -2.23)	..	0.107
Alzheimer's (ICD-9 and 10 only)	0 (0)	0 (0 to 18.5)	-1.9 (-1.89, -1.89)	..	2 (1.06)	2.74 (0.33 to 9.9)	6.43 (-7.6, 20.46)	2.25 (0.43, 11.91)	0.365
CVD	0 (0)	0 (0 to 1.4)	-25.15 (-25.15, -25.15)	..	7 (3.72)	1.32 (0.53 to 2.72)	8.62 (-17.63, 34.88)	0.84 (0.38, 1.89)	0.037
Diseases of Heart	0 (0)	0 (0 to 1.99)	-17.68 (-17.69, -17.69)	..	2 (1.06)	0.5 (0.06 to 1.81)	-10.07 (-24.1, 3.97)	0.32 (0.08, 1.36)	0.032
Hypertension without Heart Disease	0 (0)	0 (0 to 29.13)	-1.21 (-1.21, -1.21)	..	0 (0)	0 (0 to 19.92)	-0.94 (-0.94, -0.94)	..	0.307
Cerebrovascular Diseases	0 (0)	0 (0 to 6.38)	-5.5 (-5.5, -5.5)	..	4 (2.13)	4.22 (1.15 to 10.81)	15.46 (-4.39, 35.31)	2.82 (0.83, 9.55)	0.21
Atherosclerosis	0 (0)	0 (0 to 176.71)	-0.2 (-0.2, -0.2)	..	1 (0.53)	18.64 (0.47 to 103.84)	4.79 (-5.13, 14.71)	..	0.356
Aortic Aneurysm and Dissection	0 (0)	0 (0 to 98.31)	-0.36 (-0.36, -0.36)	..	0 (0)	0 (0 to 58.49)	-0.32 (-0.32, -0.32)	..	0.76
Other Diseases of Arteries, Arterioles, Capillaries	0 (0)	0 (0 to 172.37)	-0.2 (-0.2, -0.2)	..	0 (0)	0 (0 to 61.59)	-0.3 (-0.3, -0.3)	..	>.99
Pneumonia and Influenza	0 (0)	0 (0 to 13.69)	-2.56 (-2.57, -2.57)	..	0 (0)	0 (0 to 10.1)	-1.85 (-1.85, -1.85)	..	0.058
Chronic Obstructive Pulmonary Disease and Allied Cond	0 (0)	0 (0 to 13.33)	-2.63 (-2.64, -2.64)	..	1 (0.53)	1.06 (0.03 to 5.92)	0.3 (-9.63, 10.22)	2.04 (0.22, 19.21)	0.5
Chronic Liver Disease and Cirrhosis	0 (0)	0 (0 to 31.54)	-1.11 (-1.11, -1.11)	..	0 (0)	0 (0 to 15.5)	-1.21 (-1.21, -1.21)	..	0.81
Nephritis, Nephrotic Syndrome and Nephrosis	0 (0)	0 (0 to 23.37)	-1.5 (-1.5, -1.5)	..	0 (0)	0 (0 to 13.64)	-1.37 (-1.37, -1.37)	..	0.304
Symptoms, Signs and Ill-Defined Conditions	0 (0)	0 (0 to 53.18)	-0.66 (-0.66, -0.66)	..	2 (1.06)	9.01 (1.09 to 32.55)	9 (-5.03, 23.04)	3.35 (0.46, 24.39)	0.498
Accidents and Adverse Effects	0 (0)	0 (0 to 11.51)	-3.05 (-3.04, -3.04)	..	0 (0)	0 (0 to 3.68)	-5.07 (-5.07, -5.07)	..	0.262
Suicide and Self-Inflicted Injury	0 (0)	0 (0 to 40.39)	-0.87 (-0.87, -0.87)	..	0 (0)	0 (0 to 12.02)	-1.55 (-1.55, -1.55)	..	0.546
Other COD	1 (1.45)	0.89 (0.02 to 4.97)	-1.15 (-19.8, 17.49)	0.29 (0.04, 2.13)	14 (7.45)	4.84 (2.65 to 8.12)	56.23 (19.1, 93.36)	1.39 (0.75, 2.57)	0.213
Syphilis	0 (0)	0 (0 to 29697.63)	0 (0, 0)	..	0 (0)	0 (0 to 25331.38)	0 (0, 0)	..	>.99
Tuberculosis	0 (0)	0 (0 to 303.44)	-0.12 (-0.11, -0.11)	..	0 (0)	0 (0 to 1449.62)	-0.01 (-0.02, -0.02)	..	>.99
Stomach and Duodenal Ulcers	0 (0)	0 (0 to 235.14)	-0.15 (-0.15, -0.15)	..	0 (0)	0 (0 to 173.91)	-0.11 (-0.11, -0.11)	..	>.99
Puerperium	0 (0)	0 (0 to 2625.68)	-0.01 (-0.01, -0.01)	..	0 (0)	0 (0 to 774.27)	-0.02 (-0.03, -0.03)	..	>.99
Congenital Anomalies	1 (1.45)	96.96 (2.46 to 540.23)	9.42 (-9.23, 28.06)	1.72 (0.22, 13.24)	8 (4.26)	233.12 (100.65 to 459.35)	40.33 (12.26, 68.4)	1.39 (0.59, 3.25)	0.002
Certain Conditions Originating in. Perinatal Period	0 (0)	0 (0 to 1281.92)	-0.03 (-0.03, -0.03)	..	0 (0)	0 (0 to 611.64)	-0.03 (-0.03, -0.03)	..	>.99
Homicide and Legal Intervention	0 (0)	0 (0 to 110.53)	-0.32 (-0.31, -0.31)	..	0 (0)	0 (0 to 40.26)	-0.46 (-0.47, -0.47)	..	>.99
Other Cause of Death	0 (0)	0 (0 to 3.53)	-9.94 (-9.94, -9.94)	..	6 (3.19)	2.2 (0.81 to 4.78)	16.54 (-7.77, 40.85)	1.15 (0.47, 2.84)	0.31

eTable54. Causes of death among survivors diagnosed with primary malignant peripheral nerve sheath tumor by radiotherapy during 2000-2019 in 17 SEER registers.

COD	No				Yes				*r heterogen*
	Obs.	SMR (95% CI)	Absolute excess incidence per 10,000 person-year	Relative risk (95%CI)	Obs.	SMR (95% CI)	Absolute excess incidence per 10,000 person-year	Relative risk (95%CI)	
All Cause of Death	562 (100)	6.17 (5.67, 6.7)	638.7 (575.68, 847727.05)	1 (Reference)	501 (100)	8.1 (7.41, 8.84)	948.32 (853.58, 954383.99)	1.29 (1.15, 1.46)	<.001
All malignant cancers	386 (68.68)	18.06 (16.3, 19.95)	494.55 (442.32, 660540.54)	1 (Reference)	404 (80.64)	31.24 (28.26, 34.44)	844.5 (759.43, 850546.36)	1.56 (1.35, 1.79)	<.001
Head and Neck	6 (1.07)	11.57 (4.25, 25.18)	7.44 (0.92, 16847.45)	1 (Reference)	3 (0.6)	10.29 (2.12, 30.06)	5.85 (-1.48, 12058.53)	0.68 (0.17, 2.74)	0.582
Esophagus	0 (0)	0 (0, 6.34)	-0.79 (-0.79, -953.63)	1 (Reference)	0 (0)	0 (0, 11.59)	-0.69 (-0.69, -628.33)	..	>.99
Stomach	3 (0.53)	6.82 (1.41, 19.93)	3.47 (-1.13, 9757.2)	1 (Reference)	2 (0.4)	7.26 (0.88, 26.24)	3.72 (-2.26, 8885.32)	1.03 (0.17, 6.15)	0.976
Small Intestine	0 (0)	0 (0, 72.94)	-0.07 (-0.07, -83.57)	1 (Reference)	0 (0)	0 (0, 118.77)	-0.07 (-0.07, -61.25)	..	>.99
Colon and Rectum	4 (0.71)	2.08 (0.57, 5.32)	2.81 (-2.5, 9823.04)	1 (Reference)	1 (0.2)	0.82 (0.02, 4.56)	-0.48 (-4.71, 3432.13)	0.4 (0.04, 3.61)	0.378
Anus, Anal Canal and Anorectum	0 (0)	0 (0, 117.17)	-0.04 (-0.04, -50.79)	1 (Reference)	0 (0)	0 (0, 191.99)	-0.04 (-0.04, -37.54)	..	>.99
Liver and intrahepatic bile duct	4 (0.71)	4.43 (1.21, 11.34)	4.2 (-1.12, 11497.62)	1 (Reference)	1 (0.2)	2.01 (0.05, 11.22)	1.09 (-3.15, 4866.63)	0.4 (0.04, 3.58)	0.373
Gallbladder and other biliary	0 (0)	0 (0, 26.82)	-0.19 (-0.19, -226.12)	1 (Reference)	0 (0)	0 (0, 43.31)	-0.18 (-0.18, -167.95)	..	>.99
Pancreas	1 (0.18)	0.69 (0.02, 3.85)	-0.61 (-3.26, 2479.11)	1 (Reference)	2 (0.4)	2.3 (0.28, 8.3)	2.44 (-3.55, 7707.68)	2.9 (0.26, 32.76)	0.371
Retroperitoneum, peritoneum and other digestive system	3 (0.53)	76.24 (15.72, 222.79)	4.02 (-0.59, 10414.25)	1 (Reference)	2 (0.4)	82.11 (9.94, 296.61)	4.27 (-1.72, 9381.27)	0.79 (0.13, 4.76)	0.791
Lung and Bronchus	35 (6.23)	6.04 (4.21, 8.4)	39.61 (23.89, 66854.85)	1 (Reference)	20 (3.99)	6 (3.67, 9.27)	36 (17.07, 50253.68)	0.87 (0.5, 1.5)	0.605
Pleura, trachea and other respiratory	1 (0.18)	51.83 (1.31, 288.79)	1.33 (-1.33, 4818.94)	1 (Reference)	1 (0.2)	87.92 (2.23, 489.86)	2.14 (-2.1, 5826.91)	1.46 (0.09, 25.02)	0.793
Bones and Joints	7 (1.25)	150.91 (60.68, 310.94)	9.43 (2.4, 19891.32)	1 (Reference)	12 (2.4)	411.24 (212.49, 718.35)	25.85 (11.19, 37069.04)	2.37 (0.91, 6.19)	0.07
Soft Tissue including Heart	231 (41.1)	1497.88 (1310.93, 1704.01)	313.1 (272.7, 427061.15)	1 (Reference)	281 (56.09)	3018.67 (2676, 3393.04)	606.61 (535.66, 619961.64)	2.41 (2.01, 2.9)	<.001
Melanoma of the Skin	10 (1.78)	31.55 (15.13, 58.01)	13.13 (4.73, 26021.73)	1 (Reference)	5 (1)	27.21 (8.84, 63.51)	10.4 (0.94, 18175.64)	0.82 (0.28, 2.39)	0.71
Breast	0 (0)	0 (0, 2.77)	-1.81 (-1.81, -2182.53)	1 (Reference)	0 (0)	0 (0, 4.13)	-1.93 (-1.93, -1762.5)	..	>.99
Cervix Uteri	0 (0)	0 (0, 28.55)	-0.36 (-0.36, -206.43)	1 (Reference)	1 (0.2)	12.13 (0.31, 67.56)	4.17 (-4.74, 5274.93)	..	0.153
Corpus and Uterus, NOS	0 (0)	0 (0, 12.36)	-0.84 (-0.83, -476.88)	1 (Reference)	1 (0.2)	5.03 (0.13, 28)	3.64 (-5.27, 5060.48)	..	0.182
Ovary	2 (0.36)	4.38 (0.53, 15.82)	4.32 (-3.44, 6904.88)	1 (Reference)	0 (0)	0 (0, 12.47)	-1.35 (-1.35, -542.52)	..	0.162
Vagina, vulva and other female genital organs	0 (0)	0 (0, 57.08)	-0.18 (-0.18, -104.02)	1 (Reference)	0 (0)	0 (0, 80.88)	-0.21 (-0.21, -84.31)	..	>.99
Prostate	0 (0)	0 (0, 3.08)	-3.15 (-3.15, -2004.02)	1 (Reference)	1 (0.2)	1.28 (0.03, 7.12)	0.9 (-7.16, 4585.26)	..	0.172
Testis	0 (0)	0 (0, 338.44)	-0.03 (-0.03, -18.42)	1 (Reference)	0 (0)	0 (0, 477.05)	-0.03 (-0.03, -16.84)	..	>.99
Penis and other male genital organs	0 (0)	0 (0, 266.69)	-0.04 (-0.04, -23.44)	1 (Reference)	0 (0)	0 (0, 461.68)	-0.03 (-0.03, -16.84)	..	>.99
Urinary Bladder	2 (0.36)	3.4 (0.41, 12.27)	1.91 (-1.85, 6853.77)	1 (Reference)	1 (0.2)	2.72 (0.07, 15.15)	1.37 (-2.87, 5121.52)	0.83 (0.07, 9.32)	0.881
Kidney and Renal Pelvis	2 (0.36)	3.9 (0.47, 14.09)	2.02 (-1.74, 6978.3)	1 (Reference)	0 (0)	0 (0, 12.34)	-0.65 (-0.65, -590.79)	..	0.262
Ureter and other urinary organs	0 (0)	0 (0, 120.09)	-0.04 (-0.04, -50.79)	1 (Reference)	0 (0)	0 (0, 194.61)	-0.04 (-0.04, -37.54)	..	>.99
Eye and Orbit	1 (0.18)	94.3 (2.39, 525.39)	1.34 (-1.32, 4832.05)	1 (Reference)	0 (0)	0 (0, 595.4)	-0.01 (-0.01, -11.86)	..	0.239
Brain and Other Nervous System	14 (2.49)	26.48 (14.48, 44.43)	18.27 (8.32, 34089.22)	1 (Reference)	15 (2.99)	49.5 (27.71, 81.65)	31.74 (15.35, 44038.82)	1.5 (0.72, 3.13)	0.276
Thyroid	0 (0)	0 (0, 56.3)	-0.09 (-0.09, -108.14)	1 (Reference)	0 (0)	0 (0, 92)	-0.09 (-0.09, -79.04)	..	>.99
Other endocrine system	2 (0.36)	66.71 (8.08, 240.97)	2.67 (-1.09, 7769.72)	1 (Reference)	2 (0.4)	114.48 (13.86, 413.55)	4.28 (-1.7, 9395.1)	1.82 (0.25, 13.02)	0.553
Hodgkin Lymphoma	0 (0)	0 (0, 95.61)	-0.05 (-0.05, -63.9)	1 (Reference)	0 (0)	0 (0, 154.62)	-0.05 (-0.05, -47.42)	..	>.99
Non-Hodgkin Lymphoma	6 (1.07)	6.52 (2.39, 14.19)	6.89 (0.38, 16188.76)	1 (Reference)	2 (0.4)	3.51 (0.43, 12.69)	3.09 (-2.9, 8302.43)	0.51 (0.1, 2.51)	0.381
Myeloma	1 (0.18)	2.32 (0.06, 12.91)	0.77 (-1.89, 4142.23)	1 (Reference)	0 (0)	0 (0, 13.56)	-0.59 (-0.59, -537.44)	..	0.324
Acute Lymphocytic Leukemia	0 (0)	0 (0, 86.97)	-0.06 (-0.06, -68.82)	1 (Reference)	0 (0)	0 (0, 143.15)	-0.06 (-0.06, -51.37)	..	>.99
Acute Non-Lymphocytic Leukemia	0 (0)	0 (0, 8.8)	-0.57 (-0.57, -686.55)	1 (Reference)	0 (0)	0 (0, 14.85)	-0.54 (-0.54, -490.02)	..	>.99
Chronic Myeloid Leukemia	0 (0)	0 (0, 93.91)	-0.05 (-0.05, -63.9)	1 (Reference)	0 (0)	0 (0, 144.99)	-0.06 (-0.05, -49.4)	..	>.99
Others	51 (9.07)	27.4 (20.4, 36.02)	66.65 (47.66, 103451.09)	1 (Reference)	51 (10.18)	44.26 (32.96, 58.19)	107.65 (77.42, 126151.35)	1.58 (1.06, 2.36)	0.025
In situ, benign or unknown behavior neoplasm	32 (5.69)	59.17 (40.48, 83.54)	42.67 (27.63, 69713.97)	1 (Reference)	28 (5.59)	77.77 (51.68, 112.4)	59.69 (37.29, 75106.39)	1.33 (0.8, 2.22)	0.275
Septicemia	3 (0.53)	2.28 (0.47, 6.67)	2.29 (-2.32, 8325.12)	1 (Reference)	1 (0.2)	1.1 (0.03, 6.11)	0.19 (-4.04, 4044.66)	0.42 (0.04, 4.11)	0.432
Other Infectious and Parasitic Diseases including HIV	4 (0.71)	4.18 (1.14, 10.71)	4.13 (-1.19, 11410.78)	1 (Reference)	0 (0)	0 (0, 5.68)	-1.4 (-1.4, -1282.36)	..	0.024
Diabetes Mellitus	2 (0.36)	0.71 (0.09, 2.55)	-1.13 (-4.89, 3173.62)	1 (Reference)	2 (0.4)	1.09 (0.13, 3.93)	0.35 (-5.64, 5793.04)	1.44 (0.2, 10.3)	0.714
Alzheimer’s (ICD-9 and 10 only)	5 (0.89)	1.6 (0.52, 3.73)	2.54 (-3.4, 10253.47)	1 (Reference)	3 (0.6)	1.19 (0.25, 3.47)	1.03 (-6.31, 7646.35)	0.69 (0.16, 2.89)	0.602
CVD	45 (8.01)	1.52 (1.11, 2.03)	20.75 (2.92, 46613.27)	1 (Reference)	25 (4.99)	1.19 (0.77, 1.76)	8.69 (-12.47, 27316.74)	0.8 (0.49, 1.3)	0.358
Diseases of Heart	31 (5.52)	1.36 (0.93, 1.94)	11.2 (-3.6, 31412.12)	1 (Reference)	14 (2.79)	0.88 (0.48, 1.48)	-4.07 (-19.91, 10763.98)	0.65 (0.34, 1.22)	0.167
Hypertension without Heart Disease	2 (0.36)	1.92 (0.23, 6.93)	1.3 (-2.46, 6111.52)	1 (Reference)	2 (0.4)	2.53 (0.31, 9.13)	2.61 (-3.37, 7865.75)	1.19 (0.16, 8.63)	0.863
Cerebrovascular Diseases	9 (1.6)	1.83 (0.83, 3.46)	5.52 (-2.46, 16300.18)	1 (Reference)	9 (1.8)	2.5 (1.14, 4.74)	11.66 (-1.04, 22282.16)	1.52 (0.6, 3.83)	0.38
Atherosclerosis	1 (0.18)	3.95 (0.1, 22.03)	1.01 (-1.65, 4435.52)	1 (Reference)	0 (0)	0 (0, 19.05)	-0.42 (-0.42, -383.32)	..	0.273
Aortic Aneurysm and Dissection	2 (0.36)	5.02 (0.61, 18.12)	2.17 (-1.59, 7165.1)	1 (Reference)	0 (0)	0 (0, 14.13)	-0.56 (-0.56, -515.71)	..	0.148
Other Diseases of Arteries, Arterioles, Capillaries	0 (0)	0 (0, 11.1)	-0.45 (-0.45, -543.99)	1 (Reference)	0 (0)	0 (0, 15.39)	-0.52 (-0.52, -474.21)	..	>.99
Pneumonia and Influenza	7 (1.25)	3.53 (1.42, 7.28)	6.81 (-0.23, 16720.75)	1 (Reference)	4 (0.8)	2.79 (0.76, 7.14)	5.54 (-2.93, 12813.67)	0.84 (0.24, 2.88)	0.778
Chronic Obstructive Pulmonary Disease and Allied Cond	4 (0.71)	0.77 (0.21, 1.97)	-1.63 (-6.95, 4455.19)	1 (Reference)	3 (0.6)	0.89 (0.18, 2.61)	-0.77 (-8.1, 6004.39)	1.2 (0.27, 5.37)	0.815
Chronic Liver Disease and Cirrhosis	2 (0.36)	1.55 (0.19, 5.61)	0.97 (-2.79, 5710.07)	1 (Reference)	0 (0)	0 (0, 4.99)	-1.6 (-1.6, -1460.19)	..	0.175
Nephritis, Nephrotic Syndrome and Nephrosis	4 (0.71)	2.32 (0.63, 5.95)	3.09 (-2.23, 10155.66)	1 (Reference)	0 (0)	0 (0, 2.99)	-2.67 (-2.66, -2438.25)	..	0.038
Symptoms, Signs and Ill-Defined Conditions	4 (0.71)	3.85 (1.05, 9.86)	4.02 (-1.3, 11274.78)	1 (Reference)	2 (0.4)	2.49 (0.3, 9.01)	2.59 (-3.4, 7844.02)	0.76 (0.14, 4.25)	0.755
Accidents and Adverse Effects	5 (0.89)	1.23 (0.4, 2.88)	1.29 (-4.66, 8732.91)	1 (Reference)	1 (0.2)	0.36 (0.01, 2.01)	-3.83 (-8.06, 371.47)	0.26 (0.03, 2.26)	0.167
Suicide and Self-Inflicted Injury	3 (0.53)	2.52 (0.52, 7.37)	2.46 (-2.15, 8528.3)	1 (Reference)	0 (0)	0 (0, 4.81)	-1.66 (-1.66, -1515.51)	..	0.066
Other COD	55 (9.79)	3.76 (2.83, 4.89)	54.74 (35.02, 89942.24)	1 (Reference)	28 (5.59)	2.67 (1.78, 3.86)	37.84 (15.44, 55114.3)	0.72 (0.46, 1.14)	0.16
Syphilis	0 (0)	0 (0, 2679.14)	0 (0, -1.64)	1 (Reference)	0 (0)	0 (0, 3094.03)	0 (0, -1.98)	..	>.99
Tuberculosis	0 (0)	0 (0, 152.47)	-0.03 (-0.03, -39.32)	1 (Reference)	0 (0)	0 (0, 259.36)	-0.03 (-0.03, -27.66)	..	>.99
Stomach and Duodenal Ulcers	0 (0)	0 (0, 31.39)	-0.16 (-0.16, -193.35)	1 (Reference)	0 (0)	0 (0, 47.12)	-0.17 (-0.17, -154.12)	..	>.99
Puerperium	0 (0)	0 (0, 237.95)	-0.02 (-0.02, -26.22)	1 (Reference)	0 (0)	0 (0, 377.34)	-0.02 (-0.02, -19.76)	..	>.99
Congenital Anomalies	30 (5.34)	213.12 (143.79, 304.24)	40.5 (25.94, 66515.42)	1 (Reference)	14 (2.79)	170.03 (92.96, 285.27)	30.06 (14.22, 41991.01)	0.66 (0.35, 1.24)	0.186
Certain Conditions Originating in. Perinatal Period	0 (0)	0 (0, 153.36)	-0.03 (-0.03, -39.32)	1 (Reference)	0 (0)	0 (0, 1084.78)	-0.01 (-0.01, -5.93)	..	>.99
Homicide and Legal Intervention	0 (0)	0 (0, 8.32)	-0.6 (-0.6, -725.87)	1 (Reference)	0 (0)	0 (0, 11.84)	-0.67 (-0.67, -616.48)	..	>.99
Other Cause of Death	25 (4.45)	1.8 (1.17, 2.66)	15.09 (1.79, 34283.14)	1 (Reference)	14 (2.79)	1.4 (0.77, 2.35)	8.69 (-7.15, 22437.56)	0.8 (0.41, 1.53)	0.489

eTable55. Causes of death among survivors diagnosed with primary malignant peripheral nerve sheath tumor by chemotherapy during 2000-2019 in 17 SEER registers.

COD	No				Yes				*r heterogen*
	Obs.	SMR (95% CI)	Absolute excess incidence per 10,000 person-year	Relative risk (95%CI)	Obs.	SMR (95% CI)	Absolute excess incidence per 10,000 person-year	Relative risk (95%CI)	
All Cause of Death	727 (100)	5.07 (4.71, 5.45)	577.05 (524.79, 1023330.69)	1 (Reference)	336 (100)	35.31 (31.63, 39.29)	1726.78 (1536.76, 952708.71)	2.85 (2.48, 3.27)	<.001
All malignant cancers	503 (69.19)	15.66 (14.32, 17.09)	465.63 (422.16, 827847.27)	1 (Reference)	287 (85.42)	130.8 (116.1, 146.84)	1506.35 (1330.73, 835989.76)	3.48 (2.98, 4.06)	<.001
Head and Neck	7 (0.96)	9.39 (3.78, 19.35)	6.19 (1.06, 18396.32)	1 (Reference)	2 (0.6)	30.85 (3.74, 111.45)	10.24 (-4.43, 12373.45)	2.26 (0.46, 11.16)	0.353
Esophagus	0 (0)	0 (0, 4.43)	-0.82 (-0.82, -1337.84)	1 (Reference)	0 (0)	0 (0, 54.03)	-0.36 (-0.36, -178.76)	..	>.99
Stomach	4 (0.55)	6.01 (1.64, 15.4)	3.3 (-0.58, 11665.86)	1 (Reference)	1 (0.3)	19.93 (0.51, 111.06)	5.02 (-5.34, 7649.84)	4.45 (0.46, 42.58)	0.262
Small Intestine	0 (0)	0 (0, 48.41)	-0.08 (-0.08, -122.21)	1 (Reference)	0 (0)	0 (0, 678.94)	-0.03 (-0.03, -13.14)	..	>.99
Colon and Rectum	5 (0.69)	1.7 (0.55, 3.97)	2.04 (-2.29, 10362.91)	1 (Reference)	0 (0)	0 (0, 17.6)	-1.11 (-1.11, -552.05)	..	0.373
Anus, Anal Canal and Anorectum	0 (0)	0 (0, 79.11)	-0.05 (-0.05, -75.57)	1 (Reference)	0 (0)	0 (0, 906.99)	-0.02 (-0.02, -10.52)	..	>.99
Liver and intrahepatic bile duct	4 (0.55)	3.1 (0.84, 7.94)	2.68 (-1.2, 10659.27)	1 (Reference)	1 (0.3)	9.18 (0.23, 51.12)	4.71 (-5.65, 7494.74)	2.84 (0.3, 26.97)	0.411
Gallbladder and other biliary	0 (0)	0 (0, 17.62)	-0.21 (-0.21, -336.07)	1 (Reference)	0 (0)	0 (0, 276.99)	-0.07 (-0.07, -34.17)	..	>.99
Pancreas	3 (0.41)	1.38 (0.29, 4.04)	0.82 (-2.54, 6791.8)	1 (Reference)	0 (0)	0 (0, 25.07)	-0.78 (-0.78, -386.44)	..	0.647
Retroperitoneum, peritoneum and other digestive system	3 (0.41)	49.98 (10.31, 146.08)	2.91 (-0.45, 10186.24)	1 (Reference)	2 (0.6)	541.94 (65.63, 1957.69)	10.56 (-4.1, 12533.8)	7.93 (0.33, 188.54)	0.172
Lung and Bronchus	40 (5.5)	4.68 (3.34, 6.37)	31.09 (18.84, 70493.86)	1 (Reference)	15 (4.46)	26.35 (14.75, 43.46)	76.33 (36.18, 57891.82)	3.03 (1.62, 5.66)	0.001
Pleura, trachea and other respiratory	1 (0.14)	34.9 (0.88, 194.46)	0.96 (-0.98, 4712.98)	1 (Reference)	1 (0.3)	496.12 (12.56, 2764.19)	5.28 (-5.09, 7776.03)	14.98 (0.05, 4659.81)	0.287
Bones and Joints	14 (1.93)	206.46 (112.87, 346.4)	13.78 (6.52, 34194.64)	1 (Reference)	5 (1.49)	644.8 (209.37, 1504.75)	26.4 (3.22, 24644.3)	5.9 (1.7, 20.42)	0.009
Soft Tissue including Heart	293 (40.3)	1296.63 (1152.4, 1453.92)	289.51 (256.33, 524720.69)	1 (Reference)	219 (65.18)	10264.95 (8950.36, 11718.27)	1158.19 (1004.78, 651904.18)	5.53 (4.57, 6.7)	0
Melanoma of the Skin	13 (1.79)	28.04 (14.93, 47.95)	12.4 (5.41, 31520.95)	1 (Reference)	2 (0.6)	53.86 (6.52, 194.56)	10.38 (-4.28, 12447.05)	1.69 (0.37, 7.75)	0.525
Breast	0 (0)	0 (0, 1.77)	-2.06 (-2.06, -3342.98)	1 (Reference)	0 (0)	0 (0, 25.52)	-0.76 (-0.77, -381.18)	..	>.99
Cervix Uteri	1 (0.14)	5.18 (0.13, 28.85)	5.18 (0.13, 28.85)	1 (Reference)	0 (0)	0 (0, 199.24)	0 (0, 199.24)	..	0.622
Corpus and Uterus, NOS	1 (0.14)	2.14 (0.05, 11.91)	2.14 (0.05, 11.91)	1 (Reference)	0 (0)	0 (0, 125.19)	0 (0, 125.19)	..	0.823
Ovary	1 (0.14)	1.41 (0.04, 7.87)	1.41 (0.04, 7.87)	1 (Reference)	1 (0.3)	22.5 (0.57, 125.38)	22.5 (0.57, 125.38)	18.29 (0.88, 378.13)	0.085
Vagina, vulva and other female genital organs	0 (0)	0 (0, 35.32)	0 (0, 35.32)	1 (Reference)	0 (0)	0 (0, 637.11)	0 (0, 637.11)	..	>.99
Prostate	1 (0.14)	0.53 (0.01, 2.93)	-1.77 (-5.61, 1790.97)	1 (Reference)	0 (0)	0 (0, 47.06)	-0.69 (-0.69, -196.82)	..	0.71
Testis	0 (0)	0 (0, 242.41)	-0.03 (-0.03, -25.37)	1 (Reference)	0 (0)	0 (0, 1080.21)	-0.03 (-0.03, -7.57)	..	>.99
Penis and other male genital organs	0 (0)	0 (0, 182.19)	-0.04 (-0.04, -33.82)	1 (Reference)	0 (0)	0 (0, 2342.88)	-0.01 (-0.02, -5.05)	..	>.99
Urinary Bladder	3 (0.41)	3.29 (0.68, 9.62)	2.07 (-1.29, 8817.85)	1 (Reference)	0 (0)	0 (0, 81.07)	-0.24 (-0.24, -120.93)	..	0.577
Kidney and Renal Pelvis	1 (0.14)	1.32 (0.03, 7.36)	0.24 (-1.7, 3542.37)	1 (Reference)	1 (0.3)	18.16 (0.46, 101.19)	5 (-5.37, 7636.7)	9.26 (0.34, 252.03)	0.195
Ureter and other urinary organs	0 (0)	0 (0, 78.17)	-0.05 (-0.05, -75.57)	1 (Reference)	0 (0)	0 (0, 1487.14)	-0.01 (-0.01, -5.26)	..	>.99
Eye and Orbit	0 (0)	0 (0, 236.25)	-0.02 (-0.02, -25.73)	1 (Reference)	1 (0.3)	843.28 (21.35, 4698.45)	5.28 (-5.08, 7778.65)	..	0.239
Brain and Other Nervous System	24 (3.3)	31.59 (20.24, 47)	22.98 (13.49, 52809.13)	1 (Reference)	5 (1.49)	69.5 (22.57, 162.2)	26.07 (2.88, 24476.06)	1.24 (0.46, 3.35)	0.677
Thyroid	0 (0)	0 (0, 37.3)	-0.1 (-0.1, -159.19)	1 (Reference)	0 (0)	0 (0, 548.47)	-0.04 (-0.04, -18.4)	..	>.99
Other endocrine system	2 (0.28)	46.5 (5.63, 167.99)	1.94 (-0.81, 7603.89)	1 (Reference)	2 (0.6)	450.07 (54.51, 1625.79)	10.56 (-4.1, 12533.8)	311.52 (43.03, 2255.46)	<.001
Hodgkin Lymphoma	0 (0)	0 (0, 65.13)	-0.06 (-0.06, -91.65)	1 (Reference)	0 (0)	0 (0, 636.15)	-0.03 (-0.03, -15.77)	..	>.99
Non-Hodgkin Lymphoma	6 (0.83)	4.27 (1.57, 9.3)	4.55 (-0.2, 15110.15)	1 (Reference)	2 (0.6)	23.2 (2.81, 83.8)	10.12 (-4.54, 12318.24)	3.34 (0.61, 18.41)	0.205
Myeloma	1 (0.14)	1.51 (0.04, 8.39)	0.33 (-1.61, 3691.91)	1 (Reference)	0 (0)	0 (0, 93.34)	-0.21 (-0.21, -105.15)	..	0.62
Acute Lymphocytic Leukemia	0 (0)	0 (0, 61.14)	-0.06 (-0.06, -96.48)	1 (Reference)	0 (0)	0 (0, 470.31)	-0.04 (-0.04, -21.03)	..	>.99
Acute Non-Lymphocytic Leukemia	0 (0)	0 (0, 5.91)	-0.62 (-0.62, -1003.38)	1 (Reference)	0 (0)	0 (0, 85.03)	-0.23 (-0.23, -113.04)	..	>.99
Chronic Myeloid Leukemia	0 (0)	0 (0, 61.08)	-0.06 (-0.06, -96.48)	1 (Reference)	0 (0)	0 (0, 852.48)	-0.02 (-0.02, -10.52)	..	>.99
Others	75 (10.32)	26.51 (20.85, 33.23)	71.37 (54.58, 143343.14)	1 (Reference)	27 (8.04)	146.31 (96.42, 212.88)	141.83 (87.96, 97264.61)	2.25 (1.41, 3.6)	0.001
In situ, benign or unknown behavior neoplasm	42 (5.78)	49.17 (35.44, 66.47)	40.69 (28.13, 86586.67)	1 (Reference)	18 (5.36)	385.94 (228.73, 609.96)	94.96 (50.97, 69055.14)	2.64 (1.42, 4.91)	0.004
Septicemia	4 (0.55)	1.91 (0.52, 4.88)	1.88 (-2, 9360.03)	1 (Reference)	0 (0)	0 (0, 28.87)	-0.68 (-0.68, -336.49)	..	0.569
Other Infectious and Parasitic Diseases including HIV	4 (0.55)	2.8 (0.76, 7.18)	2.55 (-1.33, 10440.59)	1 (Reference)	0 (0)	0 (0, 20.69)	-0.94 (-0.94, -467.93)	..	0.12
Diabetes Mellitus	4 (0.55)	0.92 (0.25, 2.34)	-0.37 (-4.25, 5703.49)	1 (Reference)	0 (0)	0 (0, 12.22)	-1.6 (-1.6, -793.9)	..	0.485
Alzheimer's (ICD-9 and 10 only)	8 (1.1)	1.46 (0.63, 2.88)	2.49 (-2.99, 12964.65)	1 (Reference)	0 (0)	0 (0, 21.82)	-0.89 (-0.89, -444.27)	..	0.557
CVD	62 (8.53)	1.3 (0.99, 1.66)	13.95 (-1.31, 47499.69)	1 (Reference)	8 (2.38)	2.88 (1.24, 5.67)	27.6 (-1.72, 28290.53)	1.93 (0.89, 4.16)	0.121
Diseases of Heart	41 (5.64)	1.12 (0.81, 1.53)	4.49 (-7.92, 27475.68)	1 (Reference)	4 (1.19)	1.85 (0.5, 4.73)	9.7 (-11.03, 15128.81)	1.35 (0.47, 3.91)	0.589
Hypertension without Heart Disease	4 (0.55)	2.3 (0.63, 5.88)	2.23 (-1.64, 9932.47)	1 (Reference)	0 (0)	0 (0, 40.76)	-0.48 (-0.48, -239.22)	..	0.645
Cerebrovascular Diseases	14 (1.93)	1.73 (0.95, 2.9)	5.83 (-1.42, 21276.17)	1 (Reference)	4 (1.19)	9.24 (2.52, 23.66)	18.87 (-1.87, 19681.91)	4.15 (1.23, 14.02)	0.041
Atherosclerosis	1 (0.14)	2.36 (0.06, 13.17)	0.57 (-1.37, 4079.43)	1 (Reference)	0 (0)	0 (0, 157.5)	-0.12 (-0.12, -60.46)	..	0.686
Aortic Aneurysm and Dissection	2 (0.28)	3.23 (0.39, 11.68)	1.37 (-1.38, 6677.7)	1 (Reference)	0 (0)	0 (0, 89.42)	-0.22 (-0.22, -107.78)	..	0.707
Other Diseases of Arteries, Arterioles, Capillaries	0 (0)	0 (0, 6.79)	-0.54 (-0.54, -873.13)	1 (Reference)	0 (0)	0 (0, 126.91)	-0.15 (-0.15, -76.24)	..	>.99
Pneumonia and Influenza	11 (1.51)	3.39 (1.69, 6.07)	7.67 (1.24, 22924.24)	1 (Reference)	0 (0)	0 (0, 21.49)	-0.91 (-0.91, -452.16)	..	0.279
Chronic Obstructive Pulmonary Disease and Allied Cond	6 (0.83)	0.74 (0.27, 1.6)	-2.13 (-6.88, 4249.88)	1 (Reference)	1 (0.3)	2.51 (0.06, 13.97)	3.18 (-7.19, 6732.39)	2.1 (0.23, 19.17)	0.543
Chronic Liver Disease and Cirrhosis	2 (0.28)	1.11 (0.13, 4)	0.19 (-2.55, 4765.81)	1 (Reference)	0 (0)	0 (0, 16.91)	-1.15 (-1.15, -573.08)	..	0.521
Nephritis, Nephrotic Syndrome and Nephrosis	4 (0.55)	1.42 (0.39, 3.65)	1.18 (-2.7, 8218.36)	1 (Reference)	0 (0)	0 (0, 24.97)	-0.78 (-0.78, -389.06)	..	0.489
Symptoms, Signs and Ill-Defined Conditions	4 (0.55)	2.33 (0.63, 5.96)	2.26 (-1.62, 9971.06)	1 (Reference)	2 (0.6)	16.49 (2, 59.57)	9.94 (-4.72, 12226.23)	2.68 (0.42, 17.18)	0.32
Accidents and Adverse Effects	5 (0.69)	0.84 (0.27, 1.96)	-0.95 (-5.29, 5500.39)	1 (Reference)	1 (0.3)	1.16 (0.03, 6.46)	0.73 (-9.64, 5515.25)	1.14 (0.12, 10.56)	0.909
Suicide and Self-Inflicted Injury	3 (0.41)	1.82 (0.38, 5.32)	1.34 (-2.02, 7634.38)	1 (Reference)	0 (0)	0 (0, 11.91)	-1.64 (-1.64, -814.93)	..	0.222
Other COD	65 (8.94)	2.75 (2.12, 3.5)	40.89 (25.27, 91903.88)	1 (Reference)	18 (5.36)	12.2 (7.23, 19.29)	87.4 (43.42, 65301.2)	1.85 (1.06, 3.25)	0.04
Syphilis	0 (0)	0 (0, 1552.86)	0 (0, -3.22)	1 (Reference)	0 (0)	0 (0, 19053.72)	0 (0, 0)	..	>.99
Tuberculosis	0 (0)	0 (0, 103.82)	-0.04 (-0.04, -57.89)	1 (Reference)	0 (0)	0 (0, 1278.93)	-0.02 (-0.02, -7.89)	..	>.99
Stomach and Duodenal Ulcers	0 (0)	0 (0, 20.14)	-0.18 (-0.18, -294.26)	1 (Reference)	0 (0)	0 (0, 291.71)	-0.07 (-0.07, -34.17)	..	>.99
Puerperium	0 (0)	0 (0, 172.21)	-0.02 (-0.02, -33.77)	1 (Reference)	0 (0)	0 (0, 956.04)	-0.02 (-0.02, -10.52)	..	>.99
Congenital Anomalies	28 (3.85)	145.08 (96.41, 209.68)	27.5 (17.24, 61389.85)	1 (Reference)	16 (4.76)	531.33 (303.7, 862.85)	84.47 (43, 62592.01)	2.78 (1.44, 5.39)	0.004
Certain Conditions Originating in. Perinatal Period	0 (0)	0 (0, 203.26)	-0.02 (-0.02, -28.94)	1 (Reference)	0 (0)	0 (0, 396.41)	-0.05 (-0.05, -23.66)	..	>.99
Homicide and Legal Intervention	0 (0)	0 (0, 6.33)	-0.58 (-0.58, -937.45)	1 (Reference)	0 (0)	0 (0, 21.43)	-0.91 (-0.91, -452.16)	..	>.99
Other Cause of Death	37 (5.09)	1.64 (1.15, 2.26)	14.23 (2.44, 42307.79)	1 (Reference)	2 (0.6)	1.61 (0.2, 5.81)	4 (-10.66, 9274.08)	0.56 (0.13, 2.39)	0.392
